# Metaprotein expression modeling for label-free quantitative proteomics

**DOI:** 10.1186/1471-2105-13-74

**Published:** 2012-05-04

**Authors:** Joseph E Lucas, J Will Thompson, Laura G Dubois, Jeanette McCarthy, Hans Tillmann, Alexander Thompson, Norah Shire, Ron Hendrickson, Francisco Dieguez, Phyllis Goldman, Kathleen Schwarz, Keyur Patel, John McHutchison, M Arthur Moseley

**Affiliations:** 1 Institute for Genome Sciences and Policy, Duke University, Durham, NC, USA; 2 Gasteroenterology, Duke University School of Medicine, Durham, NC, USA; 3 Merck, Sharpe, and Dohme, Corp, Whitehouse Station, NJ, USA; 4 Johns Hopkins Children’s Center, Baltimore, MD, USA

**Keywords:** Proteomics, Factor, Hepatitis, Open platform, Statistics, Statistical model, Srm, Mrm

## Abstract

**Background:**

Label-free quantitative proteomics holds a great deal of promise for the future study of both medicine and biology. However, the data generated is extremely intricate in its correlation structure, and its proper analysis is complex. There are issues with missing identifications. There are high levels of correlation between many, but not all, of the peptides derived from the same protein. Additionally, there may be systematic shifts in the sensitivity of the machine between experiments or even through time within the duration of a single experiment.

**Results:**

We describe a hierarchical model for analyzing unbiased, label-free proteomics data which utilizes the covariance of peptide expression across samples as well as MS/MS-based identifications to group peptides—a strategy we call metaprotein expression modeling. Our metaprotein model acknowledges the possibility of misidentifications, post-translational modifications and systematic differences between samples due to changes in instrument sensitivity or differences in total protein concentration. In addition, our approach allows us to validate findings from unbiased, label-free proteomics experiments with further unbiased, label-free proteomics experiments. Finally, we demonstrate the clinical/translational utility of the model for building predictors capable of differentiating biological phenotypes as well as for validating those findings in the context of three novel cohorts of patients with Hepatitis C.

**Conclusions:**

Mass-spectrometry proteomics is quickly becoming a powerful tool for studying biological and translational questions. Making use of all of the information contained in a particular set of data will be critical to the success of those endeavors. Our proposed model represents an advance in the ability of statistical models of proteomic data to identify and utilize correlation between features. This allows validation of predictors without translation to targeted assays in addition to informing the choice of targets when it is appropriate to generate those assays.

## Background

### Mass spectrometry proteomics

The field of proteomics has made remarkable advances in analytical hardware and software which have provided increasingly sensitive and robust analyses on platforms capable of detecting low abundance proteins from complex mixtures, such as serum and cell lysates. The nanoscale liquid chromatography and mass spectrometry (LC-MS) proteomic technology, which has become the state-of-the-art for differential expression proteomic studies in most major laboratories around the world, typically uses a ‘bottom-up’ approach, where the samples are subjected to a total proteolytic digestion, and the peptide ‘surrogates’ of the protein are quantified and identified using tandem mass spectrometry. There are two general approaches used for bottom-up differential expression proteomics via LC-MS, those based on the use of stable isotope labeling or tagging of the peptides, and the so-called label-free methods (shotgun proteomics) [1-4]. Advances in both approaches have occurred in recent years such that currently both relative and absolute quantitation of proteins is possible from complex mixtures by either labeled or label-free methodologies [5].

Although a specific advantage of the labeling approaches is the ability to heavily fractionate the samples to “dig deeper” into the proteome while maintaining quantitative capabilities, extensive fractionation of the sample is often impractical in the context of a clinical study with tens or even hundreds of samples. The proteomics community has seen a significant increase in the use of the label free approach due to increased instrument stability and software sophistication, and it is emerging as the method of choice for larger clinically-based studies where use of the labeling strategies is impossible or impractical. In particular, an advantage of label-free strategies which measure area-under-the-curve (AUC) of the LC-MS peak is that any of a number of commercial or open-source software packages can be used to extract ion intensities from each individual analysis, and statistical analysis on the relative abundance of these ions can be performed even in the absence of a peptide identification. The ability to precisely and reproducibly quantify thousands of proteolytic peptides using the label-free method was demonstrated by Wang, et al. and has been since employed and reproduced in a number of laboratories [4,6].

Techniques for aggregating peptides into larger units generally revolve around protein identifications. A variety of approaches exist to combine individual peak areas to generate a relative or absolute aggregate expression levels. Once peptides are assigned to their parent proteins, using an algorithm such as ProteinProphet, either the peptide frequency of observation (ßpectral counting) or MS intensity is used to estimate protein abundance [7]. The spectral counting approaches have gained a fairly large degree of use in the community due to their ease of implementation, however they generally suffer from a limited dynamic range and they are insensitive to small changes in expression level due to the large number of species which have peptides observed only 1–3 times [5]. Label-free AUC approaches generally overcome these limitations by locating a peak in the retention-time and accurate-mass matrix using sophisticated software, and extracting the area under the LC-MS peak. An important characteristic of AUC label-free studies is that they need to be performed on high resolution instruments for the best results, which limits the application of this approach to more expensive QToF, FT-ICR, or Orbitrap instruments.

### Analysis of shotgun proteomics

Error in protein-level quantitation can first occur due to incorrect peptide identifications. Even at a relatively low peptide false-discovery rate (i.e. 1%), the fraction of proteins detected that contain at least one false peptide identification is much higher because multiple peptides match back to the same protein. If a false-positive peptide is included in the protein level quantitation it can cause increased error in the protein-level quantitation. This can be partially overcome utilizing only the highest-quality or best-ionizing peptides for protein quantitation, however in current implementations of “Top 3” quantitation, the individual peptide confidence is not utilized as an inclusion parameter [8,9]. A second type of error in protein quantitation occurs when many homologues share a common peptide. In this situation the protein grouping algorithm, such as ProteinProphet, makes an informed decision about which parent sequence a peptide belongs to and typically associates all of the peptide intensity to that parent sequence. This can deliver erroneous protein quantitation results when multiple homologues are present. A final challenge is with protein isoforms, post-translationally modified or proteolytically processed peptides, which may show a biologically relevant and different expression pattern than the proteotypic peptides. In these cases, they should not be grouped together with the other peptides for the purposes of modeling expression.

This paper describes a statistical model which is designed to allow the inclusion and modeling of correlation structure for the problem of differential expression in mass spectrometry proteomics. There are a number of different approaches designed for protein level quantitation. The simplest of these use direct summarization of all features/isotope groups/peptides that are identified for each protein, such as averaging or robust summarization based on quantiles [10], or averaging only the most abundant three peptides from a protein [8,9]. In addition to these algorithms, there are ANOVA approaches for protein quantitation [11] and differential expression [12,13]. These are regression models that variously include or exclude fixed effects for experimental group and random effects to handle repeated measurements of the same sample (technical replicates). All of these approaches rely on protein identifications and none make explicit use of correlations between isotope groups. While we do not consider the introduction of fixed effects for biological phenotypes or the introduction of random effects for cases in which we have replicate measurements from the same sample, the factor model we describe is a regression model. Therefore, one might introduce these features in a relatively straightforward way.

We present here a metaprotein classification approach which demonstrates the use of coexpression in addition to identification of peptides to assist in grouping with similarly-quantified peptides. We note here the specific use of the term metaprotein. This is because, while many metaproteins obtained from fitting our model are dominated by peptides from a single protein, it is entirely possible that a “metaprotein” is not representative of a single protein at all. Rather, a metaprotein may contain peptides from multiple proteins. In such a case, the metaprotein is representative of the activity of a pathway. The extent to which the model is able to distinguish individual proteins within a pathway will depend on the sample size of the data set, however, even for very large sample sizes this may be impossible for proteins that have highly correlated expression. We would argue that distinguishing these is somewhatacademic in this case, and that noting that they are highly correlated, which is a feature of our approach, offers advantages in terms of higher power in subsequent hypothesis testing and model fitting (because the resulting metaproteins will be more independent than the results of protein level quantitation). Our approach has the following features, some of which are shared by the models described above:

· Allows for the subtraction of large scale correlation structure between proteins that likely arise from technical rather than biological variability (batch effects).

· Appropriately models both identified and unidentified features of the LC-MS output

· Utilizes feature identifications from MS/MS spectra, but allows for the probability that some of those identifications will be incorrect

· Produces a full posterior distribution on the model parameters, which leads to the quantification of uncertainty in the results.

· Admits the possibility that sections of a protein will be post-translationally modified and therefore may not be representative of the expression pattern of the protein as a whole.

· Makes use of correlation structure across samples, which provides significant information about feature relationships that is unused in many other approaches.

· Can be used in the creation of predictive models based on multiple proteins, rather than just the enumeration of proteins associated with a particular outcome.

We recognize that there are many excellent approaches to modeling label-free proteomic data that share some of these properties, however our proposed model is unique in its ability to concurrently model all of them.

In addition to advances in statistical modeling of label-free, unbiased proteomics data, this paper presents a novel pre-clinical predictor of response to therapy in patients with Hepatitis C. Finally, we demonstrate the validation of that predictor in two separate cohorts. First, we show that the approach generates a predictor that is reproducible between two different labs that are utilizing entirely different mass spectrometry technologies. Second, we show that the predictor is able to accurately differentiate clinical responders from non-responders in a novel cohort of pediatric patients with Hepatitis C. In both cases, the predictor is based on unbiased, label-free mass spectrometry data. We are aware of one previous example of the validation of shotgun proteomics findings with further shotgun proteomics experiments [14], in which a number of individual peptides were validated as markers of central nervous system lymphoma. Standard practice is to validate using a targetted platform such as selected reaction monitoring (SRM). To the best of our knowledge, ours is the first example of the validation of a full predictor. Finally, we verify some of the key elements of the predictor in our original cohort using SRM.

## Results and discussion

### Metaprotein factor model

In order to estimate metaprotein abundance, we build our model from pre-processed data with intensity estimates aggregated to the isotope group level. In our modeling approach, we allow the possibility that an isotope group will be incorrectly identified, or be correctly identified, but have a pattern of expression that is distinct from the bulk of peptides from the corresponding protein. In practice, this new grouping approach often leads to metaproteins which may be dominated by isotope groups from a particular protein, but which contain isotope groups from other proteins as well.

Let *X* be a *P* × *N*-dimensional matrix consisting of measurements on *P* isotope groups across *N* samples. We utilize a modification of the latent factor model outlined previously in [15-18].

(1) $$X=\mu 1_{N}+A\Lambda^{\prime }+\epsilon$$

The *P-*dimensional vector *μ* has elements *μ*_*i*_ representing the mean expression of isotope group *i* and 1_*N*_ is a column vector of ones. The *N* × *K*-dimensional matrix Λ represents latent factors which will be learned from the data and *A* is a *P* × *K*-dimensional matrix of factor loadings with elements *a*_*i,k*_. The random variable *ϵ* is a *P* × *N* matrix of idiosyncratic noise.

Our goal is to estimate relative protein concentration from this model using the latent factors in Λ. Recall that we have identifications for some subset of the isotope groups. With this in mind, suppose we identify each column of *A* and the corresponding column of Λ with one identified protein. If we set *a*_*i,k*_ *= 1* when isotope group *i* is from a peptide identified as coming from protein *k* and *a*_*i,k*_ *=* 0 otherwise, then our model is describing the expression pattern of each isotope group as a noisy approximation of the expression pattern of the protein, where the protein is known.

Retaining, for the time being, the idea of fixing *a*_*i,k*_ in this way, we wish to handle the possibility of changing sensitivity and changing protein concentration from sample to sample. To account for this, we introduce an additional set of latent factors into equation 1.

(2) $$X=\mu 1_{n}+BH^{\prime }+A\Lambda^{\prime }+\epsilon$$

We now introduce latent factors *H* and factor loadings $B=(b_{i,j})$ where j=1⋯J which we use to account for systematic structure in the data that is sample specific. Because these features will span almost all peptides, we utilize a generic Gaussian prior for the elements of *B*.

(3) $$b_{i,j}∼N(m_{0},v_{0})$$

This distribution represents our belief that these effects span all isotope groups, but with varying effect sizes. This prior also minimizes identifiability issues between *B*, which is not sparse, and *A* which is very sparse with informative priors.

Rather than assigning isotope groups to factors based purely on identification, we want to utilize a prior on *A* that allows for possible post-translational modifications and misidentifications. With this in mind, we want to relax our strict assignment of zeros and ones in the loadings matrix *A*. Instead, our prior distribution for $a_{i,k}$ will reflect our level of certainty that we know which factor should represent the expression of this peptide. When we have an identification for peptide *i* and have mapped that peptide to protein *k*, our prior distribution will reflect an increased certainty that $a_{i,k}\neq 0$.

We introduce a *p-*dimensional vector of latent variables $\left(z_{i}\right)$ which identifies the non-zero column of *A* for each isotope group. When we have an identification that suggests that isotope group *i* comes from protein *k*, our prior distribution for $z_{i}$ is

(4) $$\begin{array}{c} z_{i}∼Multinomial(1,q_{i})\\ q_{i}∼Dir(\alpha_{0},\cdots ,\alpha_{0},\alpha_{k},\alpha_{0},\cdots \alpha_{0}) \end{array}$$

where $\alpha_{k}$ is substantially larger than $\alpha_{0}$ to reflect our prior belief that $z_{i}=k$. We default to $\alpha_{k}=500\cdot \alpha_{0}$, but have tried values from 100 through 1000 and these lead to only minor shifts in metaprotein membership. As the weight of this prior decreases we are decreasing the importance of identification information and placing progressively more importance on correlation structure. We find, for the Hepatitis application below, that the association of metaproteins with outcome doesn’t substantially change until we increase the weight of the identification data to very high levels. We find that using $\alpha_{0}=1$ leads to interpretable metaproteins without loss of association with the outcomes. For peptides which do not have identifications, we utilize an unbiased prior $z_{i}∼Dir(\alpha_{0})$. Because different peptides showing similar expression patterns may, nonetheless, show a different magnitude of expression of that pattern due to the relative sensitivity of the mass spectrometer for the peptide, we model each of the non-zero elements of *A* independently, such that $a_{i,k}∼N(m_{a},v_{a})$ when $z_{i}=k$ and $a_{i,k}=0$ otherwise.

There is not a specific threshold for determining the grouping of isotope groups into metaproteins. Instead, the assignment of an isotope group to a particular metaprotein is a function of the variance associated with that isotope group, the number of isotope groups already assigned to each metaprotein and the level of agreement between the expression pattern of the isotope group and that of the metaprotein. All of these things are estimated using MCMC within the context of the model fitting.

We note that, in the limit as $\alpha_{k}\to \infty$ we obtain an ANOVA model in which there is no uncertainty about which metaprotein each isotope group belongs to. This is a fixed effects model with feature-specific variance similar to Clough et al. [11]. That limiting model implies that identifications are assumed to be accurate and assumes that post-translational modifications are of minor importance. Clough et al. correctly point out that, by collecting features one obtains higher power for detecting associations versus simple tests of association with individual isotope groups. That model, as well as the one we present here, may be expanded with additional design vectors identifying experimental groups or particular interventions if so desired. These may be included as columns in *H* which are simply not updated in the MCMC (Markov chain Monte Carlo).

To complete the model specification, we assume a conjugate, row specific inverse gamma prior for the variance of *ϵ*. This is similar to the protein level aggregation model of [11], and allows differing variance estimates for each isotope group. Because we are working with a relatively large number of samples (and thereby have no issues with identifying variance), we use a prior with mean 1 and variance 100. We also assume that the individual columns of Λ arise from a uniform distribution on the *N*-dimensional sphere of radius $\sqrt{N}$. The model is fit via MCMC and the result of this fit is a set of draws from the posterior distribution of all of the model parameters. All prior distributions are conjugate, and therefore we may use Gibbs sampling to update the model parameters at each step of the MCMC. The data sets we are modeling have been aggregated at the isotope group level, and as such they have between 20 and 40 thousand measurements per sample. While our sampling scheme is able to fit this data in just a few hours on a desktop, we expect that some sort of parallel processing will be desirable for data that is aggregated at the feature level. We have tested our model on multiple simulated data sets of various sizes (both sample size and number of isotope groups) to verify the accuracy of the parameter recovery even in the presence of intentionally mislabeled isotope groups.

#### Overlapping peaks

By our assumption that each row of *A* have only one non-zero entry, we have restricted our peptides to belong to just one metaprotein. As an alternative, one might allow more than one non-zero element in each row of *A*. This is equivalent to assuming that more than one metaprotein is responsible for the expression pattern seen in a single peptide. This might occur in cases where multiple isotope groups have highly overlapping peaks or where multiple proteins have homologous regions that can give rise to the same peptide. Although the extent to which we see multiple isotope groups in a single peak is unclear, this type of structure can be accounted for with relaxed priors on *A*. If $a_{i,k}$ is an element of *A*, then a point mass mixture prior accomplishes our goals.

(5) $$a_{i,k}∼(1-q_{k})\delta_{0}(a_{i,k})+q_{k}N(a_{i,k}|m_{0},v_{0})$$

where $\delta_{0}$ is the distribution describing a point mass at 0. This distribution represents the prior belief that some, but not all, metaproteins will be required to describe the expression pattern of each isotope group. The normal distribution allows the magnitude of the effect to vary. For each of the metaproteins, we estimate the number of associated isotope groups by our prior distribution on the mixing probability $q_{k}$:

(6) $$q_{k}∼Be(\nu_{0},\gamma_{0})$$

This approach allows for the restriction on isotope group association with just one metaprotein to be relaxed. However, as the resolution of mass spectrometry increases, and as fractionation in multiple dimensions (such as 2D chromatography and ionmobility) makes the distinction between polypeptides clearer, this modification to the model will become less and less important. Further, experience suggests that the vast majority of measured peaks are single species. Because of this, the addition of features to deal with overlapping peaks can introduce more noise than it removes, particularly when the number of samples in the experiment (and therefore the amount of information available from the correlation structure) is limited.

### Features of the factor model

One of the strengths of our approach is the ability to collect isotope groups based not only on identifications, but also on their coexpression across samples. Perhaps the most common method for visualizing correlation structure in high-dimensional data is hierarchical clustering. However, this is most typically used as a visualization strategy and does not, by default, provide quantitative estimates of aggregate behavior. While it is possible to generate models based on what is visualized from hierarchical clustering, nearest centroids for example, these have not to our knowledge been published for proteomic data. In addition, there are questions surrounding how one might combine peptide identifications and correlation structure in a principaled way to jointly model all of the available information.

One can identify collections of coexpressed isotope groups as well as an approximation of the expression patterns of each group from our model based on posterior distributions of the model parameters. The posterior parameters of greatest interest will depend on the specific application, but often we will be most interested in the vector of factor memberships, *z*, which describes which peptides group together most often. In data sets intended for the generation of predictive models in clinical/translational studies (as well as other types of studies), we will be interested in Λ. The columns of this matrix define our estimates of the expression patterns of the metaproteins across our samples. These can be used to estimate fold change, or can be treated as independent variables in any type of model that is appropriate for the study.

**Figure 1:**
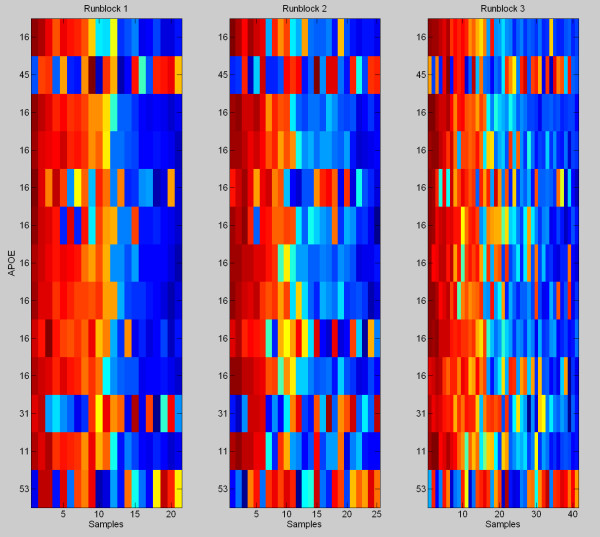
**Peptides from Apo E.** A heatmap of all of the isotope groups in the data set that are identified as originating from the protein Apoliprotein E. The numbers on the y-axis indicates which metaprotein that peptide was assigned to. The peptides are ordered from top to bottom from highest to lowest mean intensity across the samples. Note that, while the majority of those peptides share a common expression pattern (those assigned to metaprotein 16), three show a very different, conflicting pattern. Our meta-protein model automatically groups the co-expressing peptides into the same factor while assigning the peptides with conflicting patterns to meta-proteins that more closely match their expression patterns. Each row in each heatmap has been standardized to have mean zero and standard deviation one. Red is a relatively high level of expression and blue a low level of expression.

Associated with meta-protein *i* is a column of factor scores, Λ_*i*_, representing the expression of that meta-protein. In addition, there is a collection of isotope groups which make up that meta-protein, the isotope groups *j*for which $z_{j}=i$. Figure 1 shows a heatmap of all of the peptides from the dataset that are identified as belonging to the protein Apo E. Note that, while the majority of those peptides share a common expression pattern, three .(labeled 45, 31 and 53)show a very different, conflicting pattern. Our meta-protein model automatically groups the co-expressing peptides into the same factor while assigning the peptides with conflicting patterns to other meta-proteins that more closely match their expressionpatterns.

#### Inconsistent expression of peptides from the same protein

We define a “dominant metaprotein” for a protein to be the metaprotein(s) with more than half of its identified isotope groups contributed by that protein. We note that, because there are many metaproteins it is possible for a dominant metaprotein to consist largely (or even entirely) of peptides from one protein, but not contain all (or even a majority) of the isotope groups from that protein. One of the features we have observed from studying posterior parameters from our model is that there are manyexamples in which an identified isotope group (one with a peptide and protein label) does not follow the expression pattern of its corresponding dominant metaprotein. That is to say, for any given protein there is often a “consensus” expression pattern that many of the isotope groups from that protein follow, but that there is also a large minority of isotope groups which do not follow that expression pattern. We fit our model to all 109 proteins which have more than 1 identified peptide in the data set. Heatmaps similar to Figure 1 for each of these proteins are available in Additional file 1. Examination of these figures shows that the presence of peptides that show expression patterns significantly different from their corresponding dominant metaprotein is the rule, rather than the exception.

There are a few reasonable explanations for this. The most obvious possible explanation is that the poorly conforming peptides are those with the lowest overall intensity, and therefore subject to smaller signal to noise ratios. However, examination of heatmaps showing the exact same peptides, but now sorted by mean intensity across the samples rather than by meta-protein membership demonstrates that there is not a strong predominance of low intensity peptides among those that do not coexpress with the other peptides from the protein. All 109 of those heatmaps are available in the Additional file 2. We tested, using a non-parametric Kruskal-Wallis test, for association between meta-protein membership and mean signal intensity for each ofthe metaproteins, and found that, of the 109 proteins tested, only 2 showed significant association (p-value < 0.01, APOB and CERU).

Another possible explanation for the presence of poorly co-expressing peptides within a single protein is misalignment between runblocks. The data set was analyzed in three runblocks, one of which occurred months after the original two, and aligned according to the algorithm described in the methods section. There are sometimes shifts in retention time between runblocks which may lead to misalignment, however, we expect misalignment to be a rare occurrence. Additionally, if peptides are misaligned, we would expect to see a peptide that coexpresses with its dominant metaprotein well in two run blocks but is mismatched in the third, and this is not generally the case. We are able to find some examples that fit this pattern, however, it is almost always the case that when a peptide does not share an expression pattern with its dominant metaprotein one runblock, it also does not share that pattern in the other run blocks.

A third explanation for peptides that are uncorrelated is mis-identification. However, we are using identification algorithms with parameter settings that lead to very low (approximately 1%) false identifications. We expect around 34 such misidentifications, assuming that identification is correct 99% of the time (based on the 3398 total peptides with identifications). In fact, examining the list of peptides that do not belong to their dominant metaprotein, we see that more than half fall into this category (1640 out of 3398). This is true despite our prior distribution assigning a 500x greater likelihood of a peptide belonging to its dominant meta-protein than to a different metaprotein.

Post-translational modification of proteins is a well known process, but it is unclear how extensive these modifications are. If proteins are extensively and dynamically modified after translation, then we should expect many of them to exhibit expression patterns that do not match the bulk of peptides from that same protein. Also, if peptide modification is a significant contributor to observed patterns of expression, then we also expect to find peptides that have targets for post-translational modification to be more likely to be found outside their dominant meta-protein. We examined the probability of peptides containing Glutamine and Asparagine, which are known sites of deamidation, to belong to their dominant metaproteins. Correcting for the number of peptides inside and outside their dominant metaproteins, we find that peptides containing Glutamine are approximately 1.2 times more likely to not follow the dominant expression pattern for any given protein, and that this is a statistically significant difference (p-value .0013, fisher’s exact test). In addition, peptides with Asparagine are 1.22 times more likely to fail to coexpress with the dominant group of peptides from a protein (p-value 0.0010). In addition to these two sites ofpost-translational modification, we examined the motif NxS/T, which is a known site of N-linked glycosylation. For these two motifs, 25 of the 30 peptides which contain the “NxT” motif and 53 of the 60 peptides which contain the “NxS” motif follow expression patterns that are different from their dominant metaproteins (odds ratios 4.3 and 6.6 respectively, p-values 0.0013 and 2.1e-8 respectively). Additionally, both Serine and Threonine are known to be sites of O-linked glycosylationas well as phosphorylation. We find that both Threonine and Serine are also more likely to show odd expression patterns (Threonine: odds ratio 1.2, p-value .002 and Serine odds ratio 1.3, p-value 8.1e-6). We tested Proline (odds ratio = 1, *p* = .96) and Histidine (odds ratio = 1.1, *p* = .40) as negative controls.

We note that we are not directly detecting the post-translationally modified peptides. Instead, we suspect that we are detecting changes in the expression levels of the unmodified peptides due to post translational modification. Thus, peptides with any of these post-translational modification motifs are significantly less likely to follow the dominant expression pattern for the protein from which they are derived. It is possible that post-translational modification is a pervasive feature of plasma proteomics, and that protein level quantitation is likely to either introduce errors by summing across uncorrelated parts of a protein (if quantitation is accomplished through summation across all associated peptides) or to miss critical post-translational modifications (if quantitation is accomplished by summation of only the top three peptides). It is important to note, however, that inconsistent expression of peptides from the same protein is likely due to multiple different causes, including protein isoforms, increased noise in low abundance peptides, misalignment, mis-identification, peptide modification, and sample to sample variation in ion suppression from variable coelution in the LC separation. Regardless of the root cause, the statistical model we propose will identify isotope groups that show this characteristic and treat them appropriately.

#### Analysis of spike-in data: Relabeling of misidentified peptides

We obtained a publically available spike-in data set from [19] which consists of three replicate measurements of each of six conditions. The base solution is solid-phase N-glycocapture from human serum, and each of six different non-human proteins were spiked in at each of six different concentrations in a latin square design. After fitting our model to this data, we find that there is strong evidence for a significant increase in the total number of isotope groups that could be identified as coming from the known six spiked in proteins (Additional file 3). Estimated fold-change for each protein was also calculated for each experimental group and each spiked in protein. We find that, while the estimation works well for the highest four groups for each spiked in protein, the lowest two groups are typically underestimated due to the significant level of missing data.

In order to test the ability of our model to identify and correct inaccurate identifications, we randomly permuted 10% of the identifications in this data and refit our model. This experiment was repeated 100 times. Additional file 3 shows the results of this experiment for the ‘MYG_HORSE’ metaprotein for one such permutation. Note that there are 3 isotope groups that are correctly assigned to the MYG_HORSE metaprotein even though the original identification would have placed them in other proteins. This experiment was repeated 50 times, and of the 295 total reassigned identifications across those experiments, 223 were correctly reassigned to their original protein. Of the remainder, the peptide was fit to a noise factor 61 times and fit to some other protein 11 times. None were assigned to the protein associated with the incorrect relabel.

#### Comparison with protein level quantitation

While there are similarities between our meta-protein model and various techniques for protein level quantitation, the ability to group peptides based on co-expression across all samples, and therefore identify peptides that show evidence of post-translational modification is a critical difference that is shared by no protein level quantitation method that we are aware of. Nonetheless, it is interesting to compare our model to protein level quantitation algorithms. For this purpose, we will examine two such algorithms. The summation algorithm estimates protein level quantitation by summing total expression across all peptides from a protein. This algorithm automatically gives peptides with high intensity measurements a larger effect on the estimated protein level quantitation. A second algorithm, Top 3, estimates the protein level expression as the mean of the three peptides with the highest intensity (average across the samples). These two algorithms typically give similar results, however, because peptides from the same protein do not always show consistent expression patterns, these approaches can lead to protein level quantitation that is unnecessarily noisy.

In general, we find that the correlation between our metaprotein model and protein level quantitation for estimation is high when there are a large number of peptides from the given protein. For example, one of the most abundant proteins in this data set is Apolipoprotein B (represented by 409 isotope groups), and correlation between estimated expression from our factor model and from summation of all Apo B identified peptides is 0.97. However, when there are fewer peptides available or if there are many misidentifications or modifications, we find evidence that our factor model gives improved estimation of protein level expression patterns. For example, pregnancy zone protein, which has only three associated isotope groups in the data set, is known to be over expressed in women compared to men. While both the metaprotein model and the summation algorithm show differential expression for this protein, the factor model gives a *p*-value of 4.5e-4 (statistically significant even after Bonferroni correction for multiple hypotheses) as compared to a *p*-value of .0014 for estimation by summation over identified peptides (not significant after multiple hypothesis correction).

**Figure 2:**
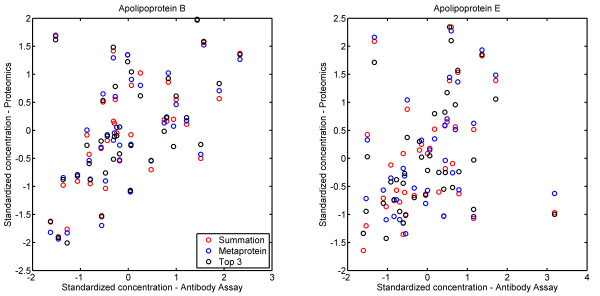
**Comparison to antibody assay.** A comparison between the antibody assay estimate of the protein level expression patterns for Apo B (panel A) and Apo E (panel B) (x-axes in both figures), and the LC-MS/MS estimates using the summation, top 3 and metaprotein methods for protein level expression. We note that the three algorithms are in agreement to a high degree. Correlations for Apo B are .55, .54 and .56 for Metaprotein, Summation and Top 3 models respectively and they are .31, .28 and .32 for respectively for Apo E. Note that there is an outlier in each of the top left and bottom right of the Apo E figure, and without these two outliers, correlations are just under .6 for all three methods. Antibody assay values, which measure protein per volume, were converted to protein per total protein using total protein levels from a Bradford assay. All measurements were standardized to mean zero and standard deviation 1.

Of the 96 subjects in this study, we have available antibody assay mesurements of both Apo B and Apo E on 38. We compared the two protein level quantitation algorithms and our metaprotein model to the antibody assay “gold standard”. Correlationsare all generally high and examination of Figure 2 shows that the three techniques are generally in agreement, even on outliers. The top three isotope groups identified as coming from Apo B show a high level of correlation, and all of these peptides are members of the main Apo B metaprotein. Also, a large majority of the Apo B peptides show this same expression pattern and are assigned to the same metaprotein, thus agreement between the three methods on Apo B is not surprising.

However, examination of the top three isotope groups from Apo E (Figure 1) shows a different picture. The second most abundant isotope group from Apo E is in a different metaprotein because it shows a substantially different expression pattern from the bulk of the Apo E isotope groups. In addition, if we delete the two outliers from the data (they are outliers by all three quantitation methods), the correlations between the three top Apo E isotope groups and the antibody assay of Apo E activity are.59, .23 and .56 respectively (*p*-values of .0002, .17 and .0004 respectively). Thus, this second most abundant isotope group should be adding noise to the Top 3 protein level estimate of Apo E. Interestingly, the correlation between the Top 3 estimate and the antibody assay is .60. This is higher than any of the three separately, which suggests that the antibody assay is in fact measuring an aggregation of two different forms of Apo E.

### Metaprotein expression in a hepatitis C cohort

In addition to the analysis of a publically available latin square data set (Additional file 3), we obtained pre-treatment serum samples from 96 patients with Hepatitis C who have a known response or non-response to the standard of care treatment with interferon and Ribavirin [20]. Serum from the patients was measured with open platform LC-MS/MS. The overall goal of the study was to predict who among the study subjects will respond to therapy and who will not. We are also interested in estimating which proteins and peptides are potential markers of response, allowing for future, targeted assay development.

Analysis of this data set proceeds in two steps. First, the model described above is fit to the proteomic data without regard to the phenotype of interest. There were a total of 6,729 peptides in the data set with either positive identifications or with average expression levels greater than the mean. Of these 3390 had identifications. These were matched with 265 different proteins of which 109 had two or more associated, identified peptides.

#### Prediction of outcome

As with the computation of protein level expression, our 109 metaproteins may be used in any context as independent predictor variables. Additionally, we may assess the level of association between metaproteins and other biological phenotypes in either case.

**Figure 3:**
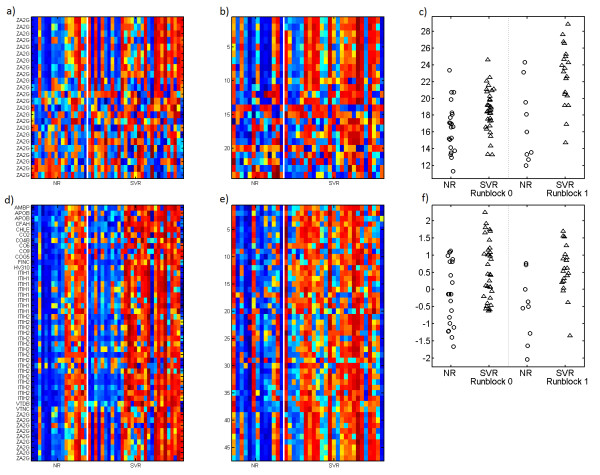
**Comparison to protein summarization.** Comparison of metaprotein and summation approaches to aggregation of large numbers of isotope groups. Panels **a** (runblock 0) and **b** (runblock 1) show all of the isotope groups identified as coming from the protein ZA2G. This is one of the two proteins significantly associated with the response to therapy phenotype in patients with Hepatitis C. Samples are on the x-axis and isotope groups are on the y-axis. Samples are labeled NR (non-responder) and SVR (sustained viral response). Panels **d** and **e** show the same type of heatmap, but now for the metaprotein containing the largest numbers of isotope groups from ZA2G. Notice that there are a number of isotope groups from other proteins that are highly correlated with the included ZA2G isotope groups and are therefore included in this metaprotein. Also, as can be seen in **a** and **b**, correlation of individual isotope groups from the ZA2G protein is high for about half of the isotope groups, but quite poor for many of the others. Panel **c** shows the predictive performance of the summation algorithm of “protein” level quantitation for ZA2G, split by runblock, and panel **f** shows the same for the metaprotein. Note that the metaprotein shows better performance and that the performance of the metaprotein is more consistent across the two separate runblocks. Performance of the top 3 algorithm for protein level quantitation is not shown because it is not statistically significantly associated with response to therapy. Each row in each heatmap has been standardized to have mean zero and standard deviation one. Red is a relatively high level of expression and blue a low level of expression.

Our first step in the analysis of the posterior distribution involves comparing the mean metaprotein expression patterns for all metaproteins to the “response to therapy” phenotype. We find three such metaproteins to be significantly associated (ANOVA *p*-value <9.8*e* − 5) even after correction for multiple hypothesis testing (ZA2G, ITIH and HRG). Protein level quantitation by summation yields only 2 and protein quantitation by top 3 yields zero. Furthermore, the relevant expression pattern is far clearer for the metaprotein analysis. Figure 3 shows the most predictive “protein” (ZA2G), as computed by the summation protein quantitation algorithm. Examination of the metaprotein with the most peptides from ZA2G, built from our model, shows that ZA2G is indeed identified as predictive by the metaprotein model as well. However, our metaprotein model identifies only those peptides from ZA2G that are highly correlated with each other, and it additionally identifies a number of other peptides from other proteins that share the same expression pattern across the samples (also shown in Figure 3). The result is better separation between responders and non-responders.

In addition to strong associations for three metaproteins, we find that there are a total of 13 metaproteins with *p*-values less than .01 (random association would dictate only 1). Thus there is clear evidence of the presence of blood-borne markers of response to therapy in Hepatitis C.

#### Identification of candidate peptides

We would like to identify a set of candidate peptides for use in future targeted studies such as selected reaction monitoring (SRM) or antibody studies. From the analysis of associations between averaged metaproteins and the phenotype, we are confident that there are markers of interest. In order to identify the most relevant isotope groups, we propose to obtain draws from the MCMC chain, and for each draw build a predictor and observe which peptides are included in that predictor. In this way, the values of Λ are computed directly from the peptides included in the corresponding metaprotein. Additionally, by keeping track of which peptides are most often included in the predictors, we obtain a list of candidate biomarkers for future study.

Because we have 109 metaproteins but only 87 samples, direct regression is not possible in this context. We instead use variable selection with model averaging. Variable selection allows regression with a small subset of the total number of predictors, while model averaging allows us to properly account for uncertainty in which models are correct. In this context, model averaging has been shown to outperform the single best model for predictive accuracy on hold out data sets [21]. We use a publicly available implementation of variable selection and model averaging called Shotgun Stochastic Search [22].

**Figure 4:**
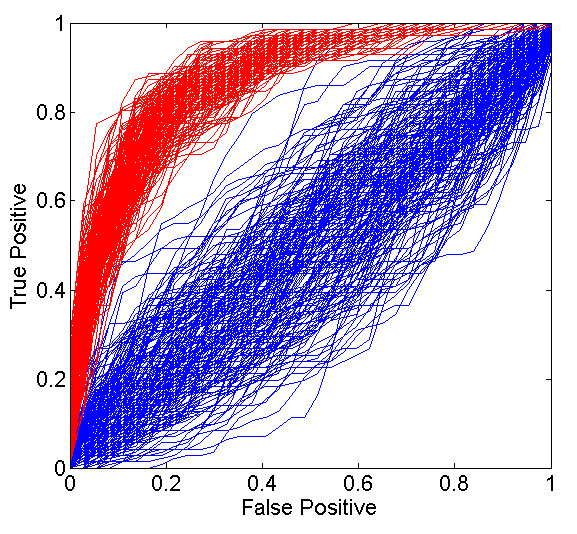
**Simulation ROC curves.** Receiver operating curves generated from 200 draws from the MCMC chain (red) compared to ROC’s generated from predictors of randomly generated outcomes (blue). The random predictors were generated from a mean zero multivariate normal distribution using a covariance matrix generated from the factor matrix Λ for the corresponding MCMC draw. The outcome variable (response/non-response) was then permuted before the ‘random predictor’ model was fit.

**Figure 5:**
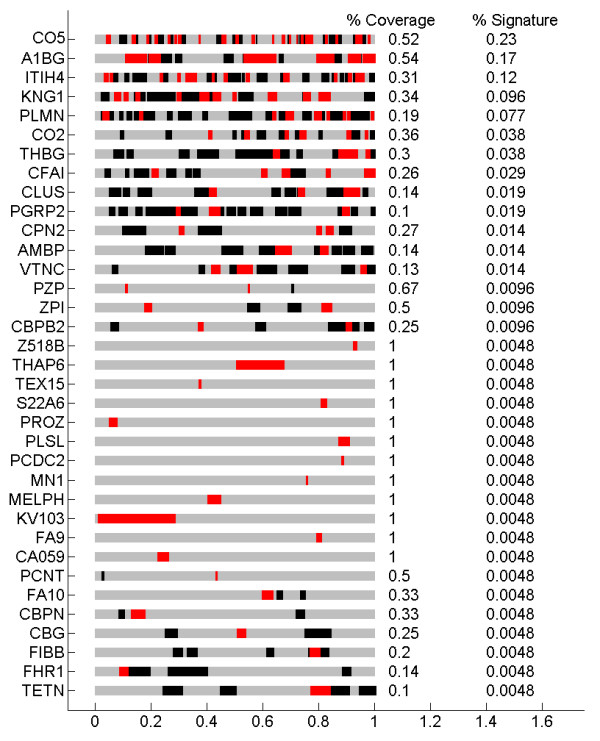
**Predictor content.** Shows the locations of the 600 most used peptides which were also identified. Proteins with fewer than 1% of their peptides represented in the figure were filtered out.

As mentioned above, our analysis consists of two steps. The first is the generation of a factor model to explain the variation seen in the peptide concentration data. This step is unsupervised; it does not take into account any phenotype data in any way. In this step we are seeking only to describe correlation structure in the isotope groups. In principal, if there were another large open platform plasma proteomics data set available this step could be performed on a different data set entirely. Prediction of the phenotype from the metaprotein expression through the use of variable selection and model averaging in a binary regression model constitutes the second, supervised step. Results from this analysis will vary slightly at each step of the MCMC chain. This allows us to estimate both the accuracy of predictors generated by this model as well as the uncertainty in that accuracy. Figure 4 shows receiver operating curves (ROC) of the model for 200 steps of the MCMC, and compares this to the same ROC’s from predicting randomly generated phenotypes with the same number of cases and controls. We see that the accuracy is significantly better than chance for all 200 draws from the Markov chain. The collection of most used isotope groups in this modeling approach are shown in Figure 5. We show the locations of the associated peptides (those with identifications) in their respective proteins.

#### Validation of a predictor discovered in an unbiased, label-free data set

There are two significant challenges facing users of label-free, unbiased, mass spectrometry proteomics. First, there are a number of different mass spectrometry machines, and some utilize different physical principals in order to measure mass-to-charge ratio. Differences in these machines and differences in protocols between laboratories can make reproducing the same results at different labs difficult—even when the samples used are exactly the same. Second, validation of findings in new samples can be difficult, particularly when the validation approach involves the use of different sample preparation or different techniques for measuring the peptides or proteins. It is quite common to see two phase experimental designs in which candidate peptides/proteins are identified by shotgun mass spectrometry then those candidates are validated on a much larger group of samples, but using antibody assays or SRM mass spectrometry. These approaches introduce additional variables in the sample preparation or measurement, and in cases where validation fails the explanation for that failure becomes uncertain—it may have failed because the peptides/proteins are not good candidates or because of the changes in the way they were measured.

We validated our results on the predictor of response to therapy by both testing the consistency of the predictor when measured in different labs and also when testing the accuracy of the predictor in a new set of samples. In each case, we are attempting to validate our predictor through the use of an additional unbiased, label-free data set. Because of difficulties with alignment between data sets, this approach is challenging and is usually not taken. The approach to this type of validation undertaken successfully by [14] involved the validation of multiple individual makers of disease. That approach produces a set of putative biomarkers, some of which validate in the follow-up data and some of which do not. It leads to a more highly filtered, and therefore more likely to succeed, list of biomarkers, but it does not lead to a validated predictor. Validation of a such a predictor must take into account uncertainties associated with alignment and false discovery. The advantage of our approach for this purpose is in the use of factors as predictors rather than individual isotope groups. By aggregating multiple isotope groups and using the aggregated expression pattern, the metaprotein model produces predictions that are robust to misalignments or false discovery associated with individual analytes.

**Figure 6:**
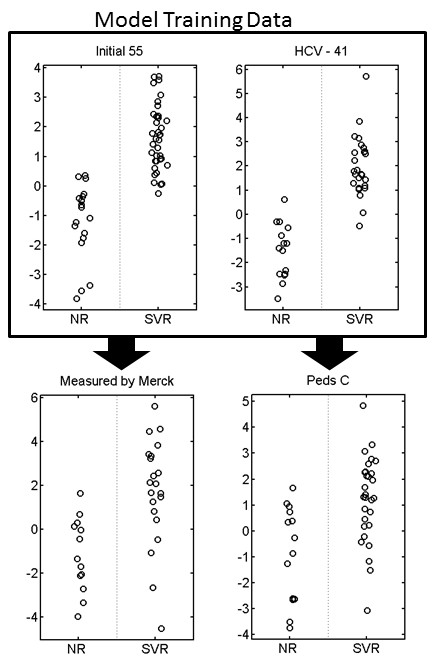
**Validation.** Validation of a predictor of response to therapy. A metaprotein model was fit to the training data (outlined). This model was then used to predict response to therapy in a subset of the training samples that was measured in a different lab using a different machine and different protocols. In addition, it was used to predict response to therapy in a new set of pediatric patients. In both cases, the validation was successful. For the Peds C cohort, the Area under the ROC curve is .8 and the p*-*value is 4.4 × 10^−4^.

We reanalyzed 28 of our original samples in an independent laboratory on a different mass spectrometry machine using a different technology (orbitrap versus time of flight). Those samples were analyzed using the protocols of the independent lab without modification. In addition to this, 51 additional samples were obtained from a pediatric cohort [23], and the predictor was evaluated on this cohort. All data sets were aligned to each other (as described in methods), and a predictor was built using our original data (as described above). This predictor was then tested for accuracy in each of the two additional data sets. Figure 6 shows that in both the independent laboratory measurement and the independent validation were successful. The area under the receiver operating characteristic (ROC) curve for the new, untrained pediatric patients was .8 and the predictor was significantly different between patients with a sustained viral response compared to those who did not respond to therapy (*p*-value 4.4 × 10^−4^ by *t*-test). These two results represent a validation of a clinically relevant predictor of disease state from label-free, unbiased mass spectrometry by further label-free, unbiased mass spectrometry.

#### Verification of differential expression for individual peptides

**Figure 7:**
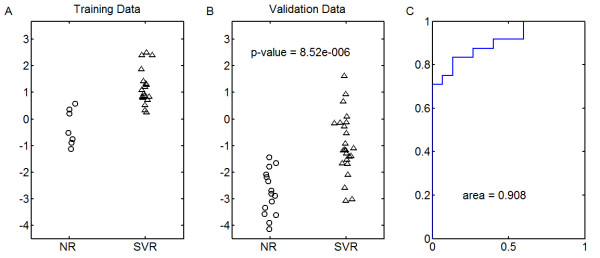
**Verification of predictor.** Predictive accuracy of the SRM assay for predicting response to therapy in Hepatitis C. Training was on the initial 25 samples, and validation was on the followup 38. Panel **a** shows behavior of the model on the trained samples and panel **b** shows behavior on the hold-out samples. The ROC curve in panel **c** shows accuracy only on the hold-out samples.

In order to verify our findings, we identified 87 peptides which were subsequently targeted for quantitation with selected reaction monitoring (SRM) without the addition of stable-labeled peptides, similar to the LF SRM method described in [24]. Of the samples that were measured with the shotgun proteomics approach, 25 from the first two run blocks (55 samples) were measured as well as 38 from the third run block (41 samples). In order to test whether we can generate predictors of response to therapy in patients with Hepatitis C, we trained a regression model on the 25 and validated on the remaining 38. All samples were used in the selection of peptides to target with SRM, therefore this does not constitute a true validation of an SRM based predictor. However Figure 7 shows that our predictive accuracy on the held out samples is quite good. This verifies, on the same samples and for a subset of the peptides, that our initial findings in the open platform can be reproduced by measurements of individual peptides. Because we utilized the shotgun proteomics results from all samples to select isotope groups for SRM, this does not address the question of out of sample accuracy, however, we have addressed this issue separately in Patel et al. [20].

**Figure 8:**
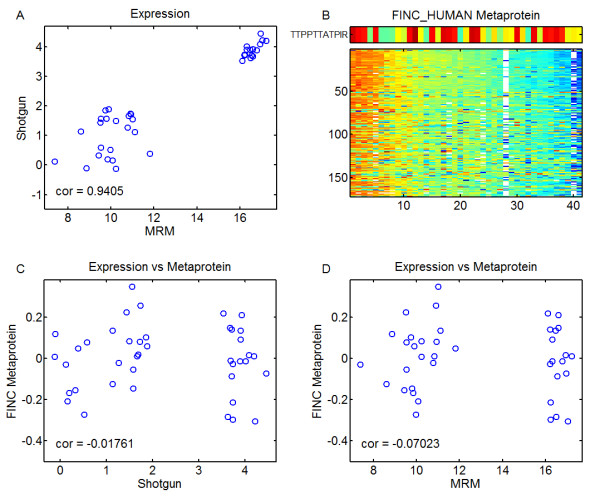
**Verification of poor coherence.** Peptide that doesn’t follow the pattern of it’s dominant metaprotein. Panel **a** shows the correlation between the measured intensity of the polypeptide TTPPTTATPIR from the label-free, unbiased platform compared to the label-free SRM platform. The high level of correlation demonstrates the reproducibility of measurement of this peptide. Panel **b** shows the pattern of expression, as measured in the unbiased platform, as compared to the pattern of expression of the FINC metaprotein. Panel **c** shows a scatterplot comparing the pattern of expression of the FINC metaprotein and the intensity of the peptide as measured with the shotgun approach and panel **d** shows the same comparison, but now with the SRM approach instead of the shotgun approach. The peptideis measured consistently and it does not correlate with the expression pattern of the FINC metaprotein.

In addition to biomarker verification, we sought to validate some examples of isotope groups which were determined not to follow their dominant metaprotein during the analysis of the original shotgun proteomics assay. An example is shown in Figure 8. The polypeptide “TTPPTTATPIR” from the protein FINC, which is known to be potentially O-glycoslyated at two residues [25], has a consistently-measured expression pattern on both SRM and QToF platforms (Figure 8a). This peptide exemplifies the type of peptide the Metaprotein approach would not group into the dominant metaprotein for FINC, since it does not correlate with the expression pattern of other FINC peptides (Figure 8b) or the FINC metaprotein (Figure 8c and 8d). Intensity data from the SRM experiment is available in Additional file 4.

## Conclusions

To date, the algorithms and models for aggregating mass spectrometry features in larger groups than peptides have relied on protein identifications and do not use correlations across samples in any way. The model we have described makes use of these correlations to identify peptides that do indeed show consistent expression in addition to agreement in identification. This paper describes both a novel statistical approach for the analysis of label-free, unbiased mass spectrometry proteomics and an approach for the validation of discoveries on that platform by further experiments on the same platform. Furthermore, we have demonstrated the application of this approach in the context of the treatment of Hepatitis C, which is a disease causing significant morbidity and mortality worldwide. The predictor of response to therapy described in this paper has the potential to significantly affect therapeutic decision making.

The model offers an approach to aggregation of mass spectrometry proteomics data that differs from protein level quantitation, and that in some situations, offers significant advantages over previous approaches. The critical difference between protein level quantitation and our metaprotein model is the inclusion of correlation structure in the metaprotein model. We should note that the inclusion of correlation structure may offer only minimal advantage low sample size situations. Additionally, in cases where there is an overwhelming biological effect, that effect can dominate the observed correlations and drown out what would otherwise be differing expression patterns. However, we have demonstrated the utility of the approach for validation studies and for potential clinical applications.

Our model is particularly well suited to situations in which we have a relatively large number of samples (>30) with a high degree of biological variability. We have shown that it can be used to gain insights into translational studies, and have exemplified its use with a study of response to therapy in patients with Hepatitis C. The model is appropriate for any high-quality quantitative (area-under-the-curve) proteomics data. This is not limited to data-independent acquisitions, rather the approach can be utilized on any label-free quantitative data which is based on precursor intensity for the quantitation and collects enough points across the peak to accurately define the peak area.

## Methods

### Sample selection

Chronic hepatitis C (HCV) patients, n = 96, were selected for proteomic analysis from the Duke Hepatology Database and Biorepository, as detailed previously [20]. Samples analyzed were all pre-treatment serum aliquots, but patients were classified based on their sustained response to Pegylated Interferon/Ribavirin combination therapy, the standard of care for HCV. Patients were matched as well as possible on the basis of relevant clinical parameters, including gender, viral load, metavir fibrosis score, and race (Table 1). Patients were divided between Genotype 1 HCV non-responders (n = 42), Genotype 1 HCV responders (n = 34), and Genotype 2/3 HCV responders (n = 20). A subset of this cohort, including 25 of the original 55 and 38 of the subsequent 41 samples, were also utilized for label-free SRM analysis.

**Table 1:** Demographics of the training data

	Responders	Non-responders
Gender	13 female, 24 male	4 female, 14 male
Race	50 caucasian, 11 AA	23 caucasian, 8 AA
Log-viral load	14 ± 3.7	15.6 ± 1.6
Metavir	1.6 ± 1.7	1.7 ± 2.3

### Sample preparation, instrument operation, and data preparation

The plasma sample preparation by immunodepletion and trypsin digestion, as well as the unbiased LC-MSE data collection on a nano Acquity and QToF Premier mass spectrometer (Waters Corporation), has been described in detail previously. [20]. Unbiased proteomic data analysis using label-free area-under-the-curve quantitation was performed in Rosetta Elucidator, utilizing both Mascot (Matrix Sciences, Inc) and PLGS v2.4 (Waters Corporation) and exported for statistical analysis, also as previously described [20]. Peptide annotation for this dataset was performed at a 1 peptide FDR using decoy database validation. To enable external analysis of the quantitative data using alternative methods, we have made the two unbiased quantitative data sets available (see Additional files 4 and 5). LC-SRM analyses were performed using a subset of the same samples, which were stored at −80 C between the initial unbiased analyses and the targeted analysis. A scheduled SRM method was generated in Skyline v0.7 (https://brendanx-uw1.gs.washington.edu/labkey/project/home/software/Skyline/begin.view) for 82 of the most promising biomarker peptides, and 5 peptides from yeast alcohol dehydrogenase (ADH1_YEAST) were also included as internal standards. The method included up to 3 transitions per precursor ion, and the MS method details as well as quantitative data have been included in Additional file 4. Analyses were performed on a nanoAcquity UPLC system coupled to a Xevo TQ mass spectrometer (Waters Corporation), using a method identical to that described for the unbiased data, with the following exceptions. The gradient length was 30 min, the column utilized was a 75 μm x 150 mm BEH, the flow rate was 0.4 μL/min and column temperature was 35°C. Raw data was imported into Skyline, quantified using a label-free approach, and exported for statistical analysis.

### Preparation of data for analysis

To accomplish data alignment and feature quantitation across all biological samples and thus form the matrix discussed in the statistical methods section below, we utilized Rosetta Elucidator™v3.3 software package (Rosetta Biosoftware) to import and align all MSE and data-dependent acquisition (DDA) raw data files [26-30]. Database searches were performed against a forward/reverse Swissprot database (v 56.5) with human taxonomy, using ProteinLynx Global Server v2.4 (IdentityE algorithm, Waters Corporation) for MSE searches or Mascot v2.2 for DDA data. Database searches are either performed externally and results imported (PLGS 2.4) or queued directly from within Elucidator (Mascot) to allow identification of many of the quantified features in the proteomic dataset. All database searches were performed with high mass accuracy on precursor and product ions (typically 20 ppm precursor and 0.04 Da product ion tolerance), with fixed carbamidomethylation(Cys), variable oxidation(Met) and variable deamidation(Asn and Gln). Annotation of the peptides is accomplished at an estimated 1% FDR using the Elucidator implementation of PeptideProphet algorithm [31]. Visual scripting within Elucidator is utilized to extract feature intensities for those features which have quantitative values above the 1000 counts (approximately 10th percentile) in 50% of the samples. The final file for statistical analysis is made up of a matrix of intensities, with the rows corresponding to isotope groups and the columns to technical observations (LC-MS analysis). An isotope group is defined as all of the peaks associated with a single peptide at a specific charge state and retention time. This level of quantitation combines peaks from the same peptide that differ according to the number of carbon 13’s incorporated, but does not combine the same peptide measured at different charge states. The intensity of an isotope group for a given sample is the total volume under the feature peaks associated with that isotope group. This is monotonically related to the concentration of that isotope group in the original sample, and it is these intensities that we work with. We have made the matrices of intensity values associated with the study available in Additional file 5.

### Technical variation

This data set was collected in three run blocks. Two of these were consecutive and the third was run months later. There are significant batch effects present in comparing the first two to the third even after correcting for observed total protein. Even though we did not include explicit design vectors for batch in our regression, our inclusion of a factor matrix describing systematic effects (*β* and *H*) allows this source of technical variation to be automatically modeled without foreknowledge. We find that the first row of *H* perfectly distinguishes runblocks 1 and 2 from runblock 3, with values between .95 and 1.05 for the former and between − .95 and −1.05 for the latter. This ability to bridge between two different data sets supports our claim that this model is able to identify and subtract out some variation in sensitivity due to batch effects.

### Alignment of data sets

The data from a single experiment consists of a list of features along with their associated mass-to-charge ratios and retention times. Because there is some level of randomness to all of these measurements, there is some uncertainty in the identification of which feature from one experiment should be associated with a given feature in another experiment. The process of matching features across experiments is termed data alignment. For matching features within a single experiment, we utilize Rosetta Elucidator™, which is a commercial package for the processing and analysis of proteomics data. However, there were sufficient differences between the different experiments (which were run months apart, at different labs and on different machines) to make the Rosetta algorithm inadequate for the task of alignment across datasets. For data alignment across the different batches, we utilized the following construction.

Let *i* be an index over the set of all peptides measurable in our experiment. Further, define $\gamma_{i}$ to be 1 if the *i*th peptide was measured in the experiment. Let $x_{i}$ be a vector containing the “true” retention time and mass-to-charge ratio associated with the *i*th peptide. Then, if $\gamma_{i}=1$, we assume our measured values, $x_{i}^{*}$ are normally distributed around $x_{i}$ with some shift and scale along with an unknown covariance, $x_{i}^{*}∼N(\mu +\delta x_{i},\Phi^{-1})$. There is a small subset of isotope groups that have been identified in all data sets. We initialize all of our parameters to maximize the likelihood of this small subset, then use a greedy algorithm to select matches for the remainder of the isotope groups. The algorithm stops assigning matches based on the prior probability that $\gamma_{i}=1$ (we have used .5).

There is substantial information available that is not being used in this algorithm. First, it would be possible to assign prior distributions to the model parameters and iteratively fit this model. This would lead to better estimates of model parameters allong with full posterior distributions. However, because the distribution is extremely spiky, the estimation of uncertainty from this algorithm is somewhat uninteresting and uninformative. Second, there is information available in the high energy mass spectrometry trace even when that trace is insufficient to fully identify the peptide which one might include in our model as additional dimensions to $x_{i}$. Third, we are not making use of the intensity of the measured isotope groups across the samples. This allows for the possibility that there may be drastic changes in peptide concentrations between the two experiments. Even so, it is likely possible to obtain and use reasonable and informative distributions on these intensities for the purposes of alignment. However, because all of our results are based on factors, which are the aggregate expression of multiple isotope groups, a low but non-zero level of inaccurate alignments may lead to a mild increase in noise, but not a drastic change in our overall results.

### Full model specification

The list of variables we will use is as follows:

· *P* is the number of isotope groups measured

· *N* is the number of samples in the study

· *X* is a *P* × *N* dimensional array of intensity values with elements $x_{i,j}$

· *ϵ* is a *P* × *N* matrix of normally distributed noise (residuals). We assume that each isotope group, *i*, has its own noise variance, $\tau_{i}^{-1}$

· *μ* is a *P*-dimensional vector representing mean intensity for each isotope group. The elements of this vector are *μ*_*i*_.

· *B* is a *P* × *D* matrix of loadings and *H* is a *N* × *D* matrix of factors. These are intended to describe patterns of expression that span most or all isotope groups (as reflected by the priors). The elements of extitB are $b_{i,d}$ and the elements of *H* are $h_{n,d}$.

· *A* is a *P* × *K* matrix of loadings and Λ is a *N* × *K* matrix of factors. *K* is the number of metaproteins. These factors are distinguished from the previous ones by their prior structure. Specifically, *A* will contain only *P* non-zero elements, and the non-zero elements for any particular factor should consist of a large number of isotope groups from the same protein. This contrasts with *B* which contains no non-zero elements and describes expression patterns across the entire set of peptides. The elements of *A* and Λ are $a_{i,k}$ and $\lambda_{n,k}$ respectively.

· *z* is a *P*-dimensional vector (with elements *z*_*i*_) containing, for each isotope group, the index of the metaprotein to which that isotope group has been assigned.

· $\mu_{0}$, $\phi_{0}$, $v_{0}$, $\nu_{0}$, $\delta_{0}$, $a_{0}$ and $a_{k}$ are constants set at the time of model fitting.

The full hierarchical model specification is as follows.

(7) $$\begin{array}{l} X=\mu 1_{N}+BH^{\prime }+A\Lambda^{\prime }+\epsilon \\ \mu_{i}∼N(\mu_{0},\phi_{0})\\ b_{i,d}∼N(0,v_{0})\\ h_{n,d},\lambda_{n,k}∼N(0,1)\\ \epsilon_{i,n}∼N(0,\tau_{i}^{-1})\\ \tau_{i}∼Gamma(\nu_{0},\delta_{0})\\ a_{i,d}|z_{i}\neq d=0\\ a_{i,d}|z_{i}=d∼N(0,v_{0})\\ z_{i}Multinomial(1,q_{i})\\ q_{i}Dir(\alpha_{0},\cdots ,\alpha_{0},\alpha_{k},\alpha_{0},\cdots \alpha_{0}) \end{array}$$

All of the constants are set in order to minimize the influence of the prior distribution. Specifically, we use $\mu_{0}=8$ and $\phi_{0}=100$ corresponding to a 95% confidence interval on the log-transformed intensities between −12 and 28 (significantly overdispersed relative to the empirical distribution). Similarly, we set $v_{0}=100$ which allows potential fold changes $>10^{8}$. We set $\nu_{0}$ and $\delta_{0}$ both to .001 corresponding to a mean estimated residual of 1, but with a variance of 1000. We note that estimation of variance can be difficult, however, we have >100 samples in our study. For smaller studies, one might use more informative priors for variance. Both $a_{0}$ and $a_{k}$ are pseudocounts for our Dirichlet distribution. We use $a_{0}=.01$ and $a_{k}=500$. We note that this last parameter is the only one in the model that is potentially strongly informative, so we have tried values from 1 to 1000. We considered the percentage of peptides that are members of their dominant metaprotein. We find that, in our study population the affect of varying this parameter is to move that percentage between 43% and 53%. The relatively minor effect of this large change in pseudocounts is probably due, again, to our large sample size. This leads to very high data likelihoods for membership in particular metaproteins for almost all isotope groups. This parameter will have a larger effect in smaller studies.

## Authors’ contributions

JEL - Development of the statistical approach, all modeling and statistical analysis, the majority of the writing. JWT - Generation of the data through the utilization of the mass spectrometers. Writing of the sections of the paper describing the mass spectrometry. Key participant in discussions that led to the development of the statistical methods. LGD - Generation of the data through the utilization of the mass spectrometers. JMcH and JMcC - Design of the experiment. HT, AT, KP - Collection of biological samples and curation of the medical records in association with the HCV55 and HCV41 samples. MAM - Experimental design and management of the proteomics core. Key participant in discussions that led to the development of the statistical methods. NS, RH, FD and PG - Generation of repeat open platform measurements in an independent lab using different mass spectrometry technology. KS - Collection of biological samples and curation of the medical records in association with the Peds C samples. All authors read and approved the final manuscript.

## Supplementary Material

Additional file 1 **Protein level grouping.** Heatmaps for all identified isotope groups. The rows are sorted according to metaprotein membership and the columns are sorted so that the first principal component is increasing. Each isotope group is associated with a single metaprotein. Those metaproteins are labeled on the y-axis. Click here for file

Additional file 2 **Protein level, sorted by expression.** Heatmaps for all identified isotope groups. The rows are sorted according from highest to lowest expression level and the columns are sorted so that the first principal component is increasing. Each isotope group is associated with a single metaprotein. Those metaproteins are labeled on the y-axis. Click here for file

Additional file 3 **Analysis of spike-in data.** Estimates of the protein levels of spiked in samples from the Super Hirn experiment. Also included are the results of one of the label substitution experiments. Click here for file

Additional file 4 **Expression from SRM.** Raw expression levels from the label free SRM proteomics assay for a subset of the original HCV 55 cohort. Click here for file

Additional file 5 **Shotgun proteomics data and Matlab code for analysis.** Raw expression levels from the various shotgun proteomics experiments and Matlab code for performing the analysis described in this paper. Click here for file
