# Supplementary figures and images for: Metaprotein expression modeling for label-free quantitative proteomics
Source: BMC Bioinformatics. 2012 May 4;13:74. doi: 10.1186/1471-2105-13-74 (PMC3436780; doi:10.1186/1471-2105-13-74)

# Supplemental Information 1

E-mail:

---

\*To whom correspondence should be addressed

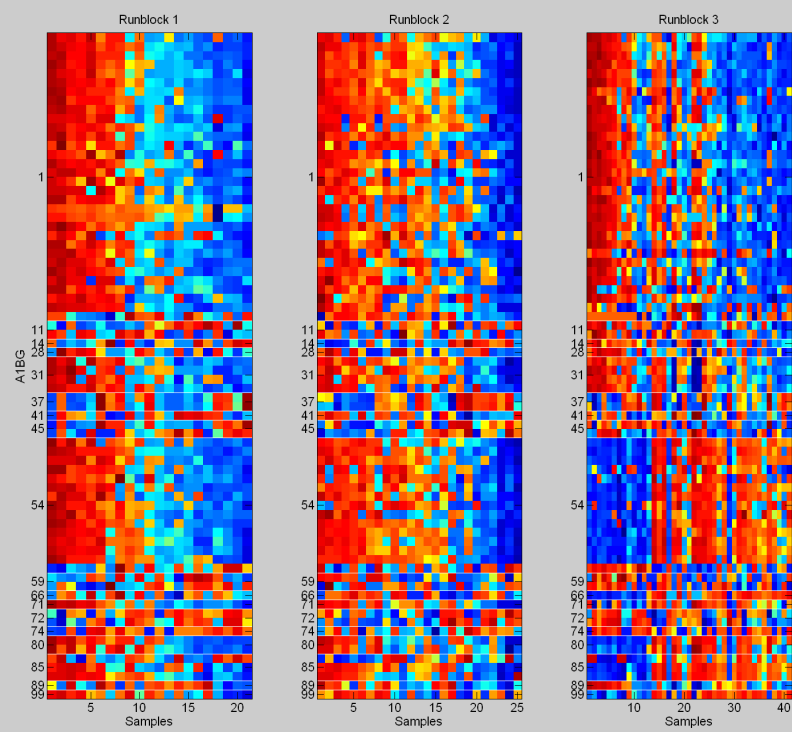

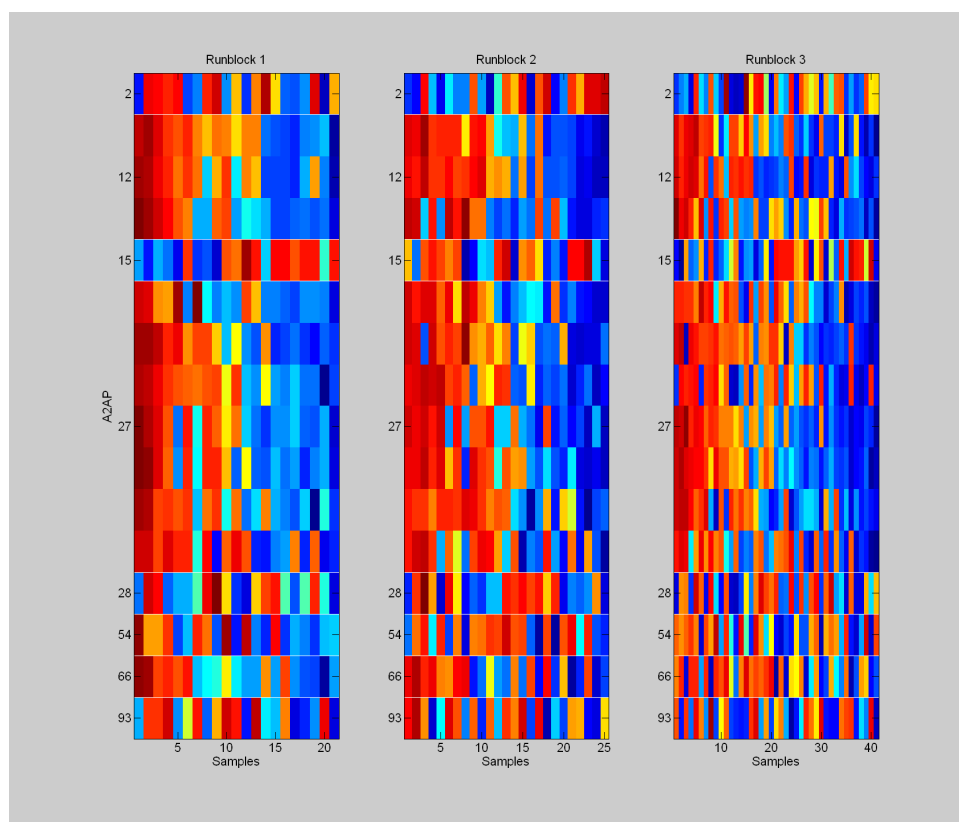

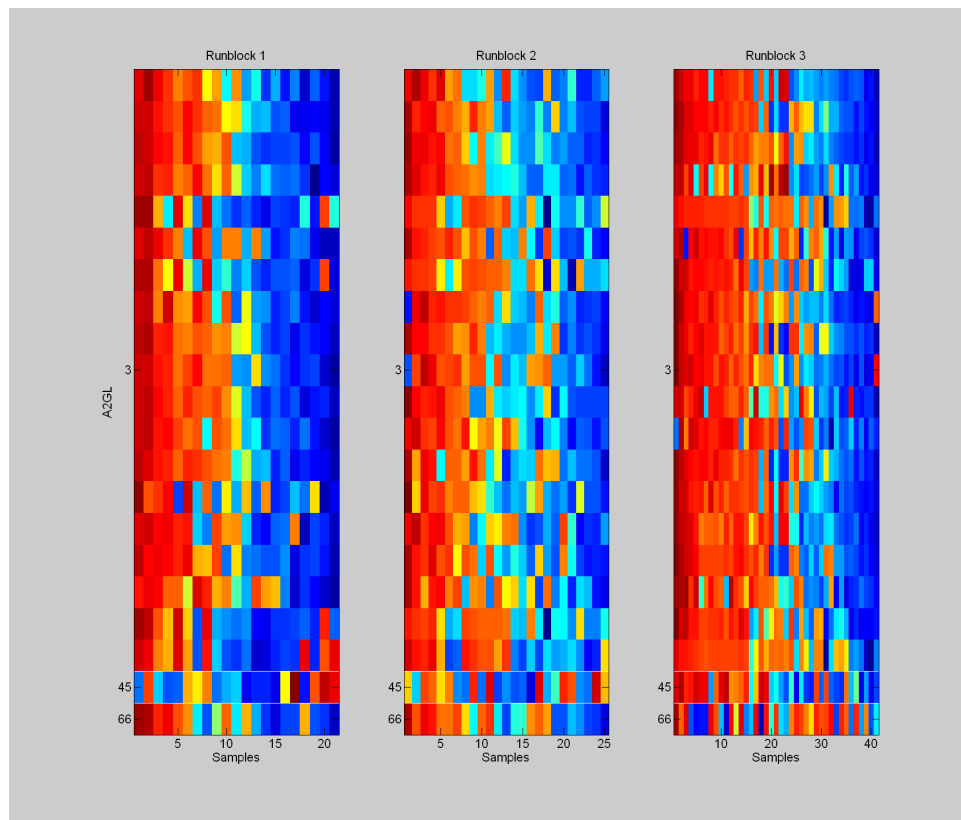

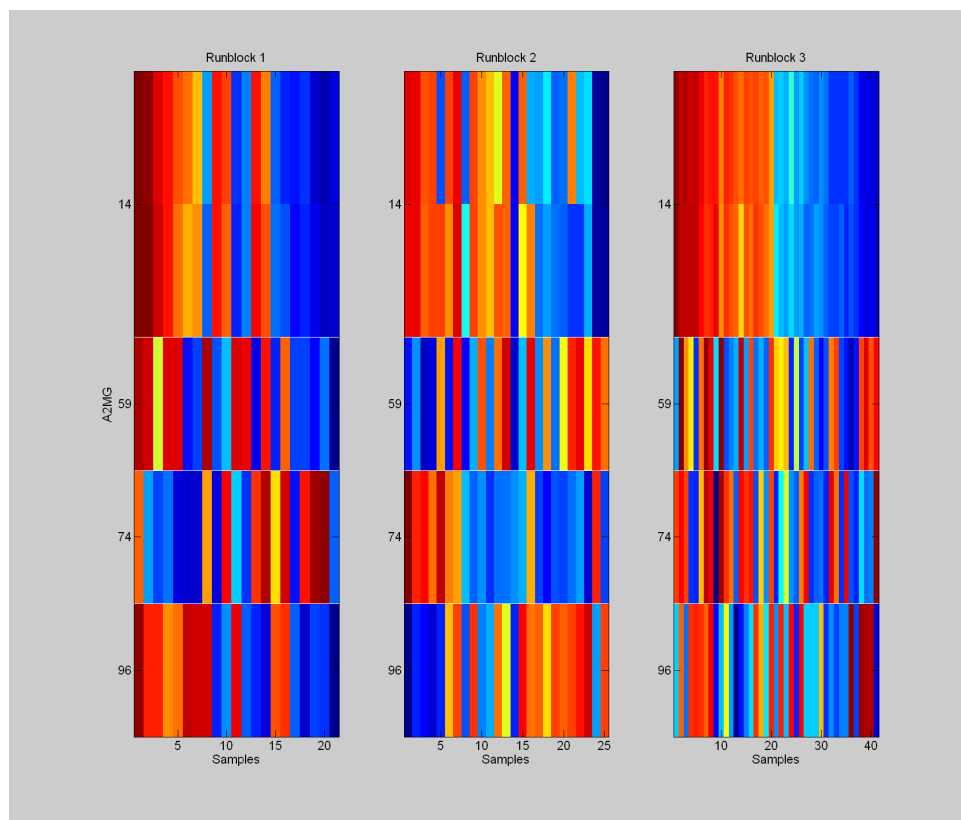

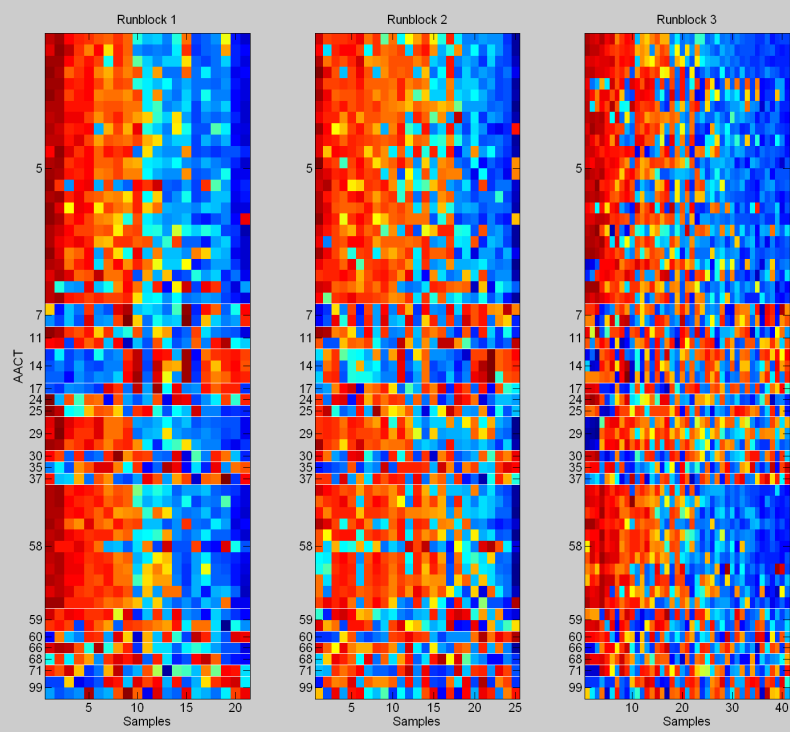

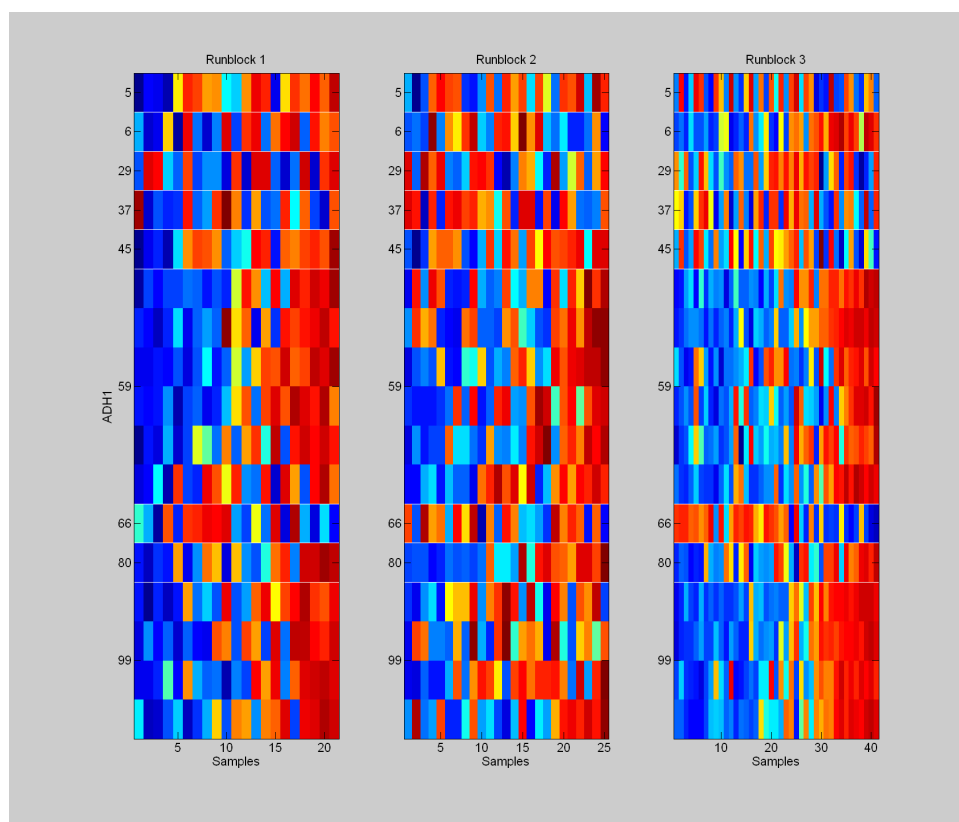

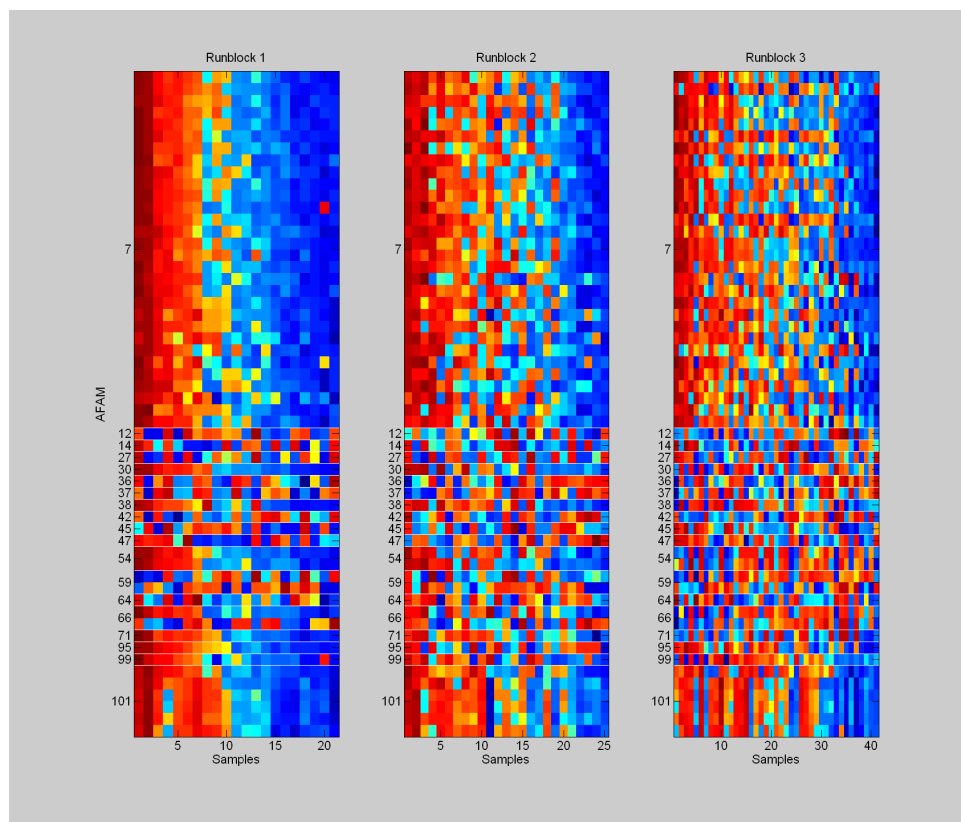

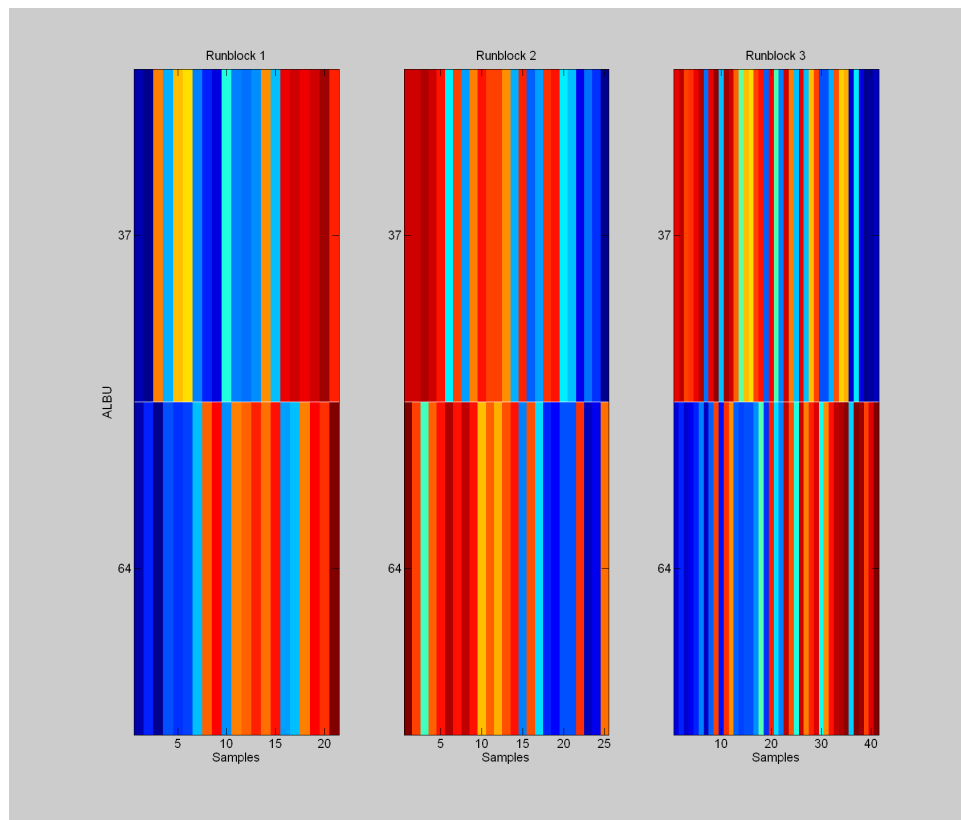

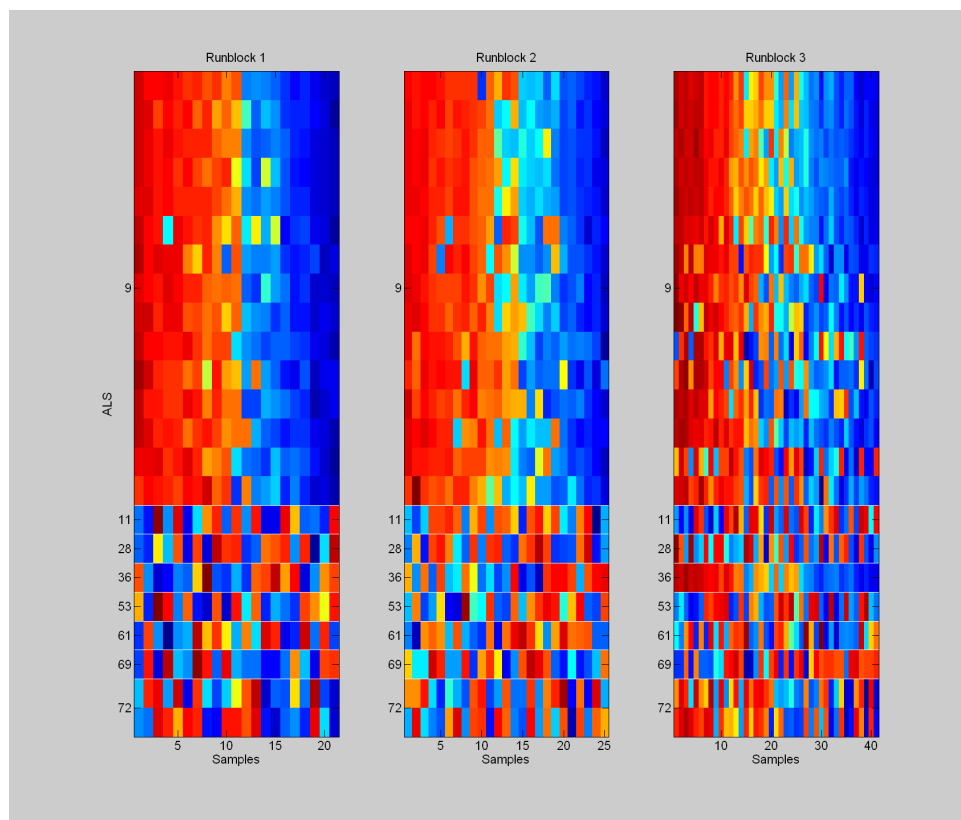

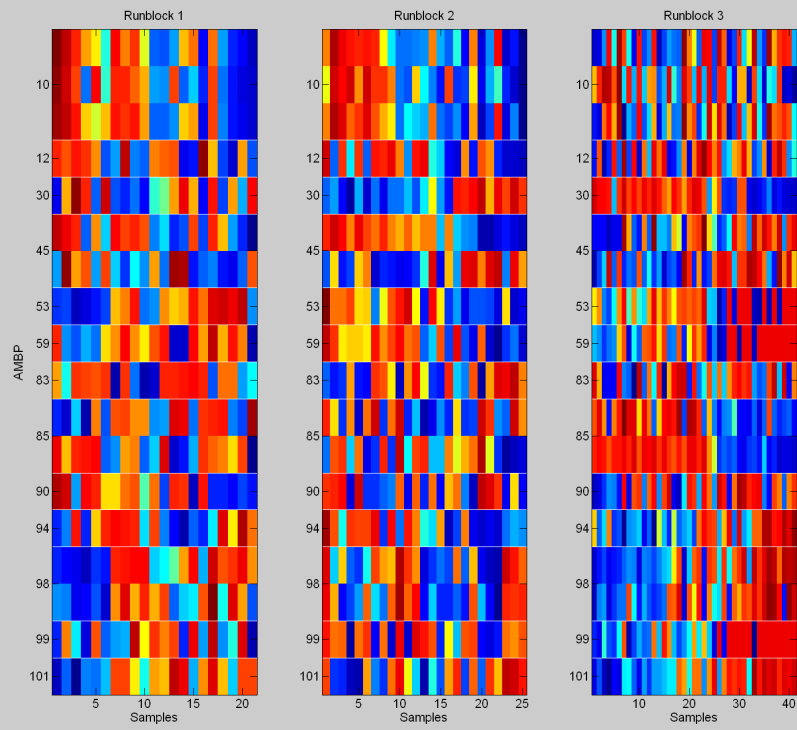

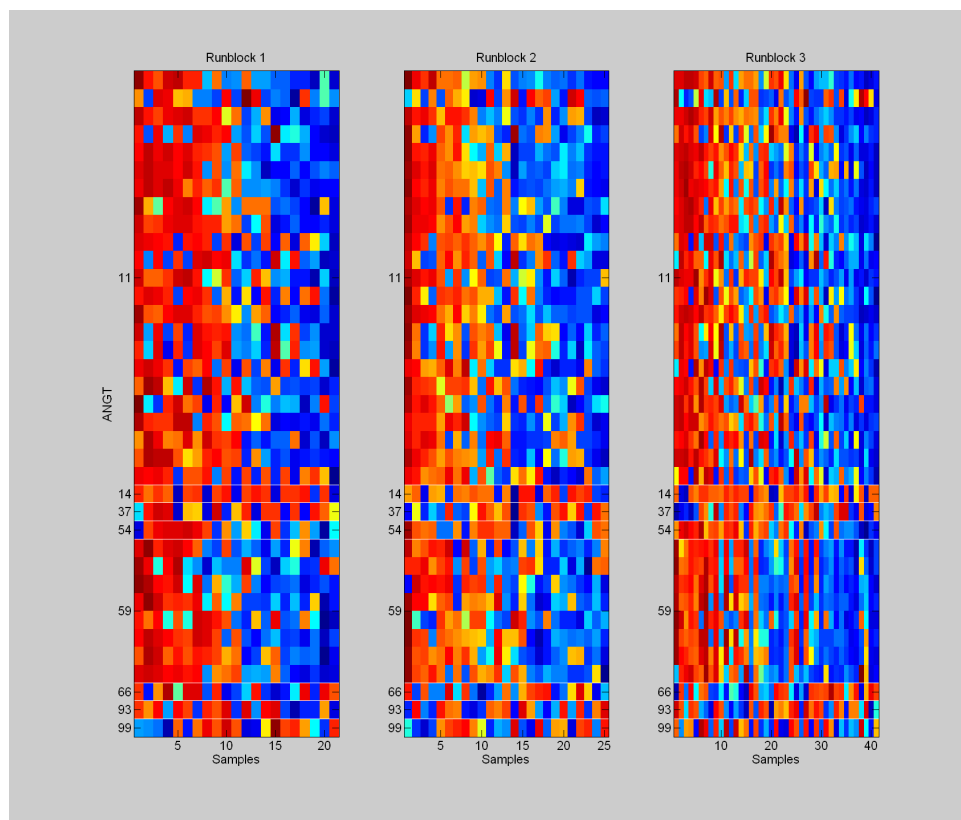

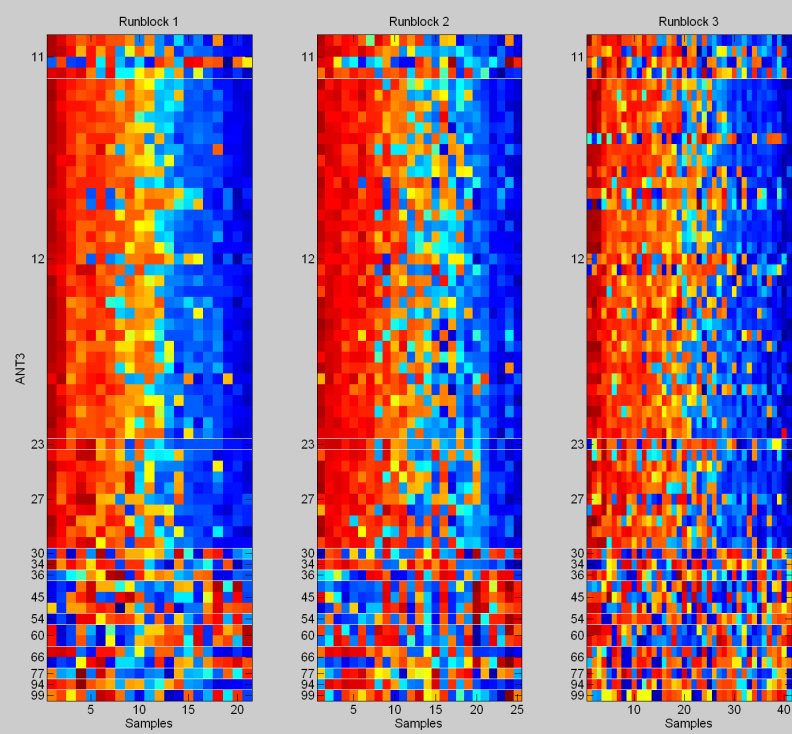

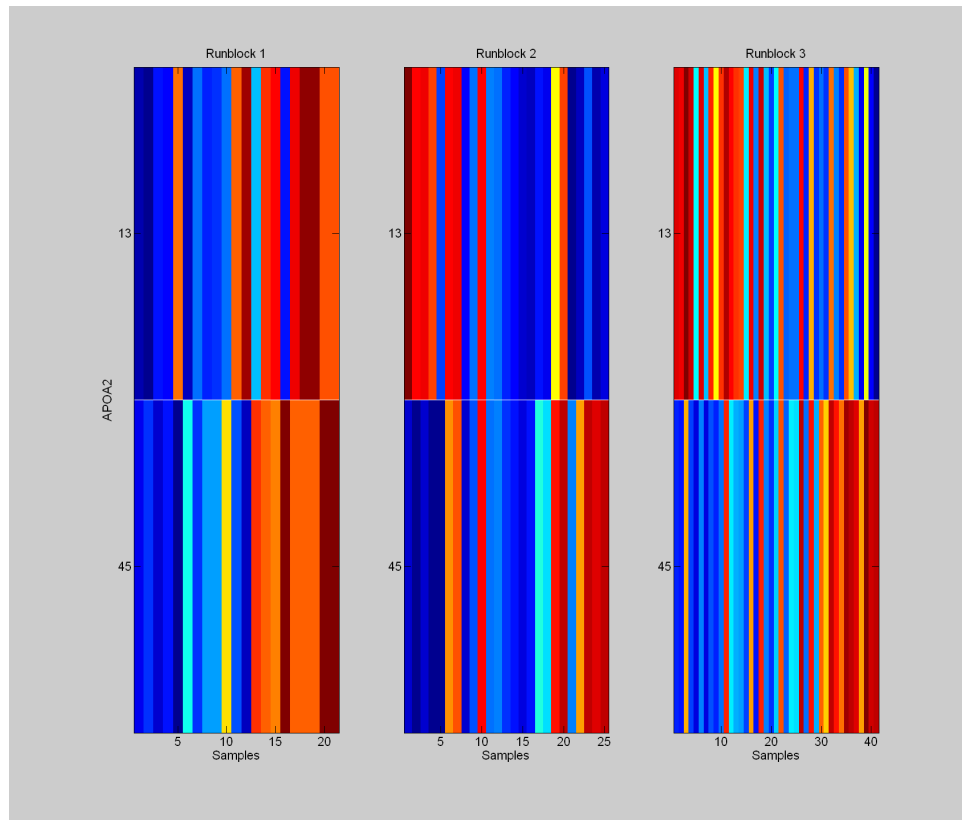

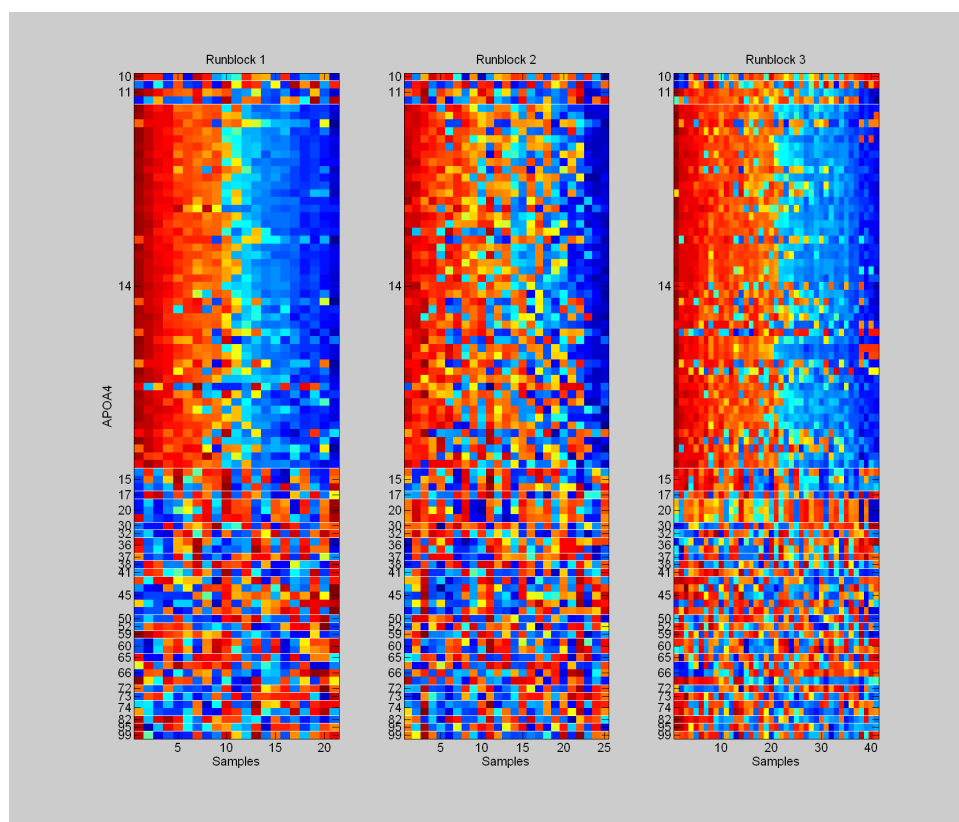

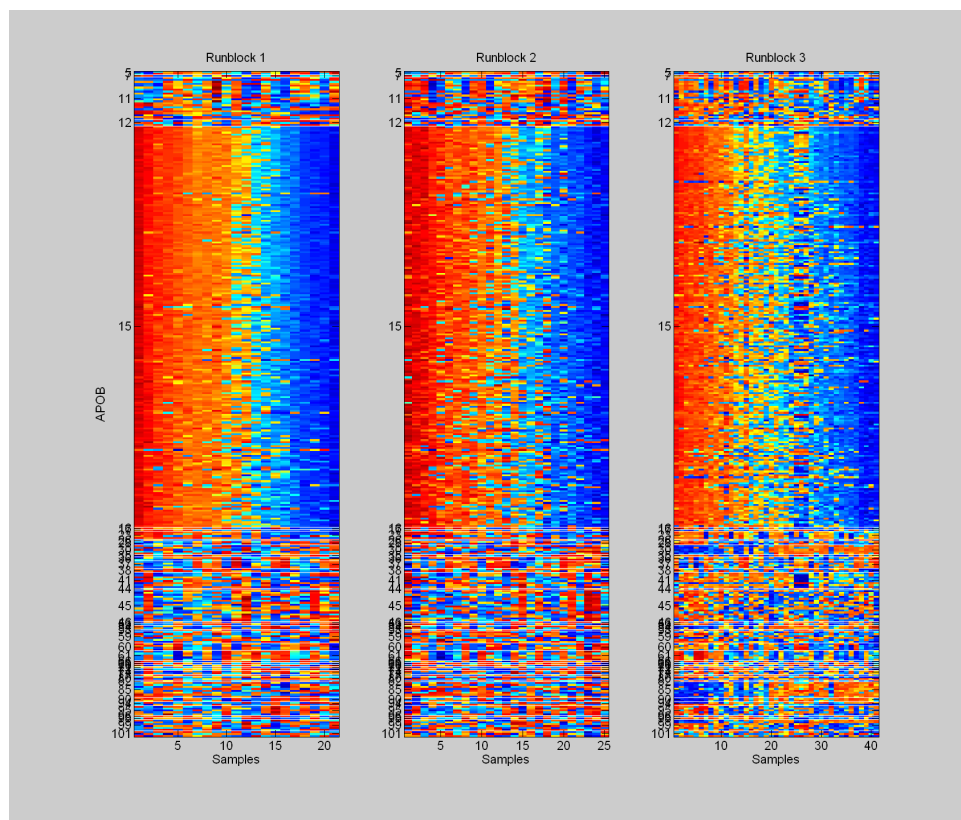

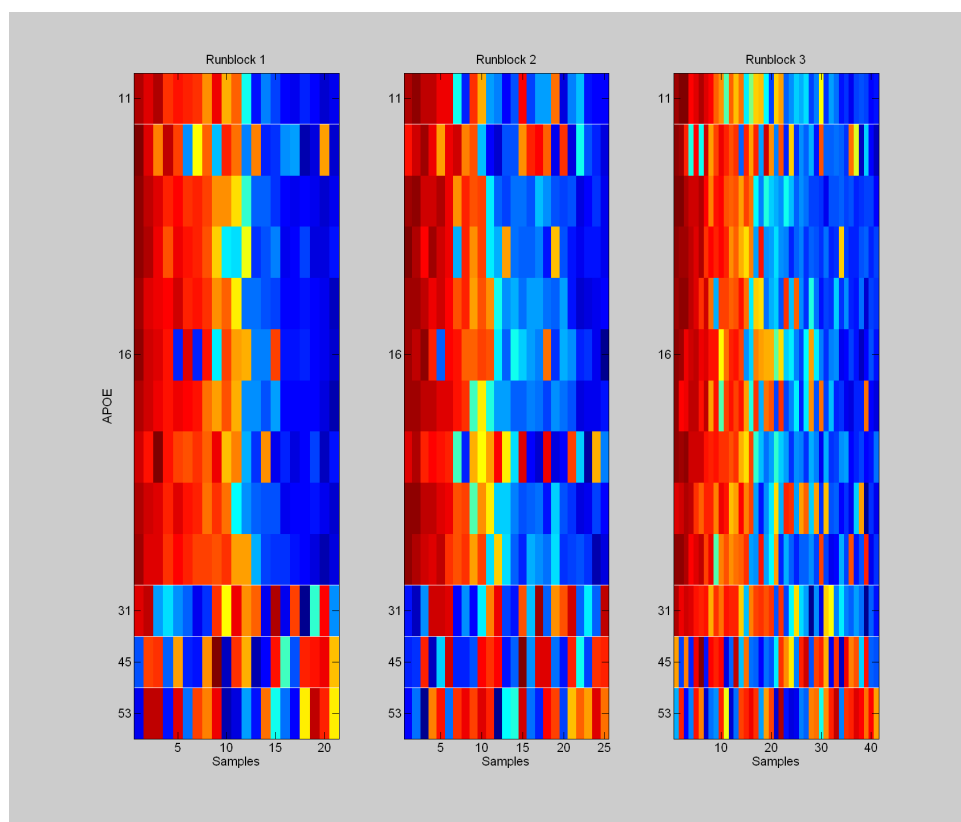

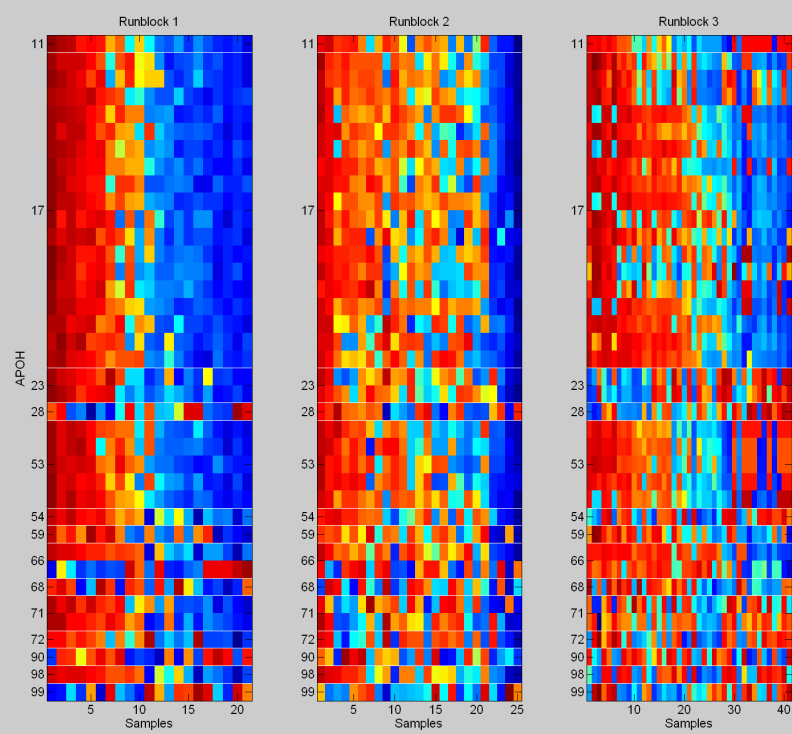

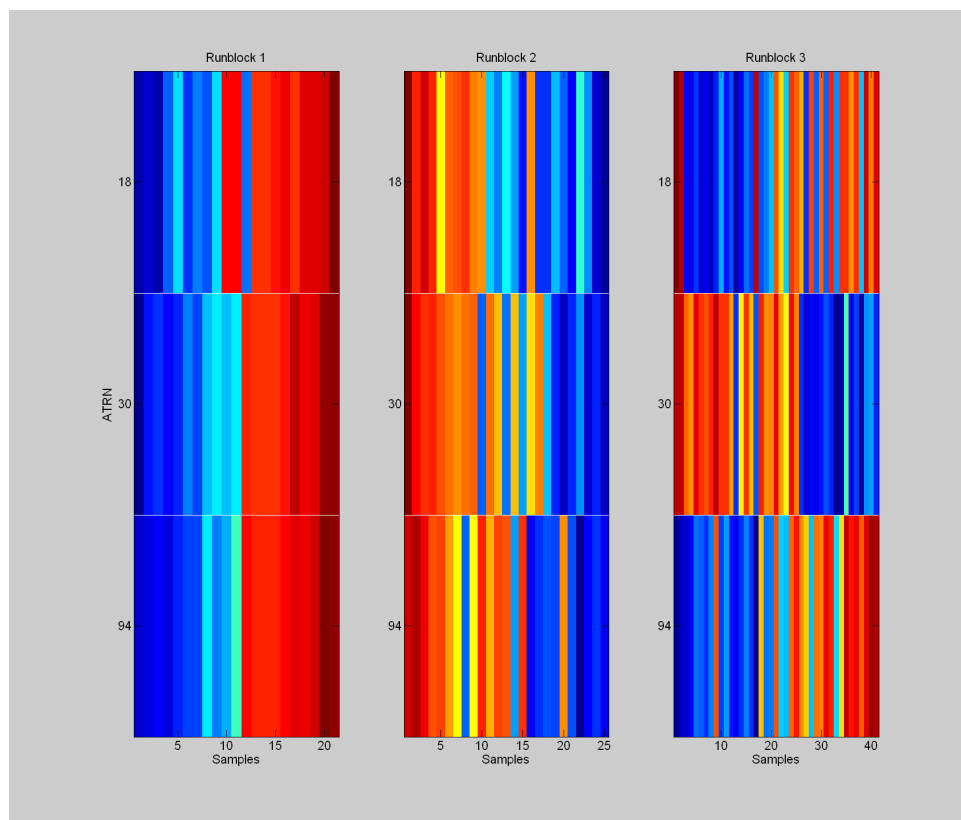

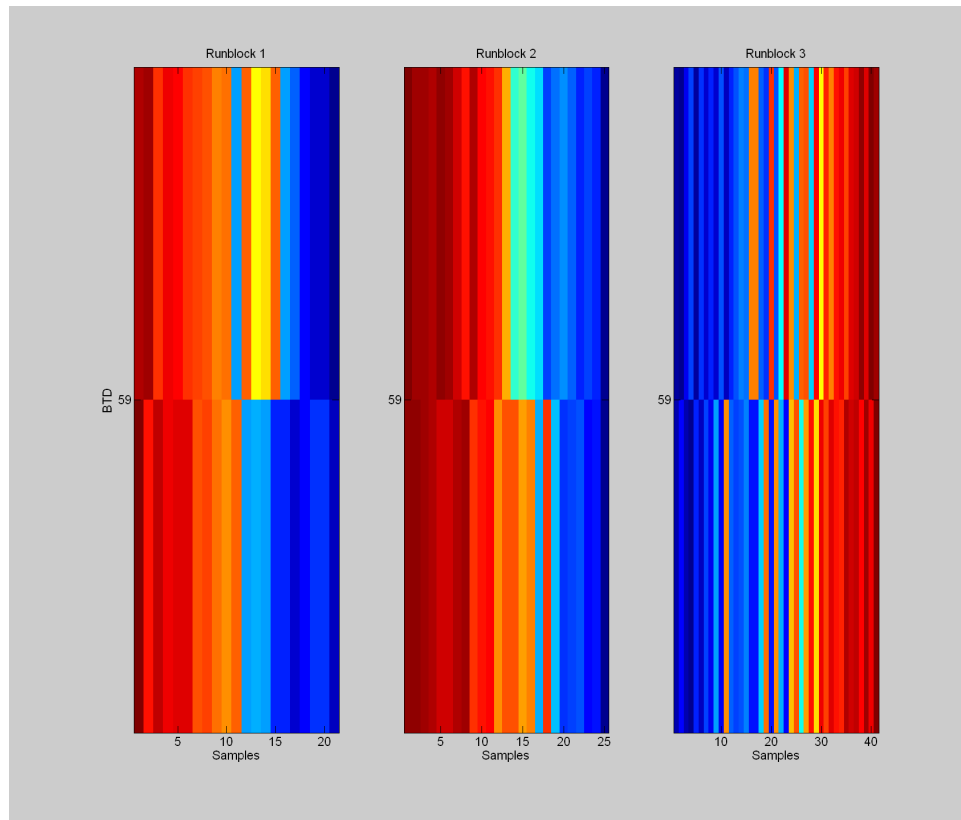

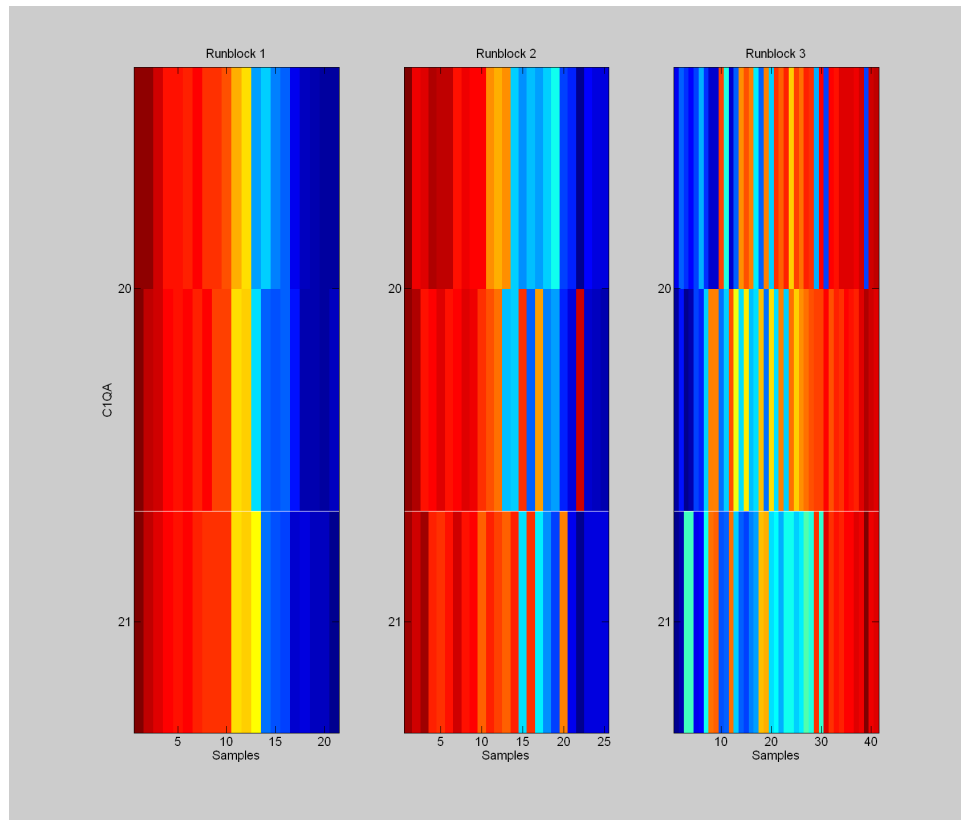

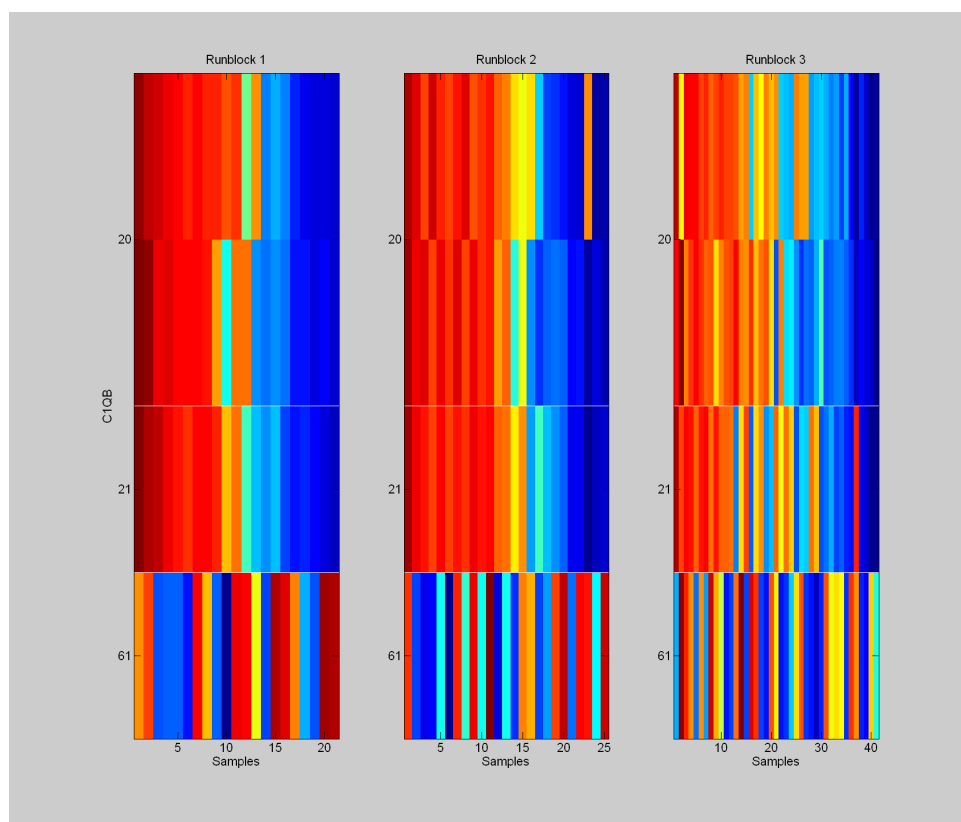

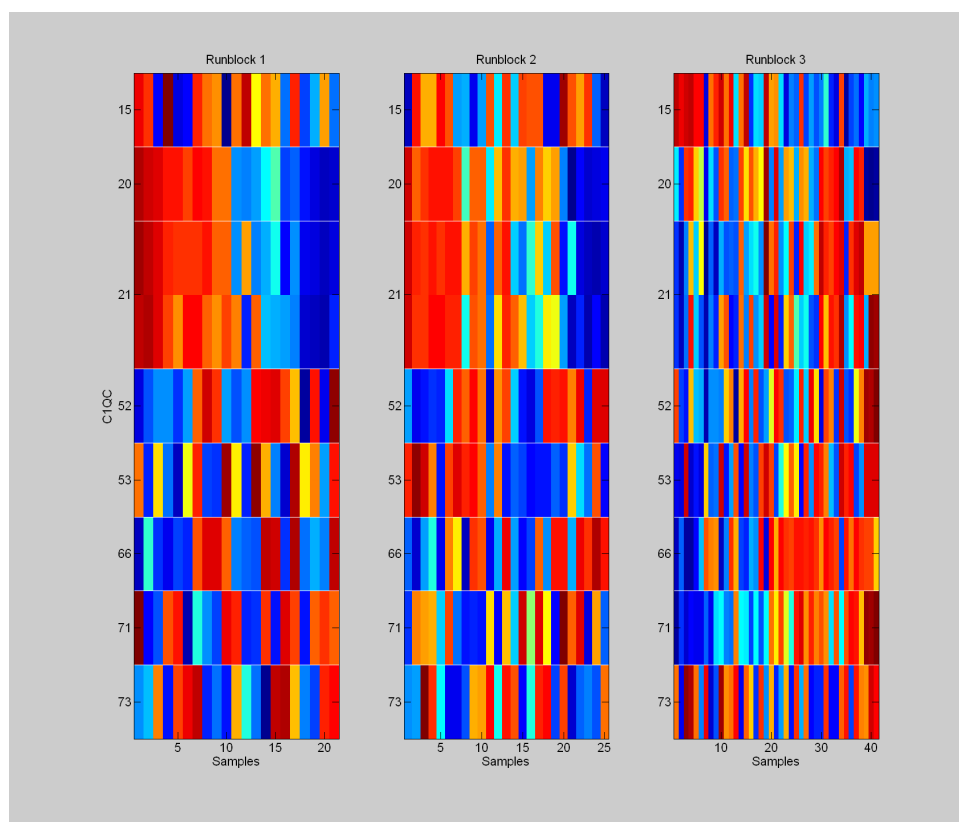

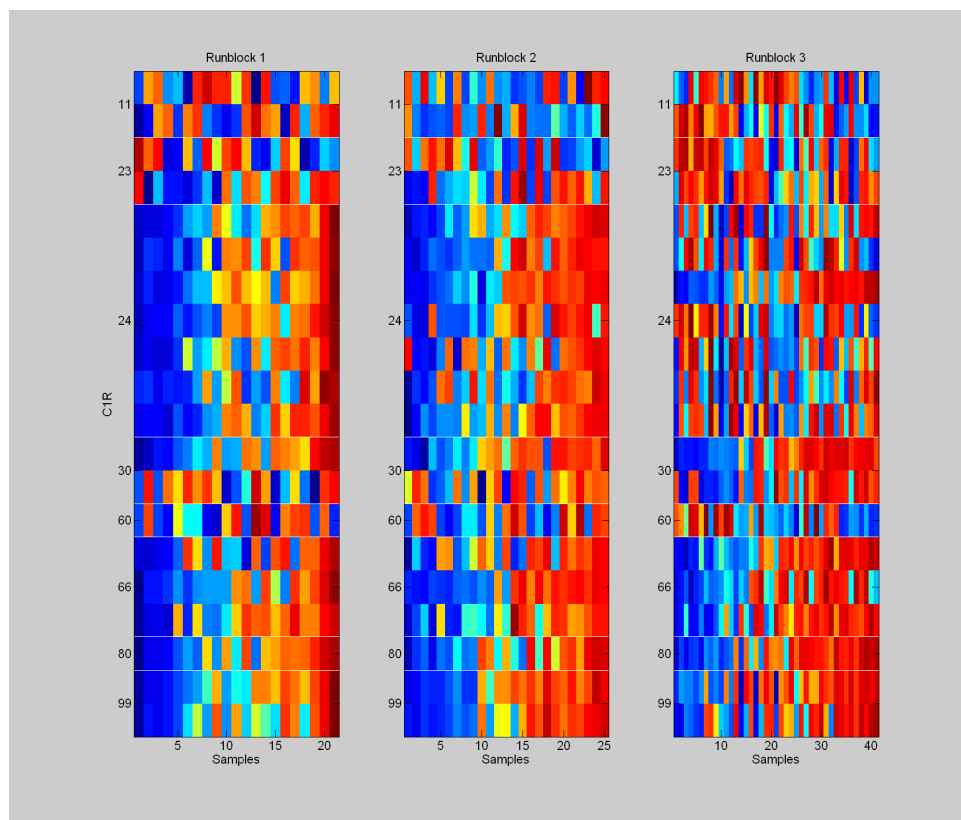

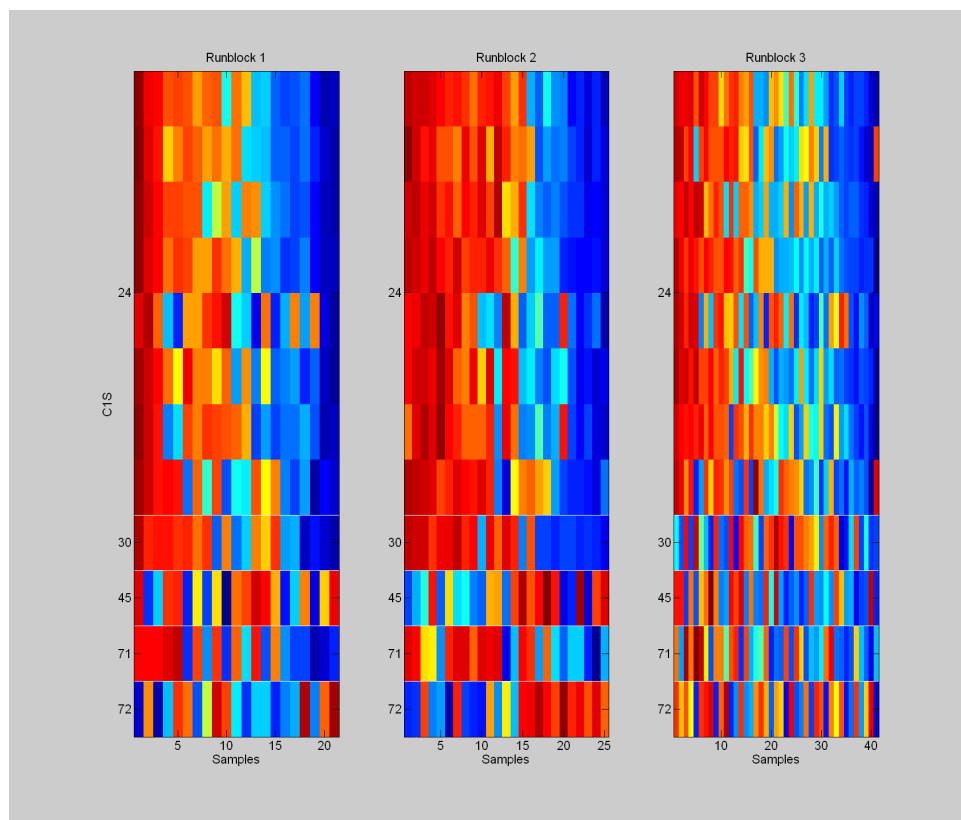

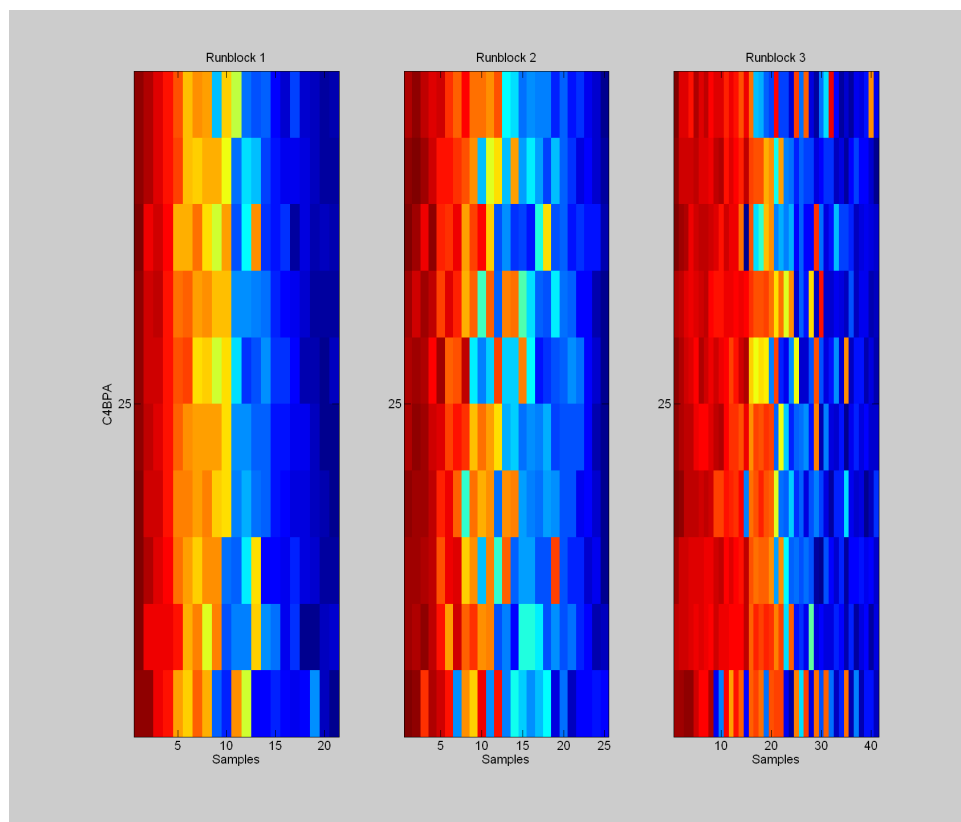

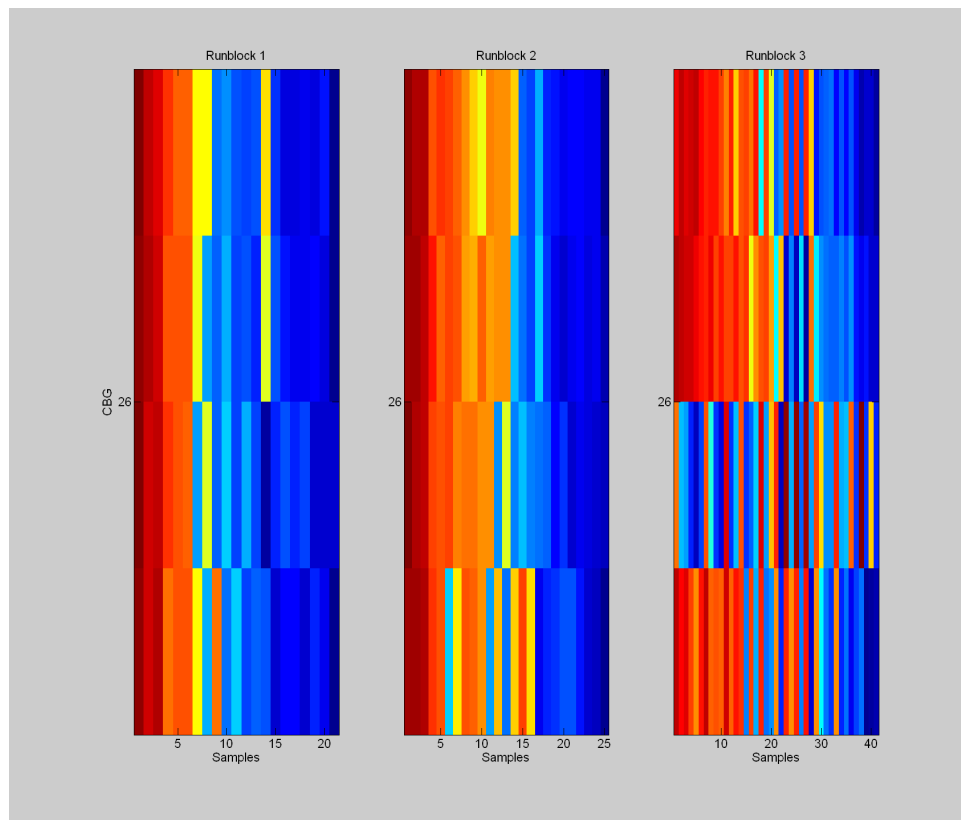

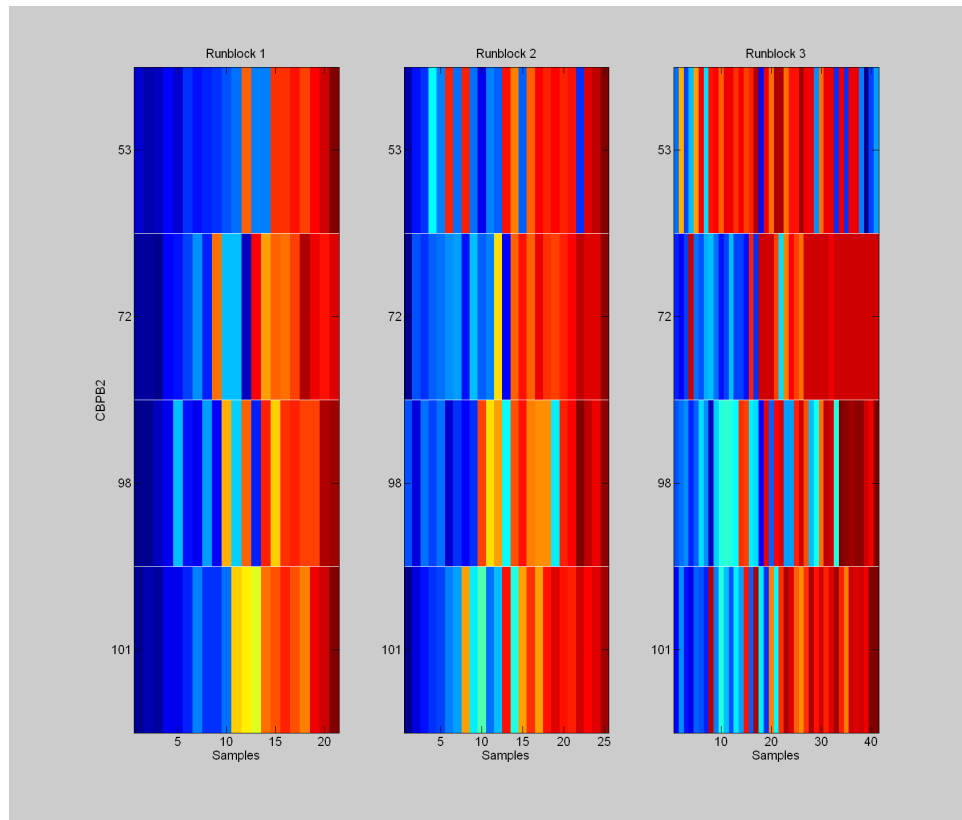

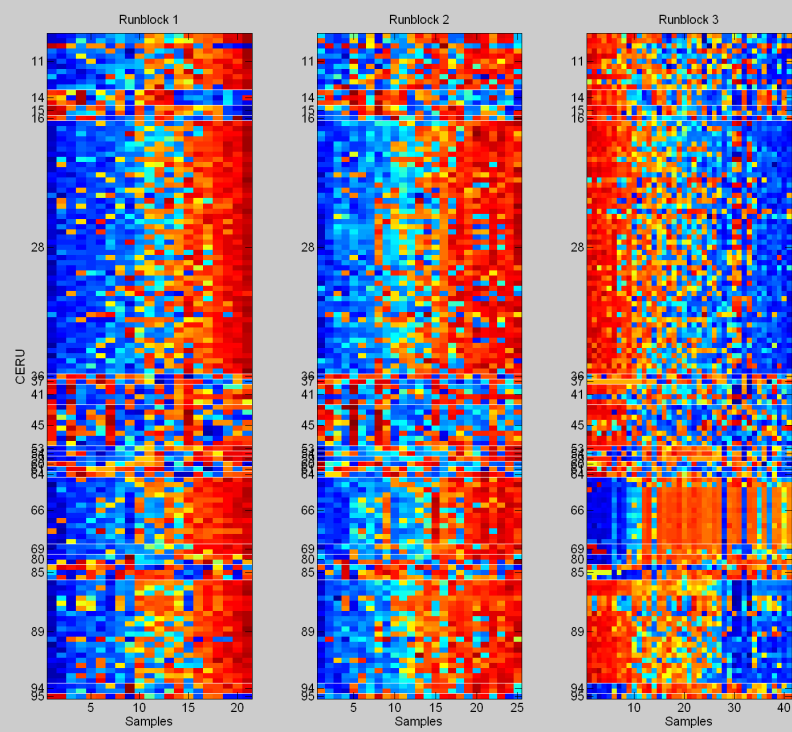

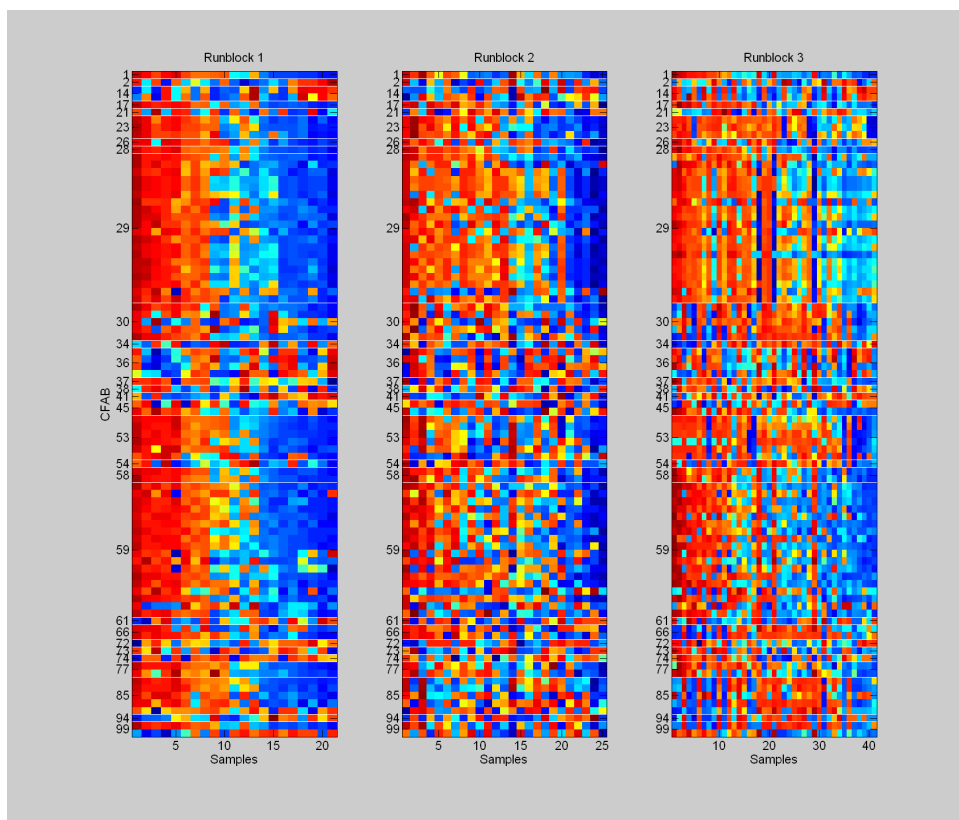

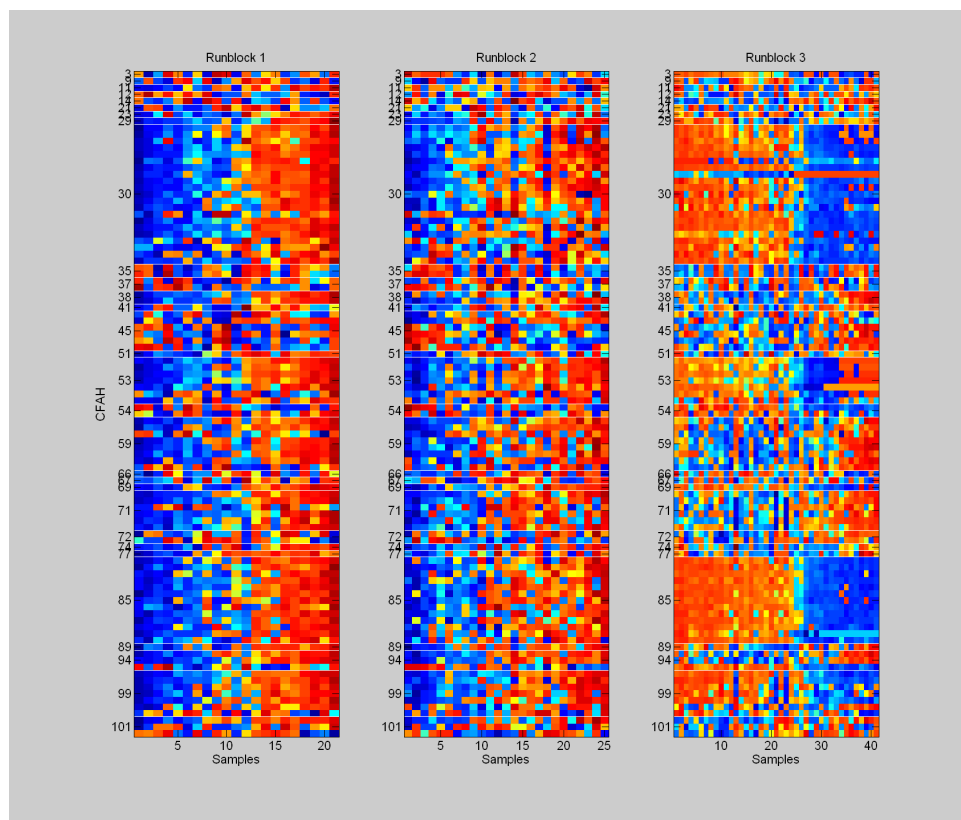

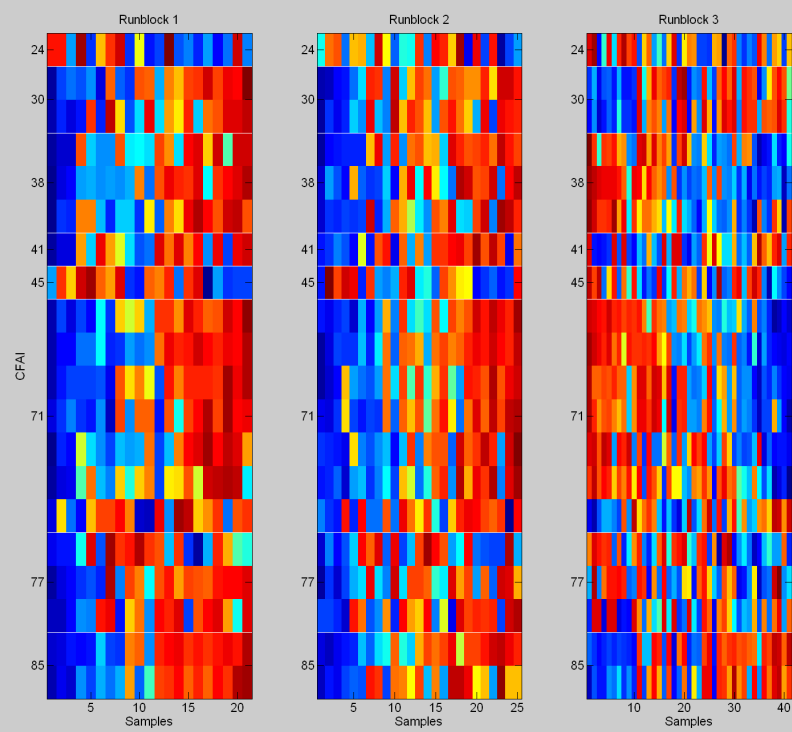

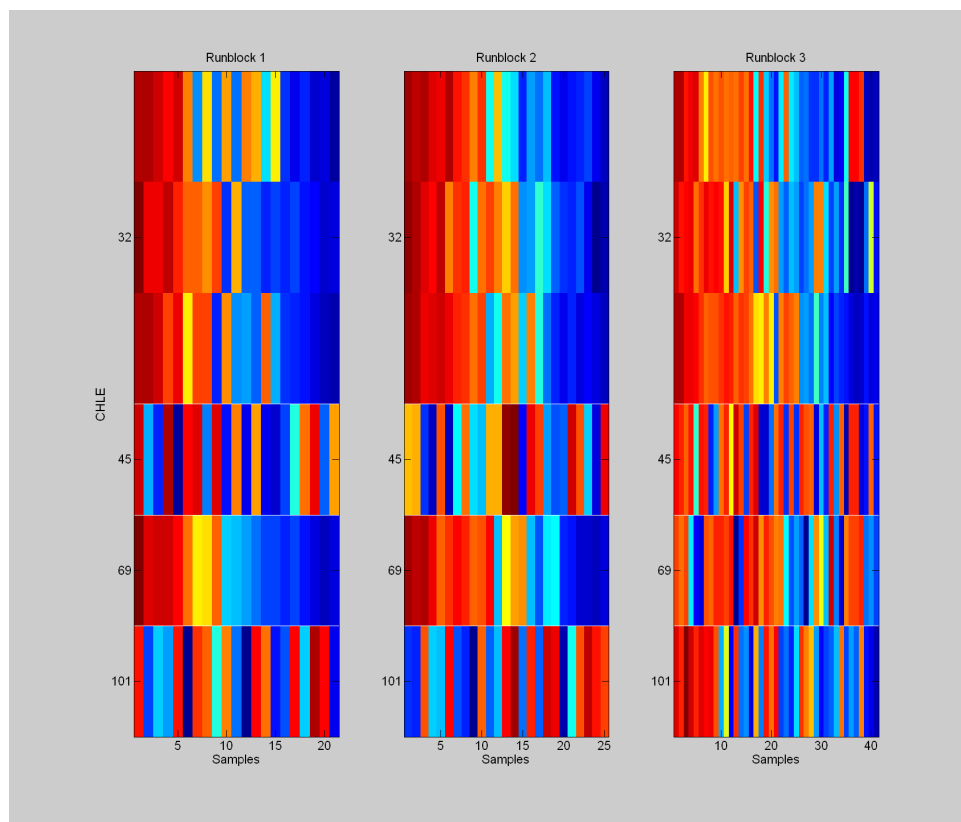

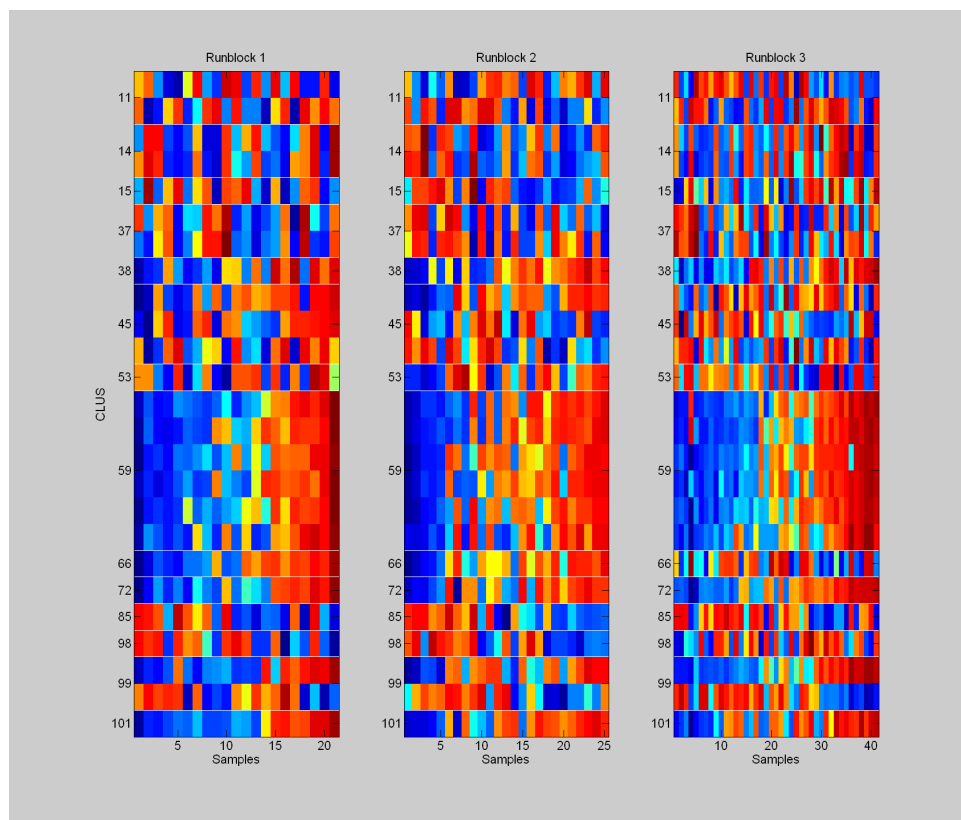

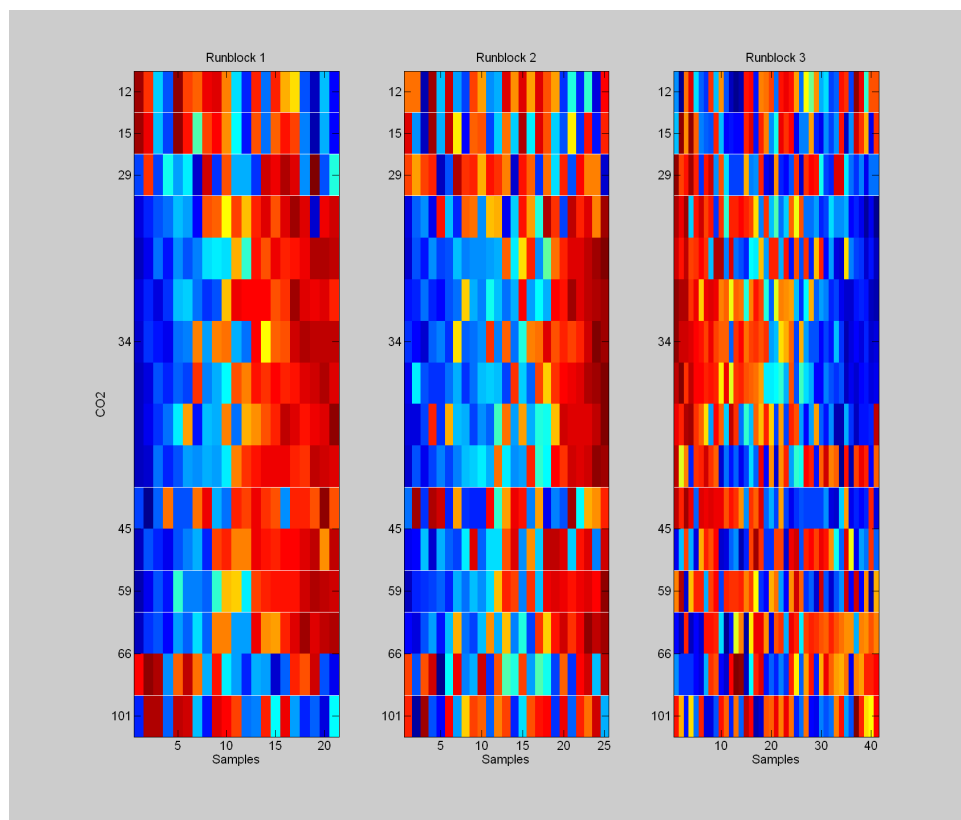

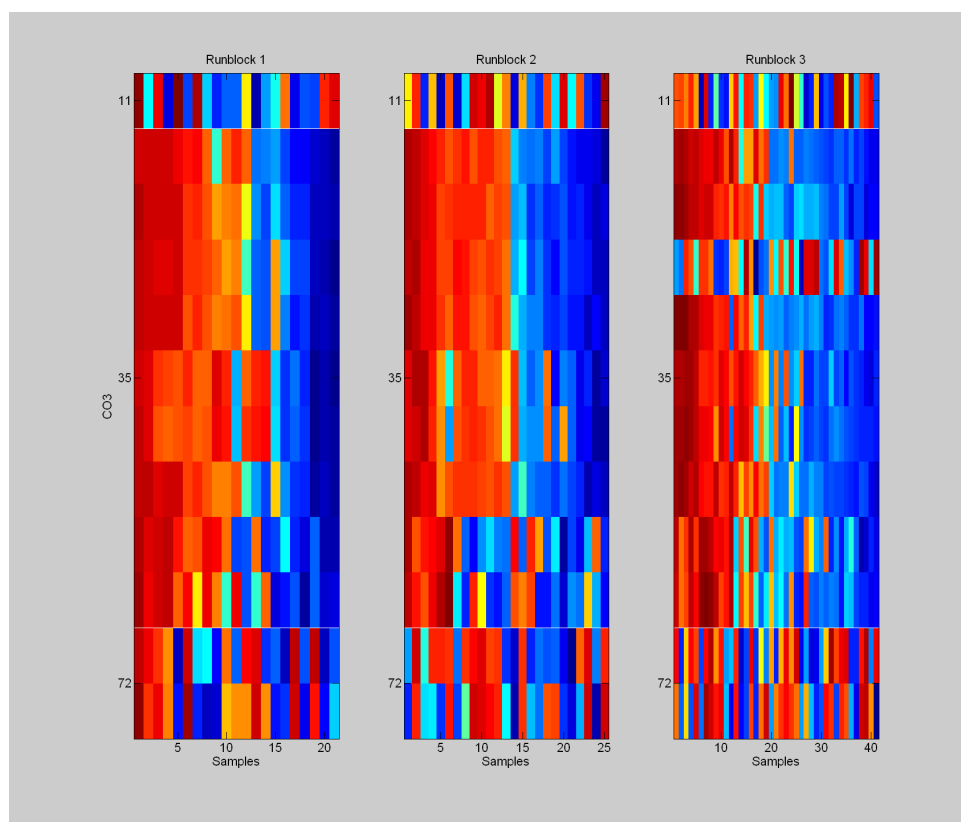

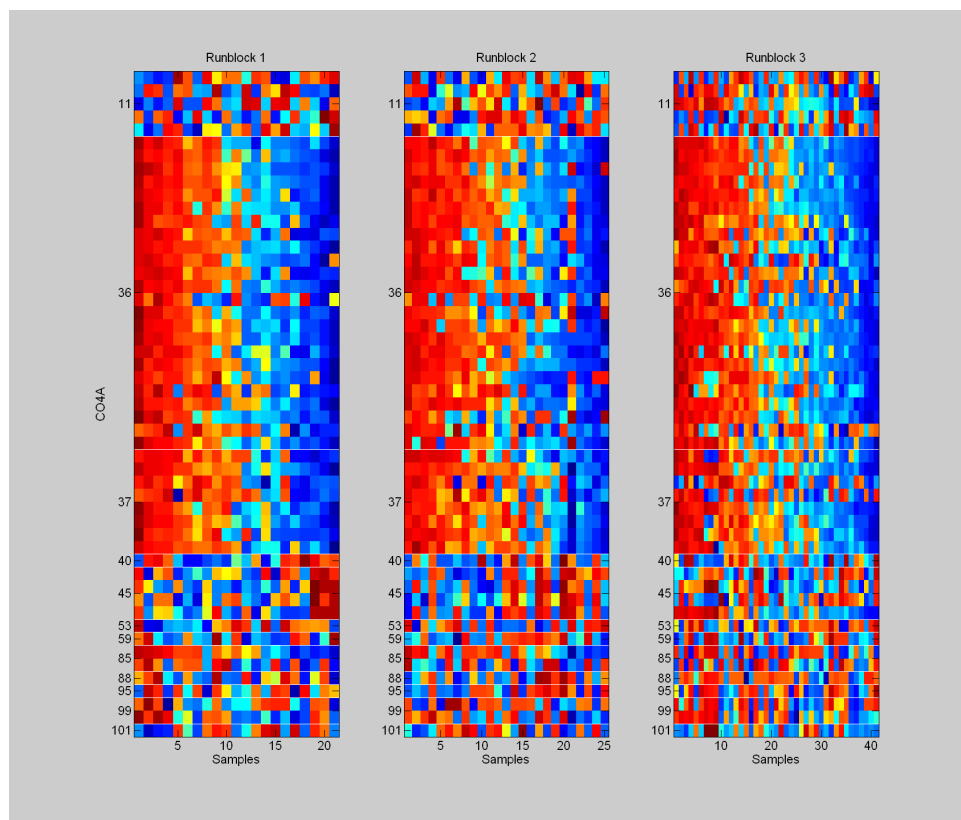

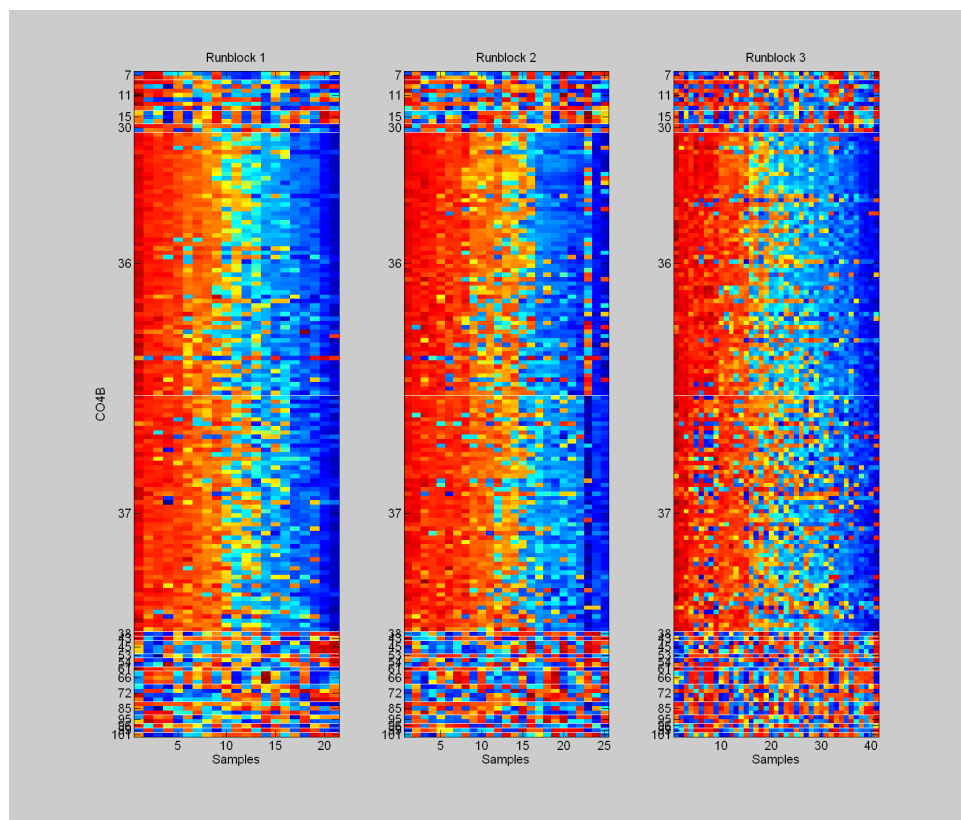

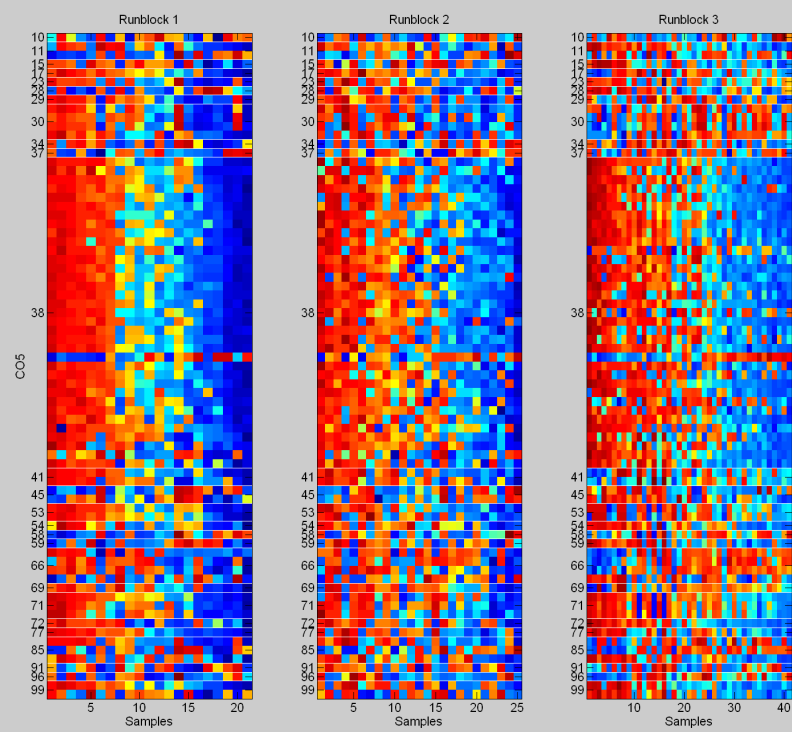

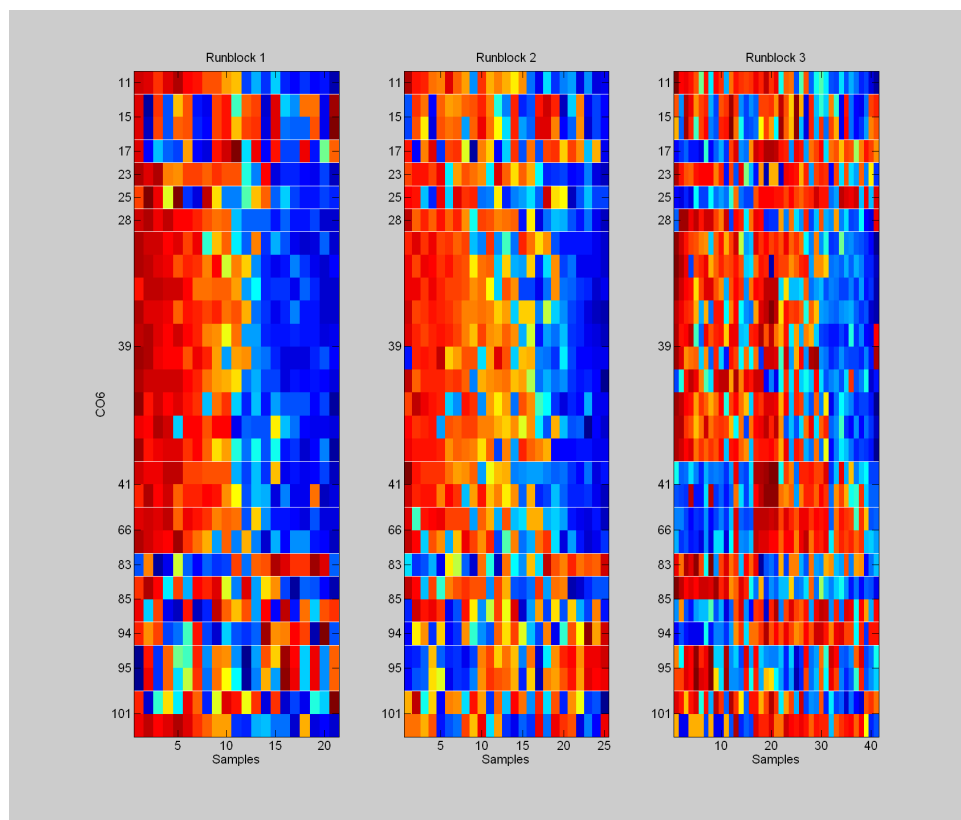

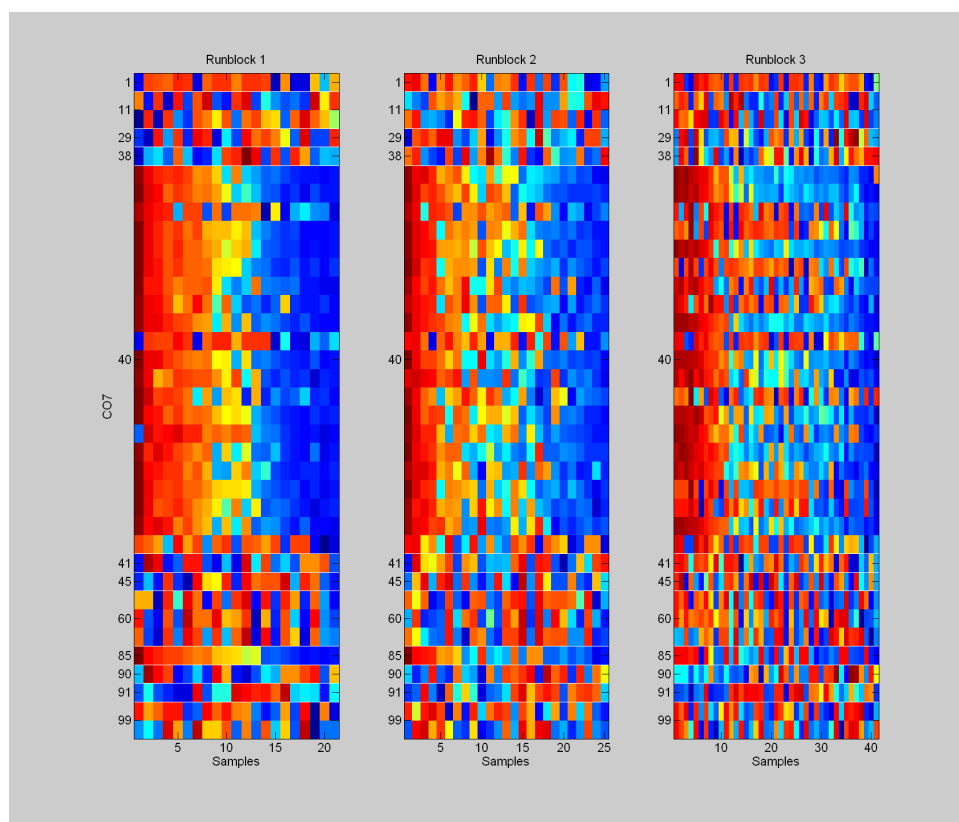

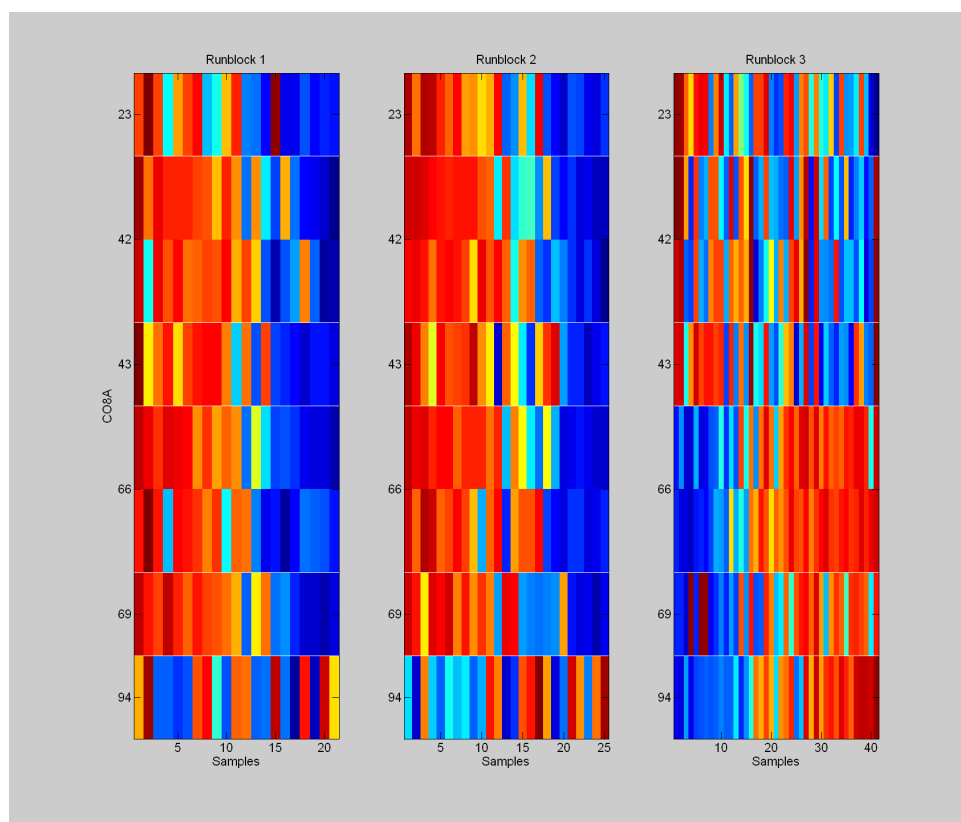

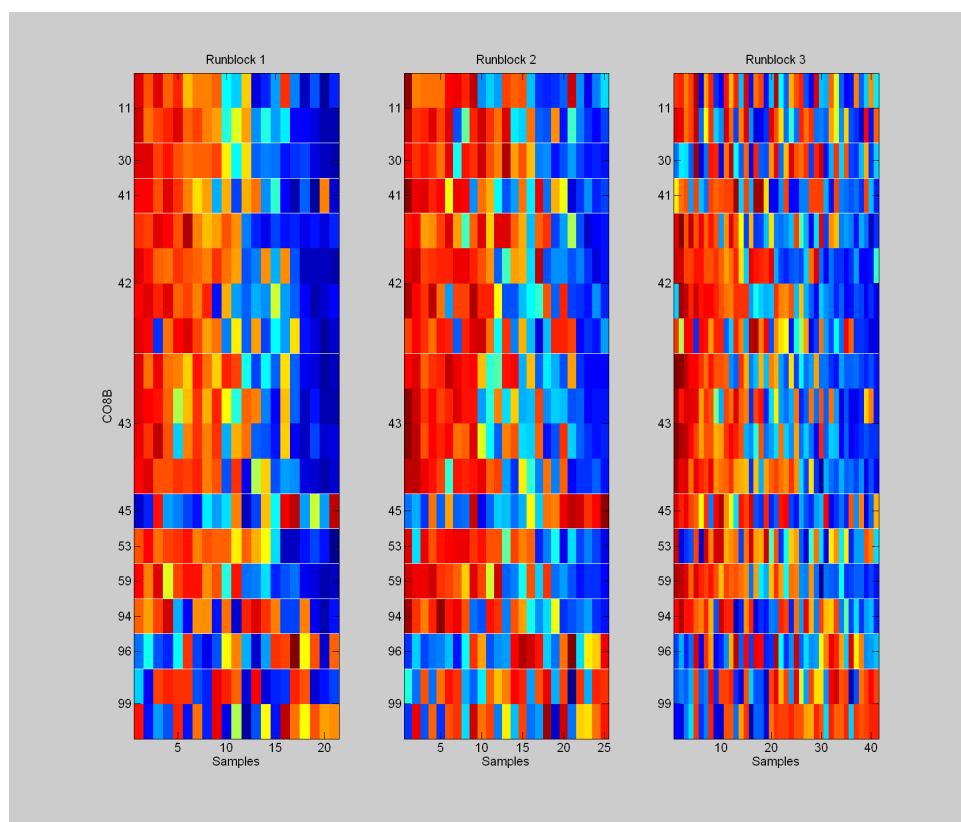

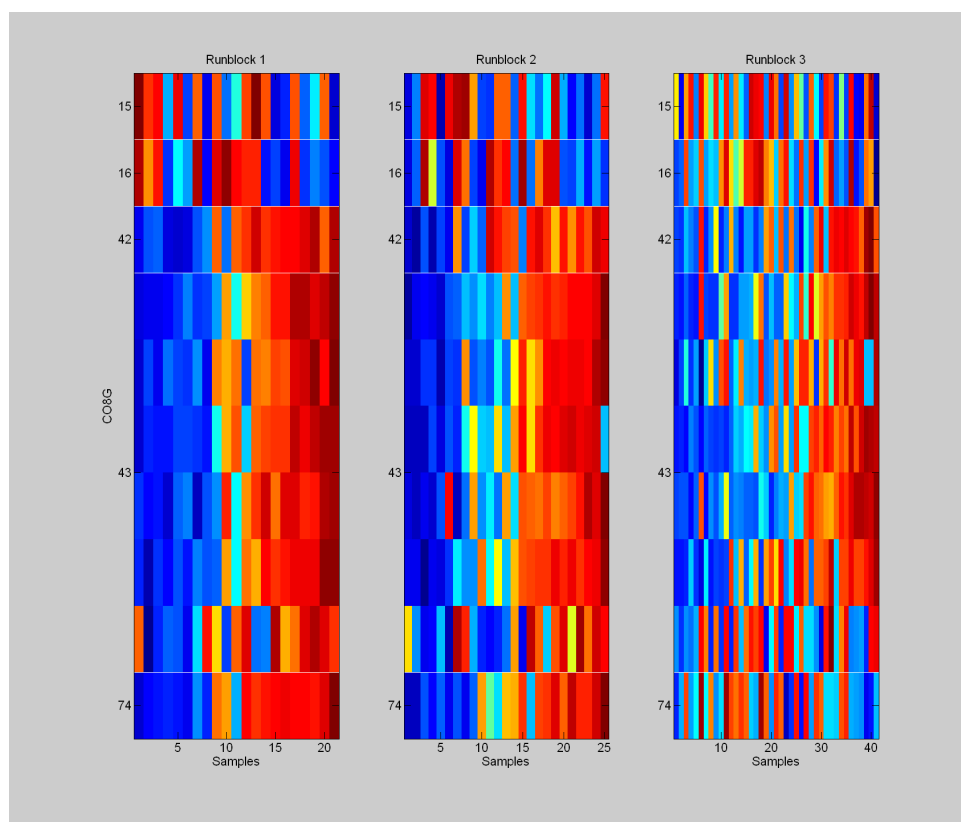

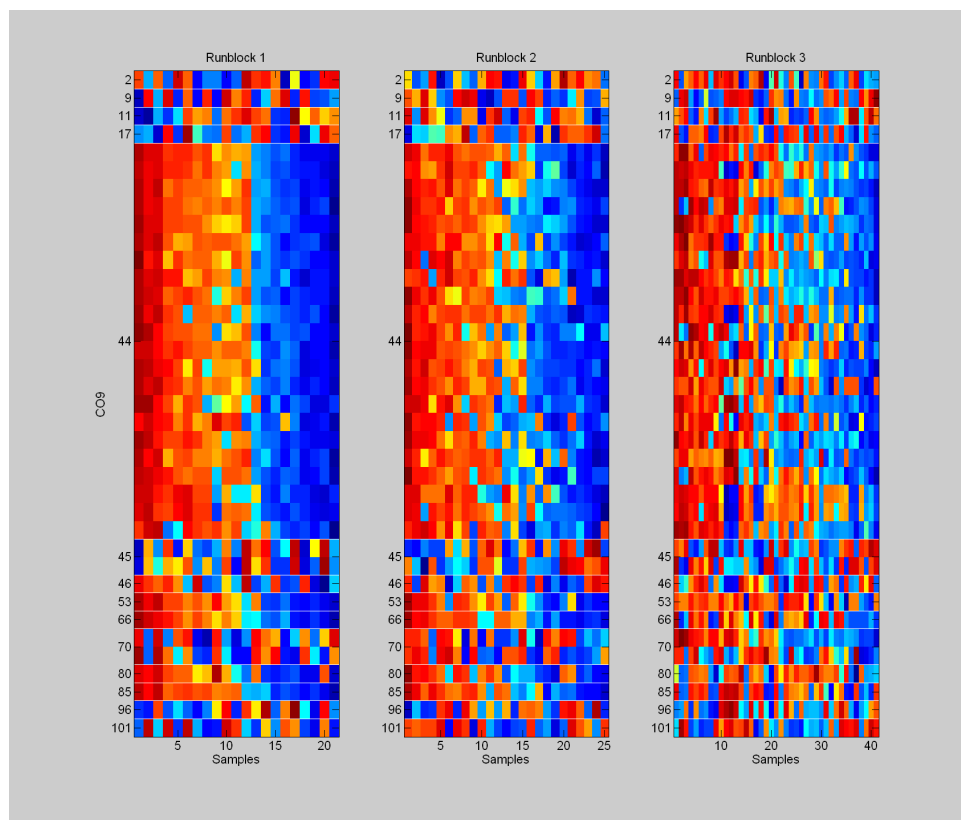

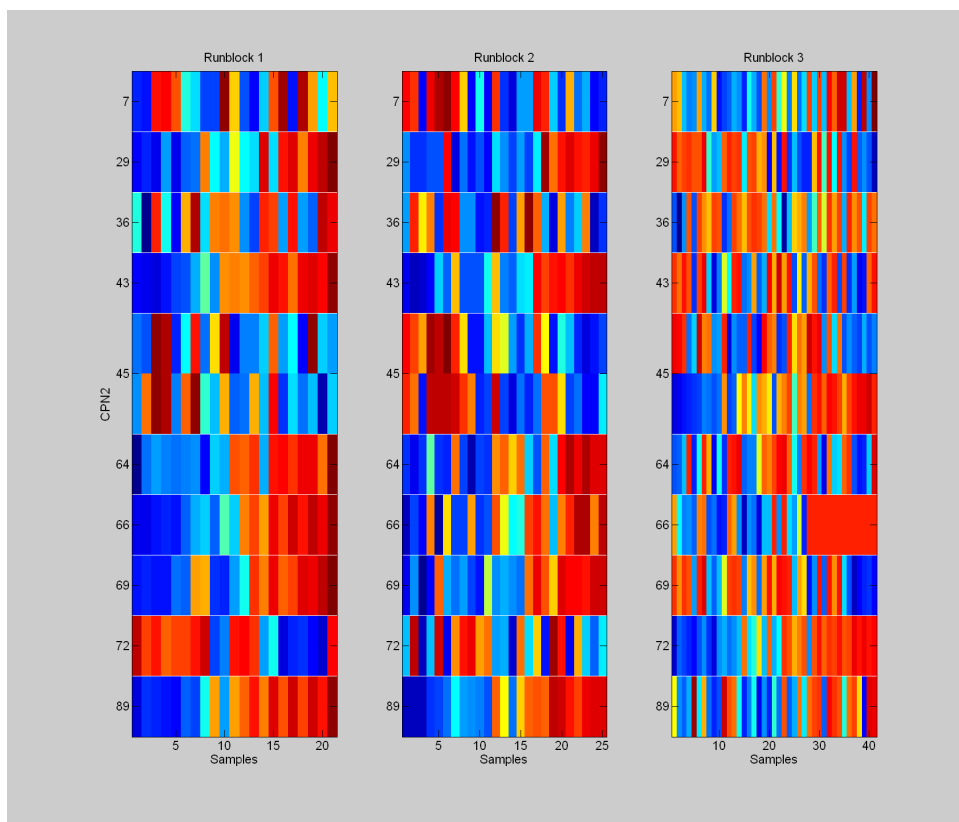

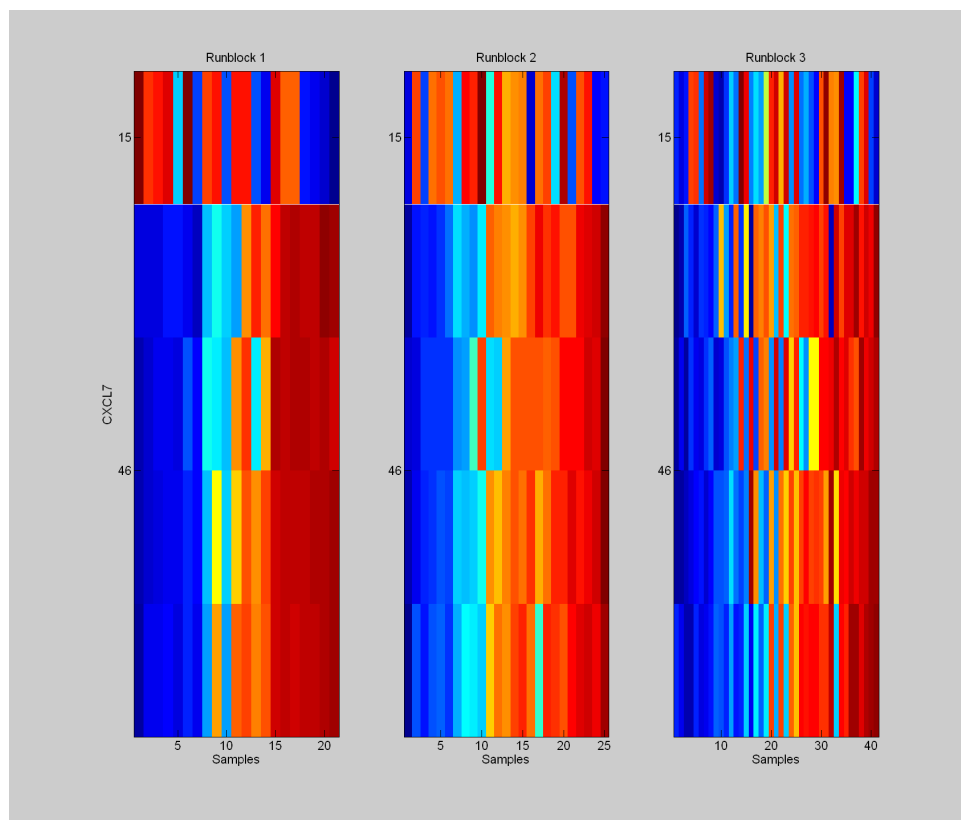

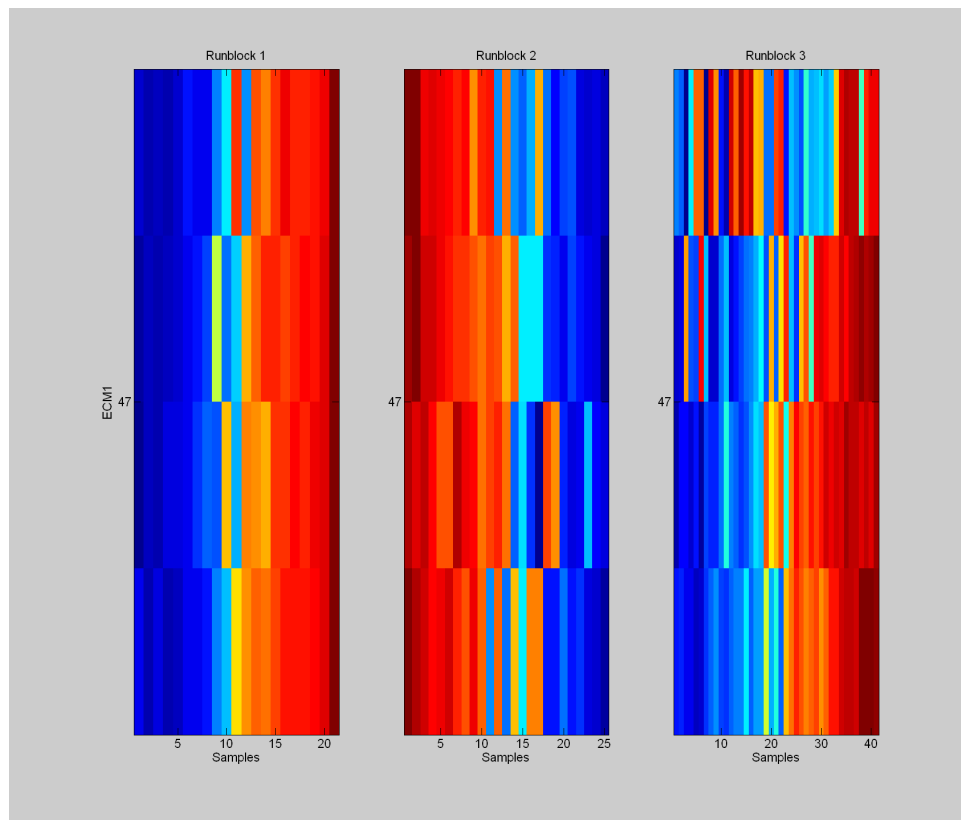

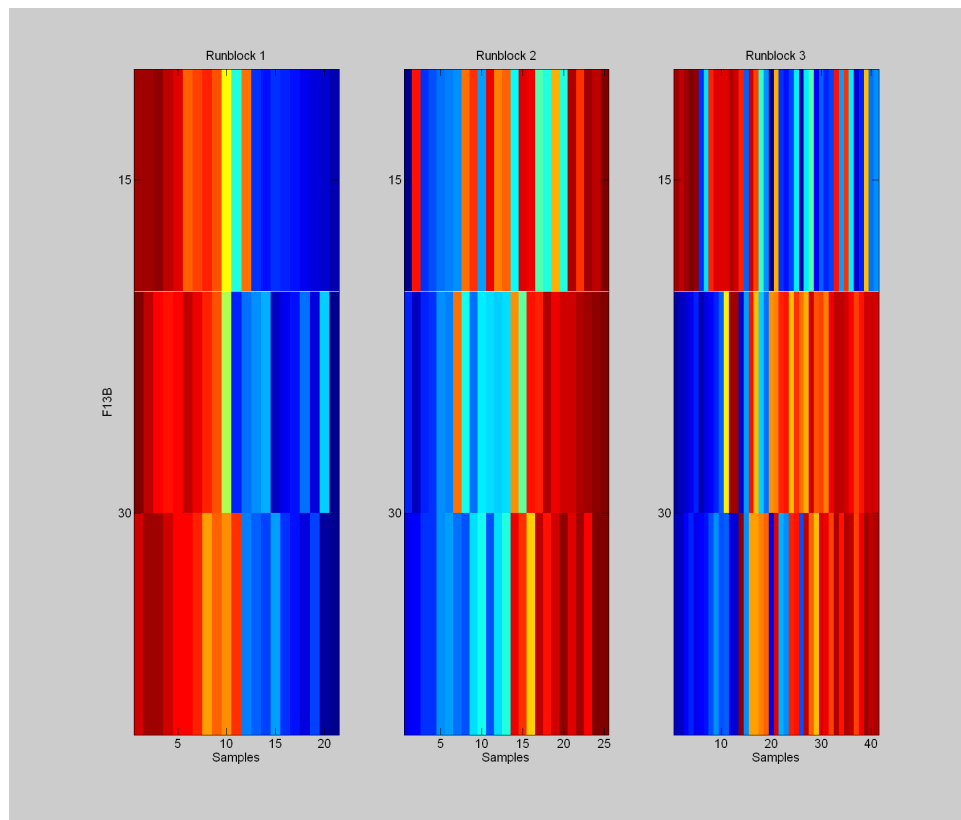

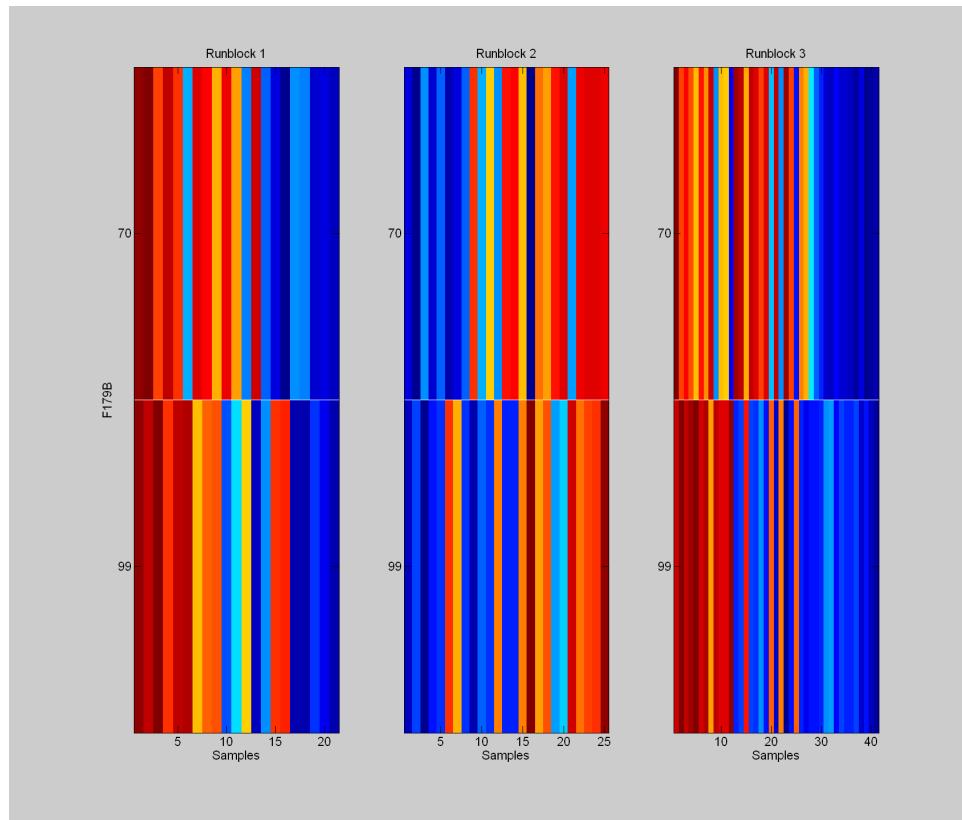

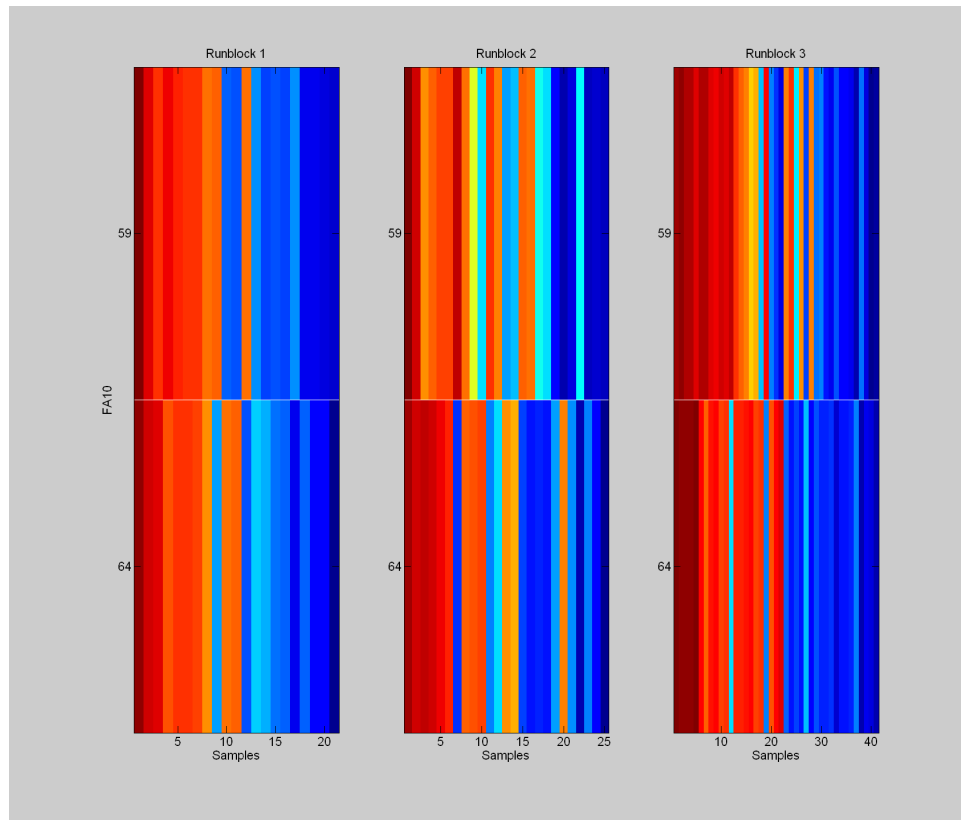

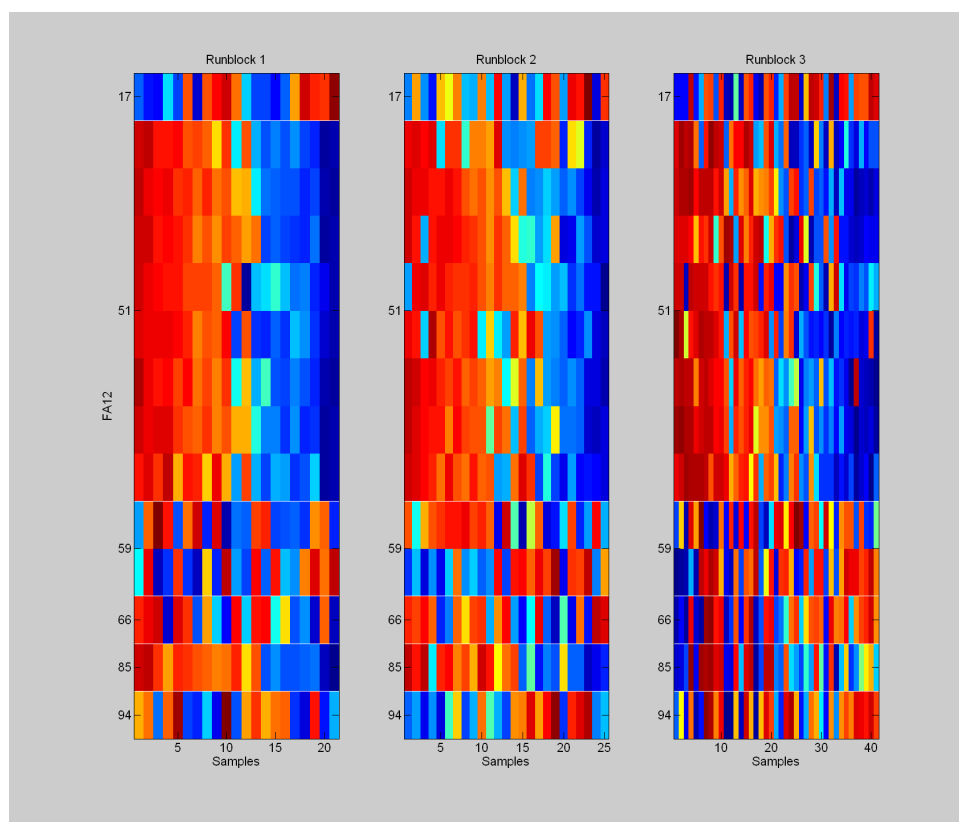

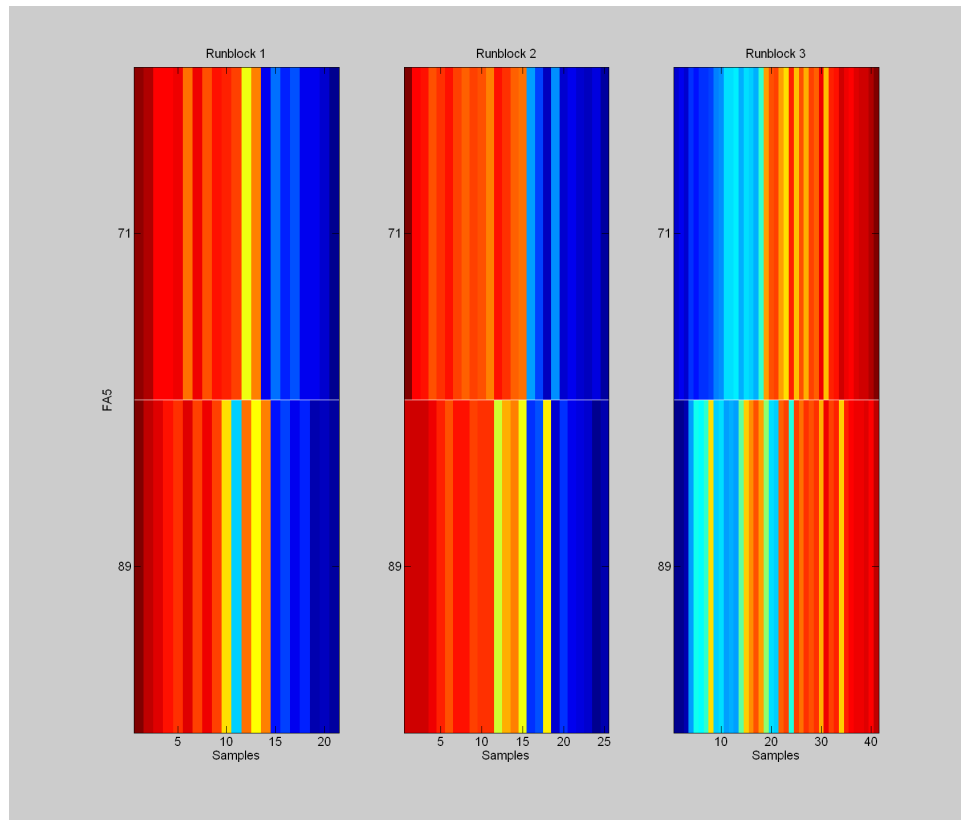

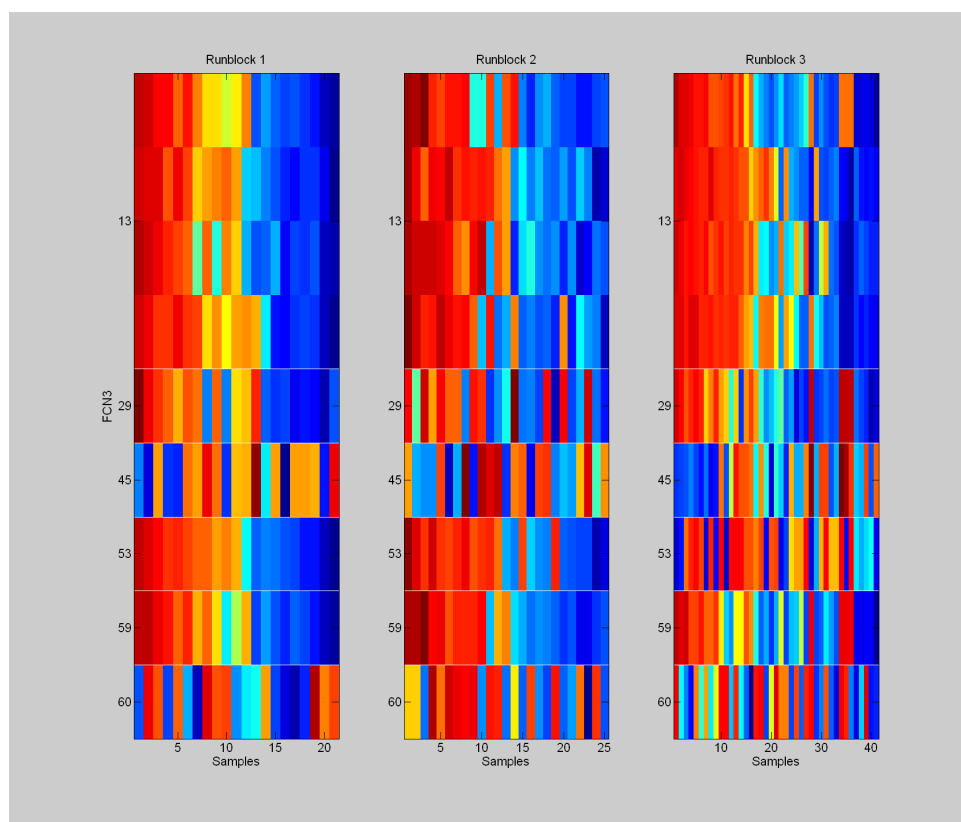

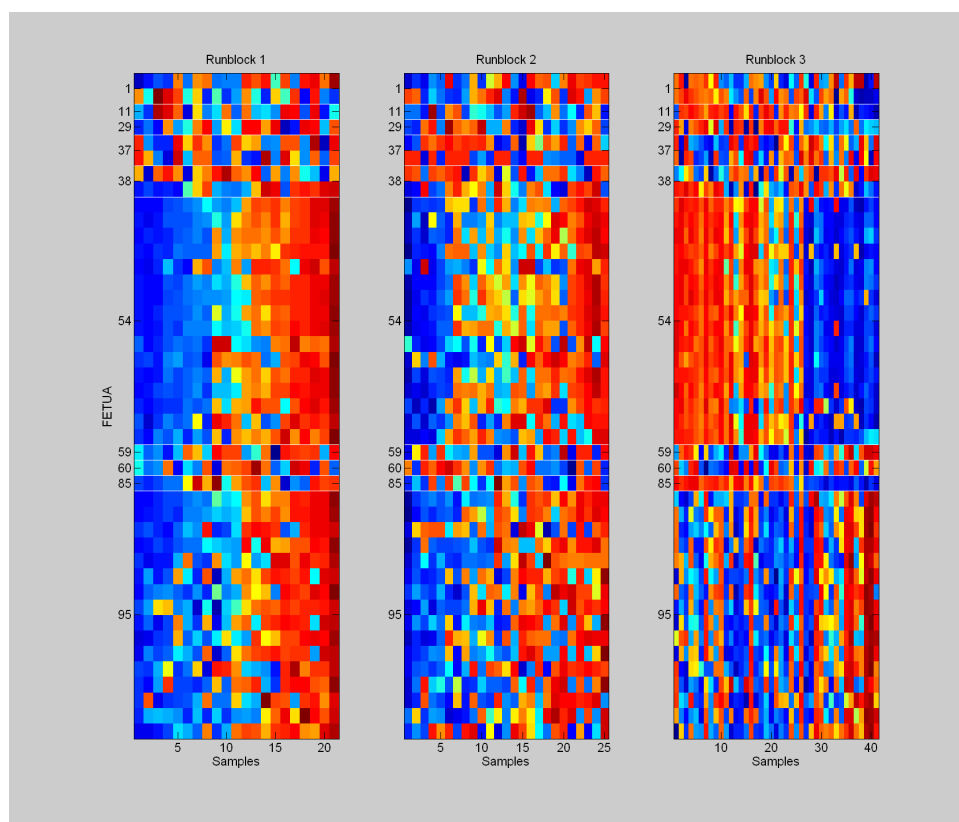

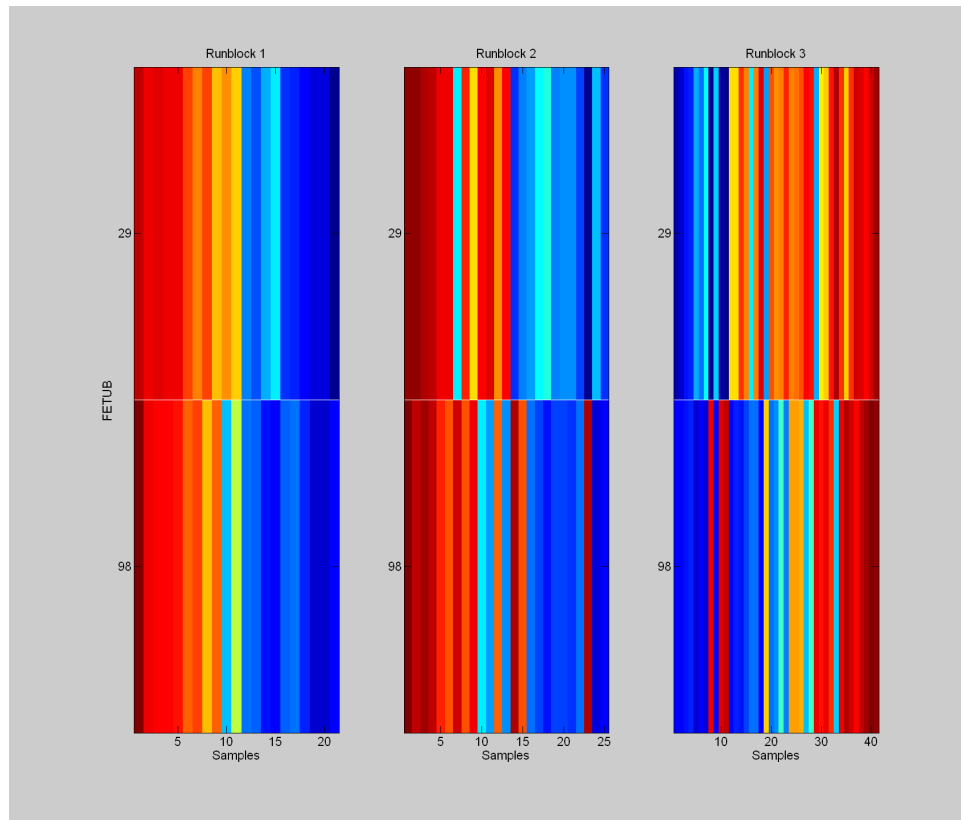

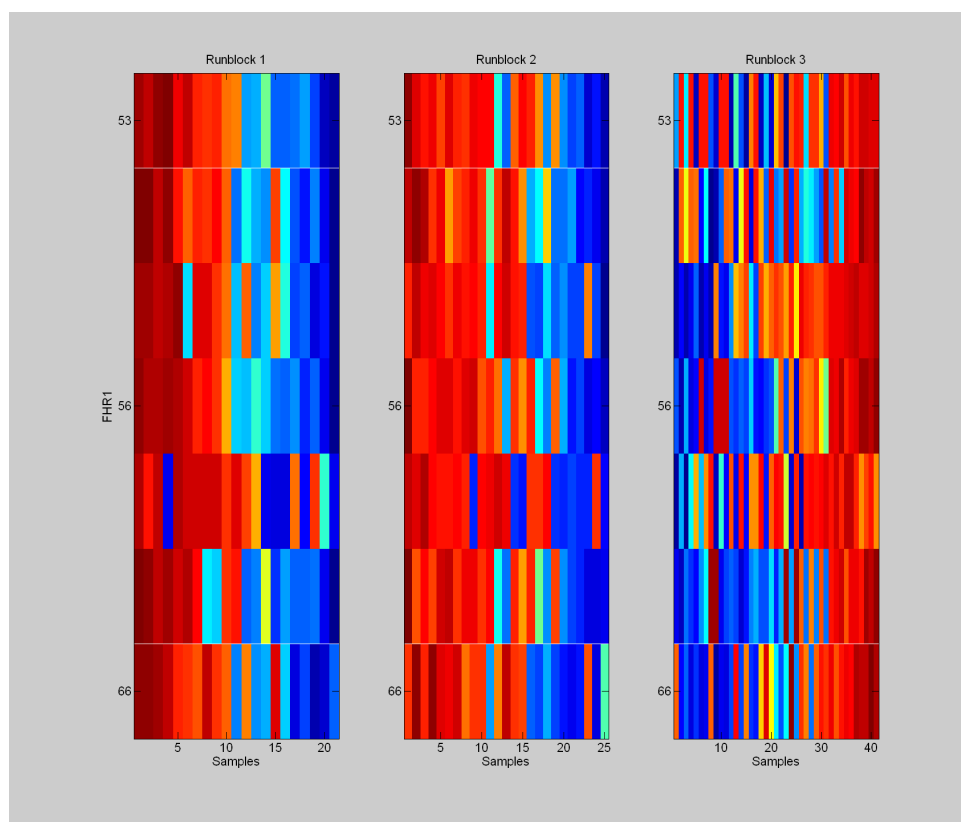

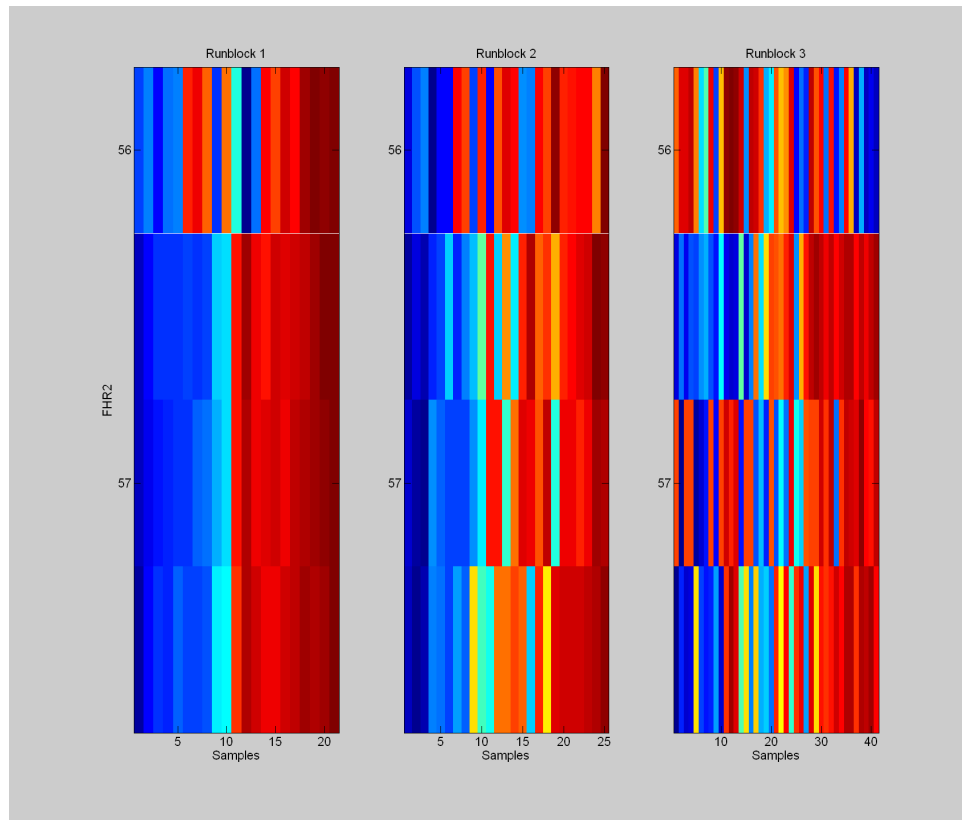

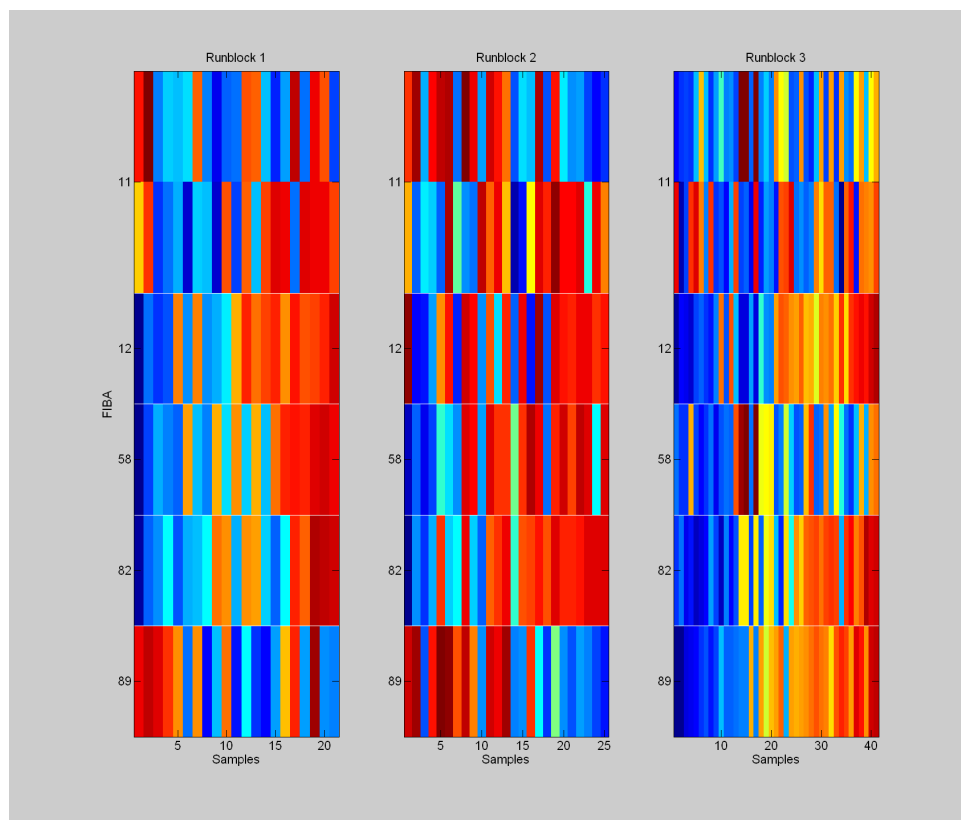

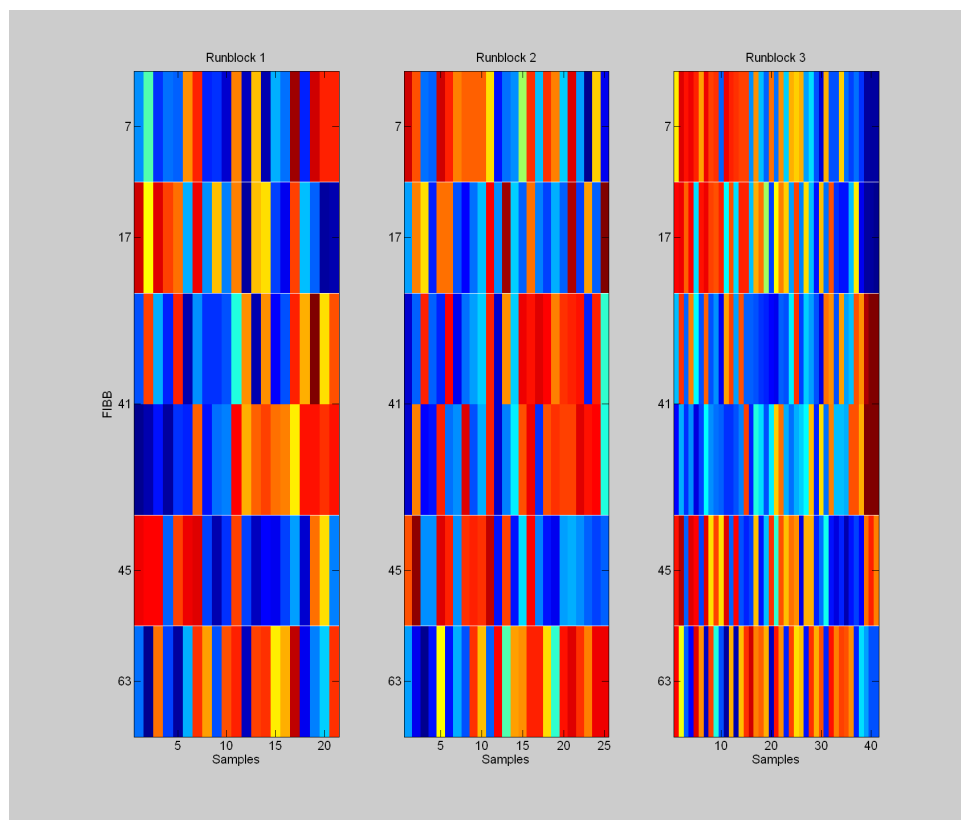

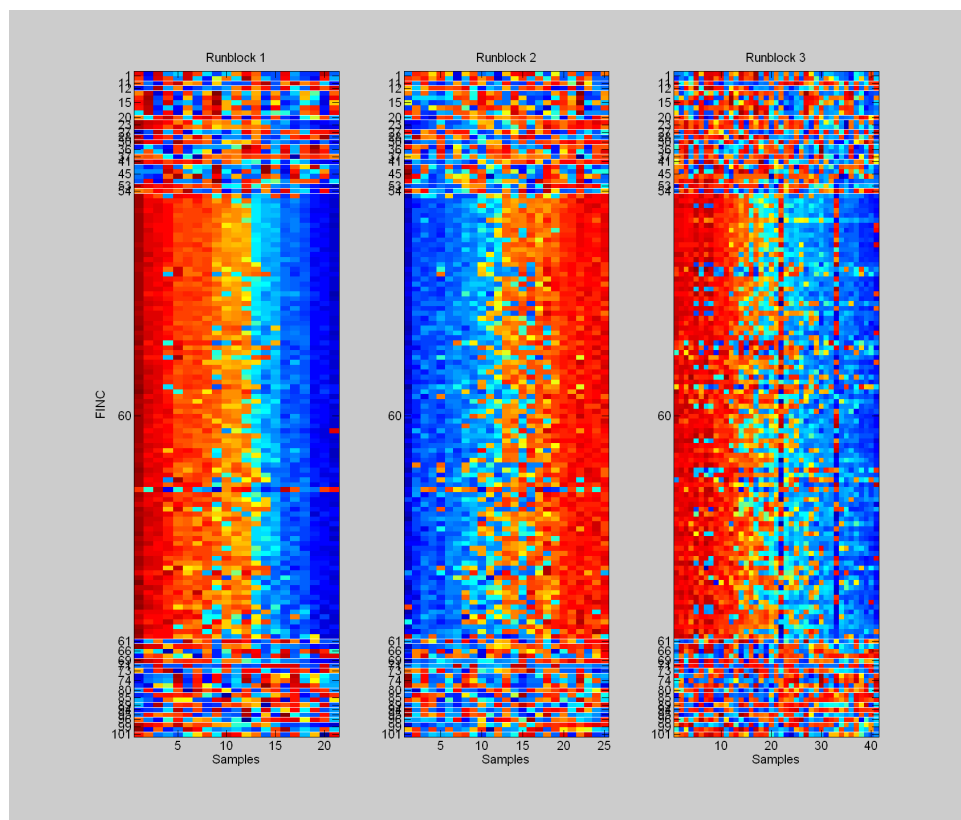

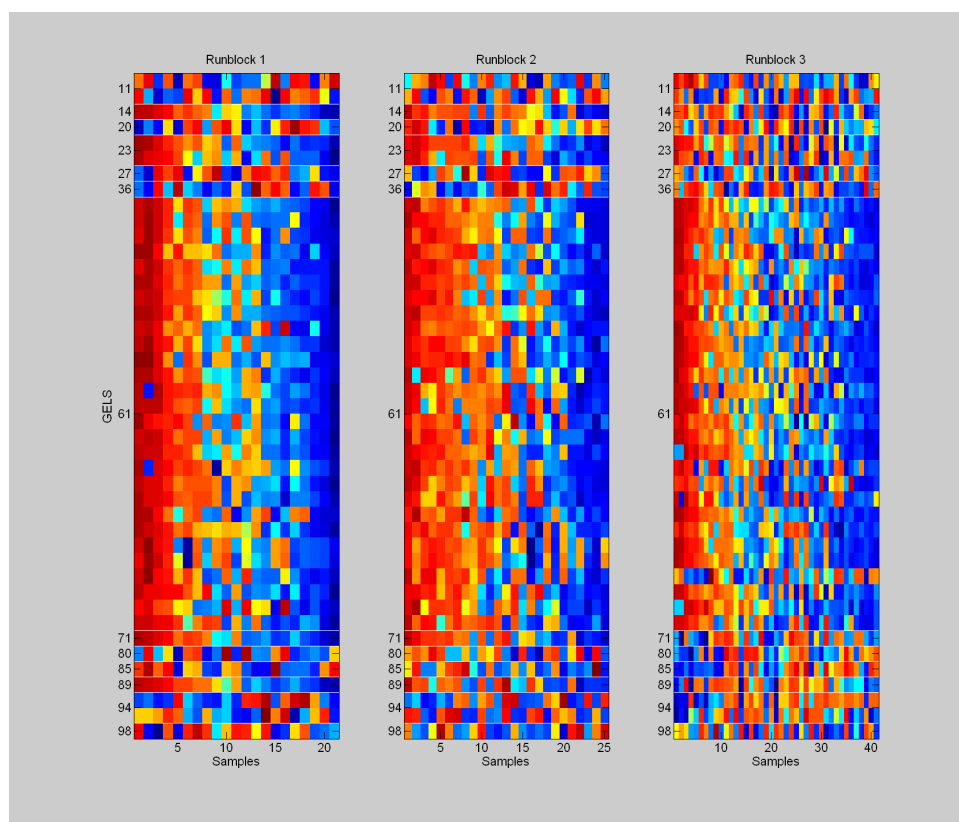

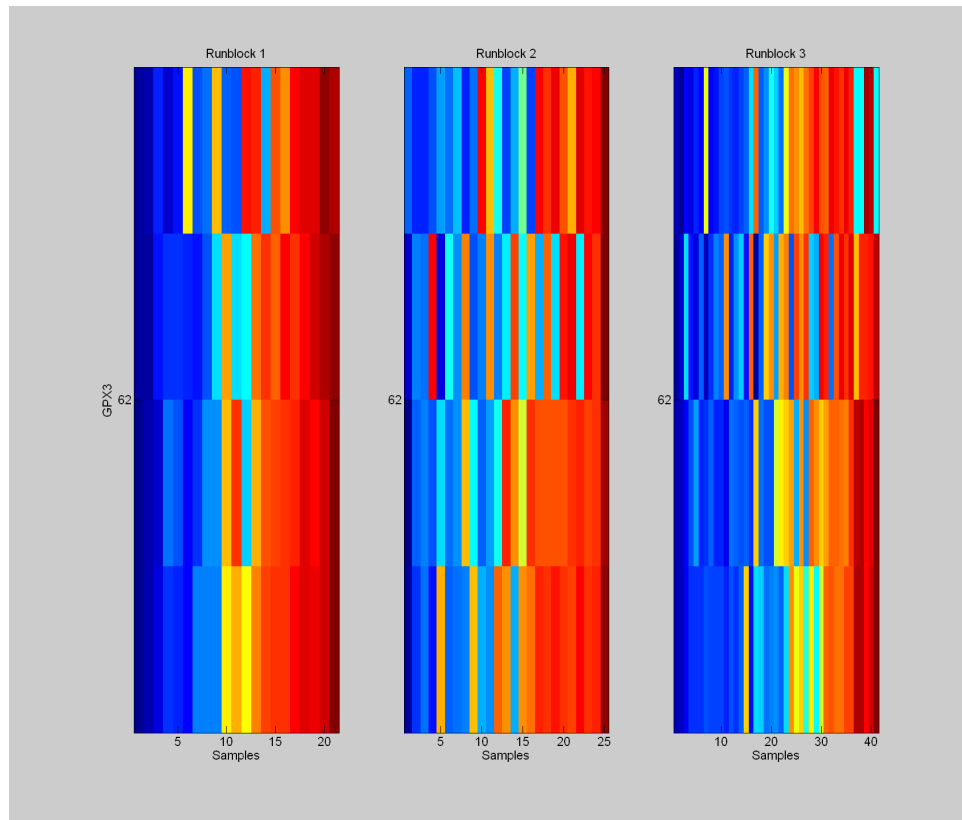

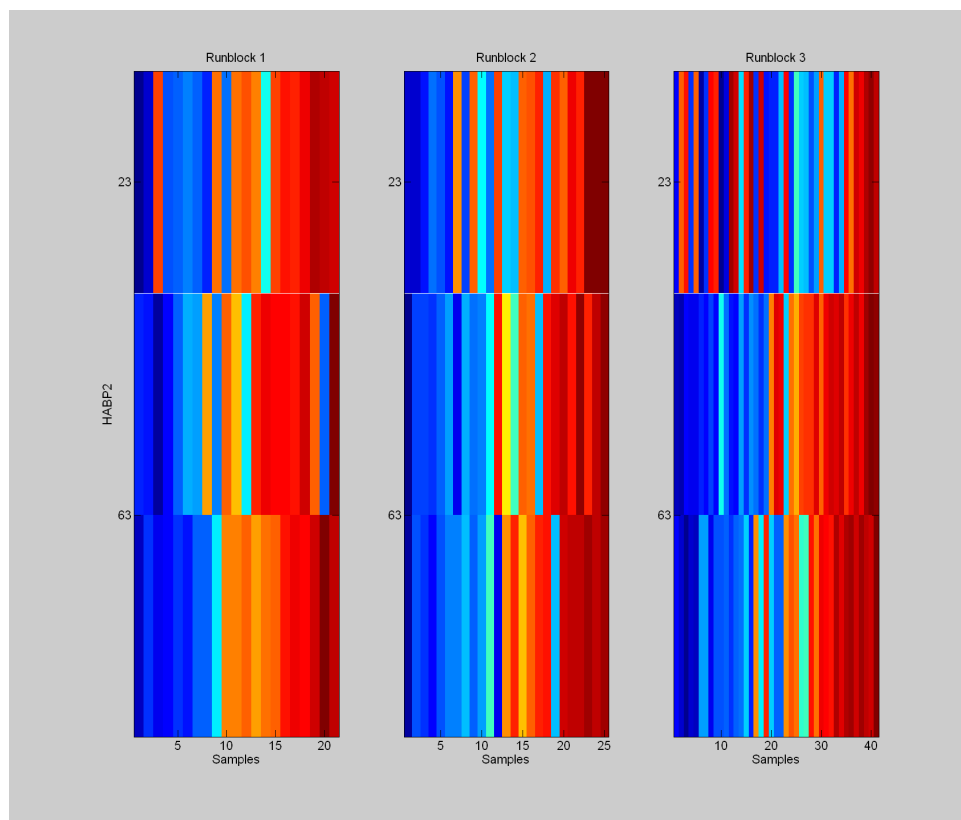

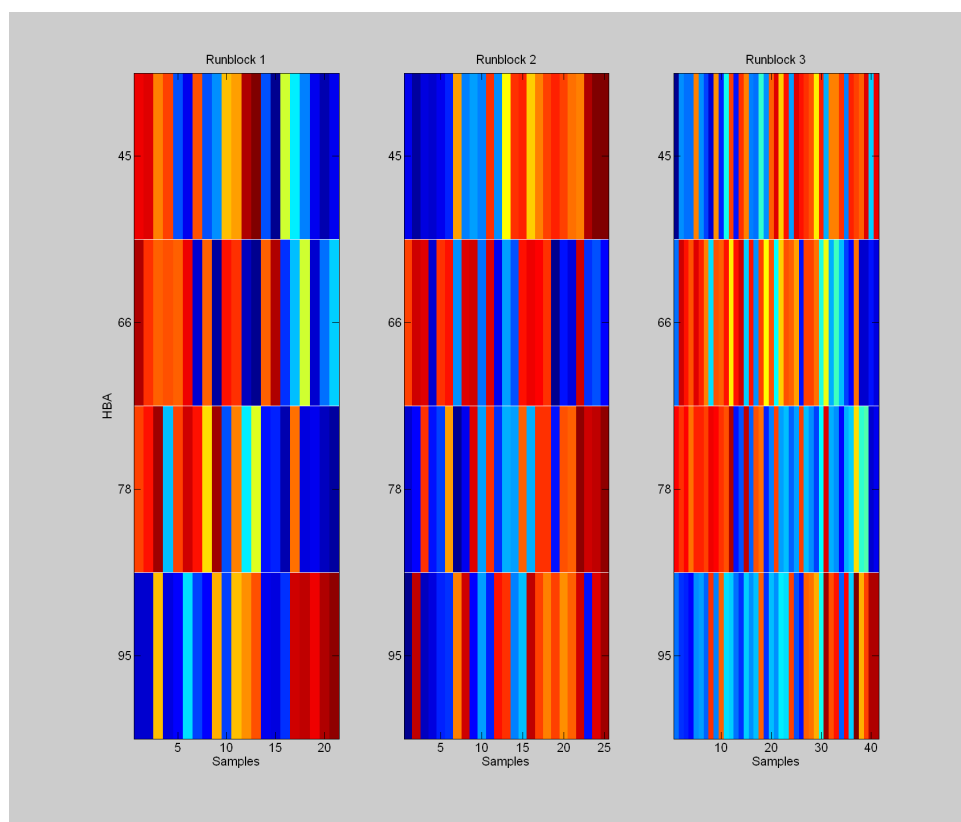

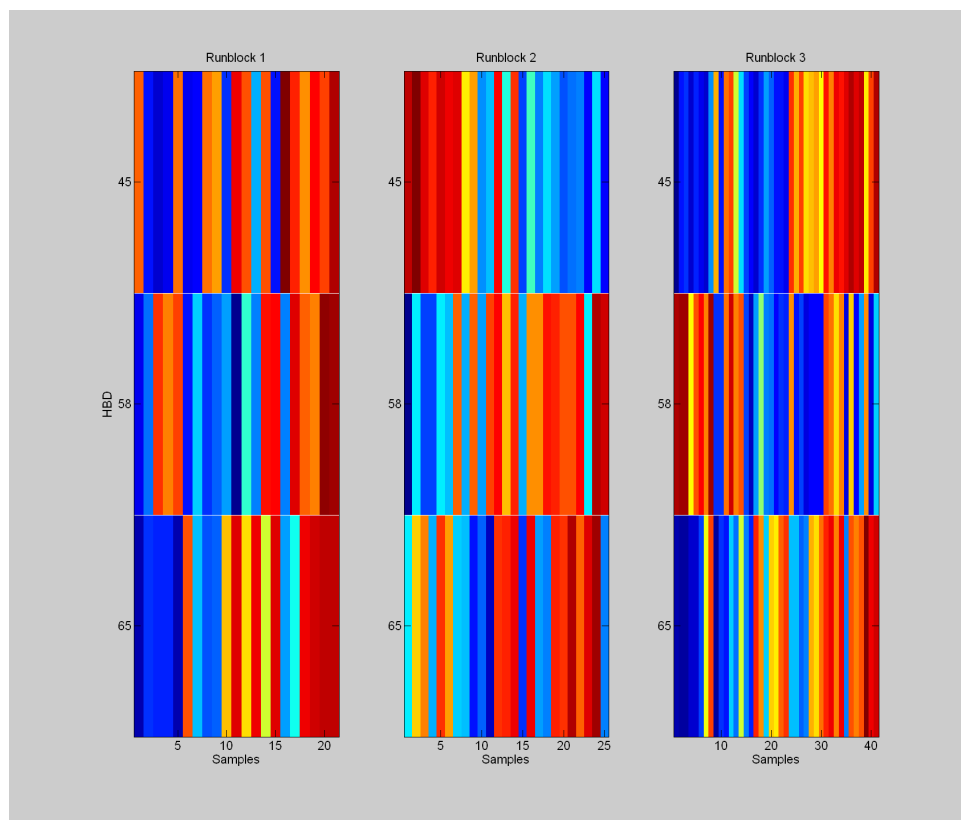

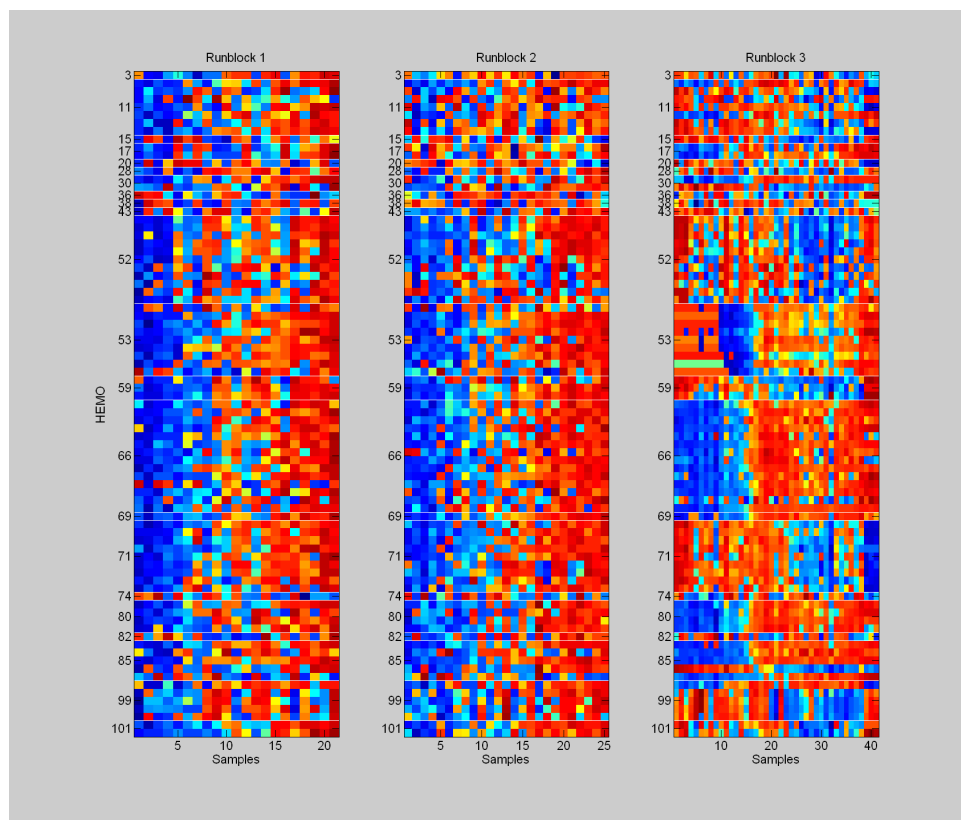

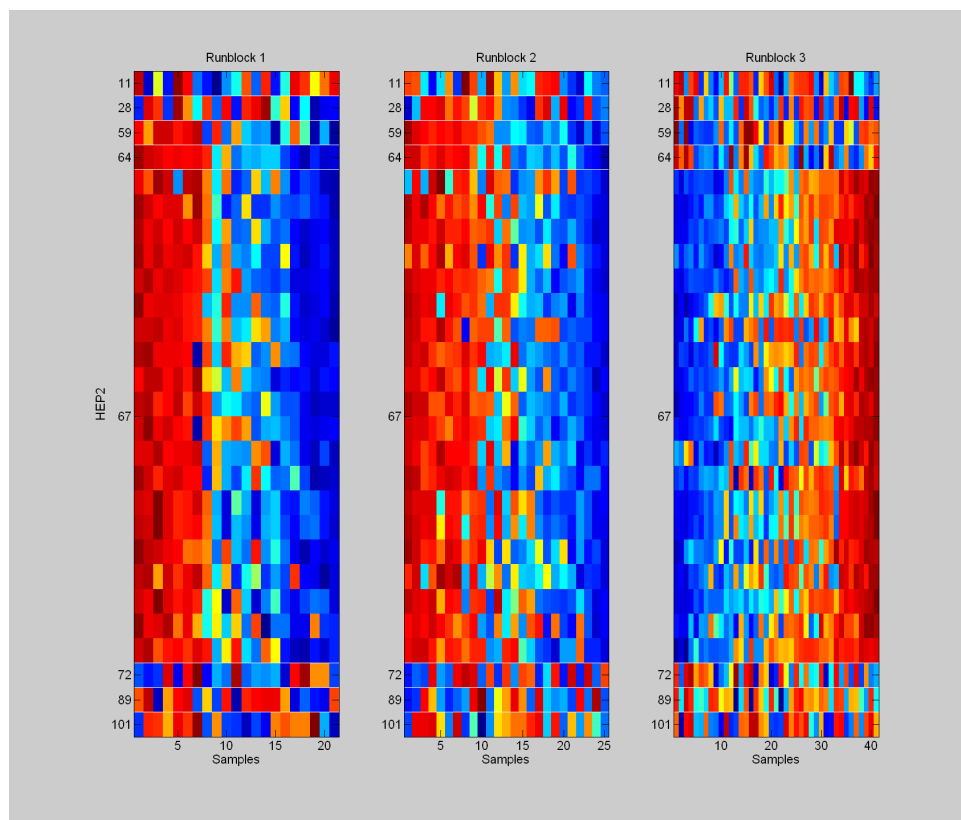

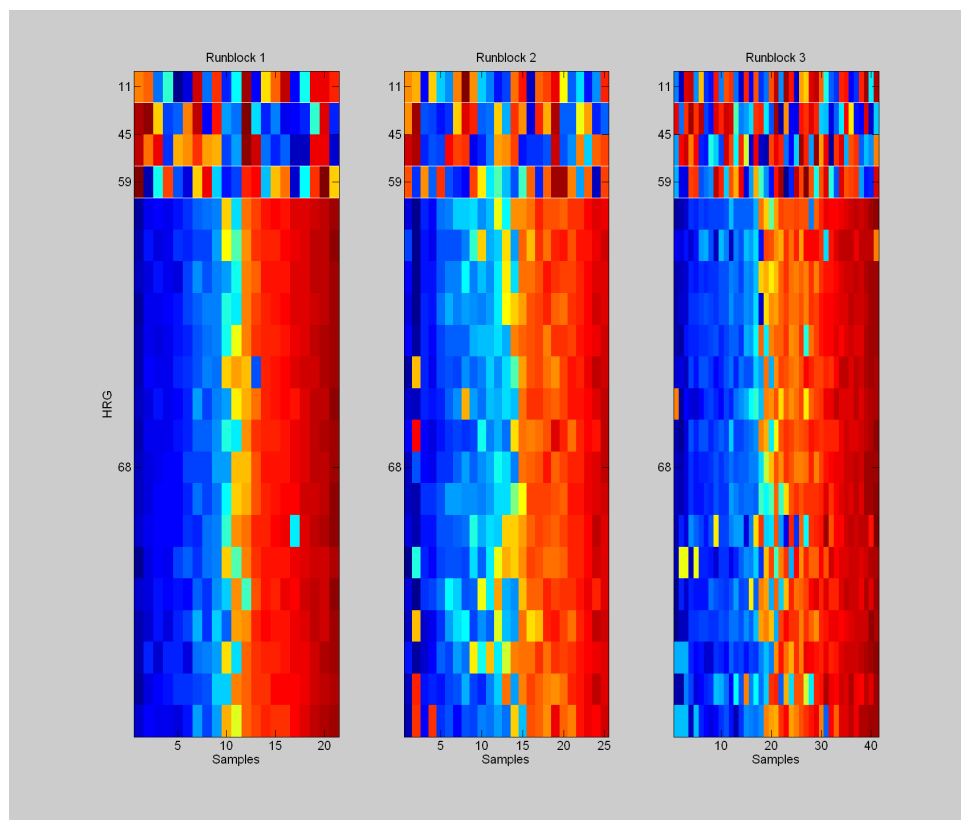

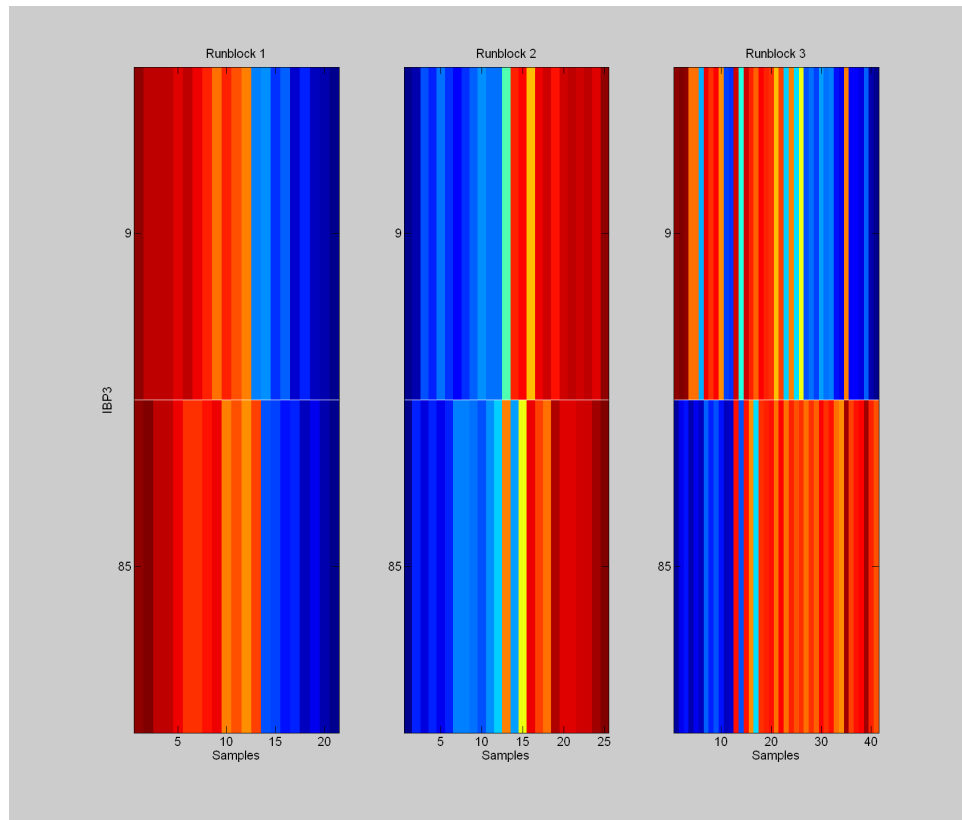

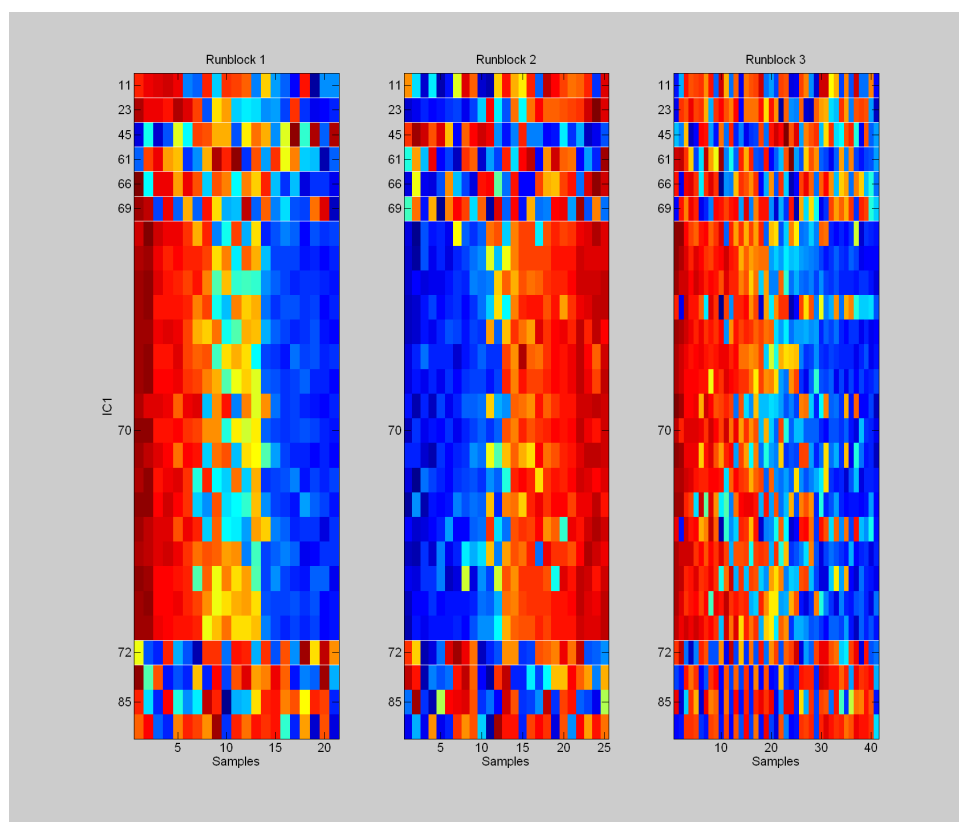

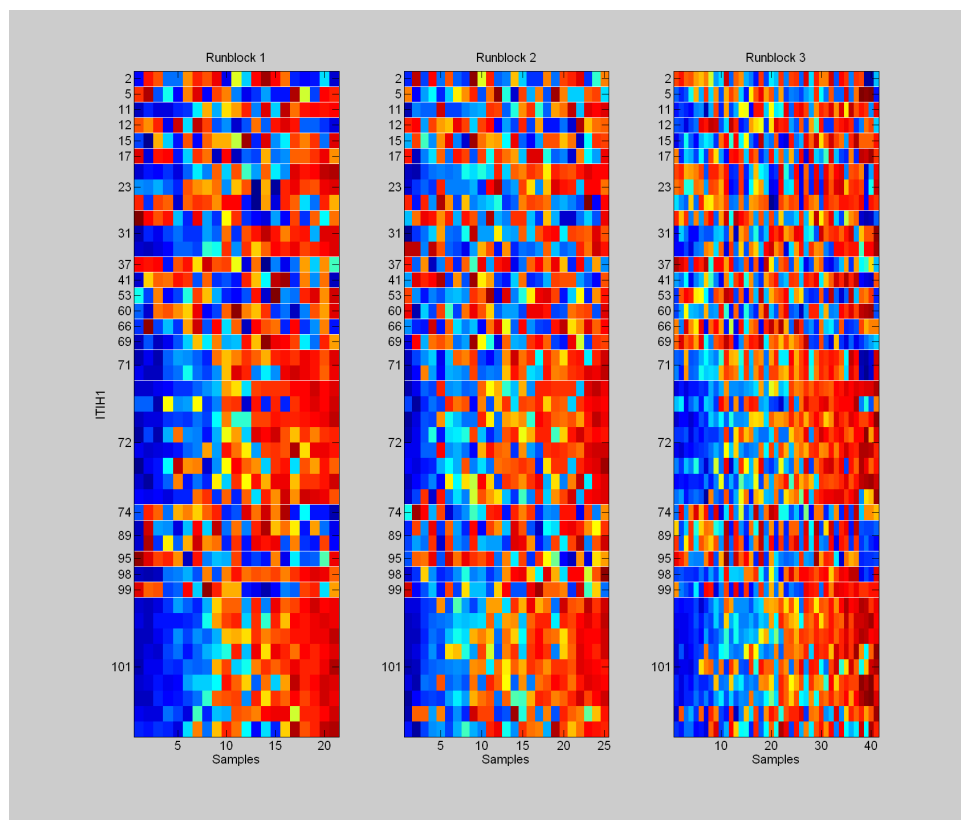

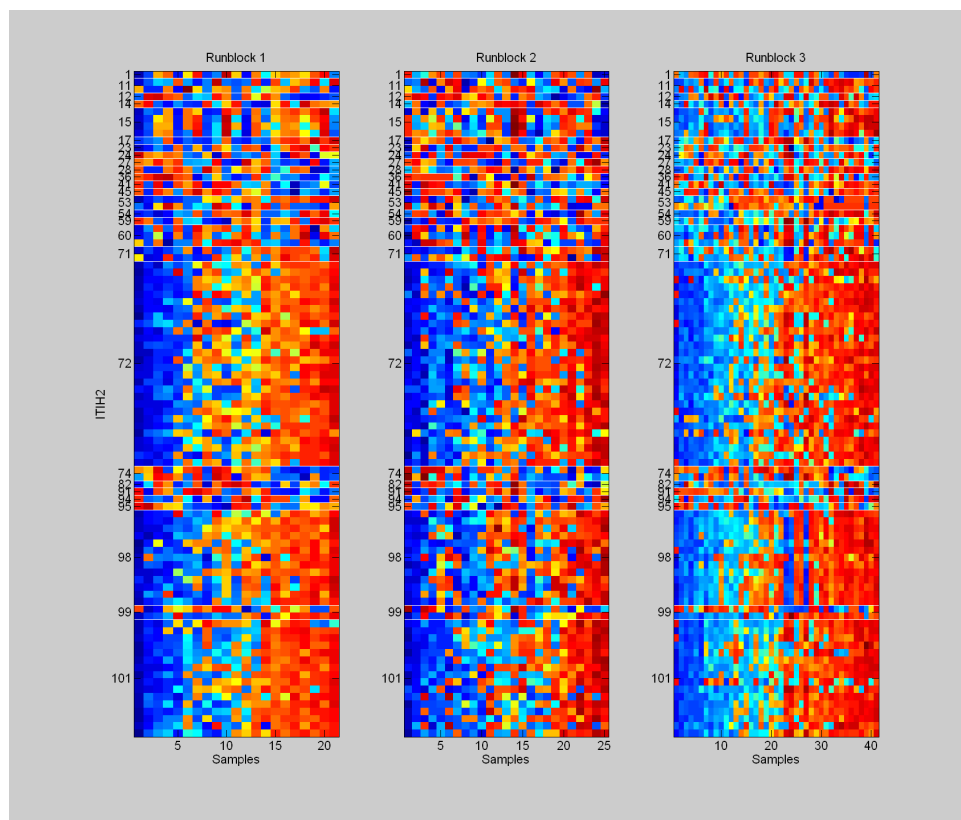

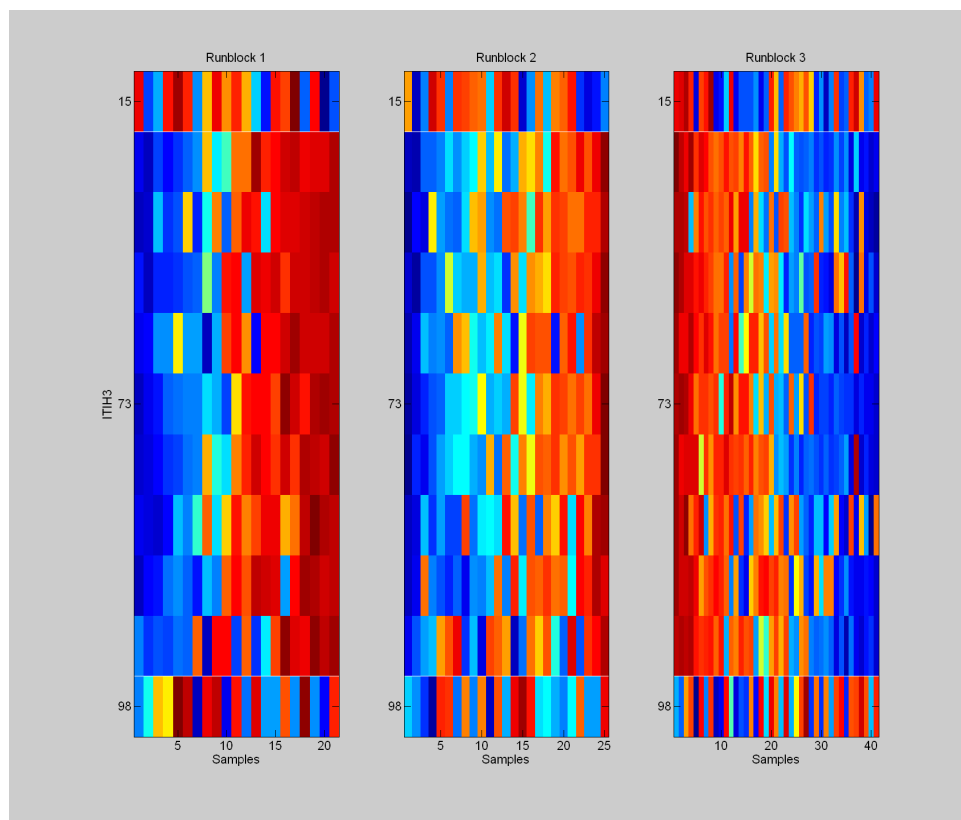

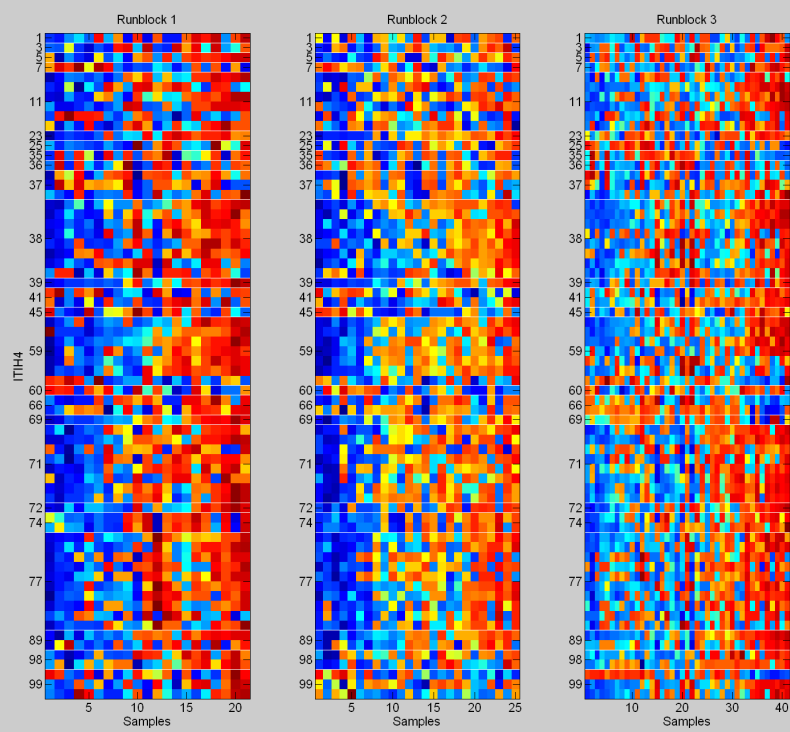

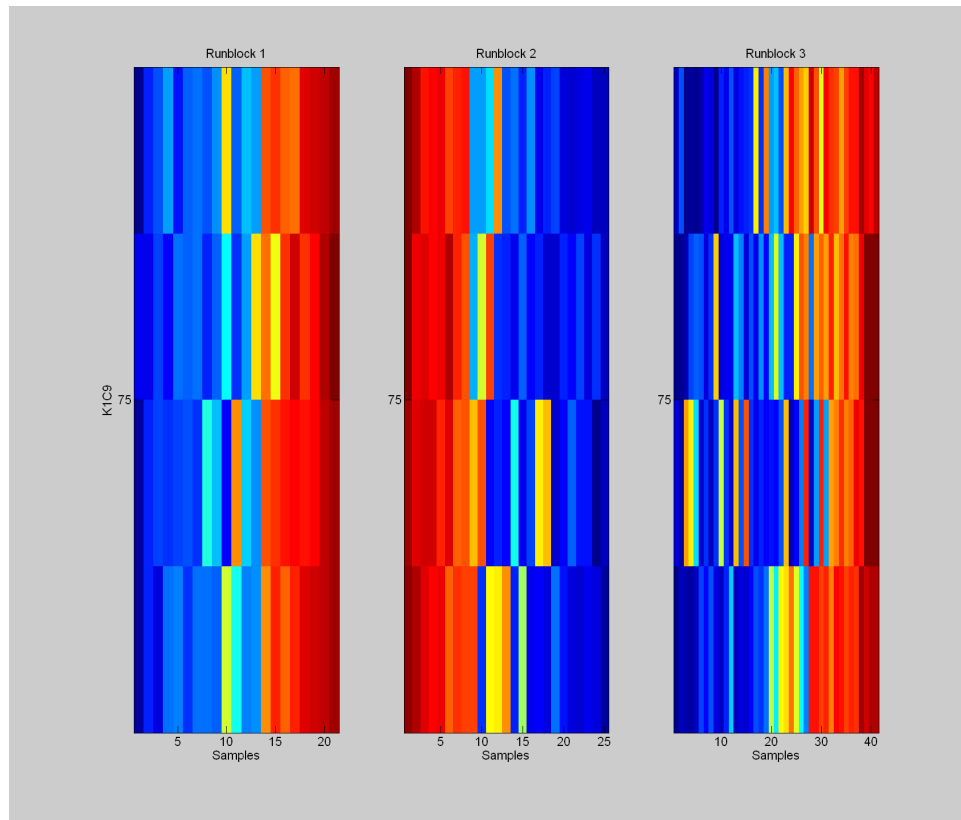

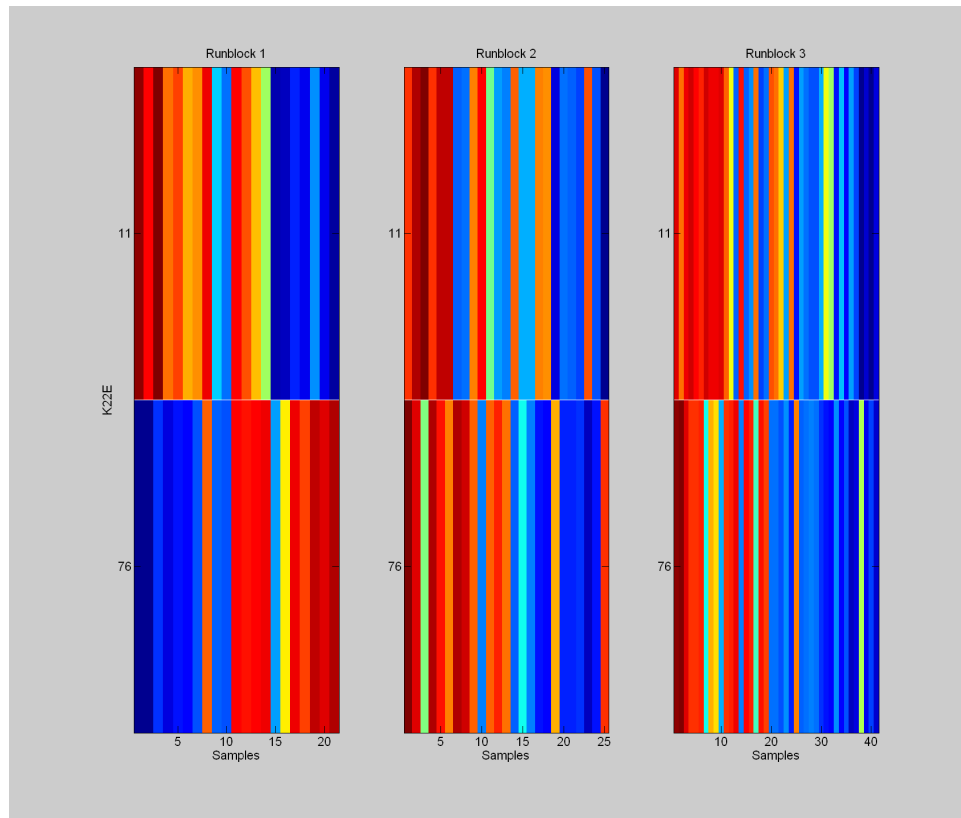

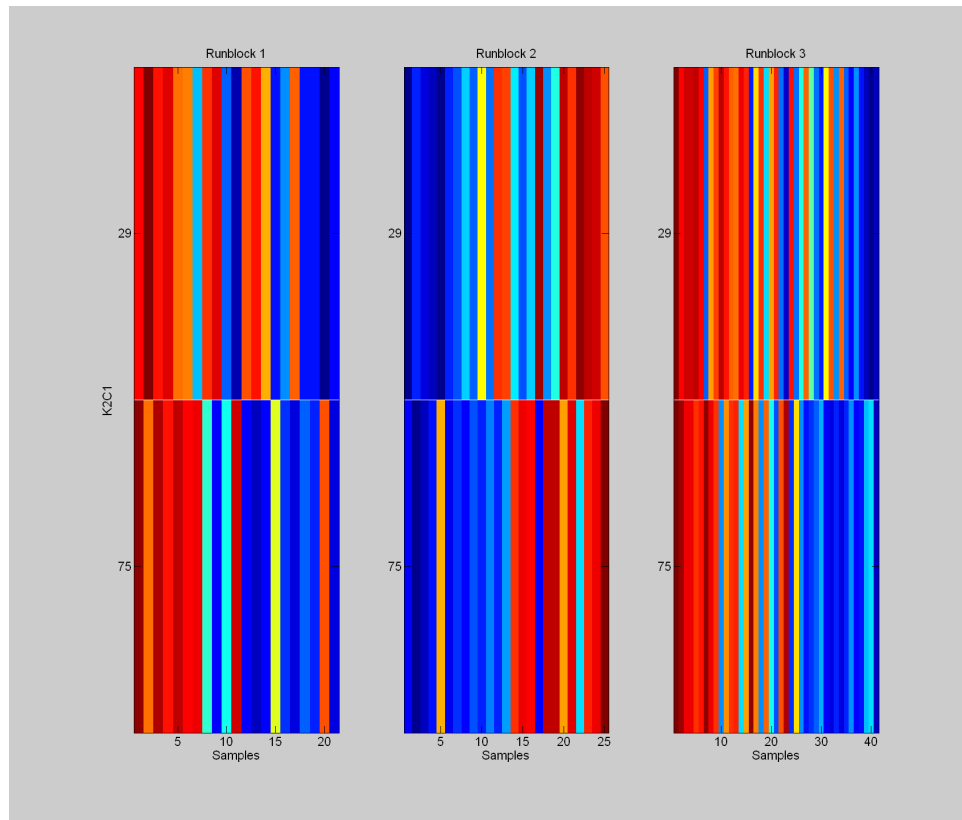

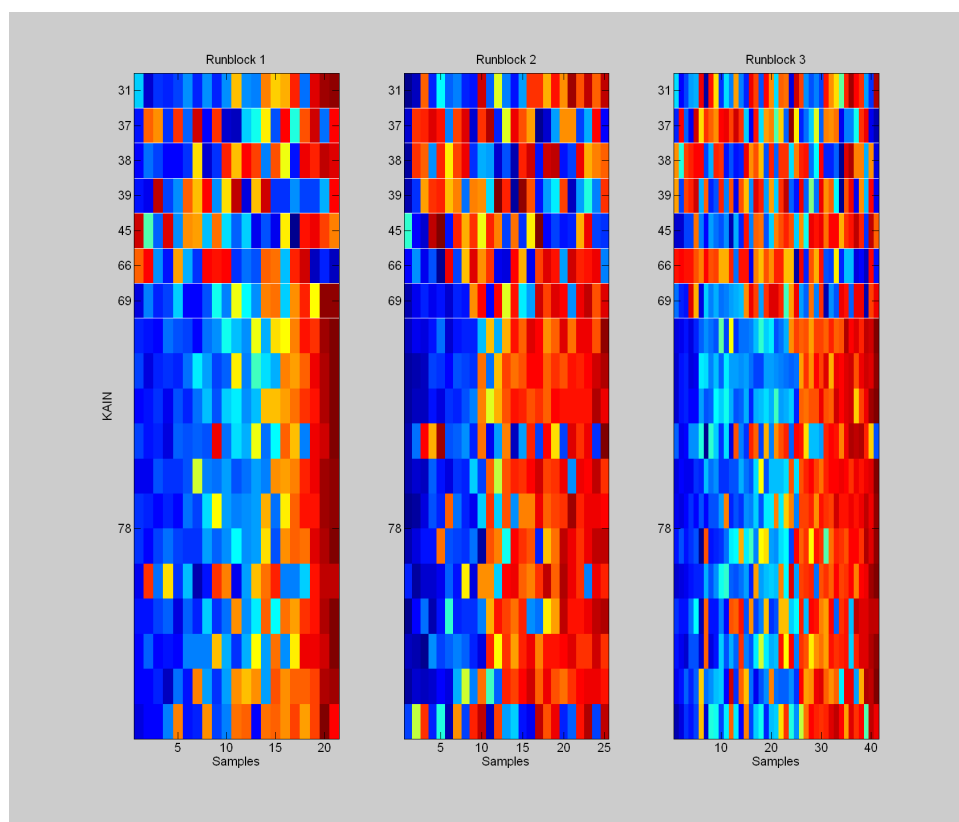

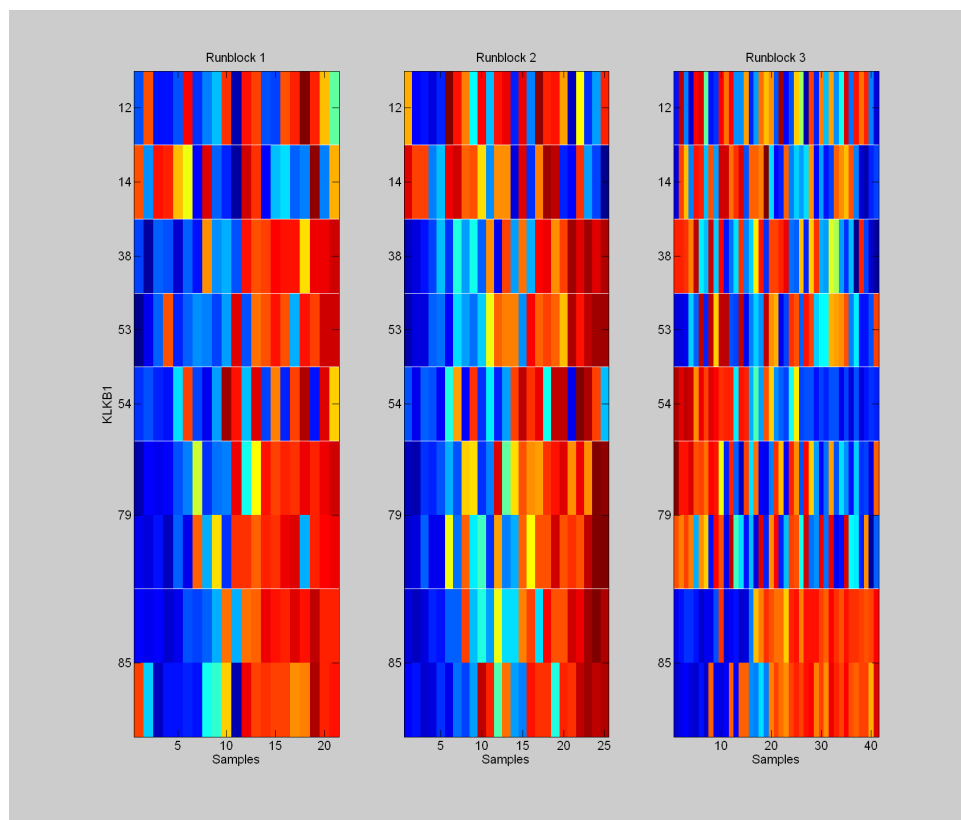

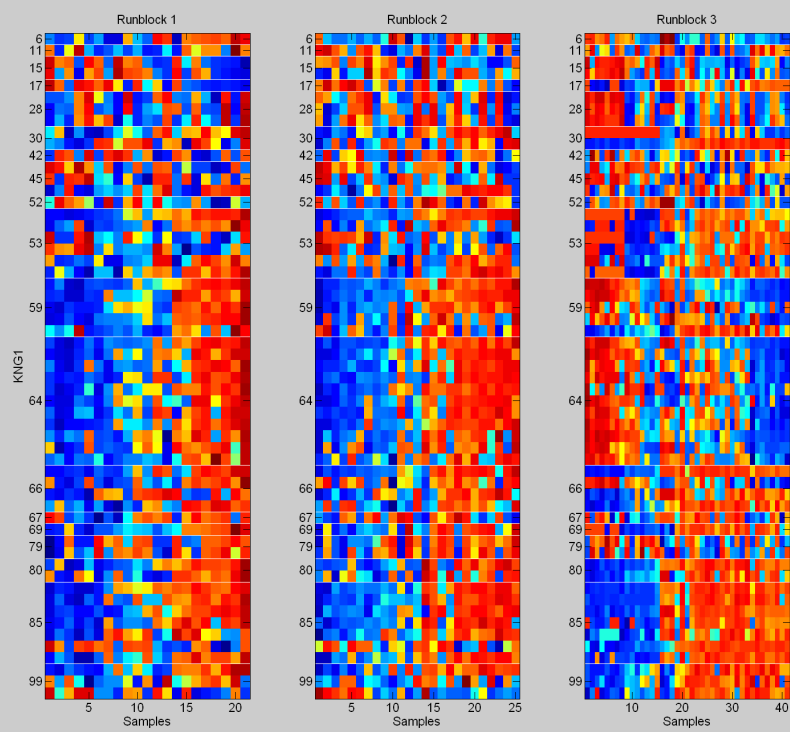

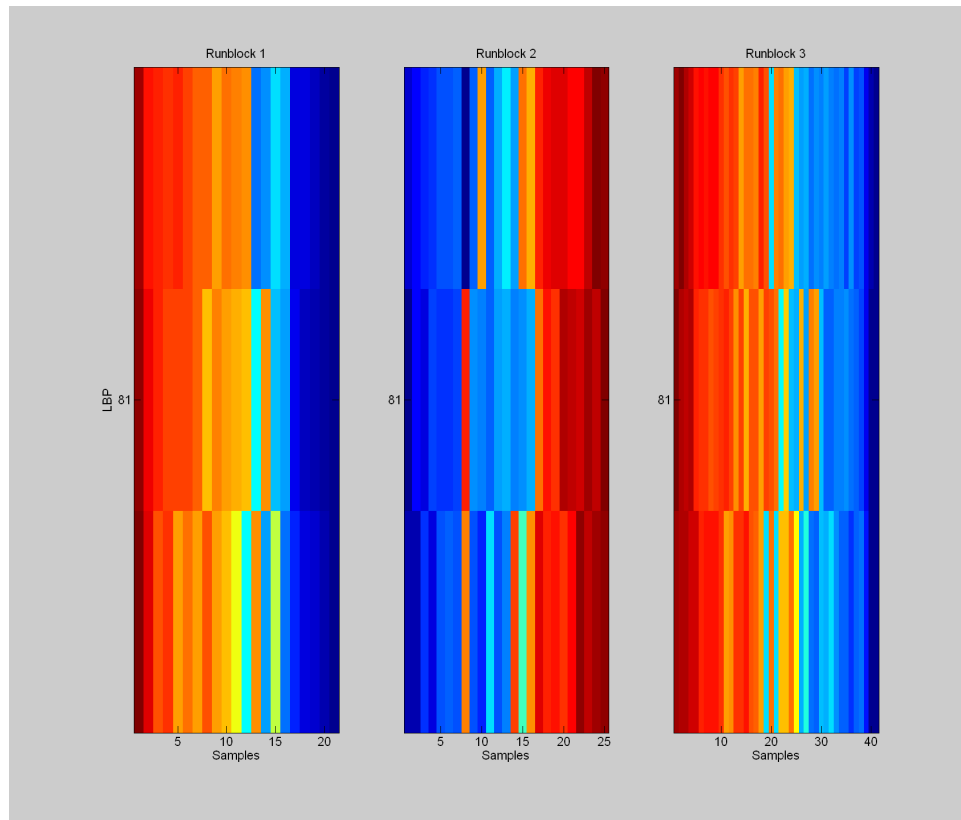

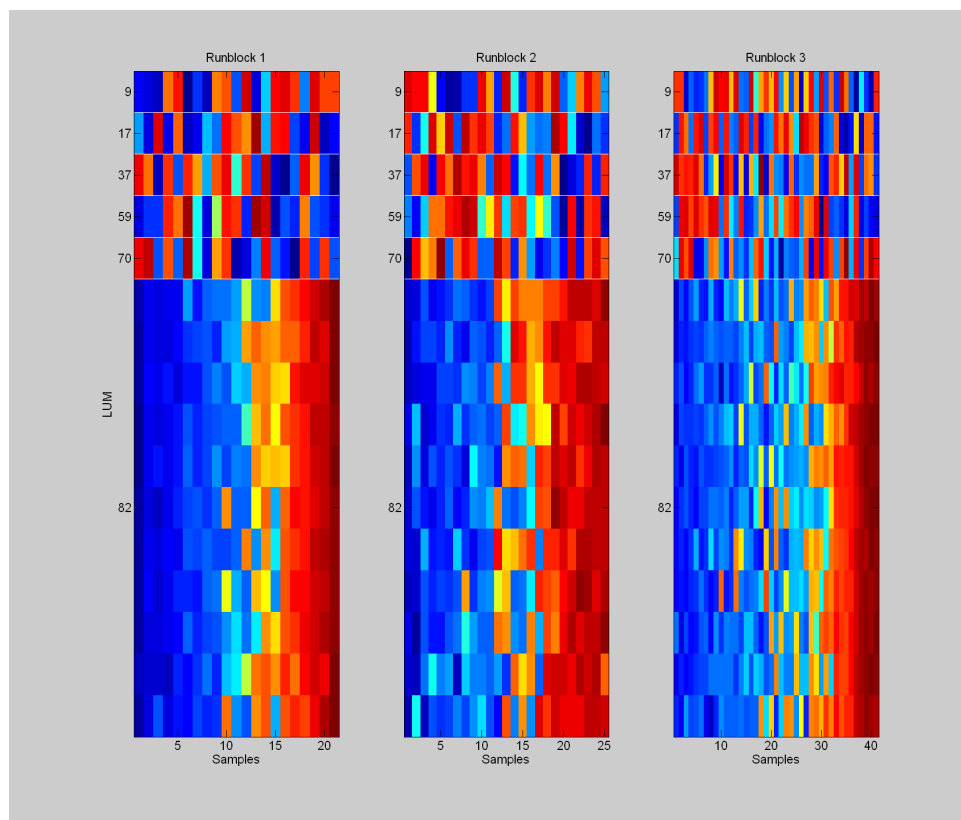

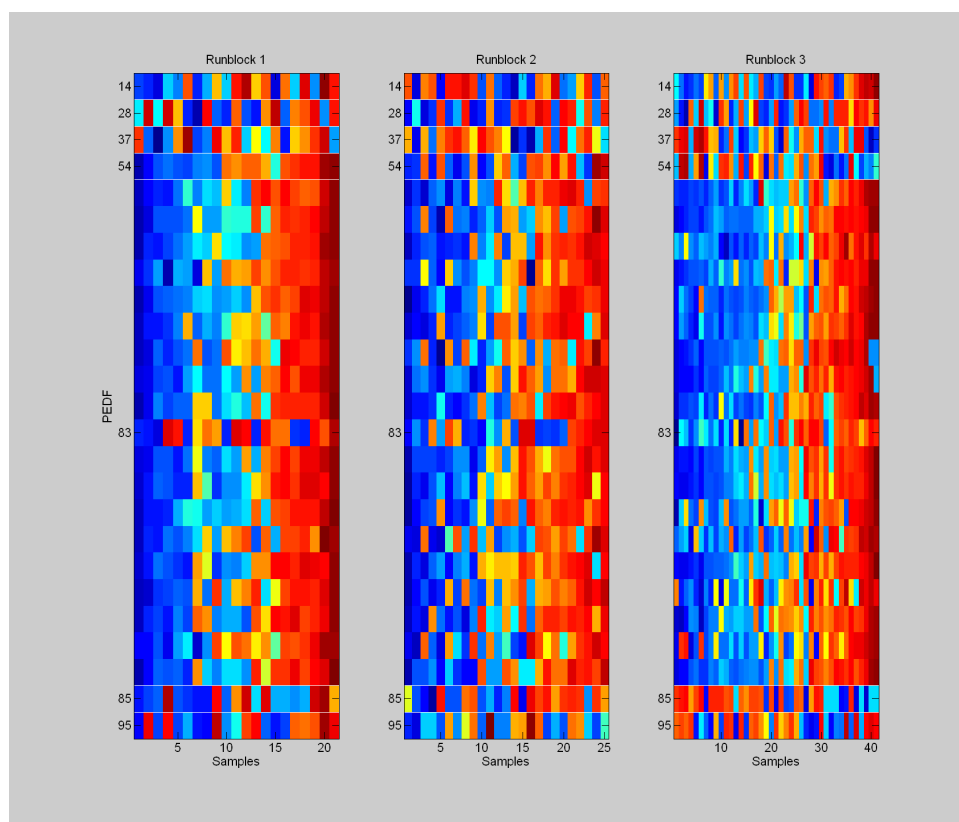

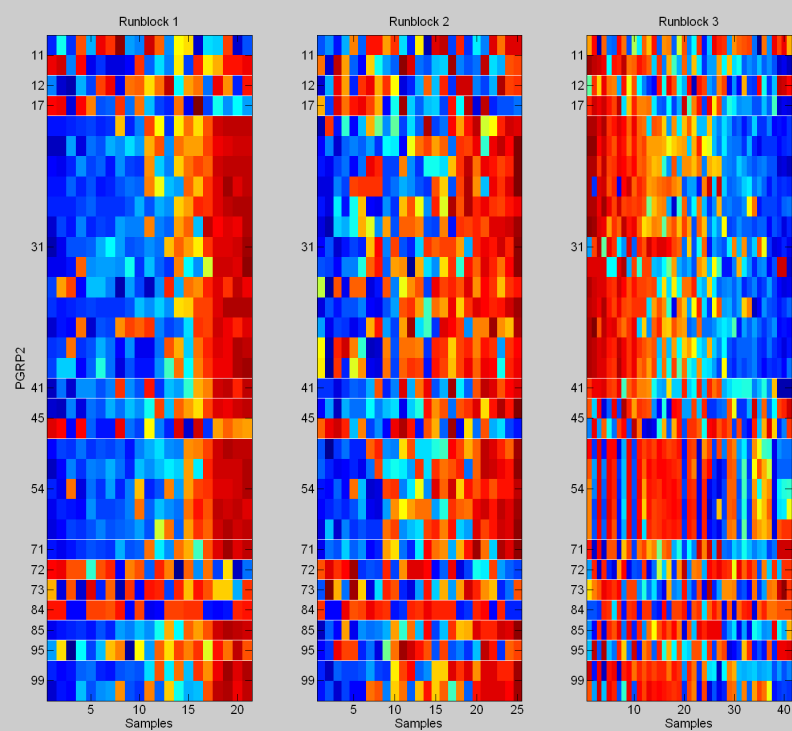

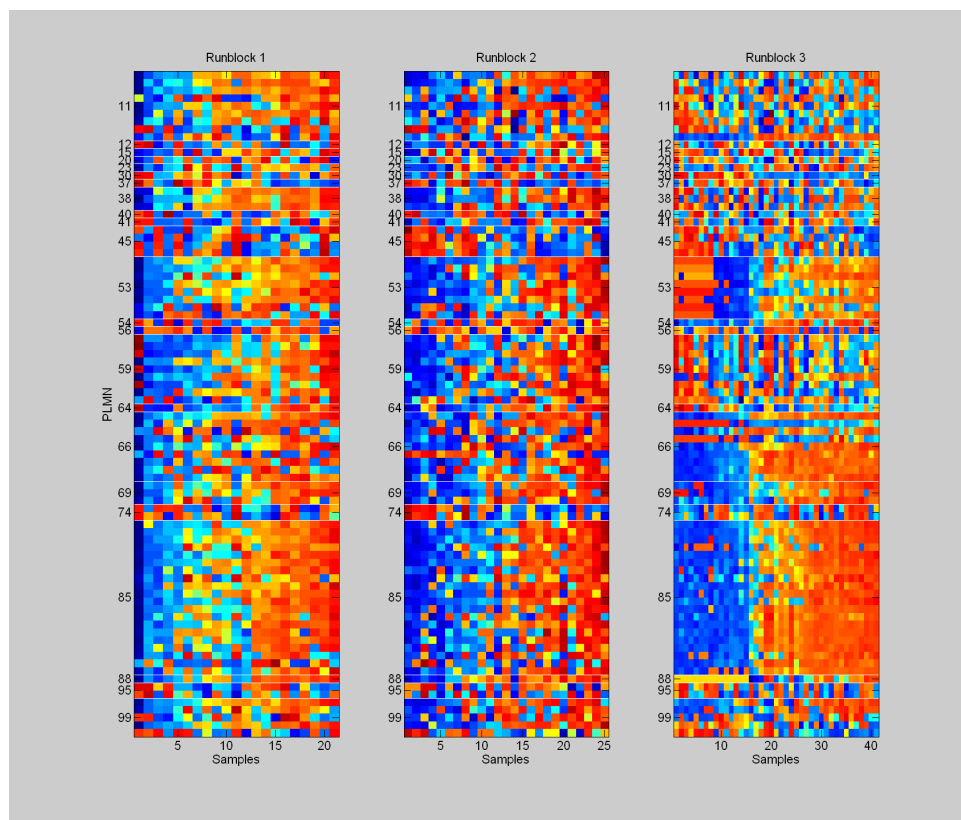

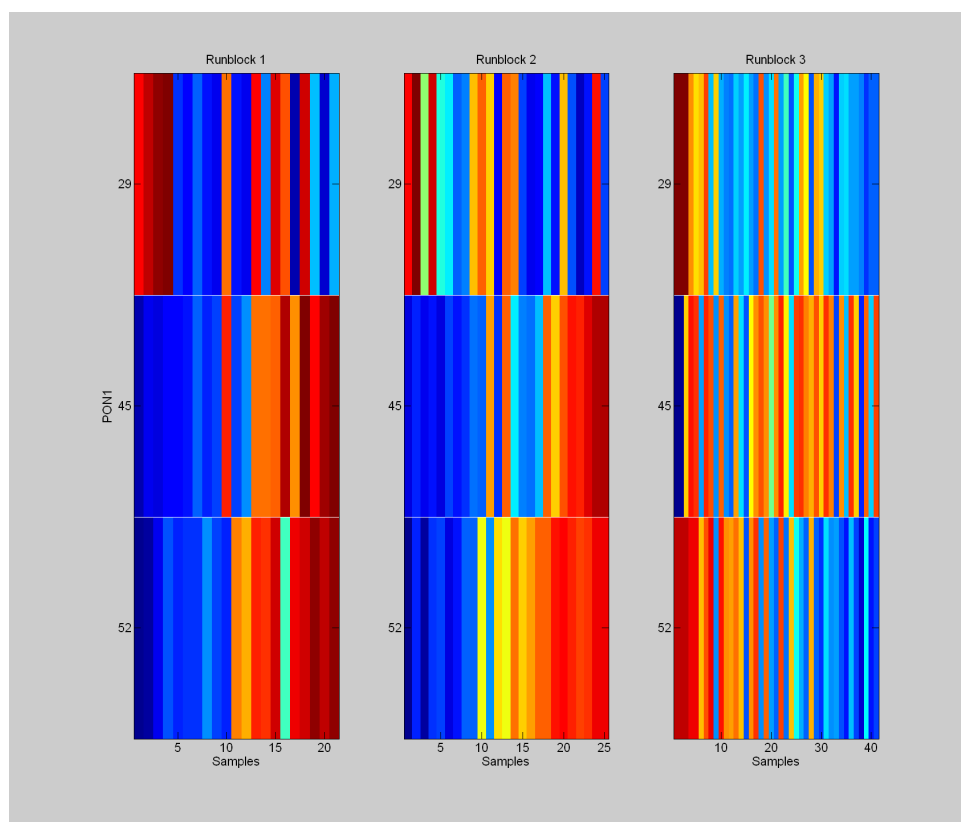

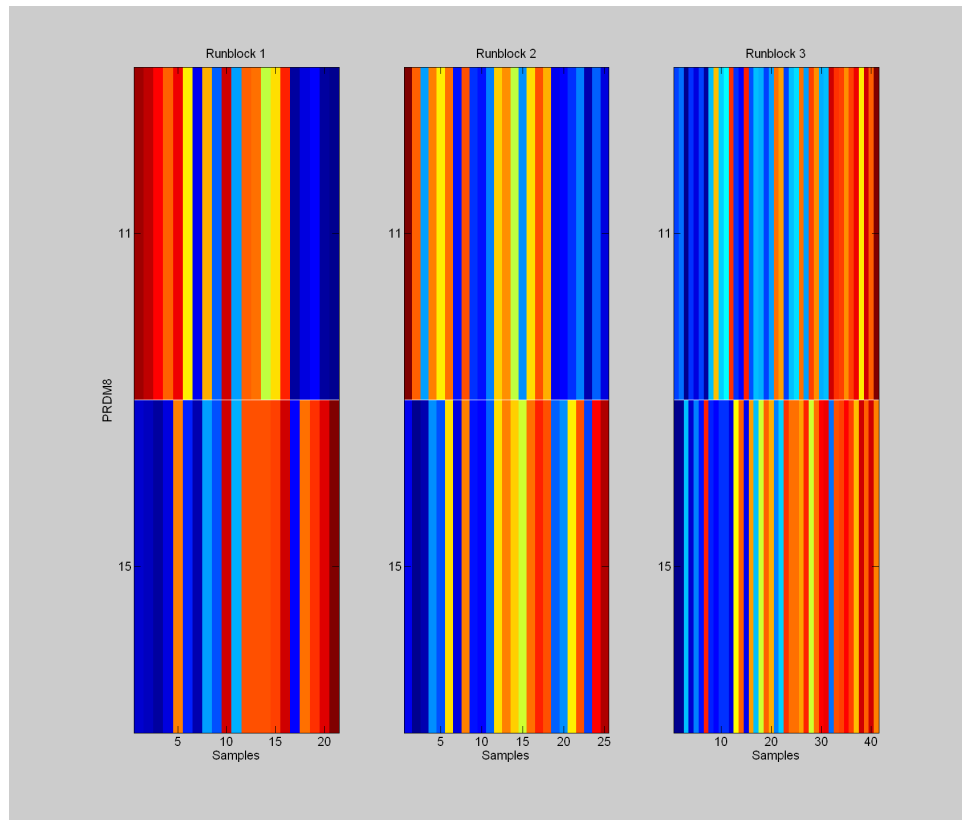

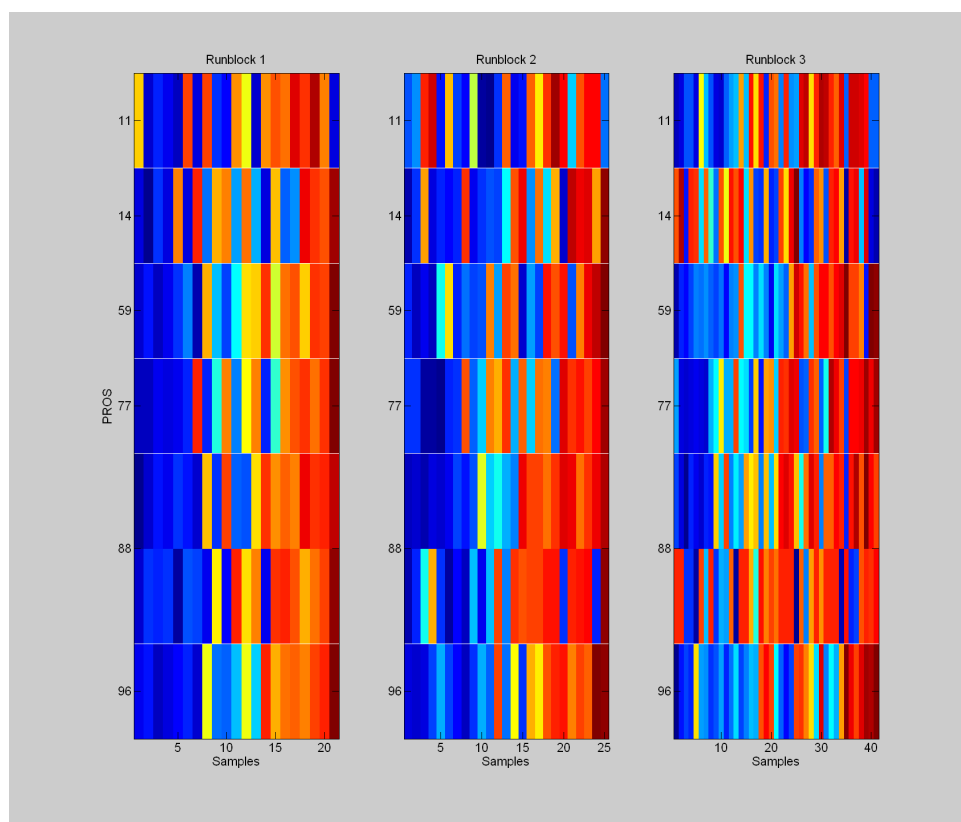

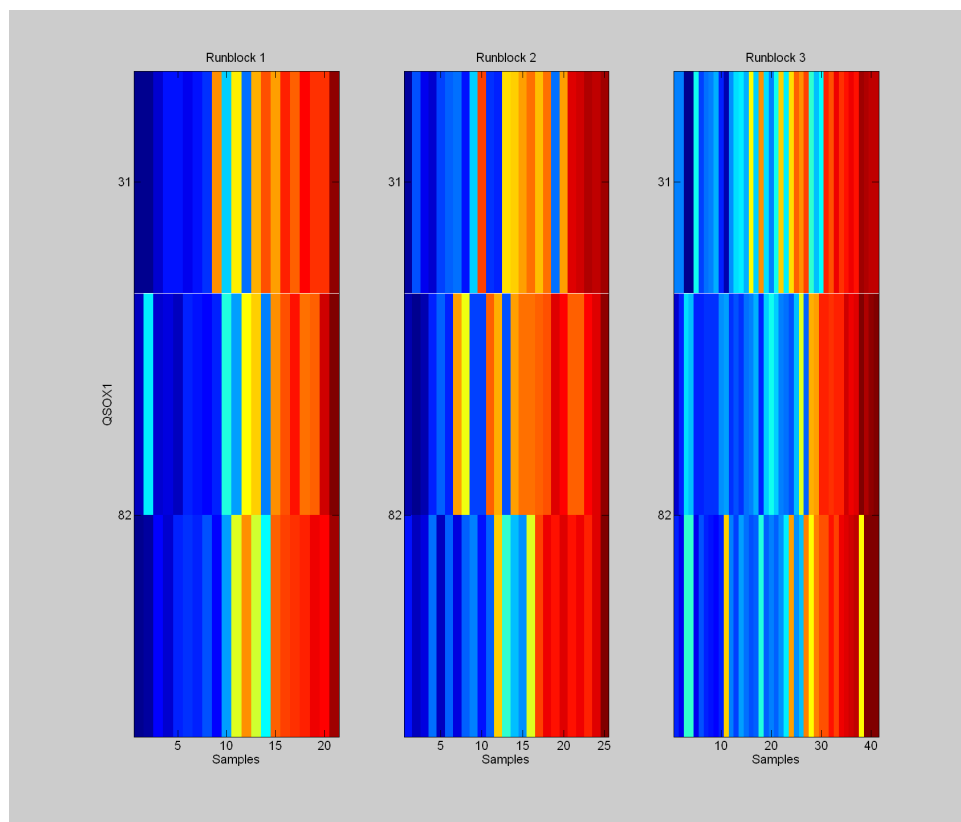

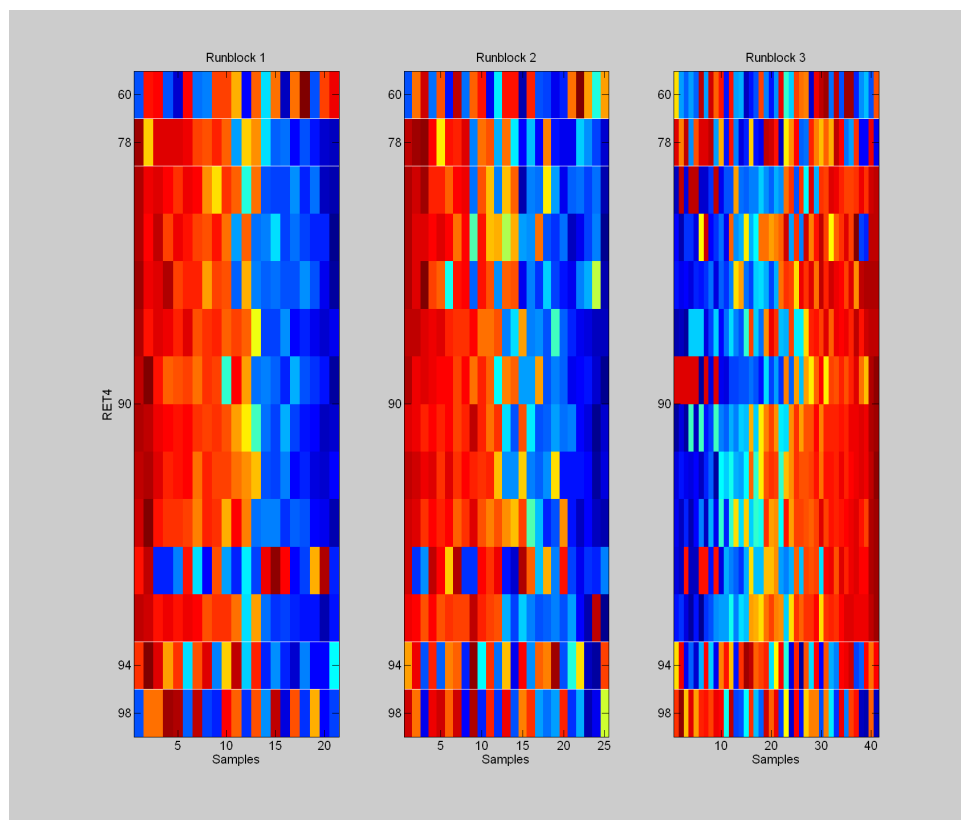

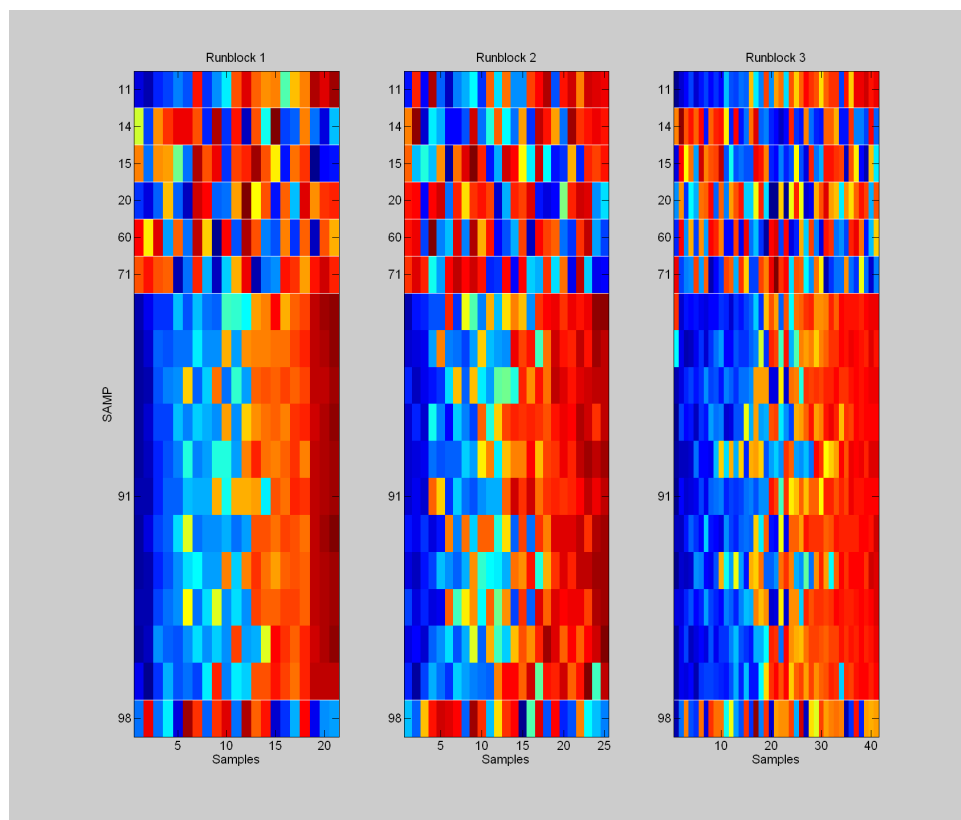

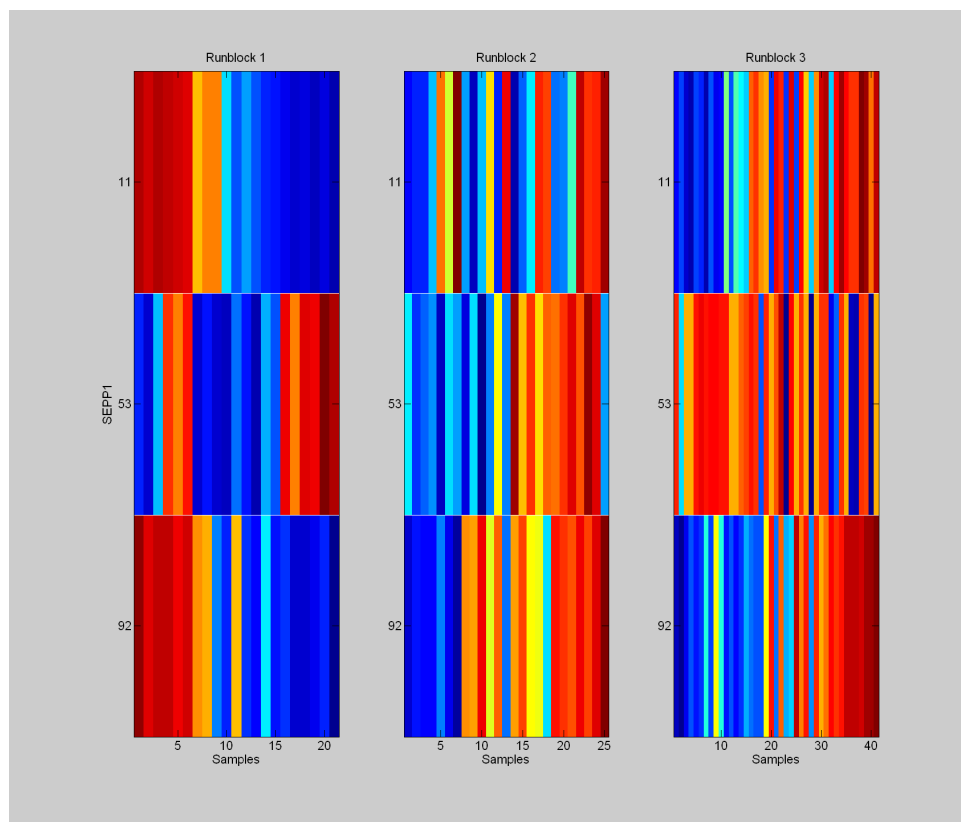

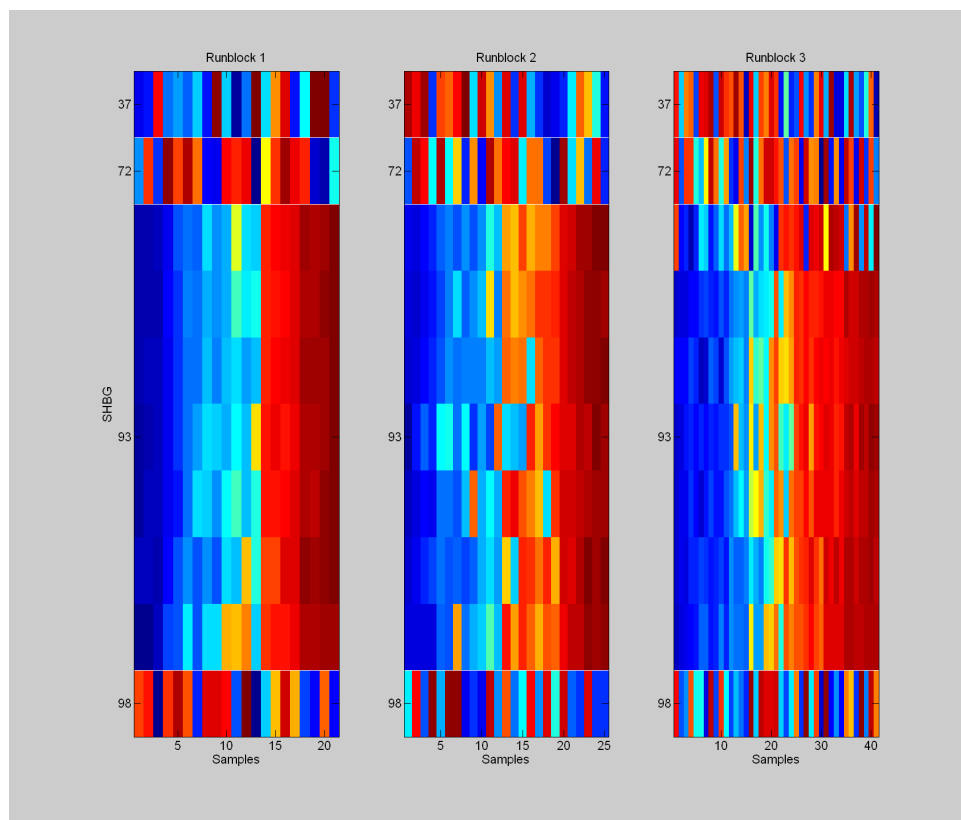

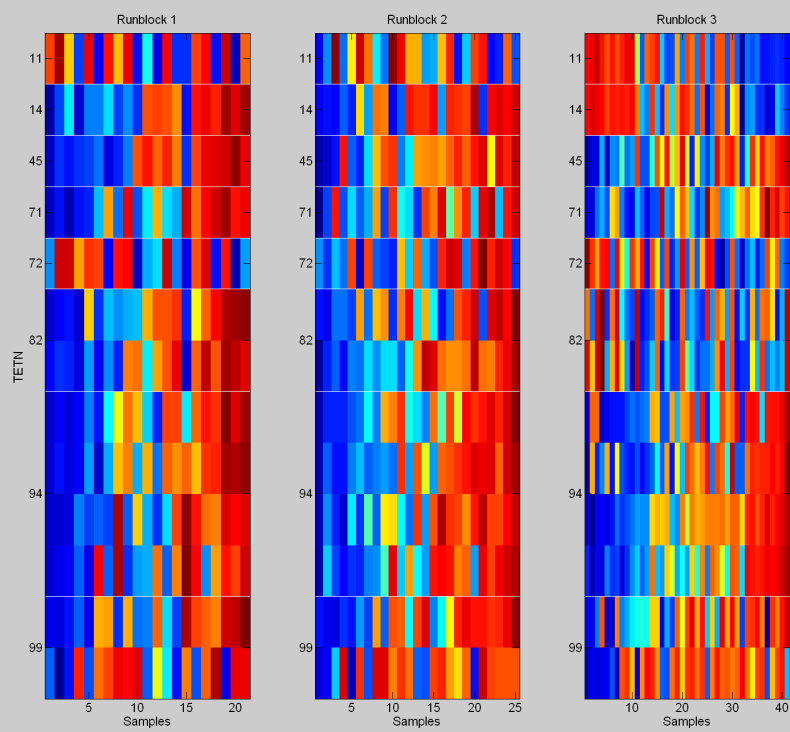

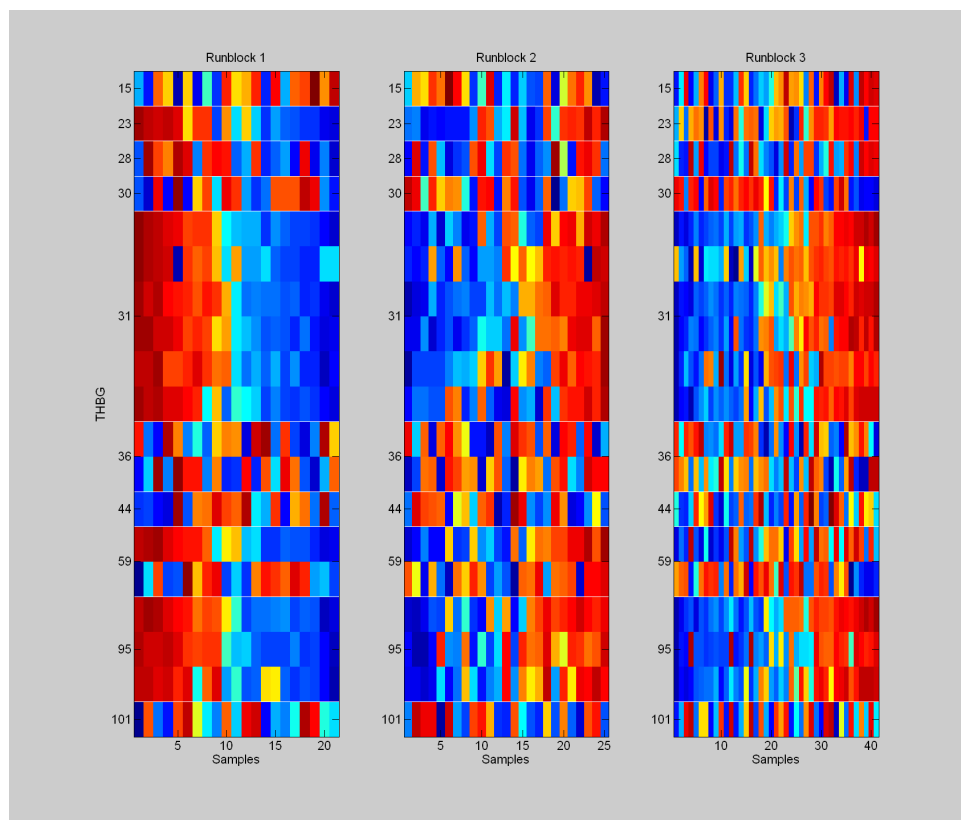

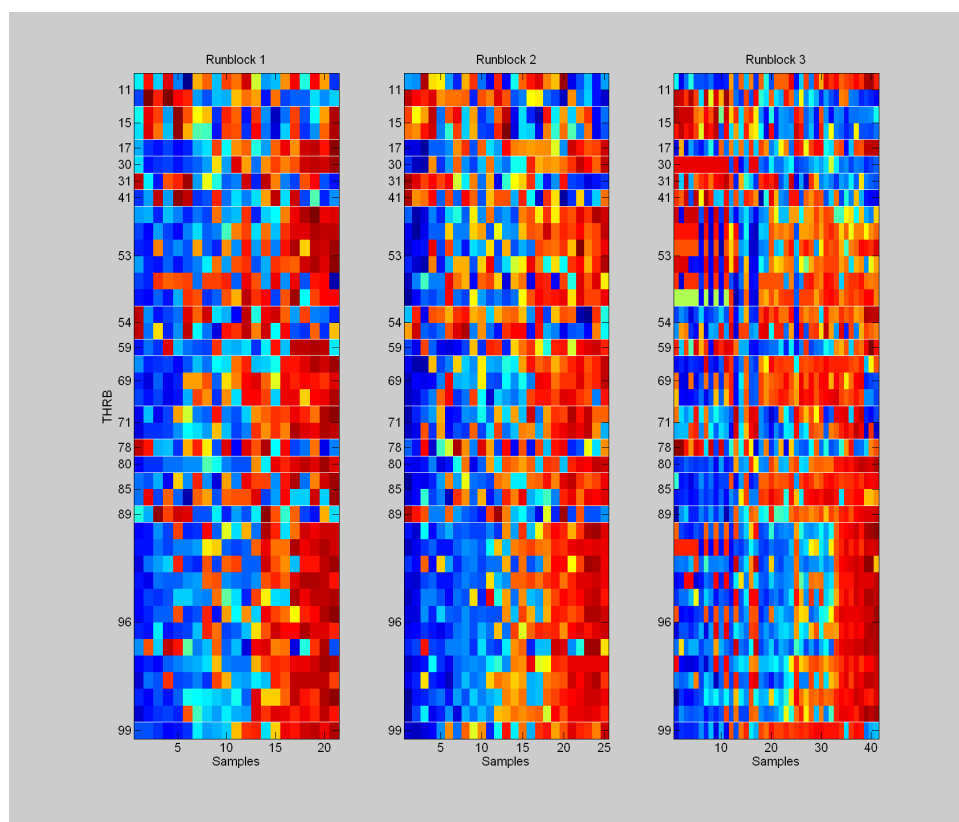

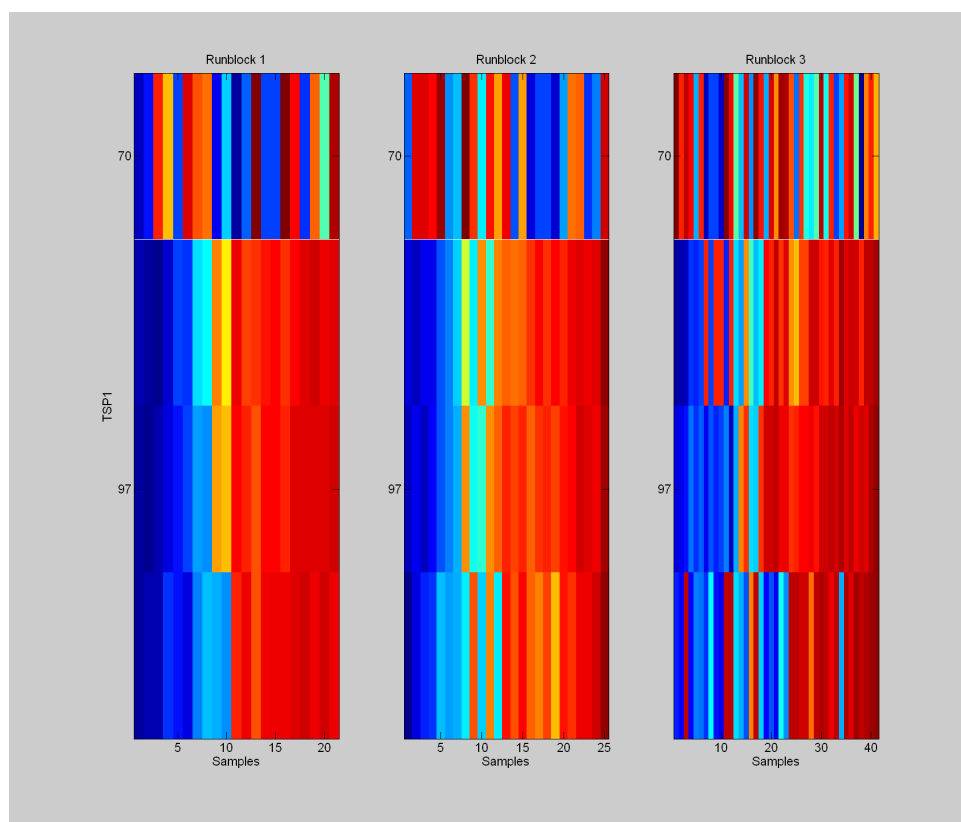

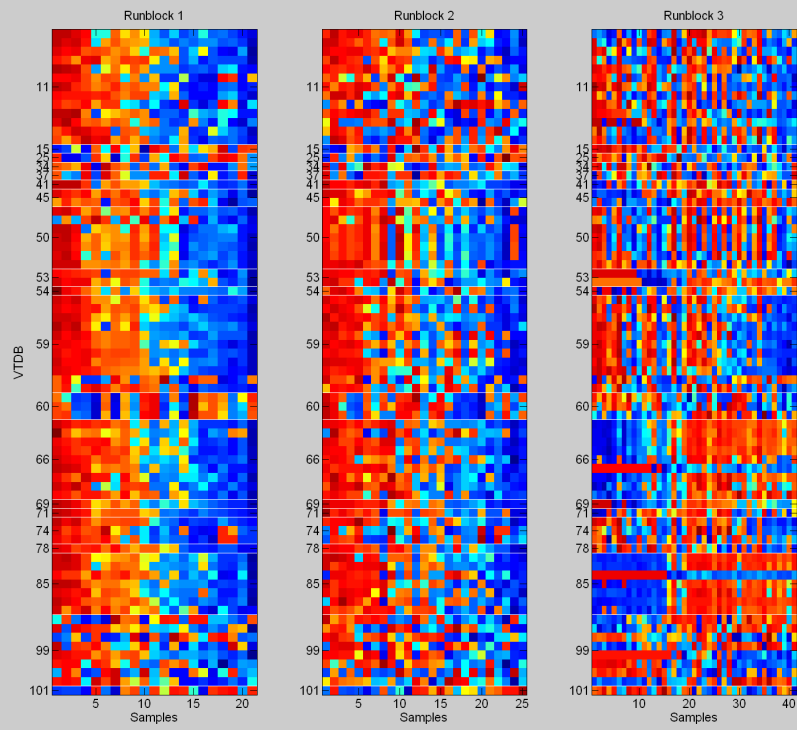

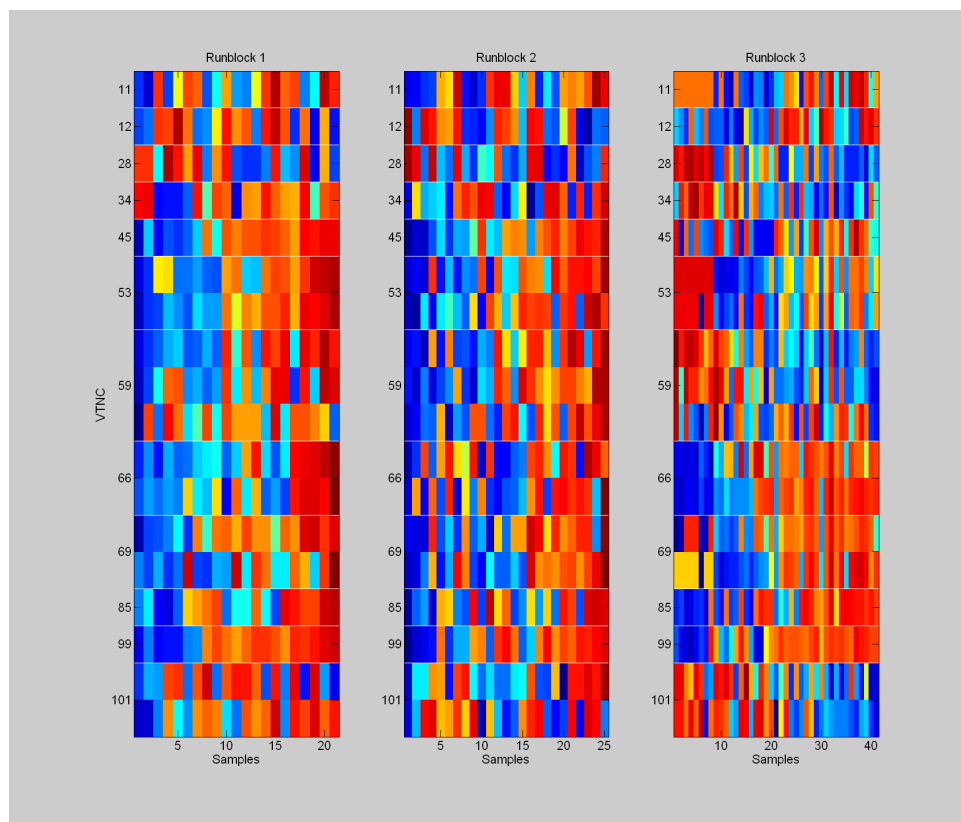

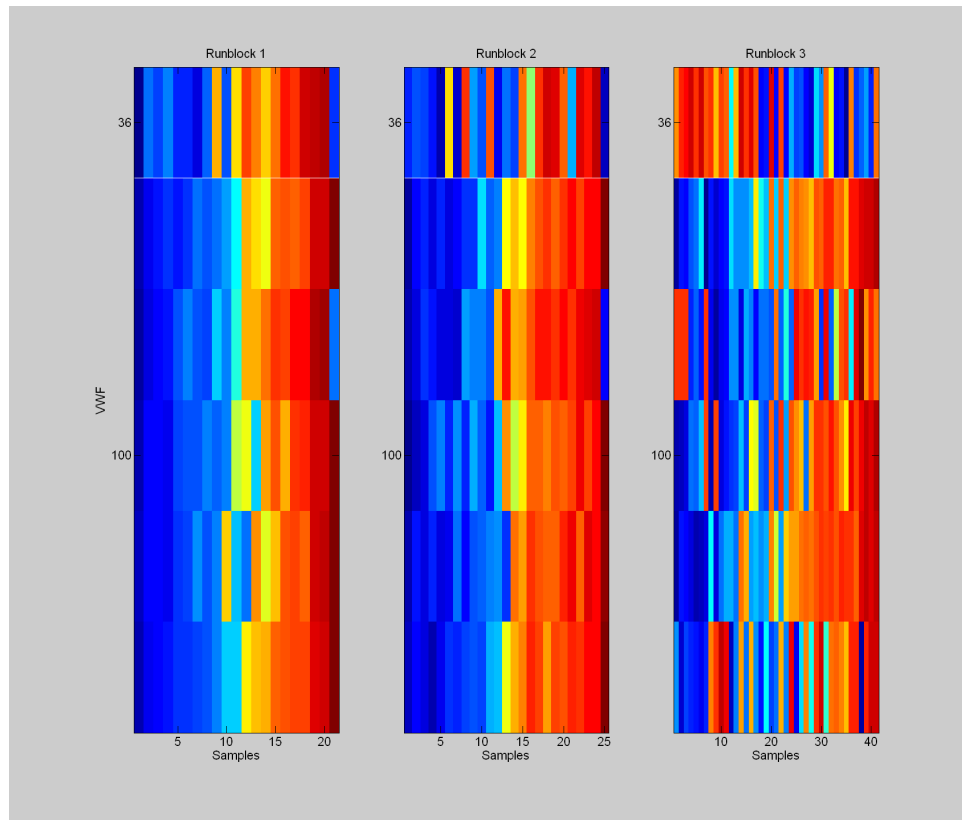

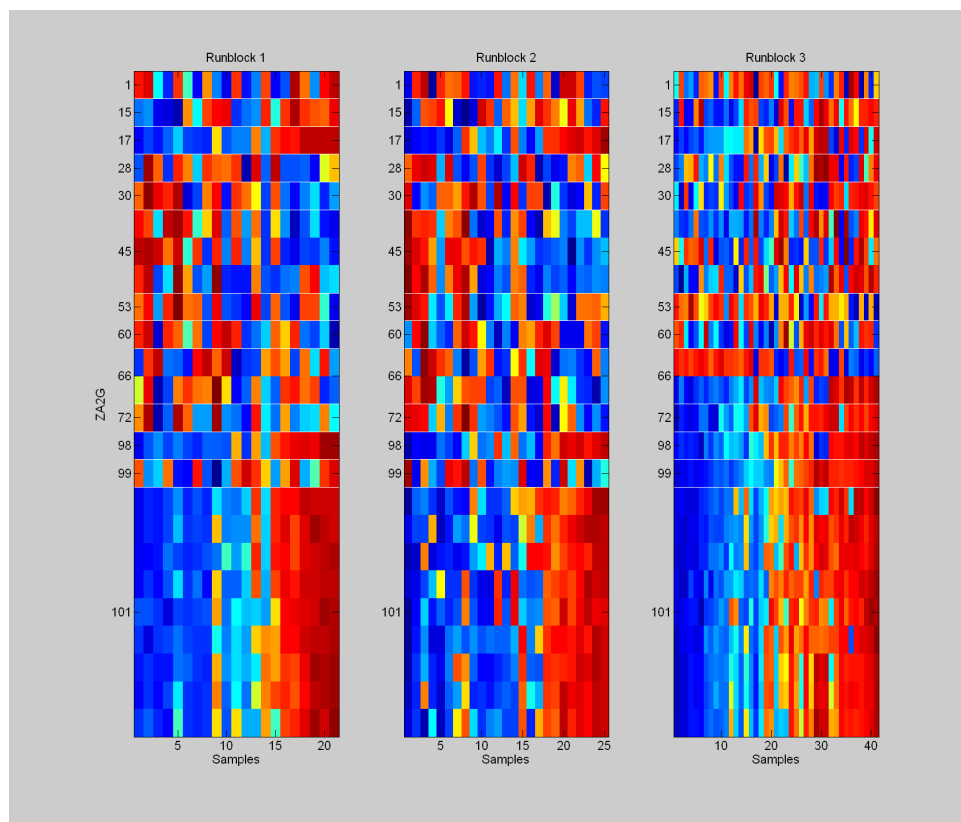

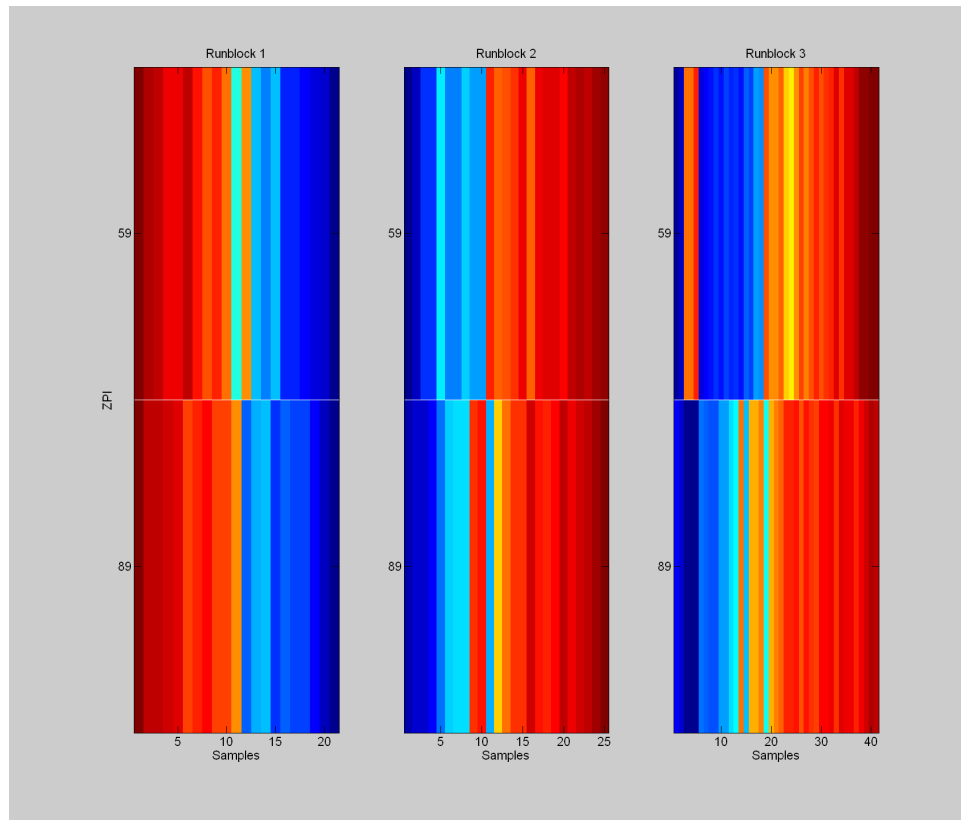

Supplement: Additional file 1 — Protein level grouping. Heatmaps for all identified isotope groups. The rows are sorted according to metaprotein membership and the columns are sorted so that the first principal component is increasing. Each isotope group is associated with a single metaprotein. Those metaproteins are labeled on the y-axis. [file 1471-2105-13-74-S1.pdf]

## **Supplemental Information 2**

E-mail:

---

\*To whom correspondence should be addressed

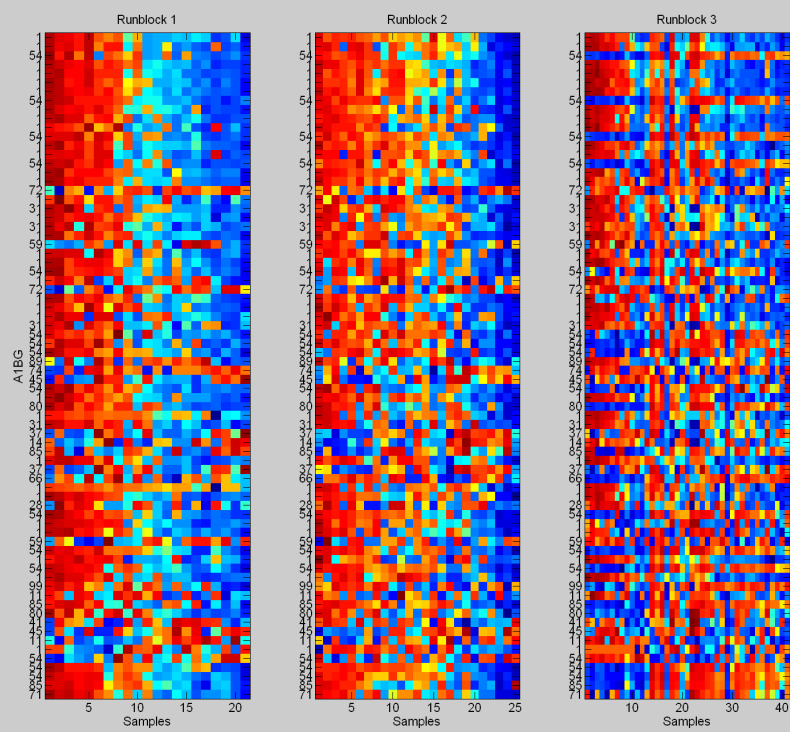

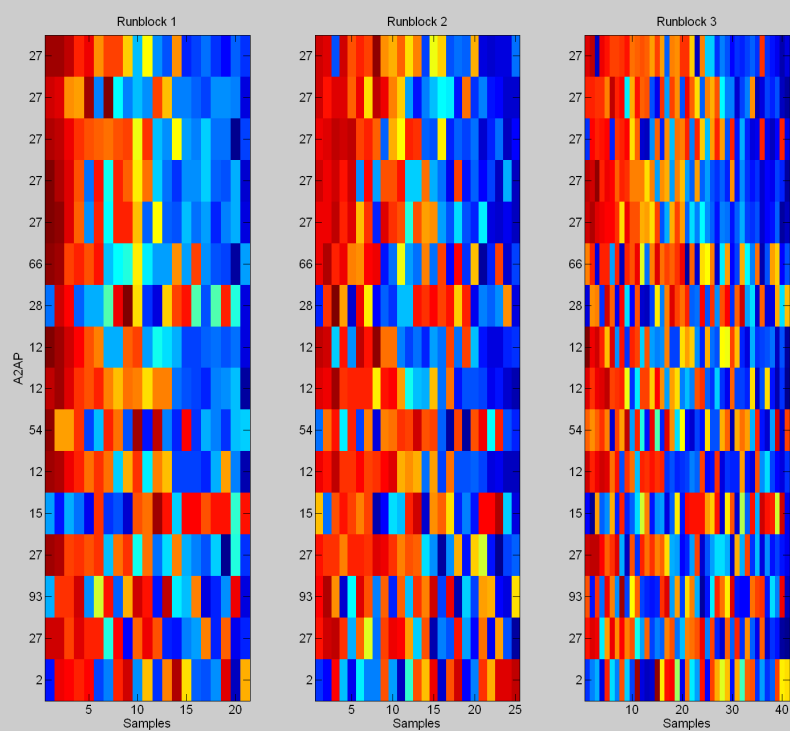

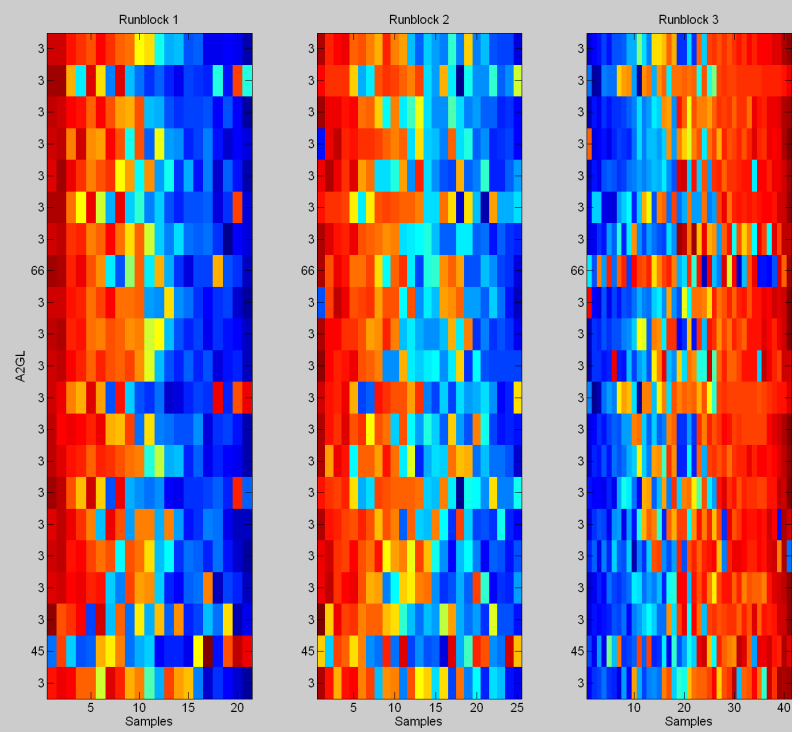

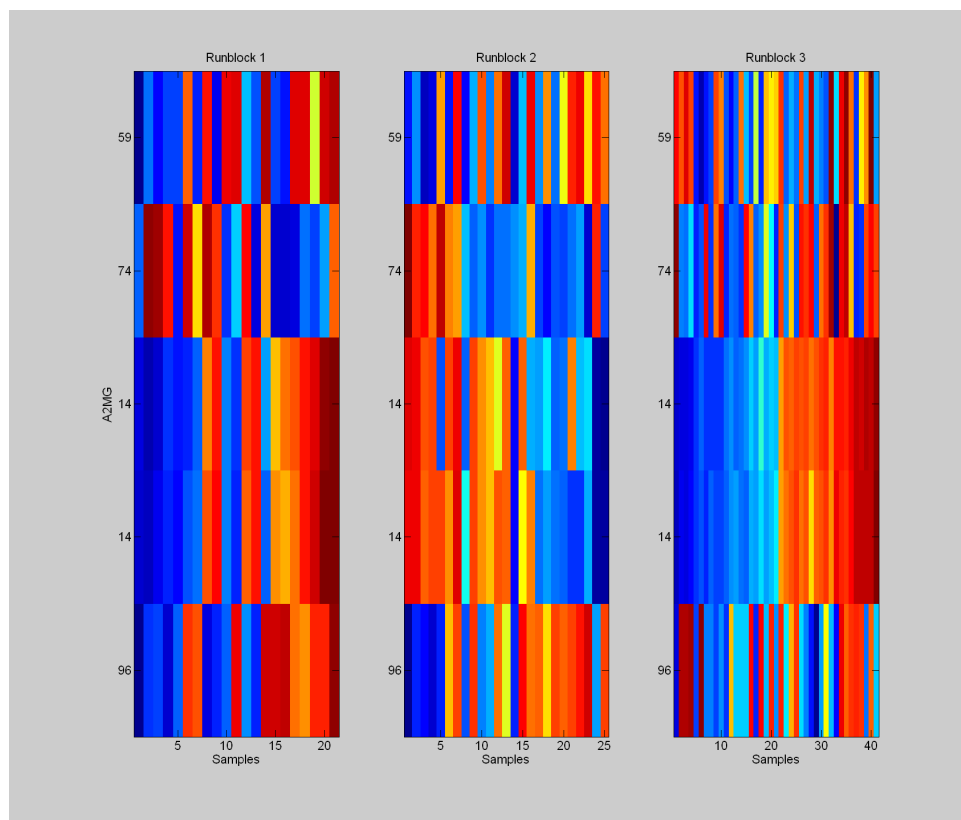

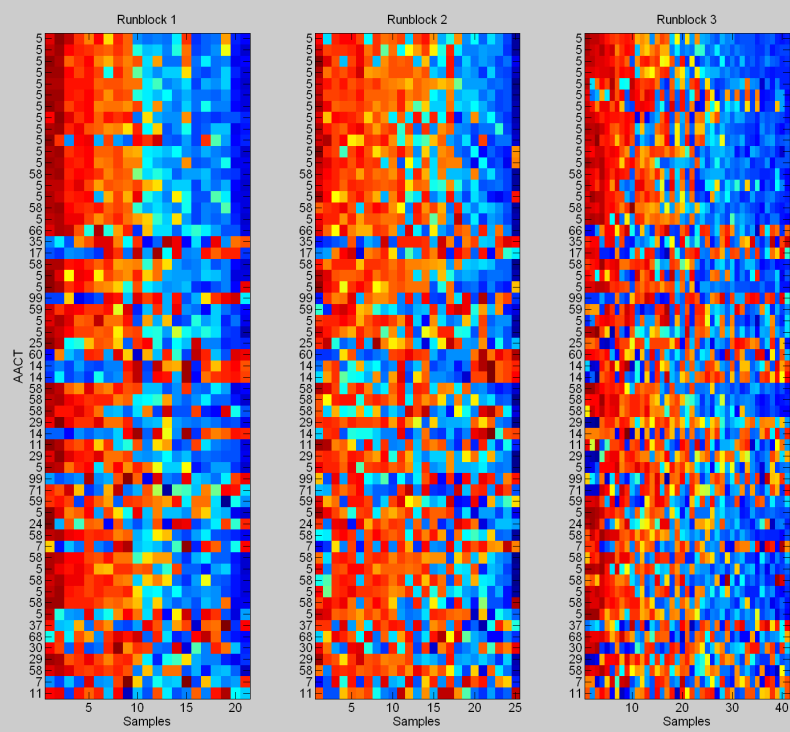

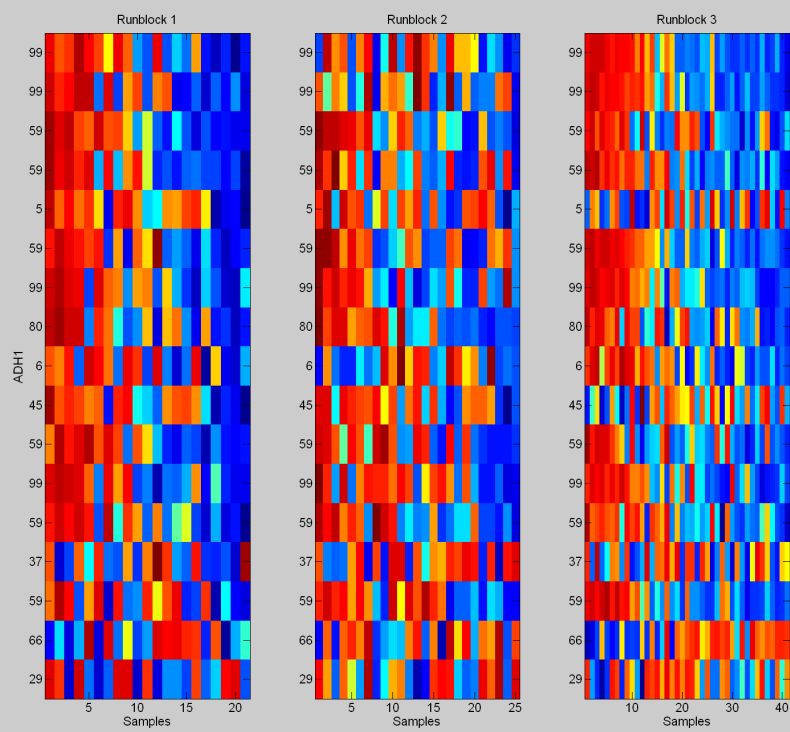

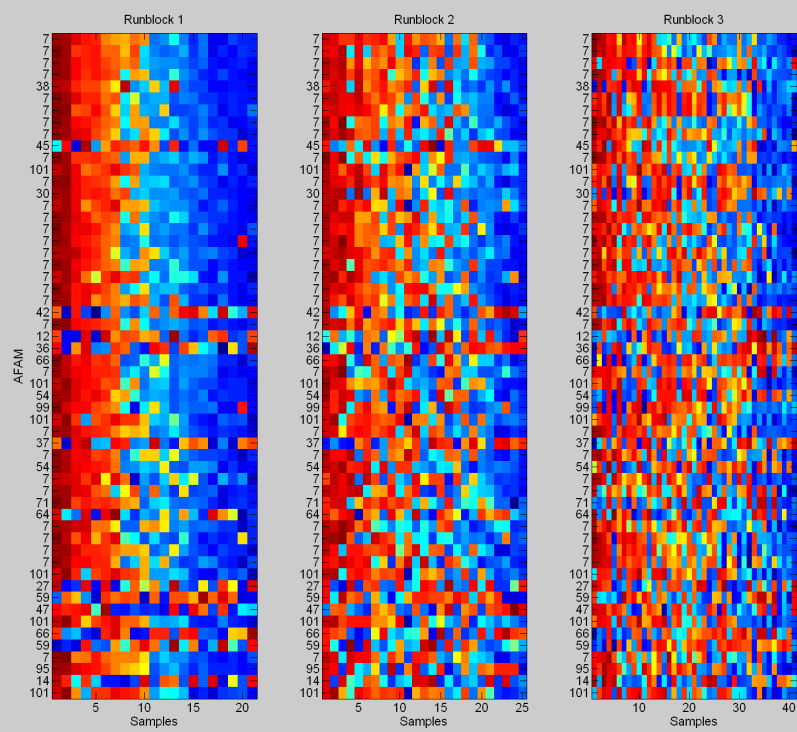

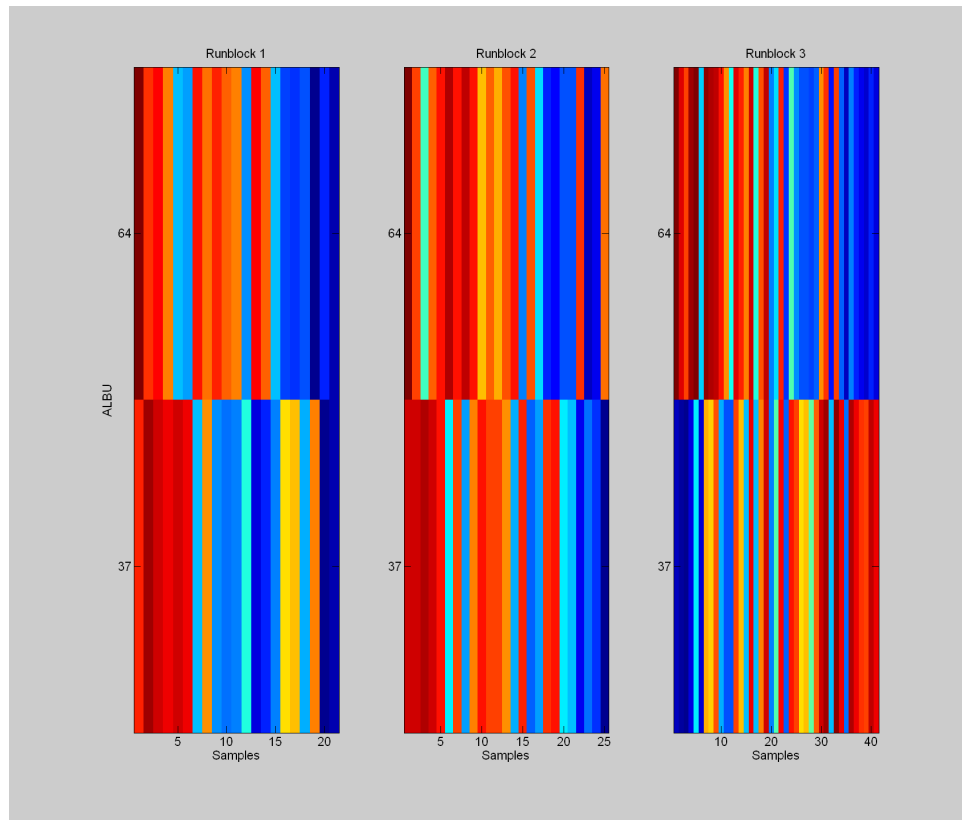

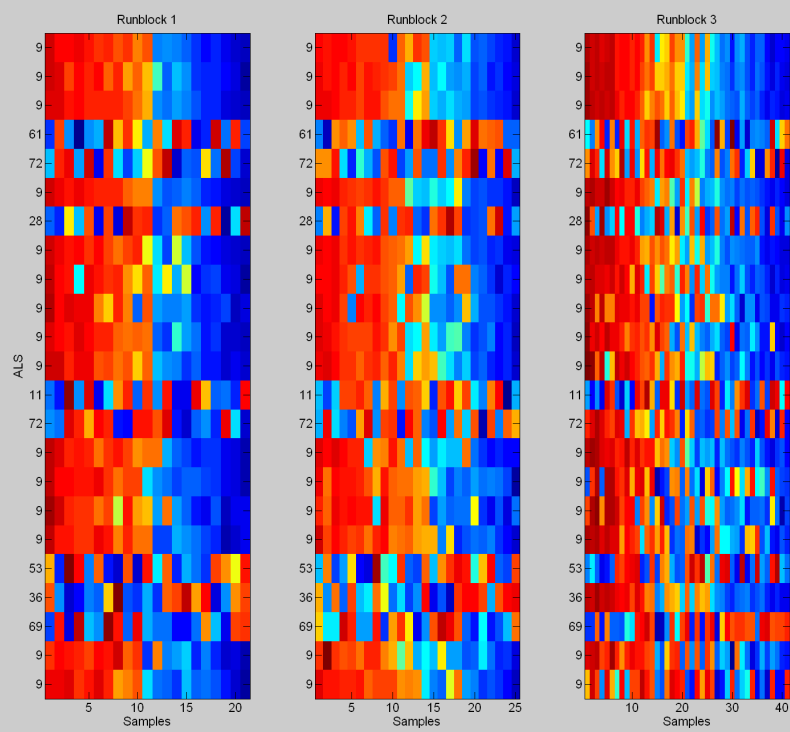

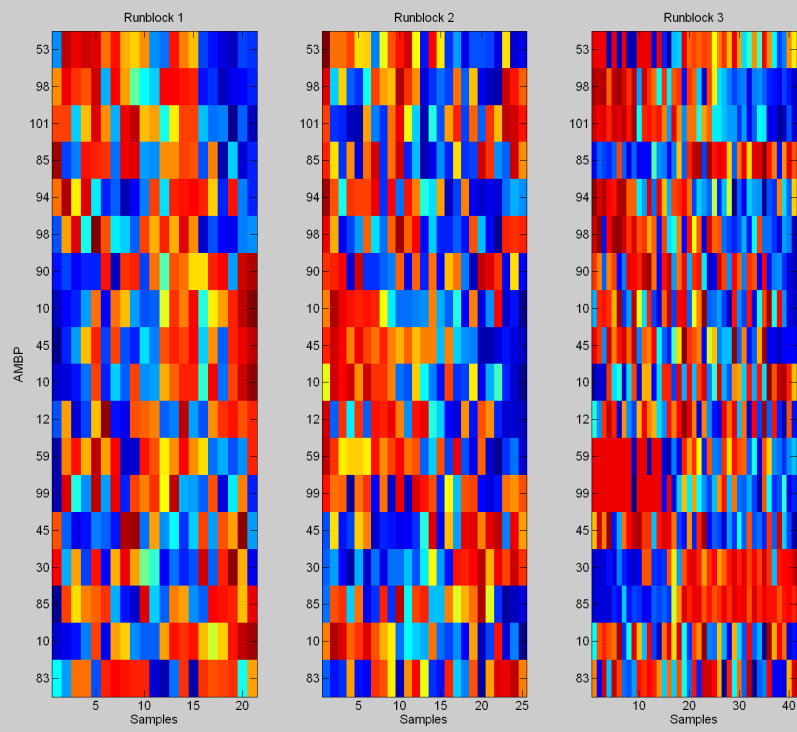

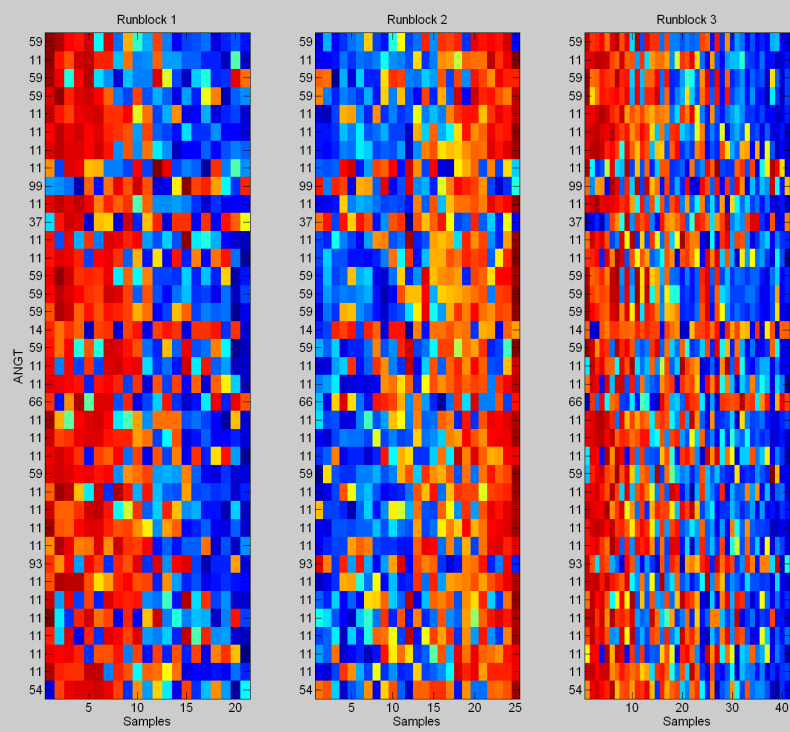

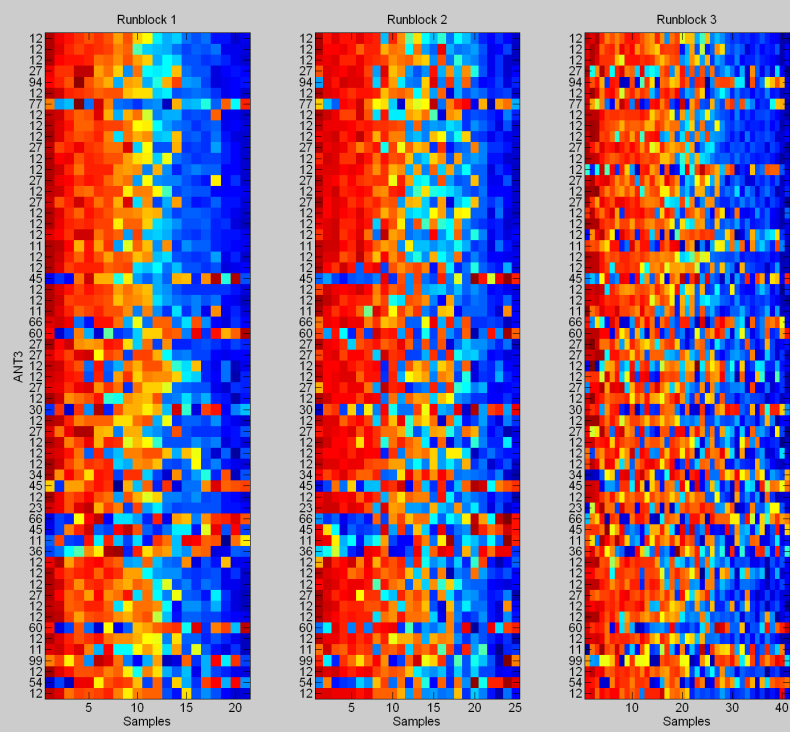

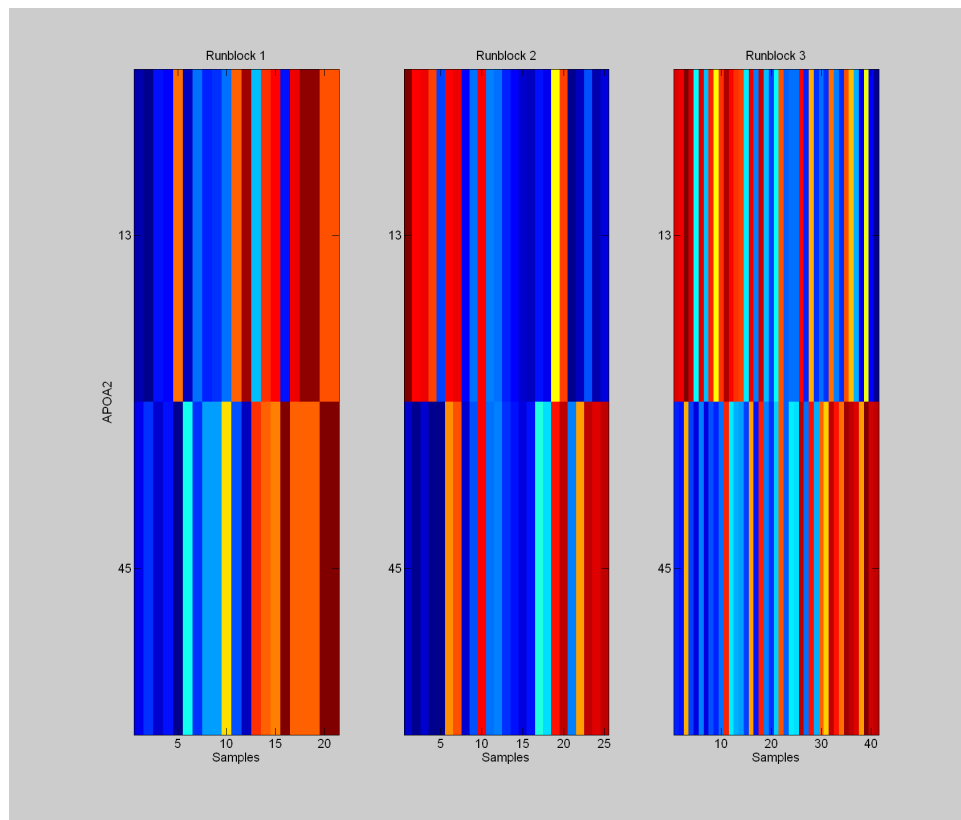

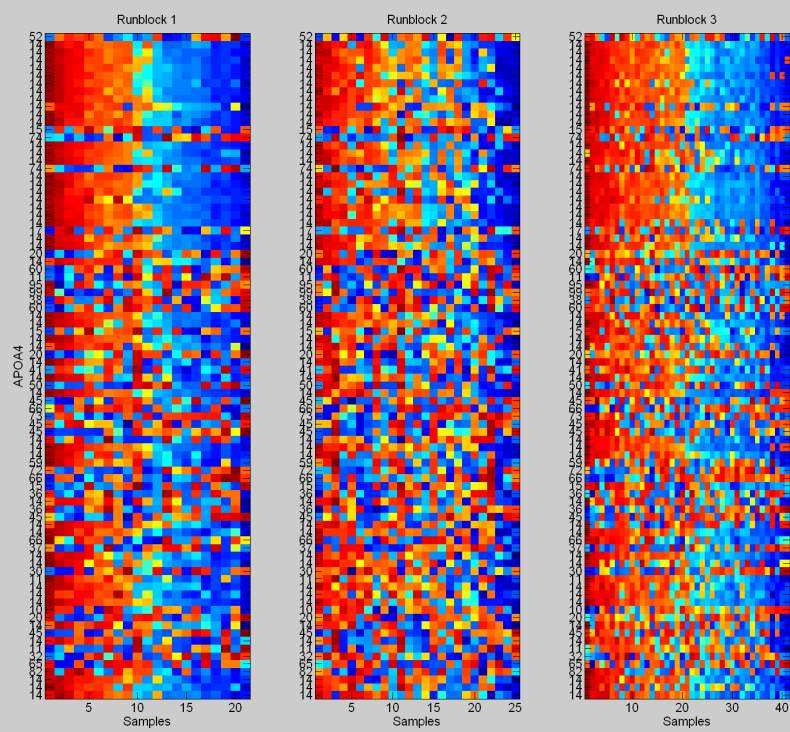

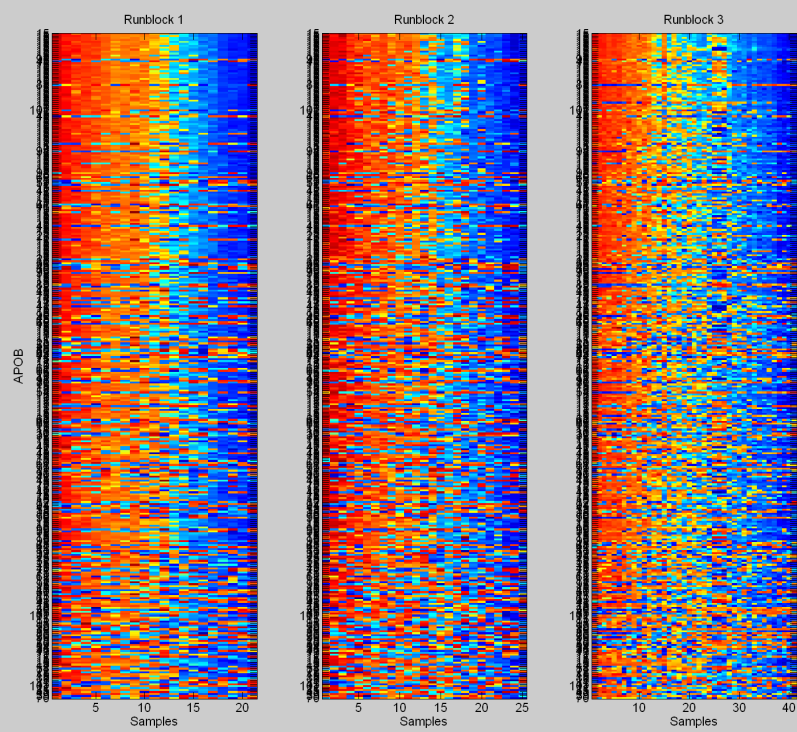

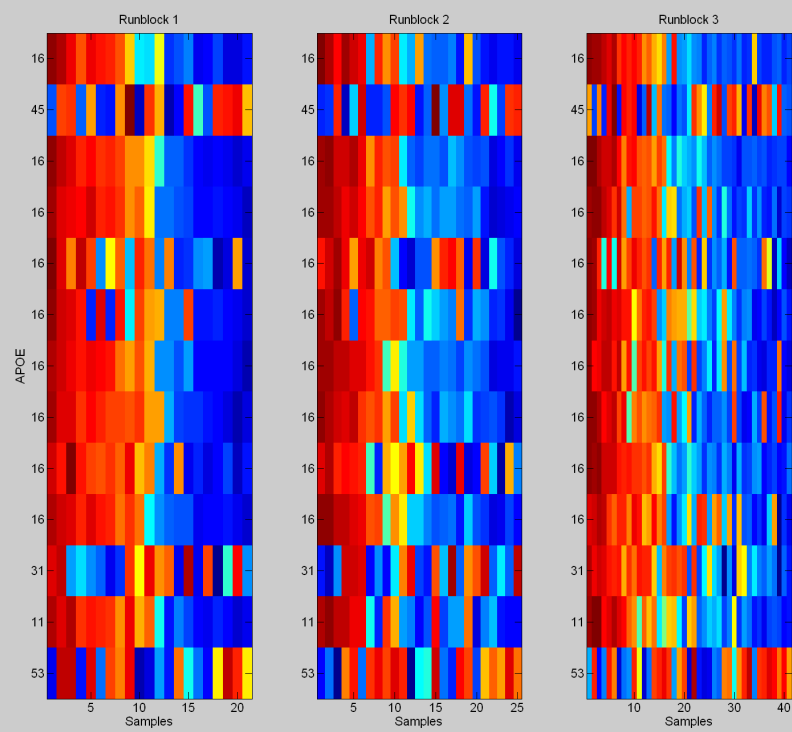

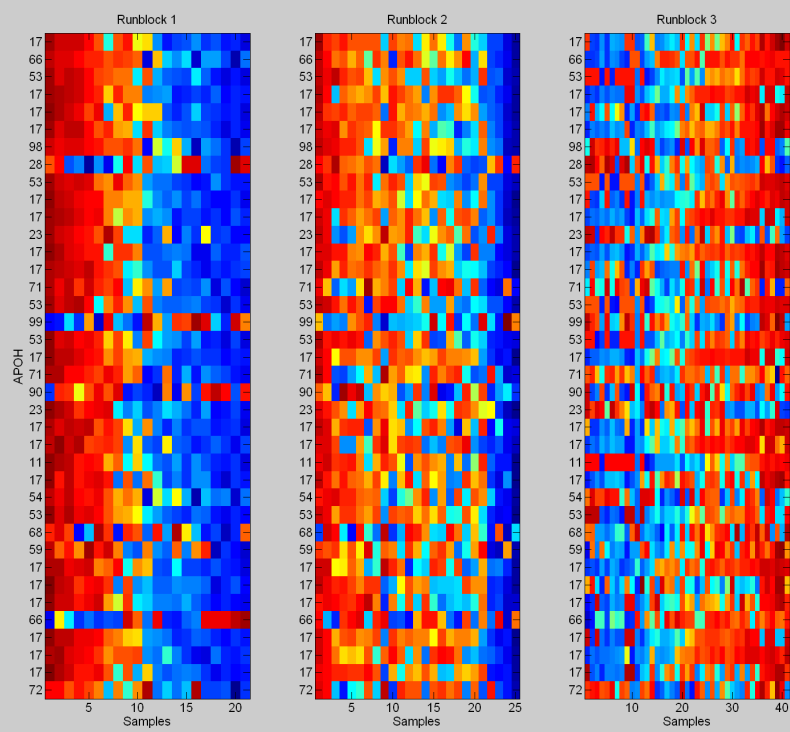

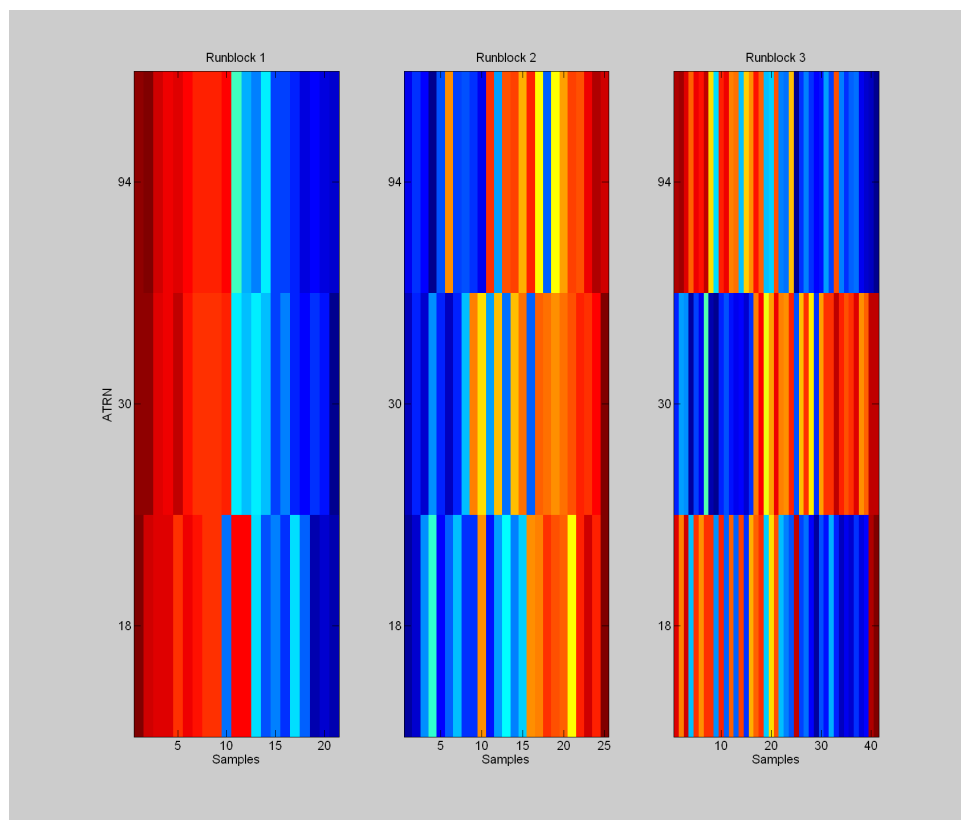

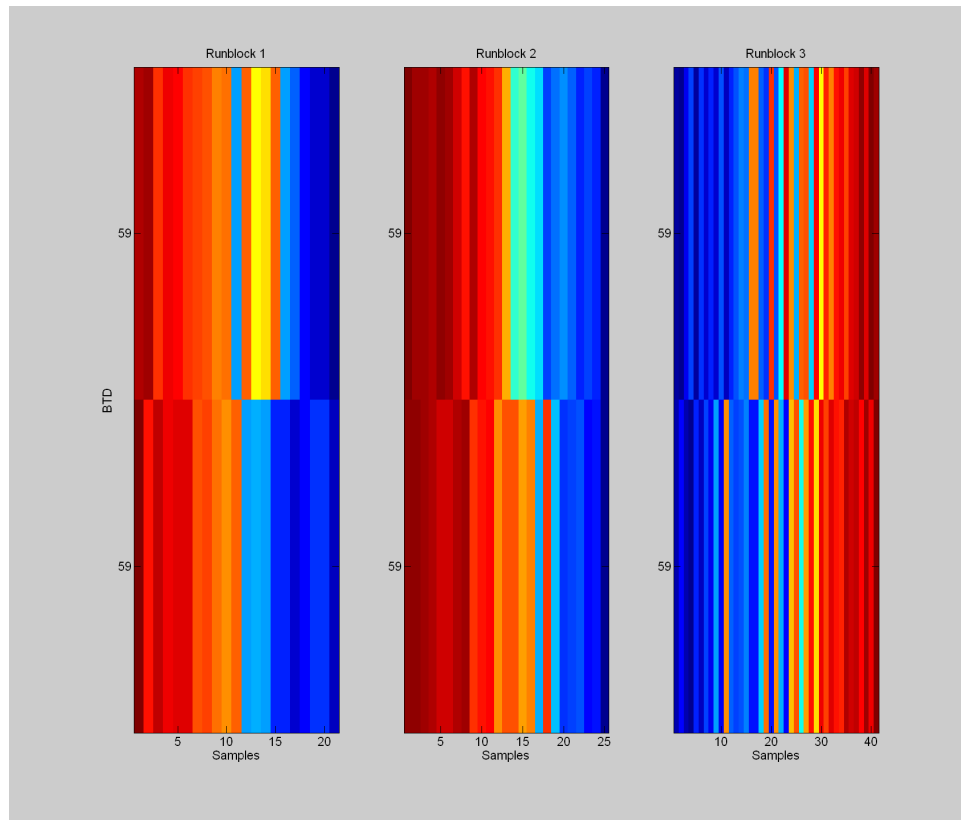

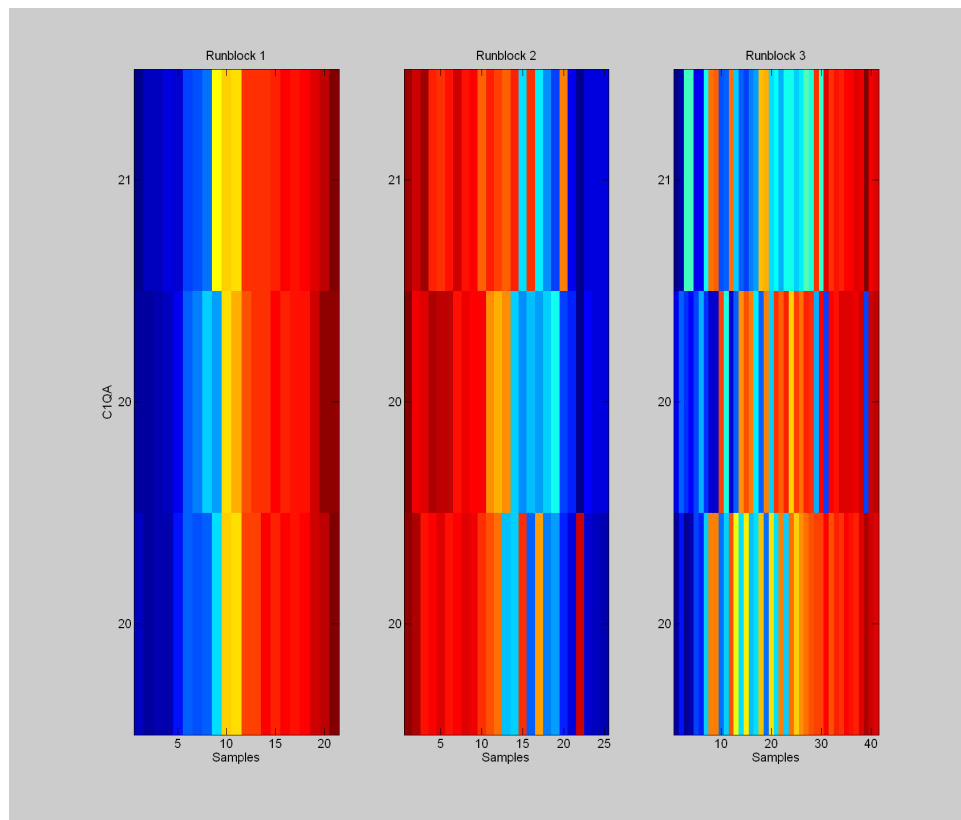

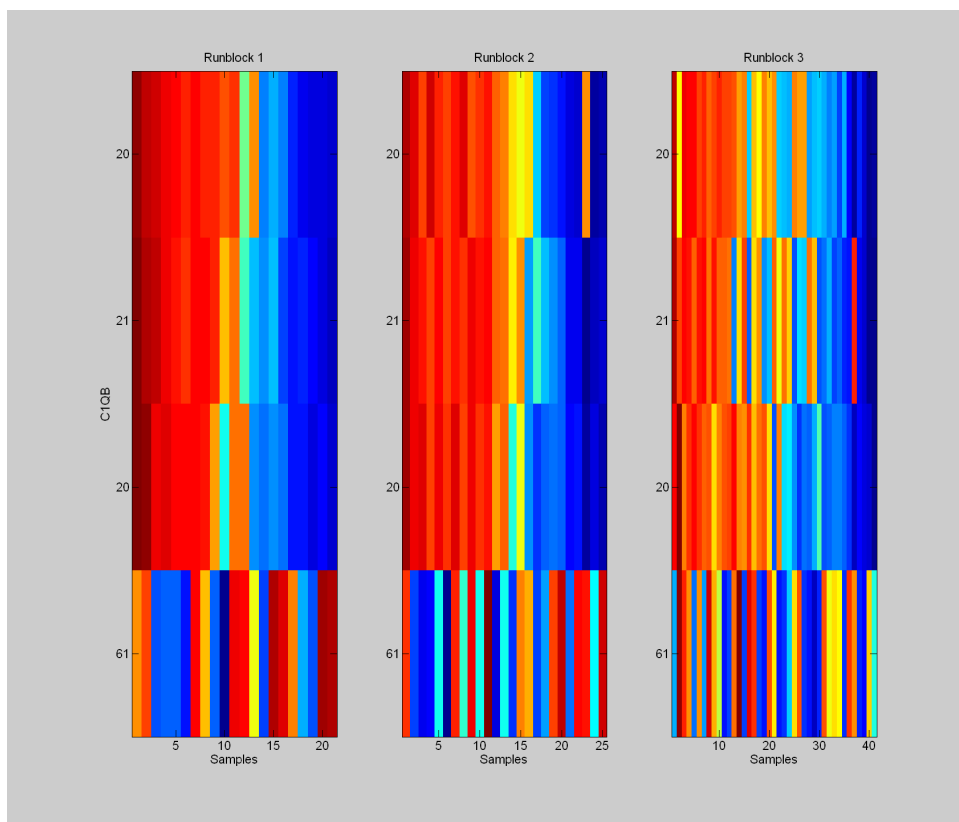

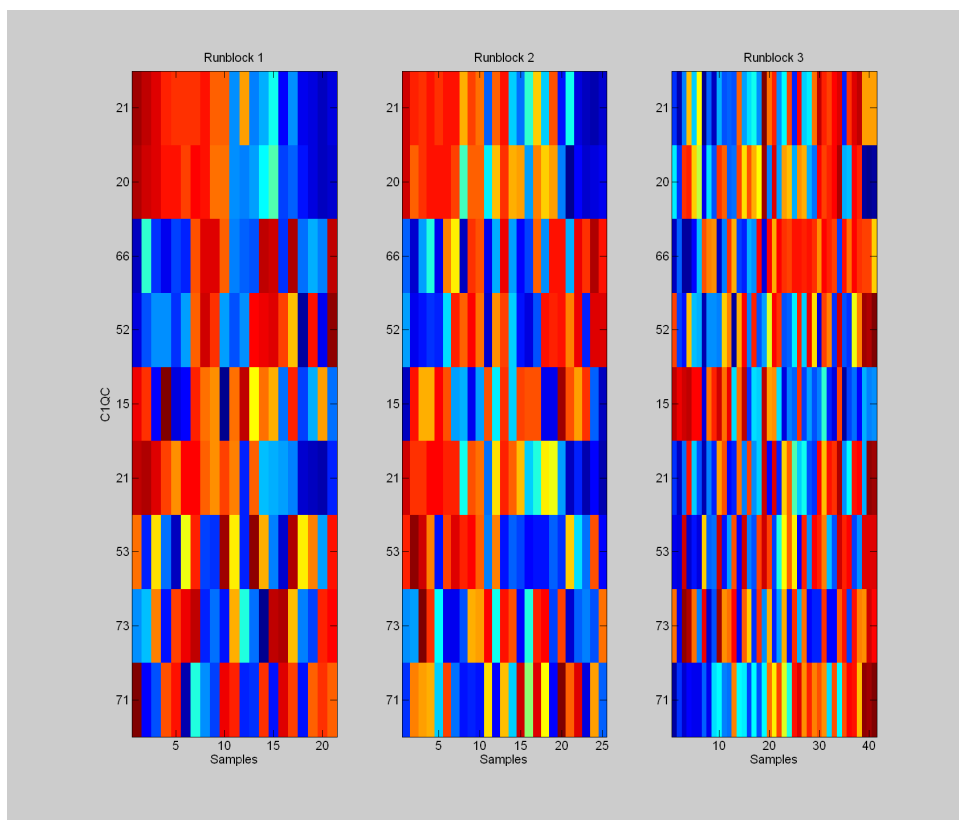

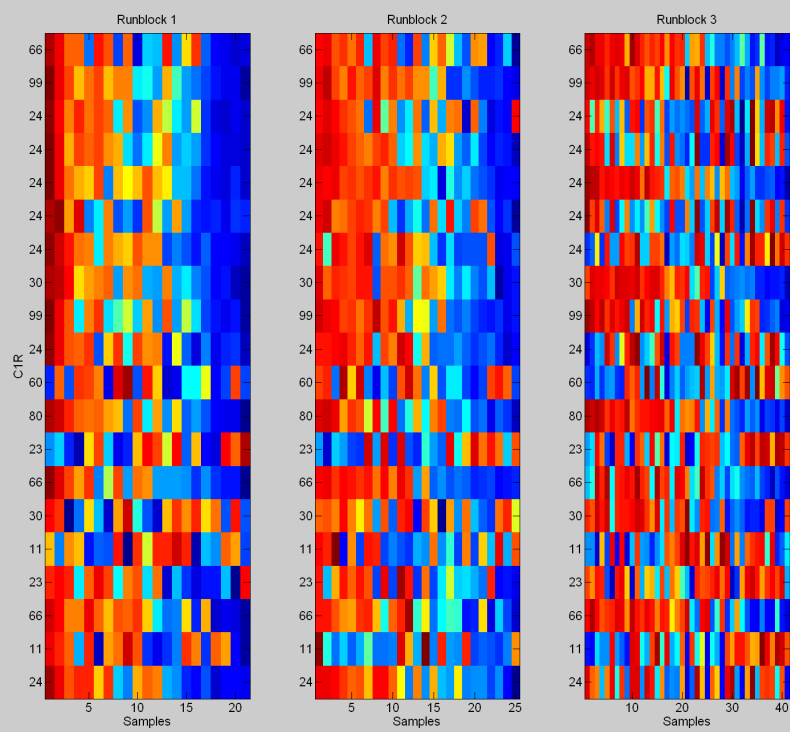

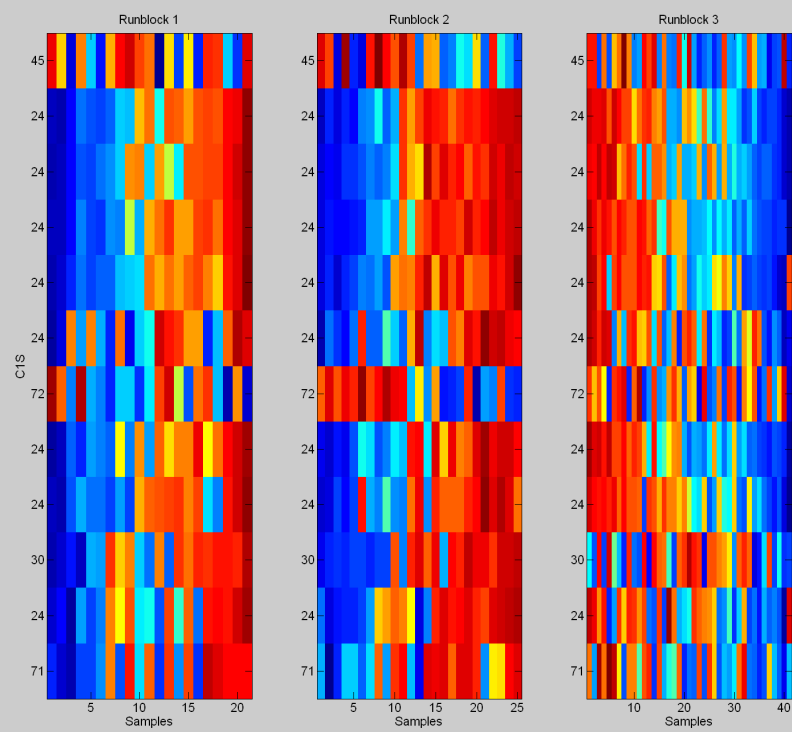

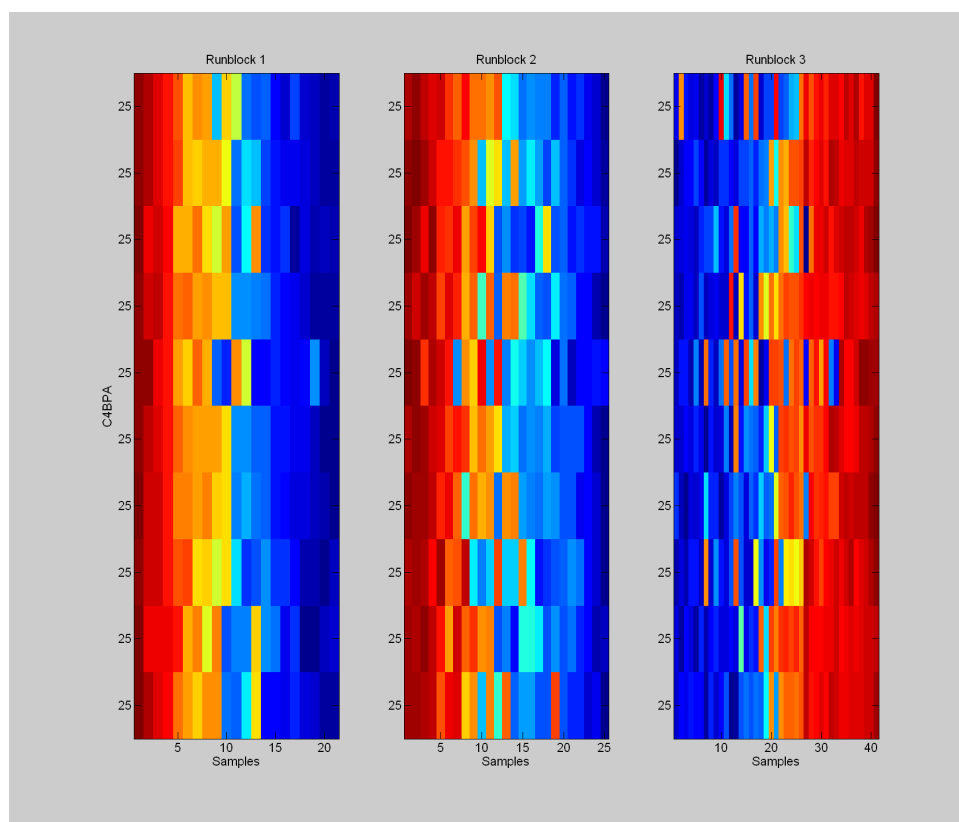

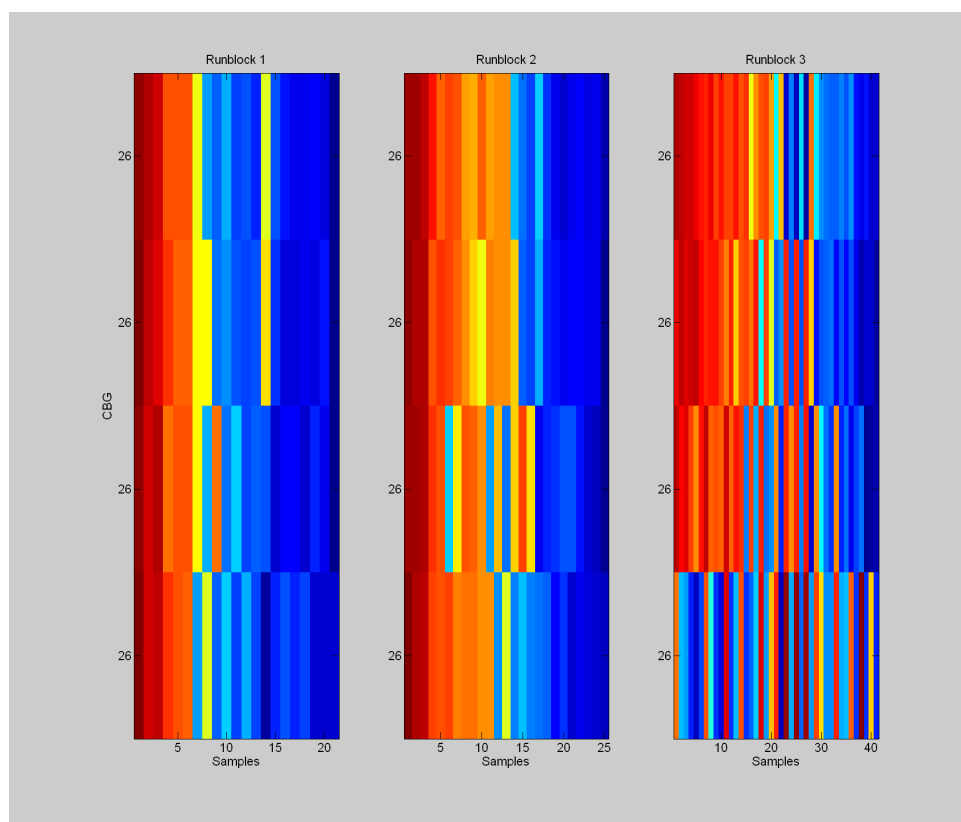

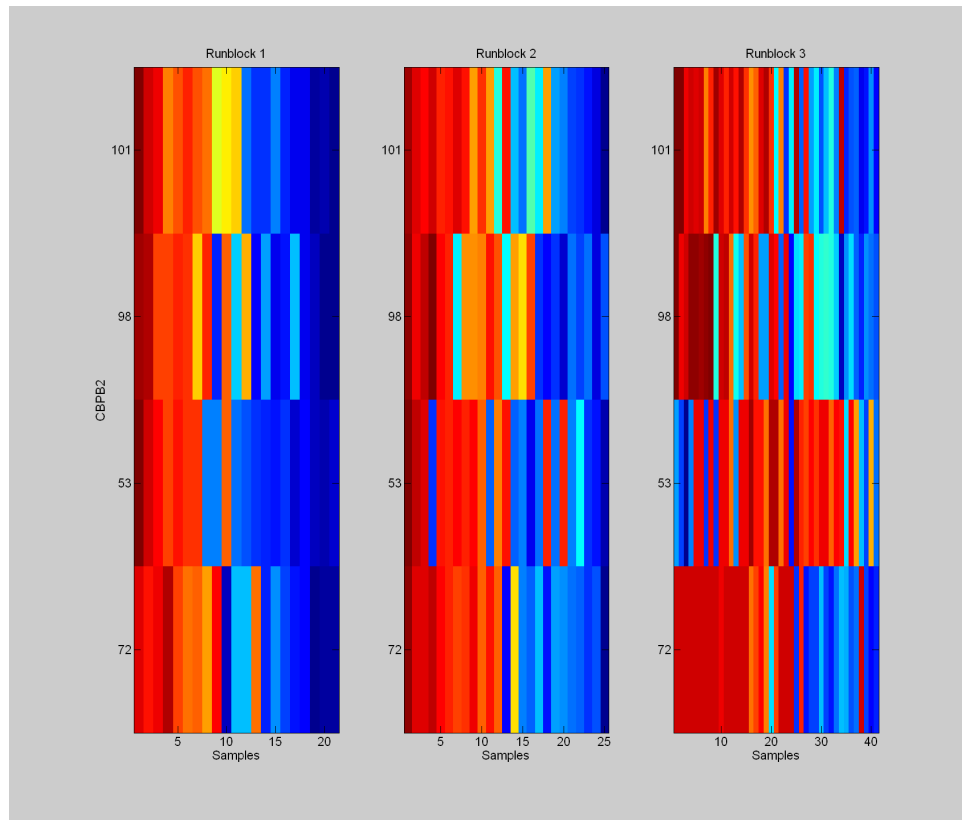

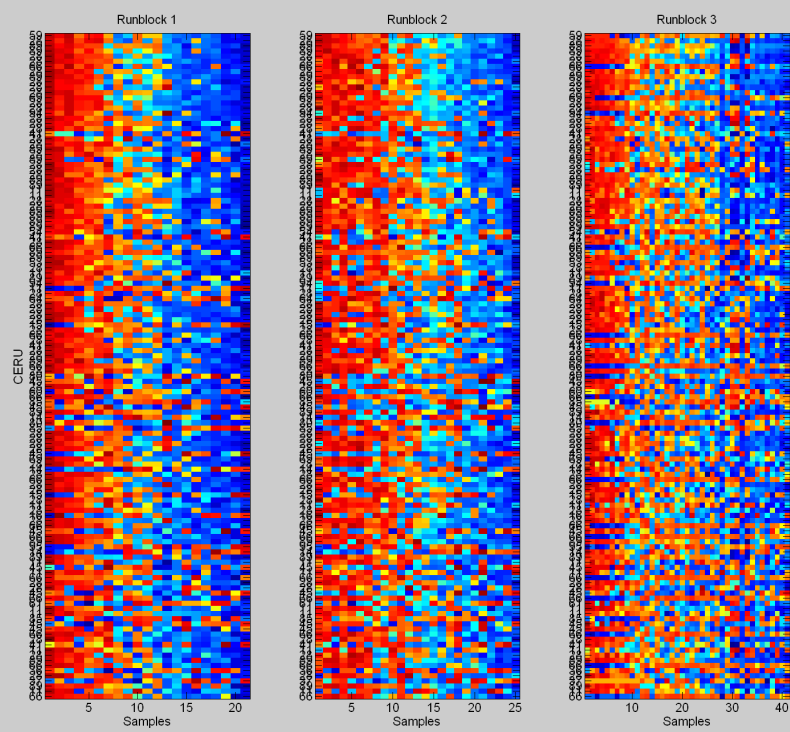

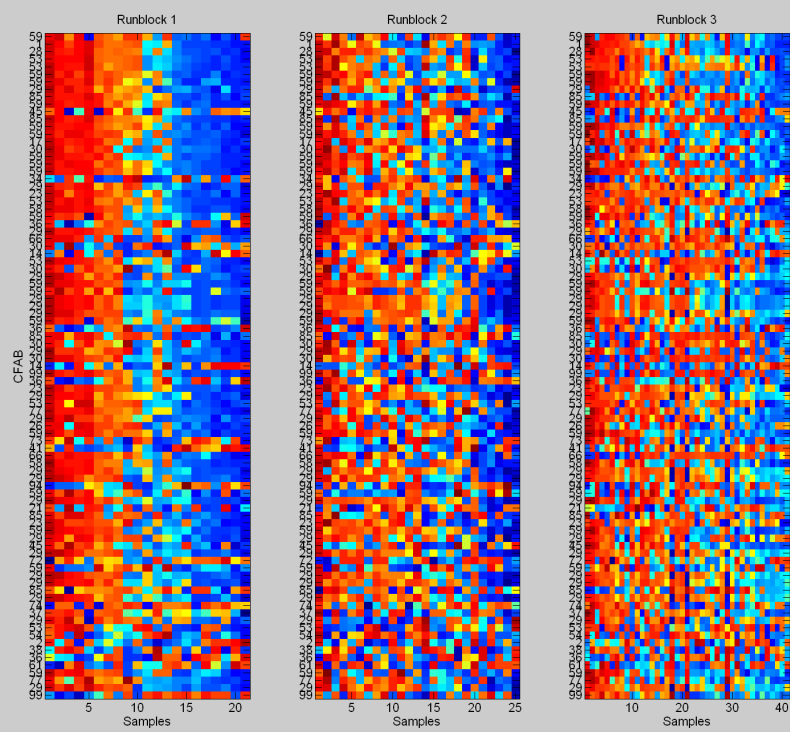

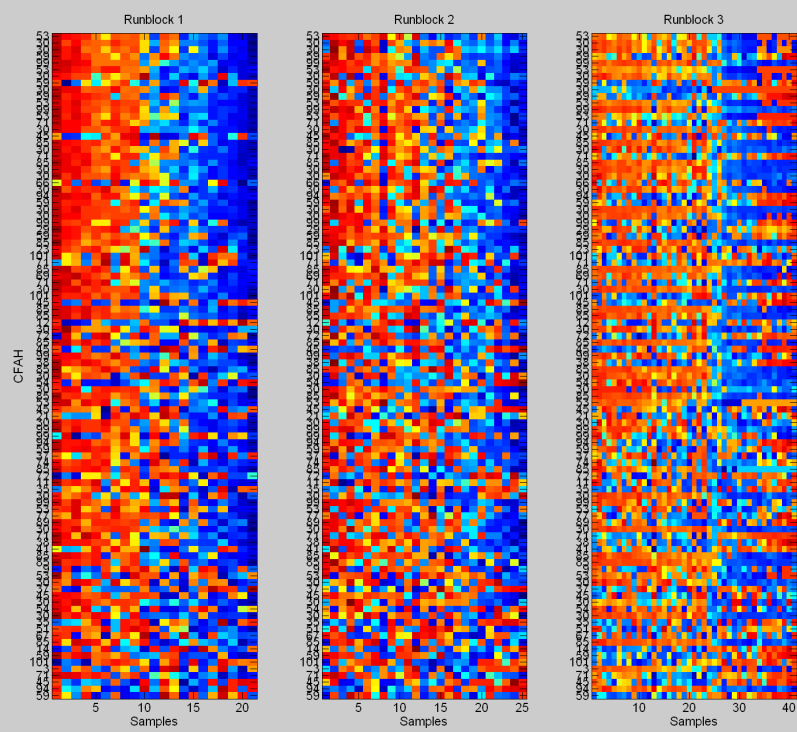

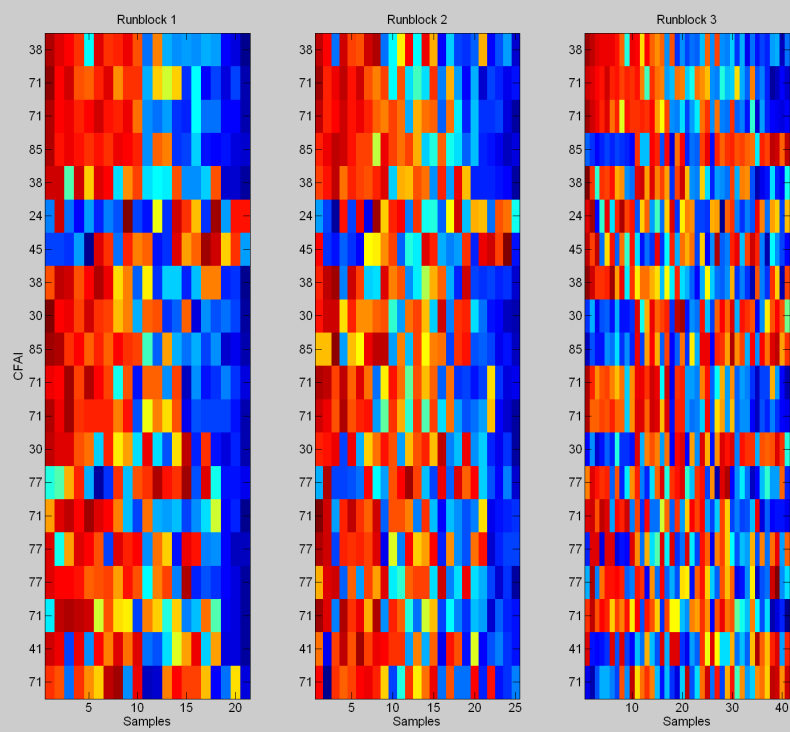

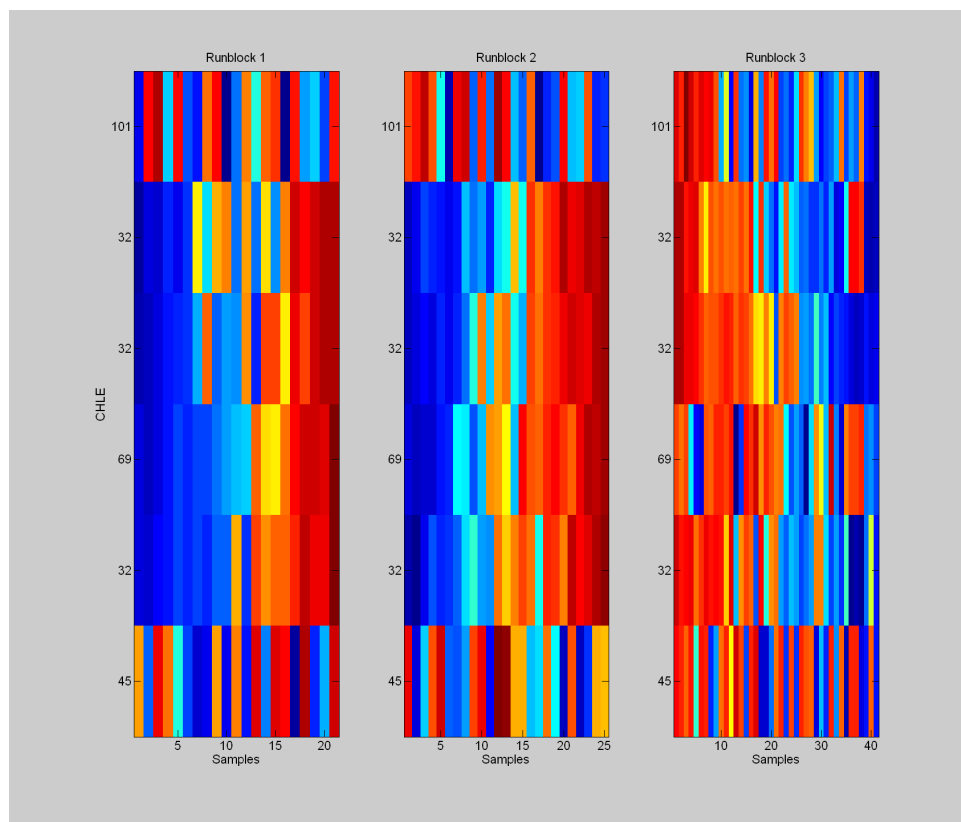

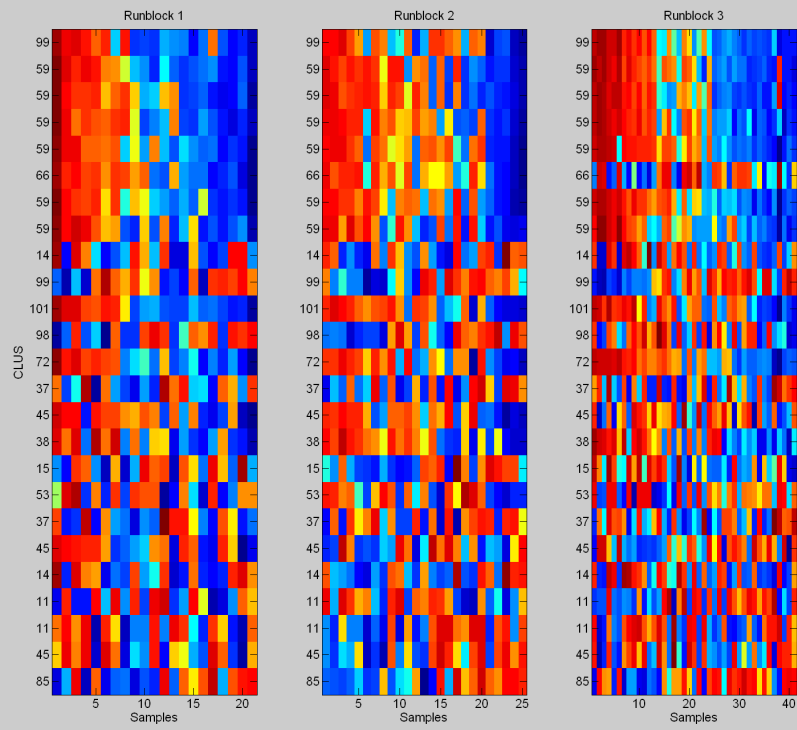

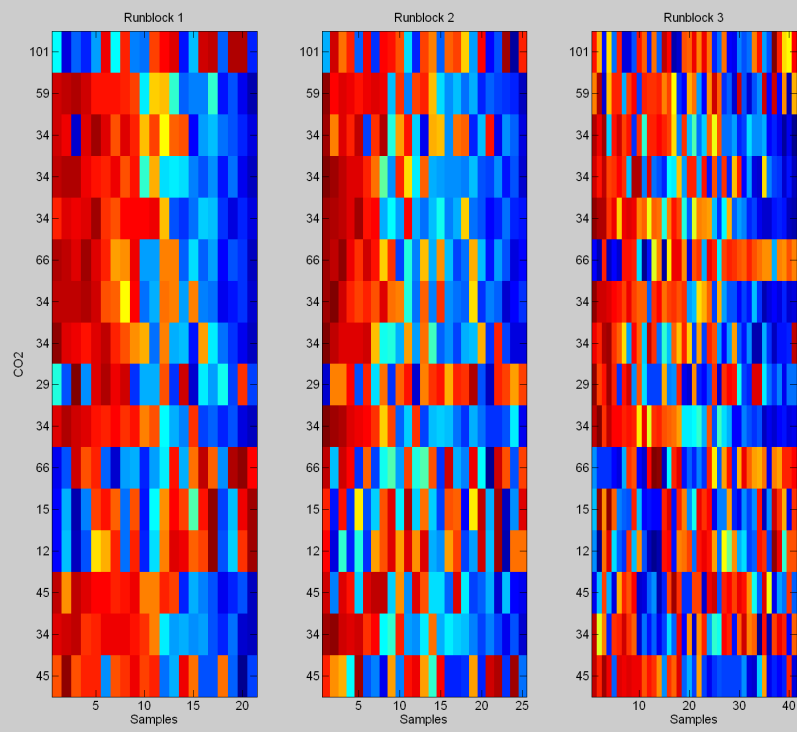

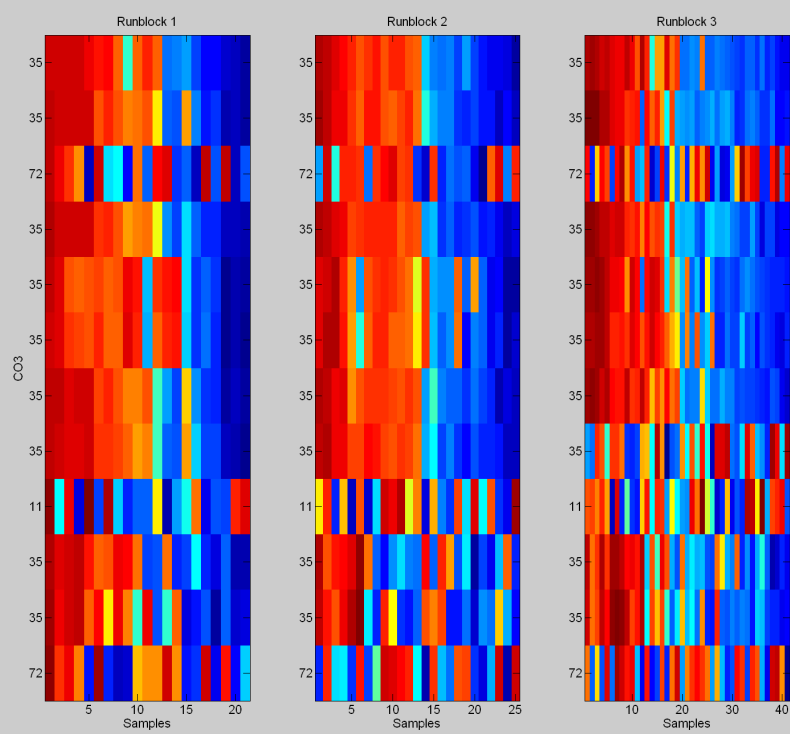

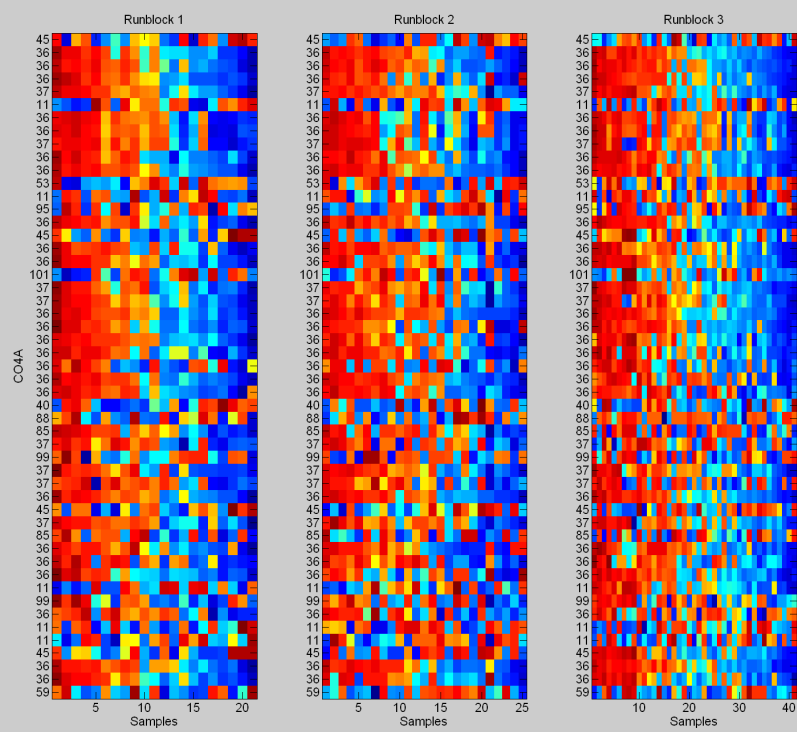

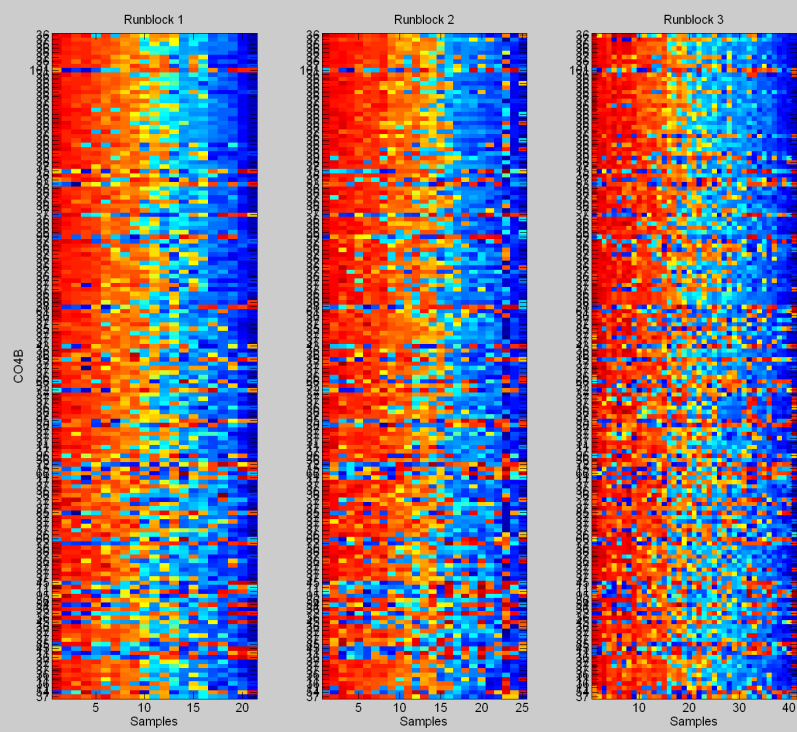

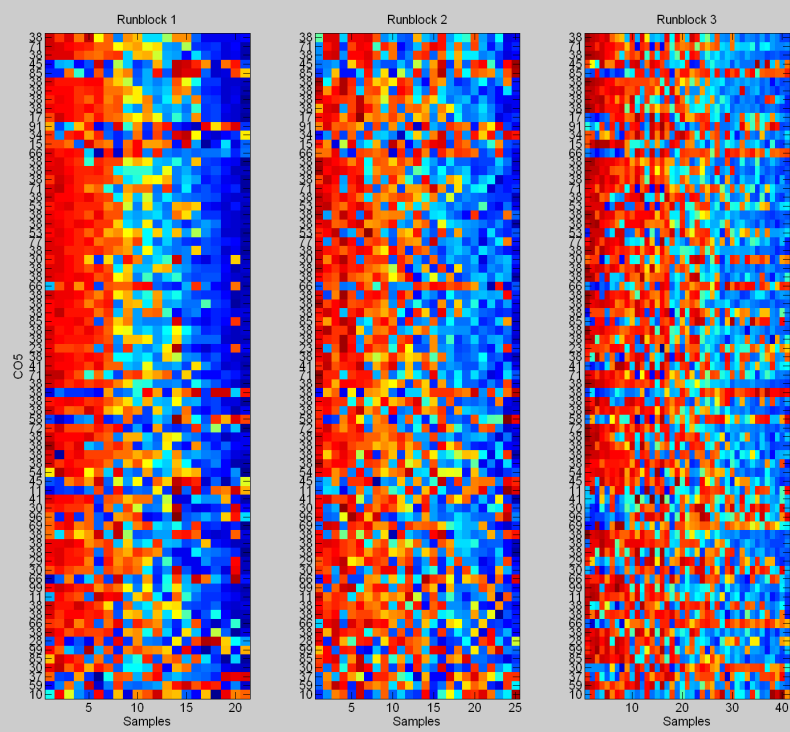

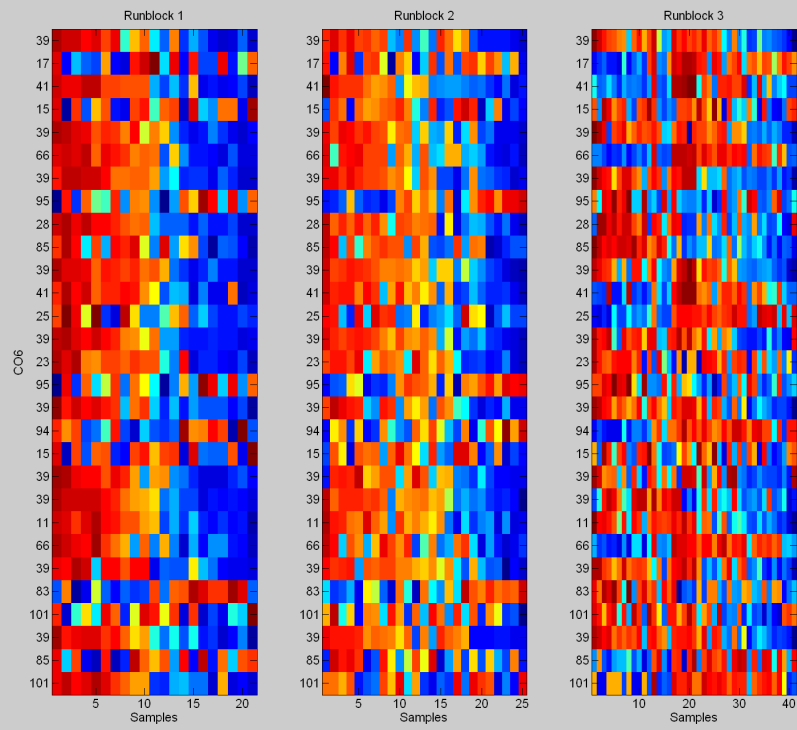

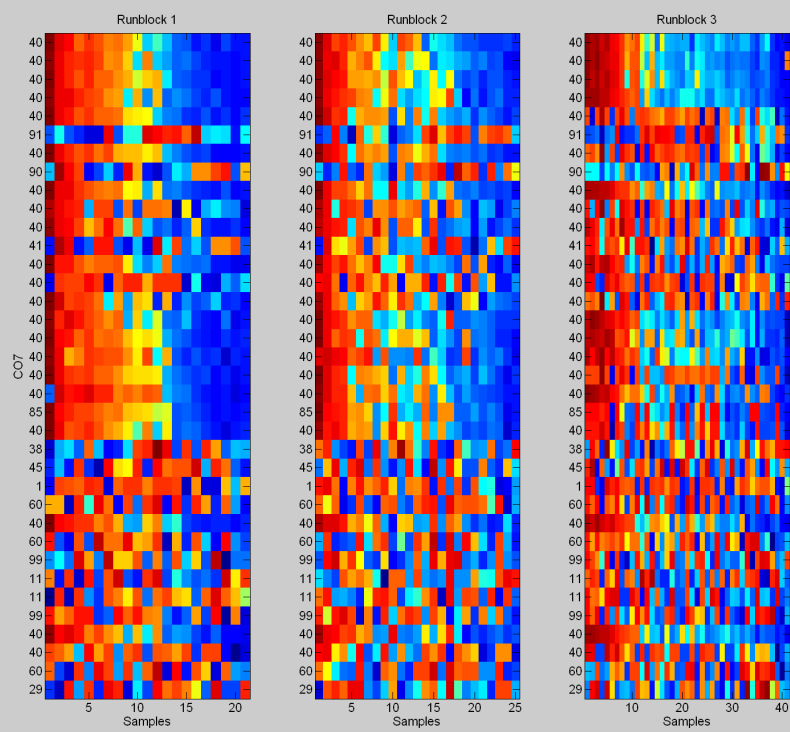

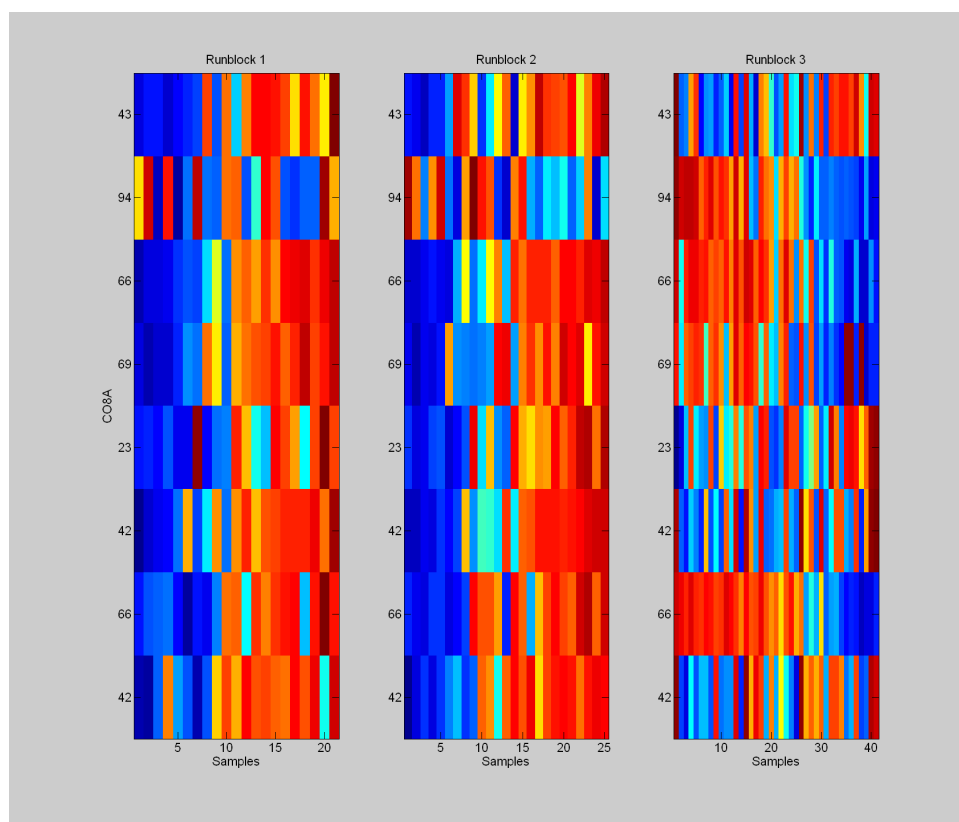

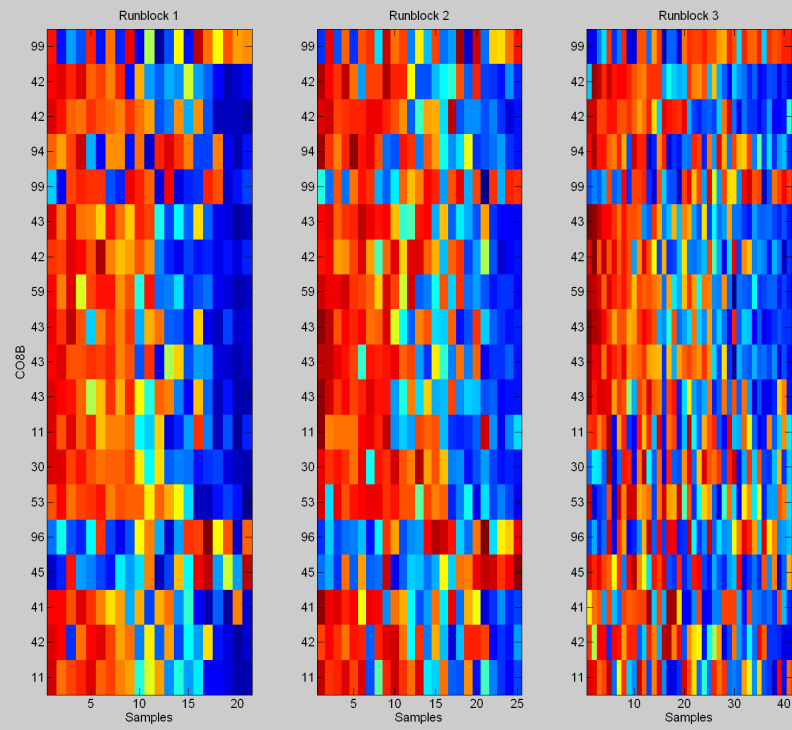

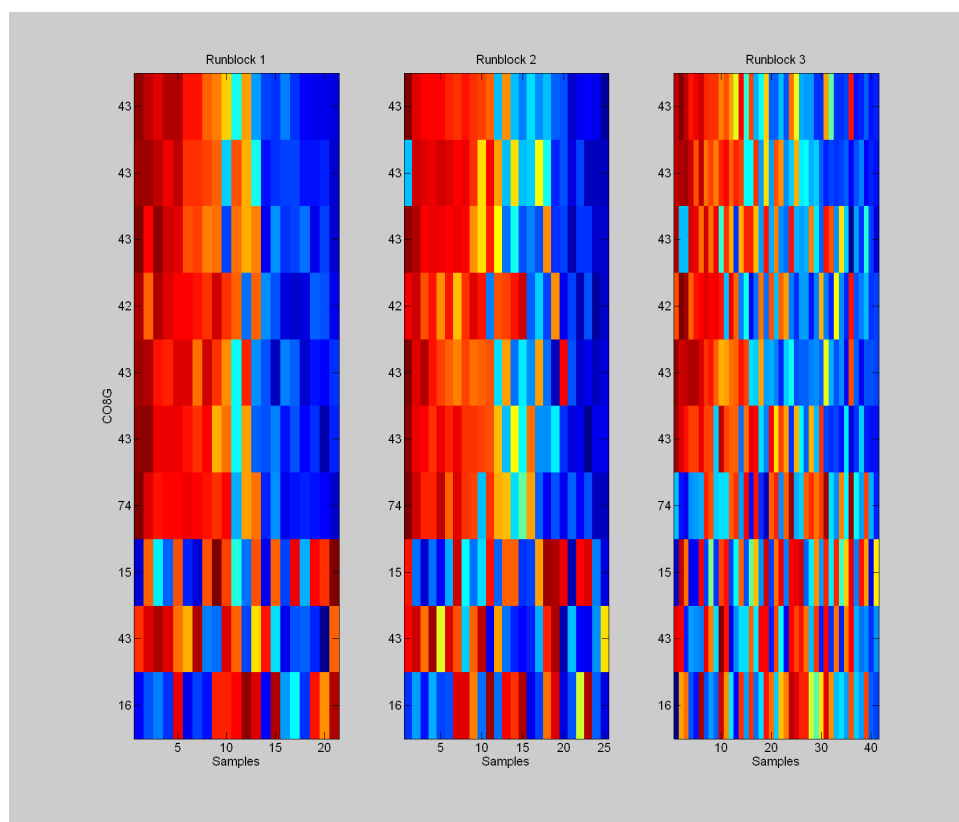

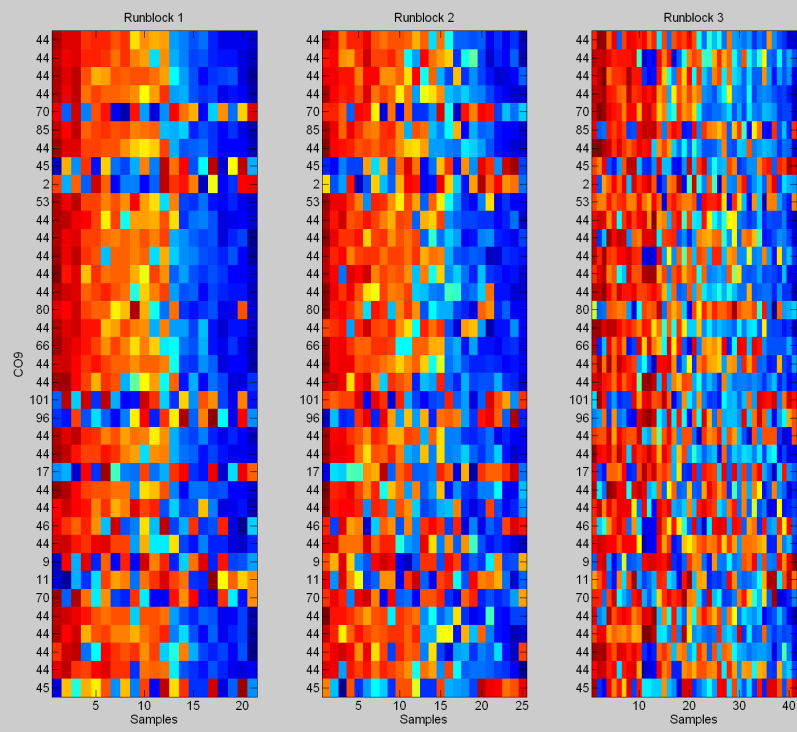

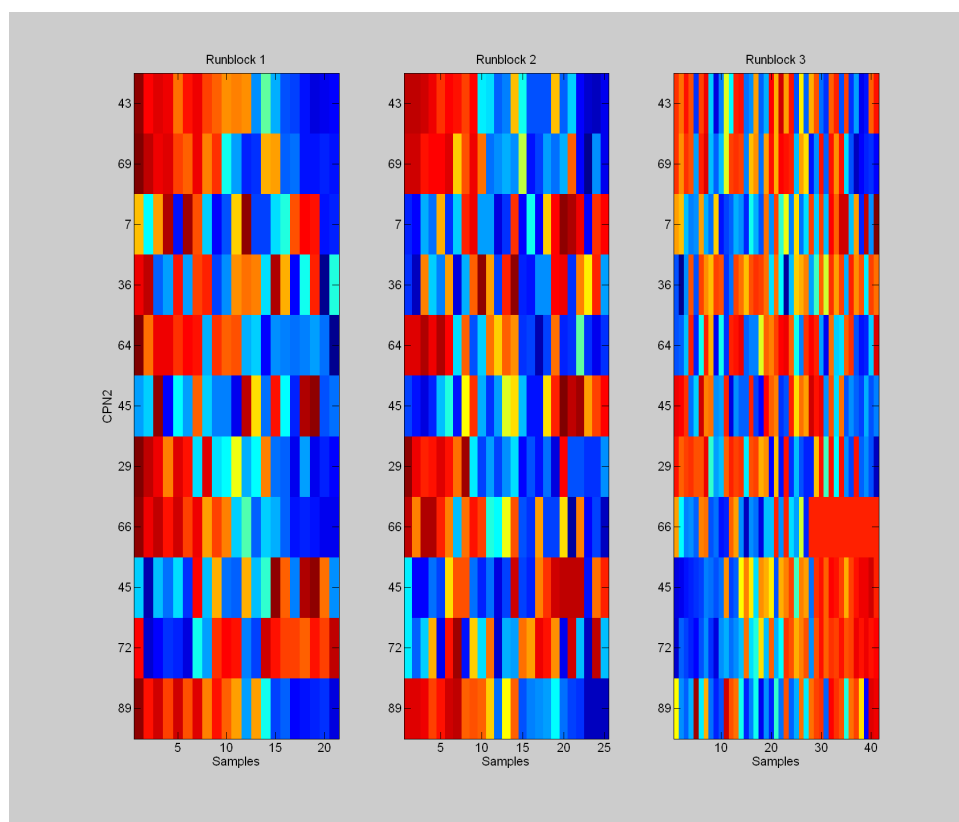

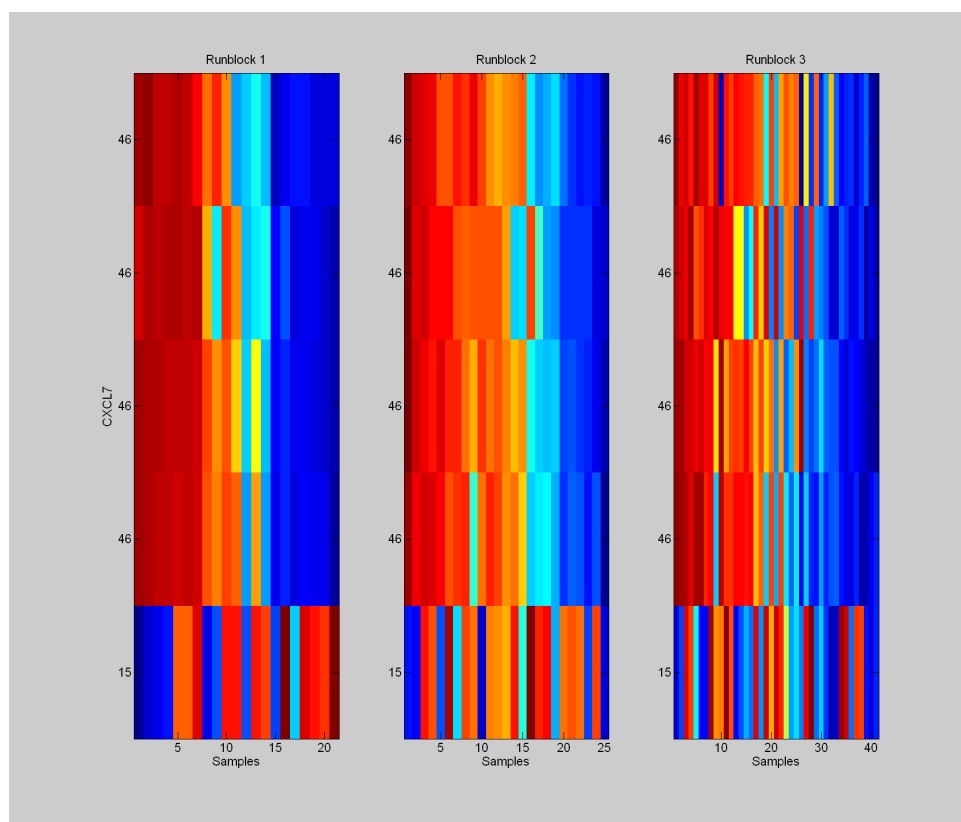

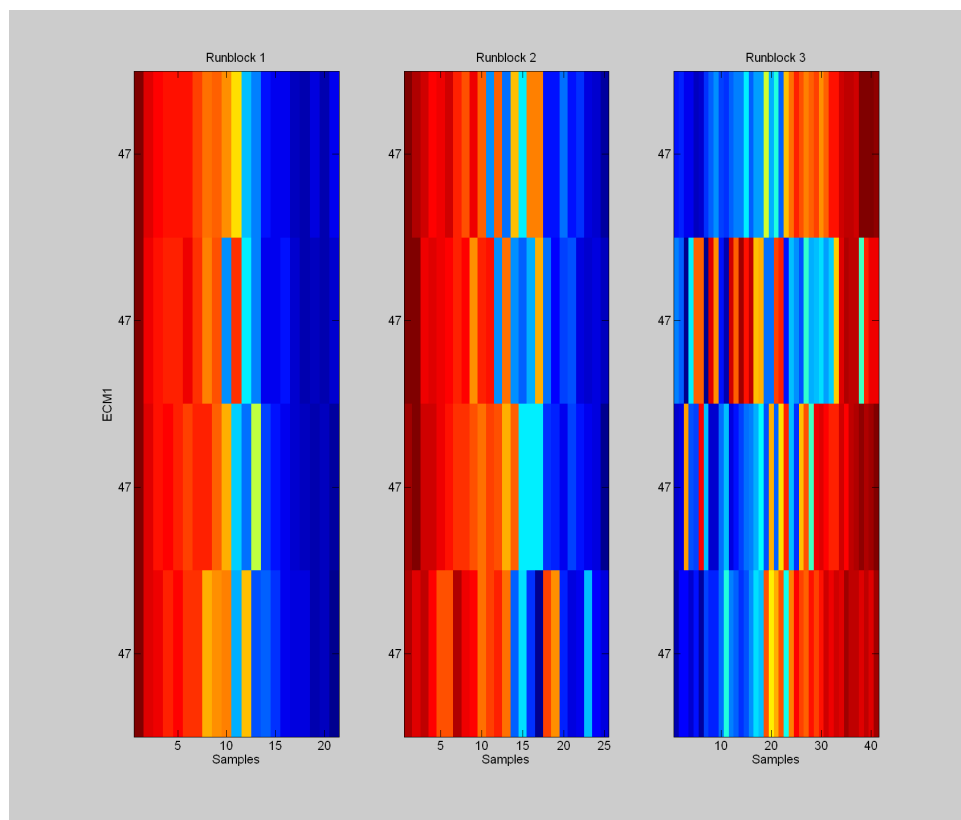

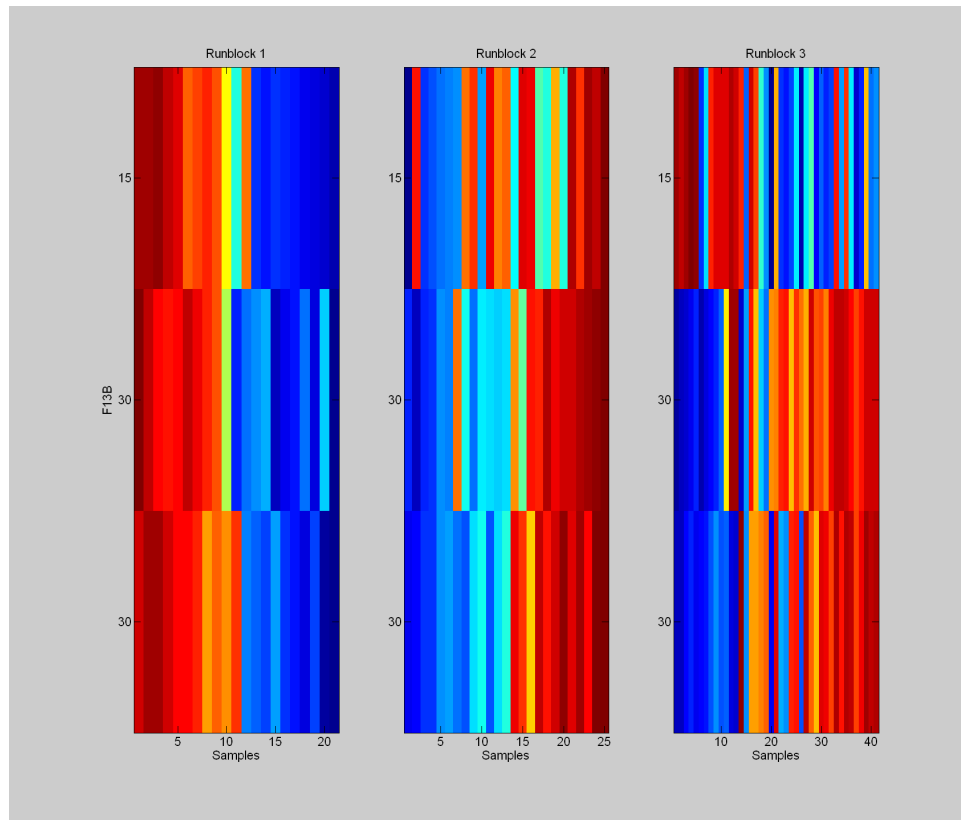

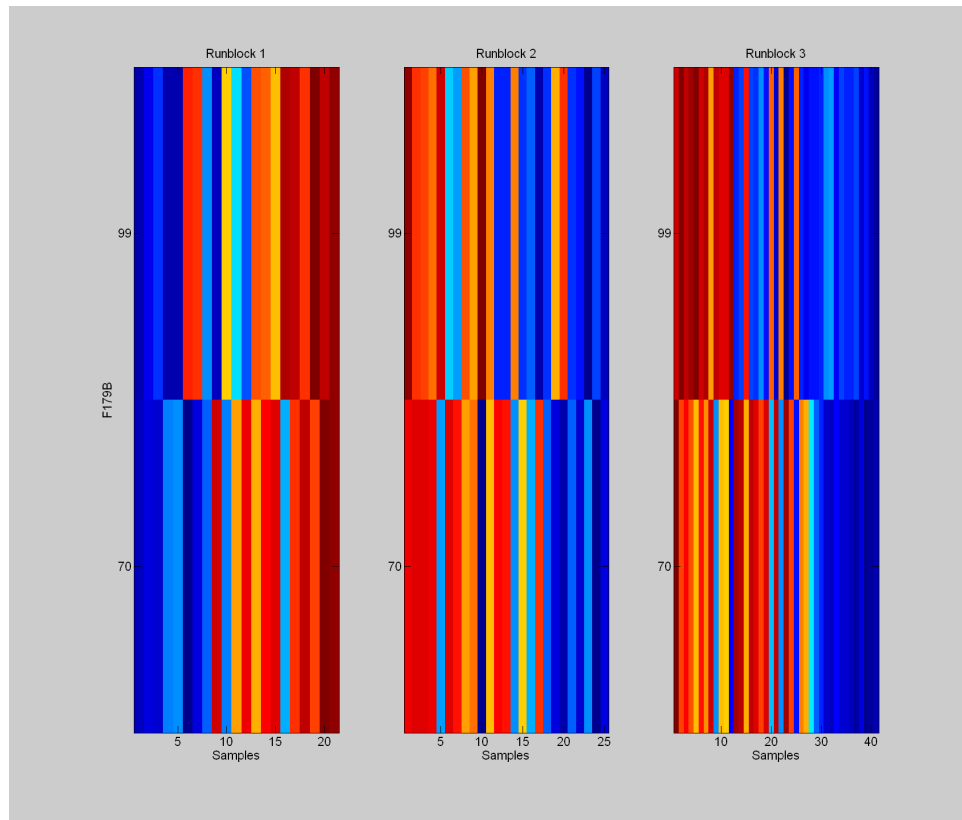

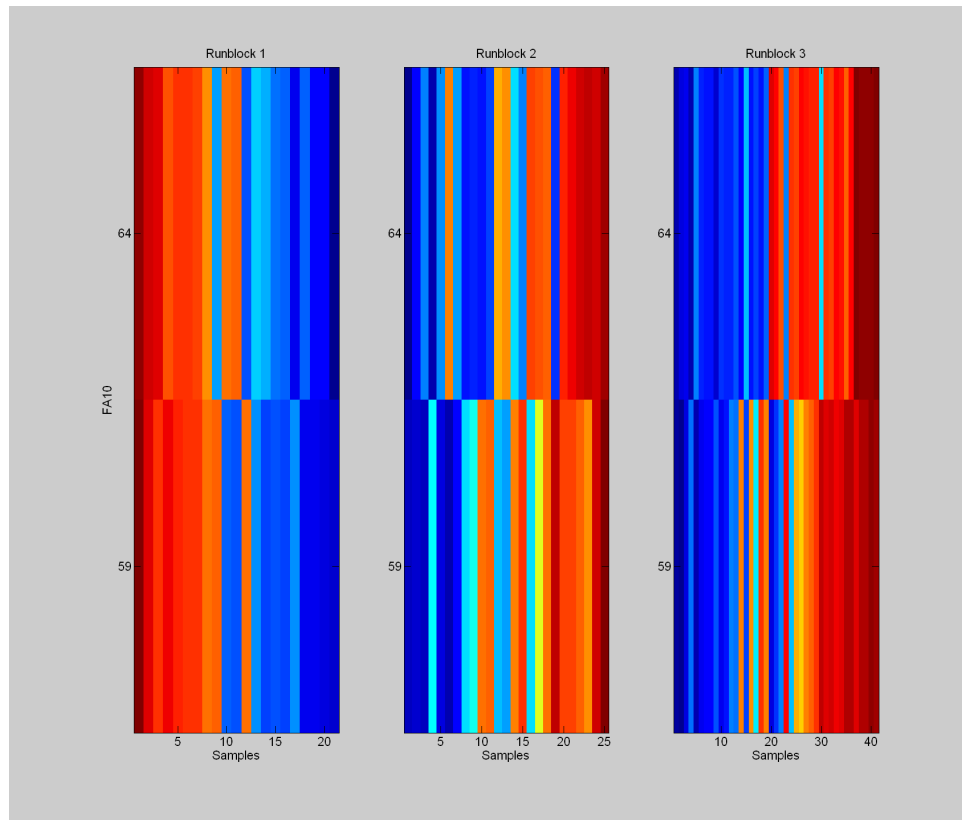

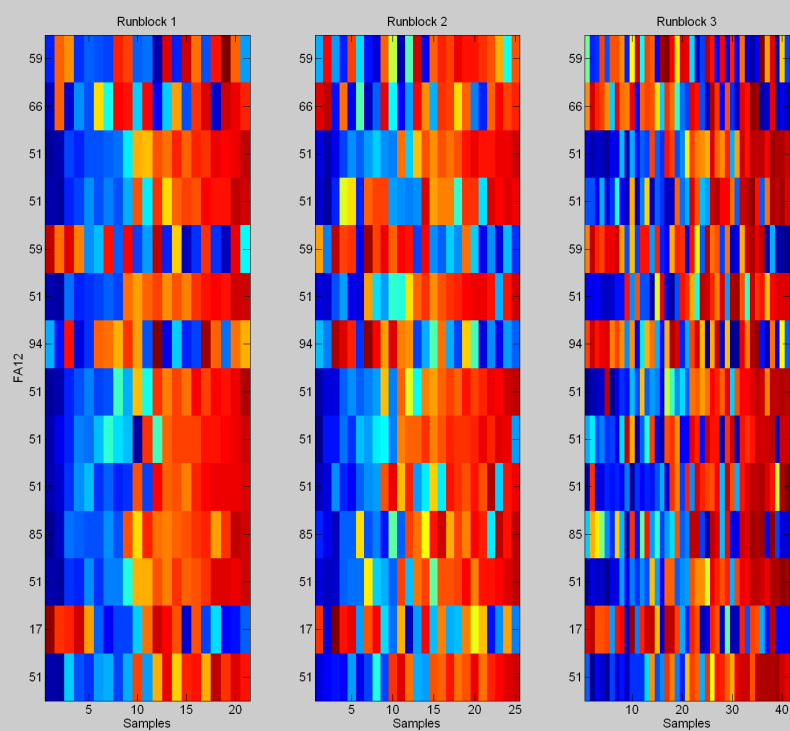

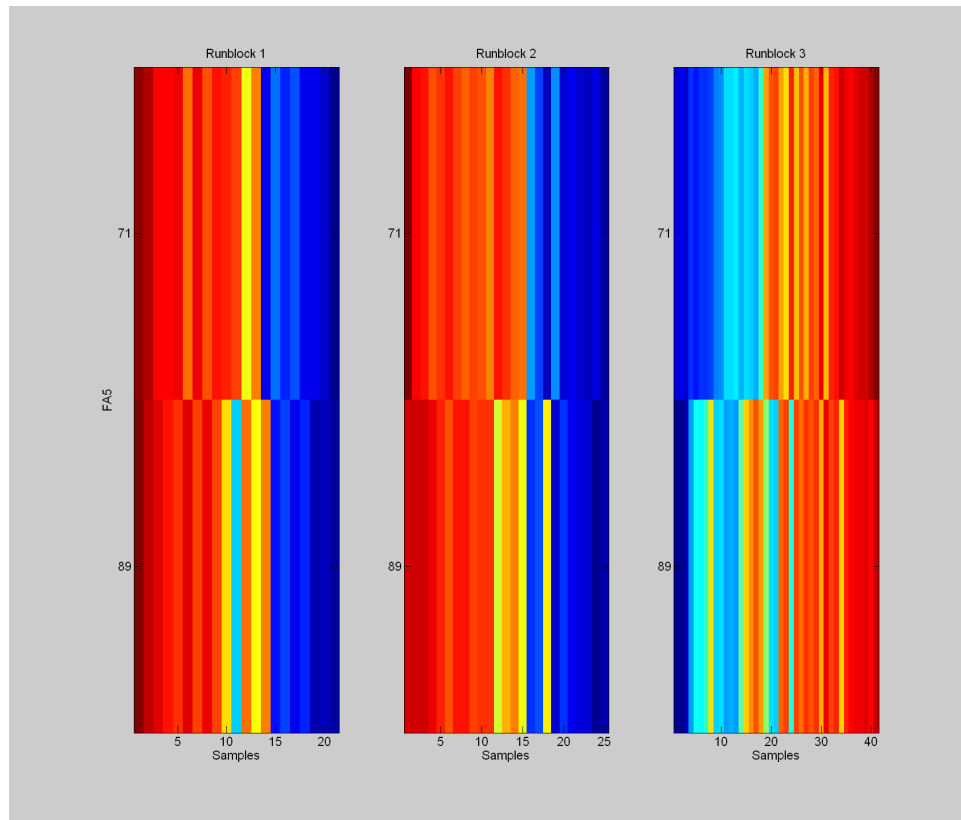

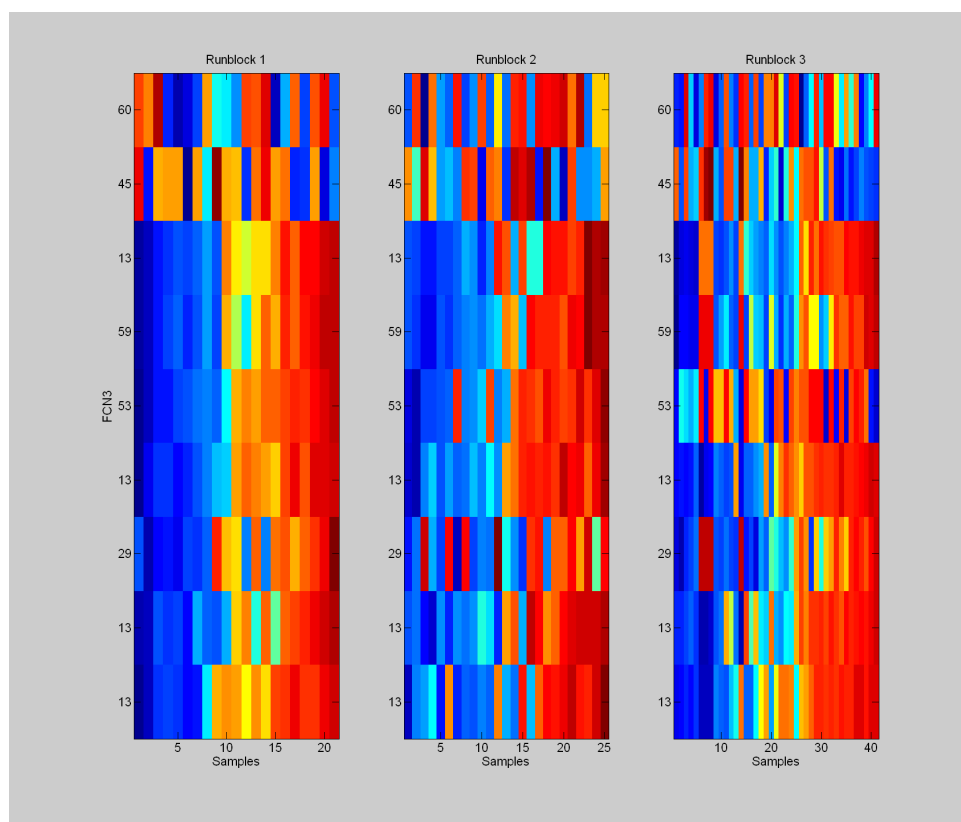

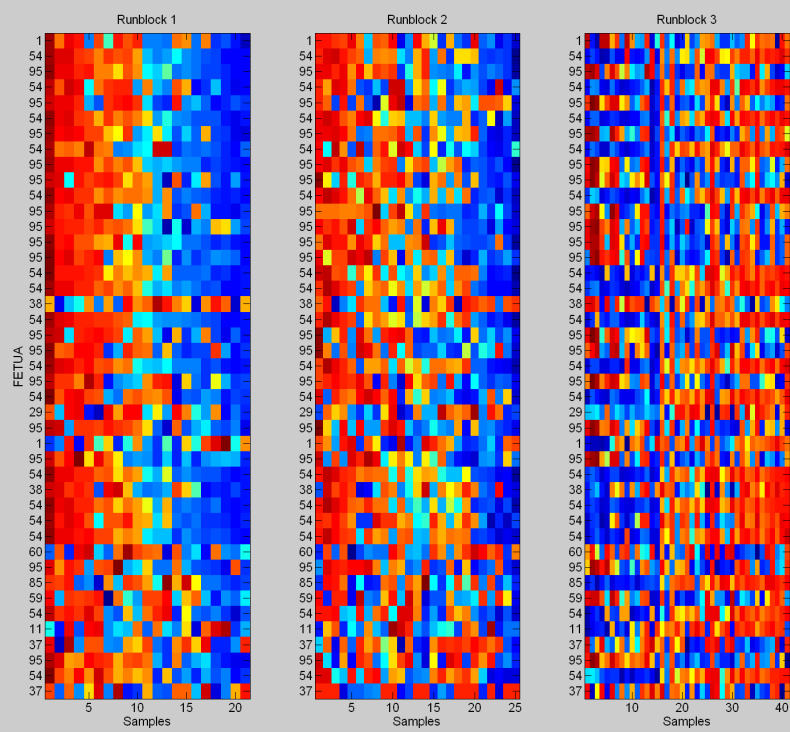

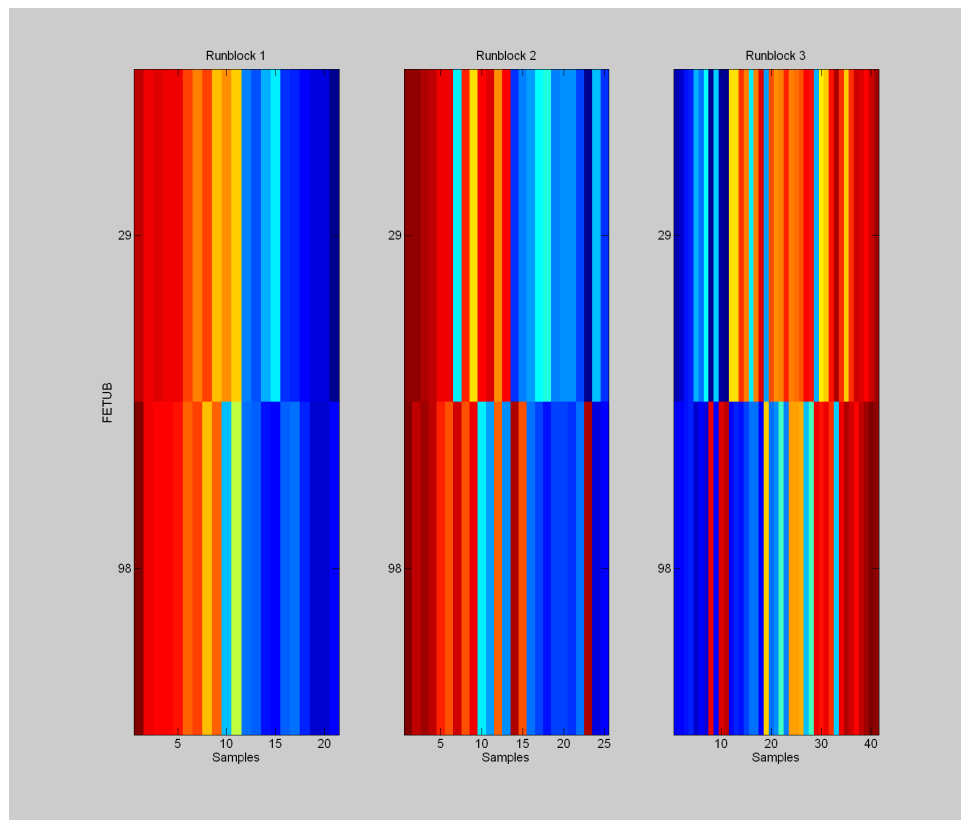

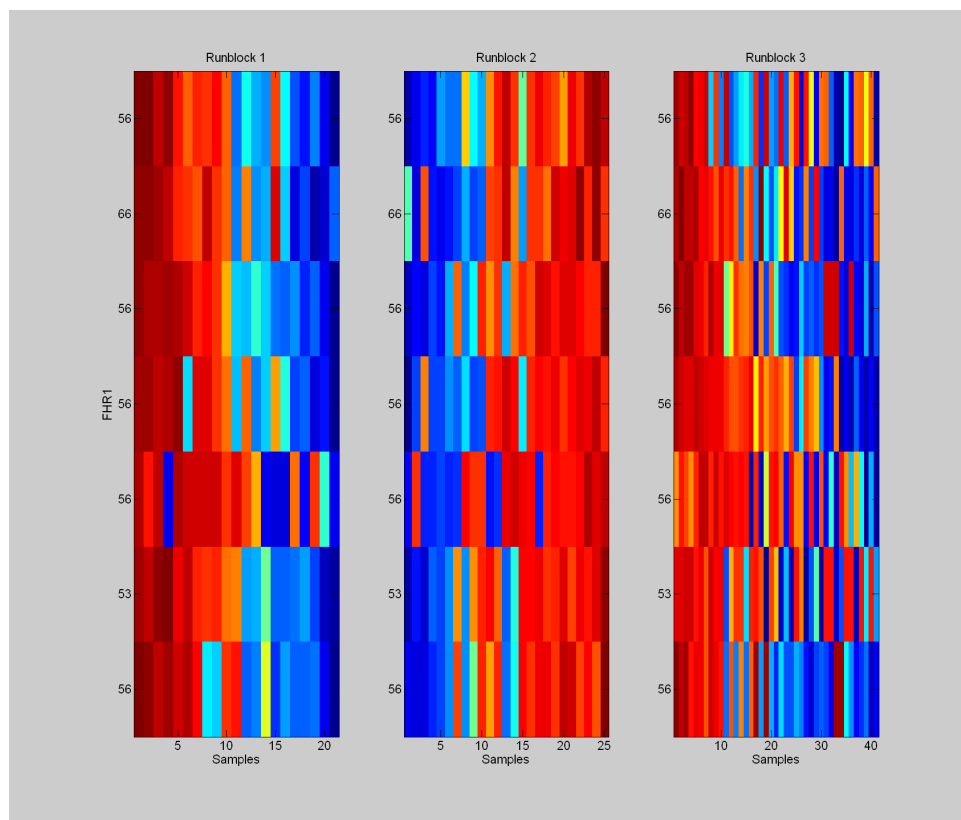

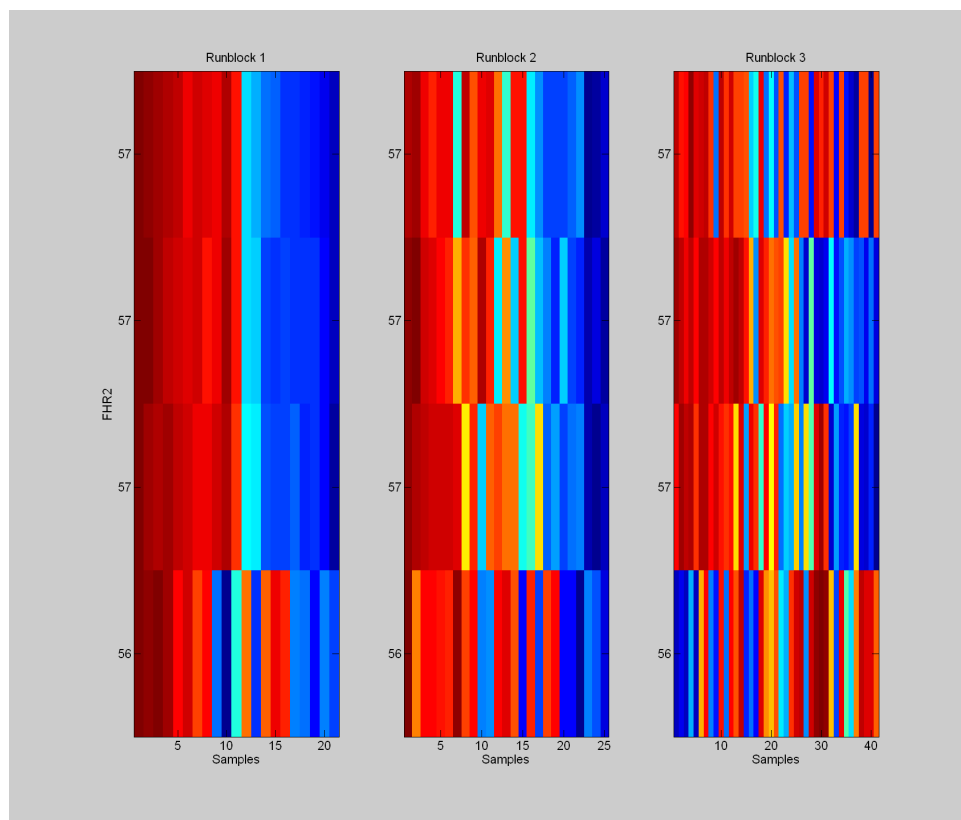

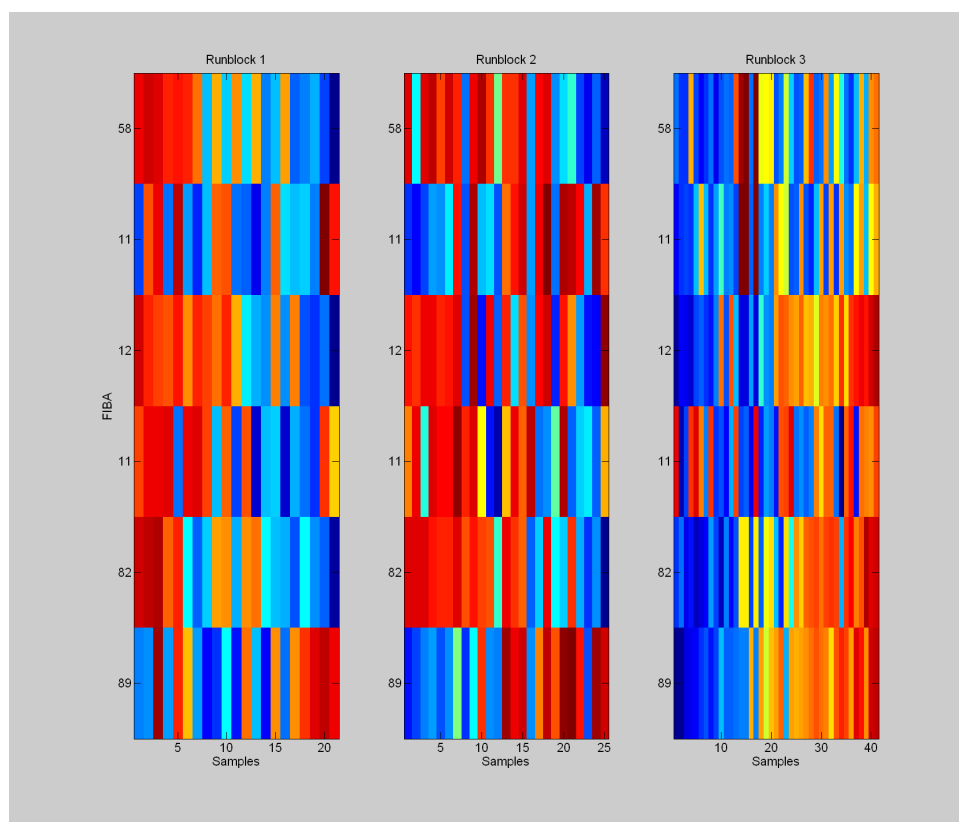

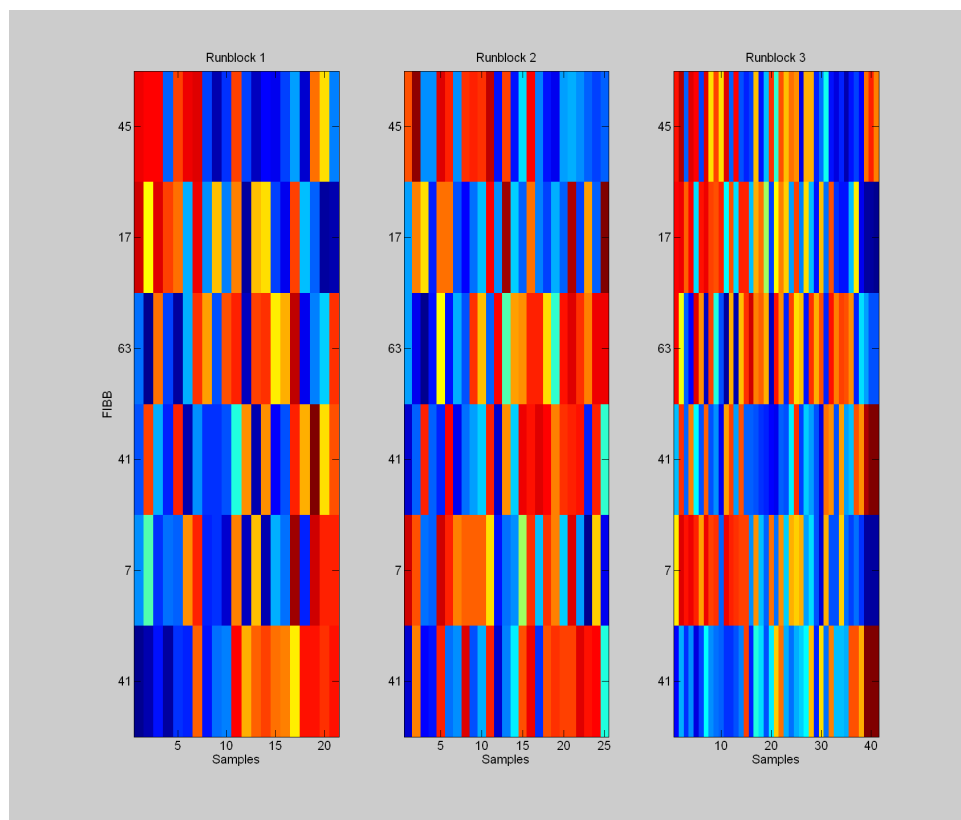

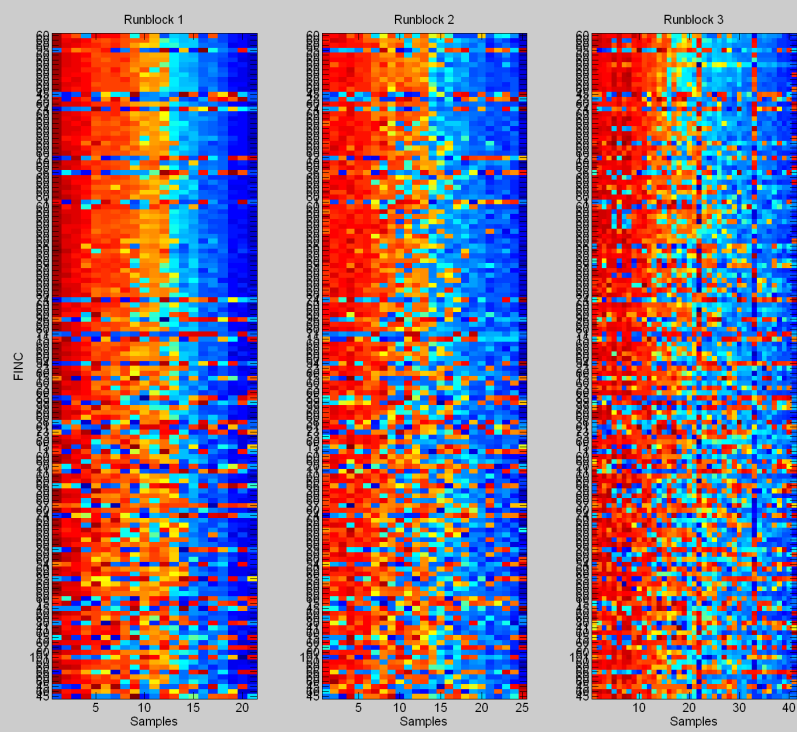

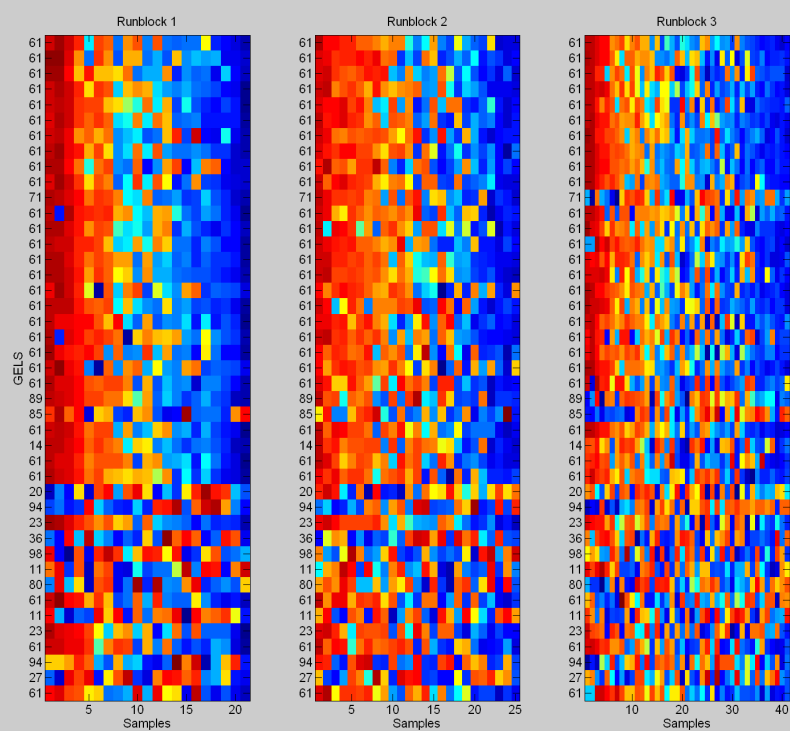

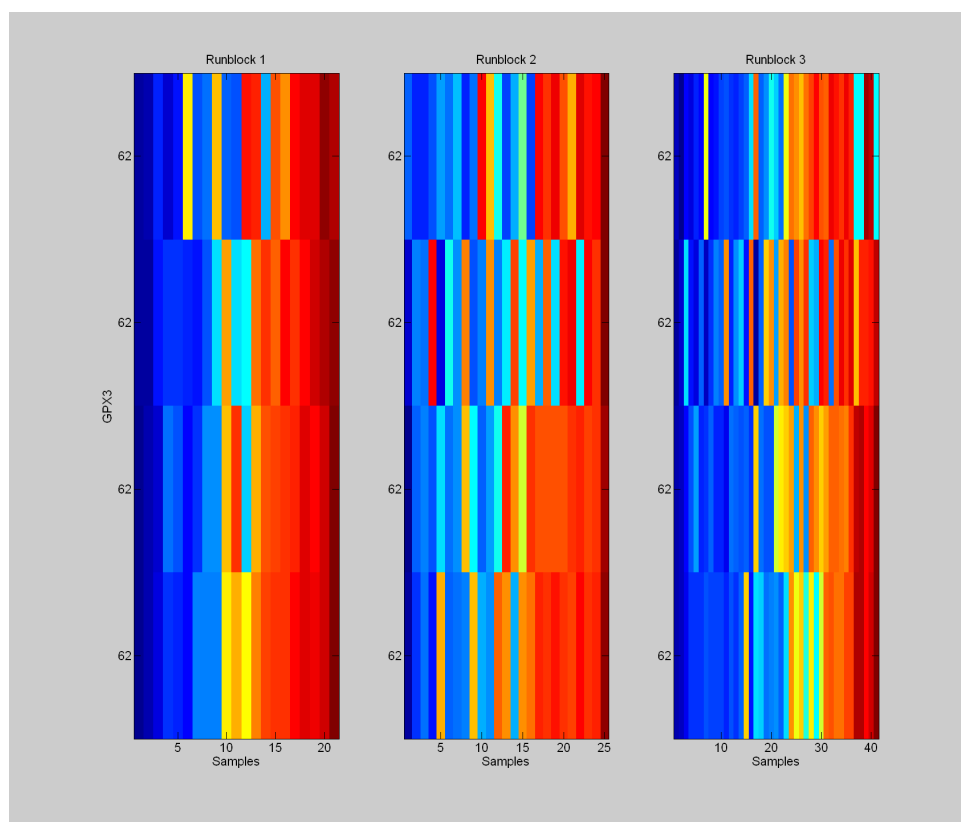

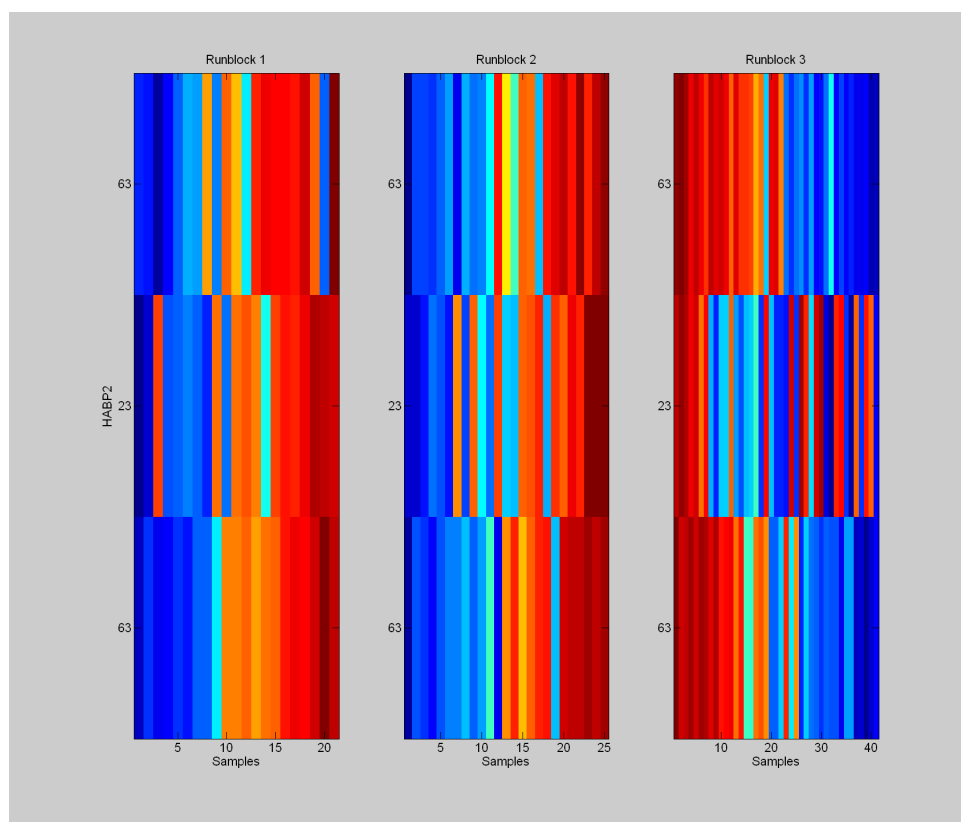

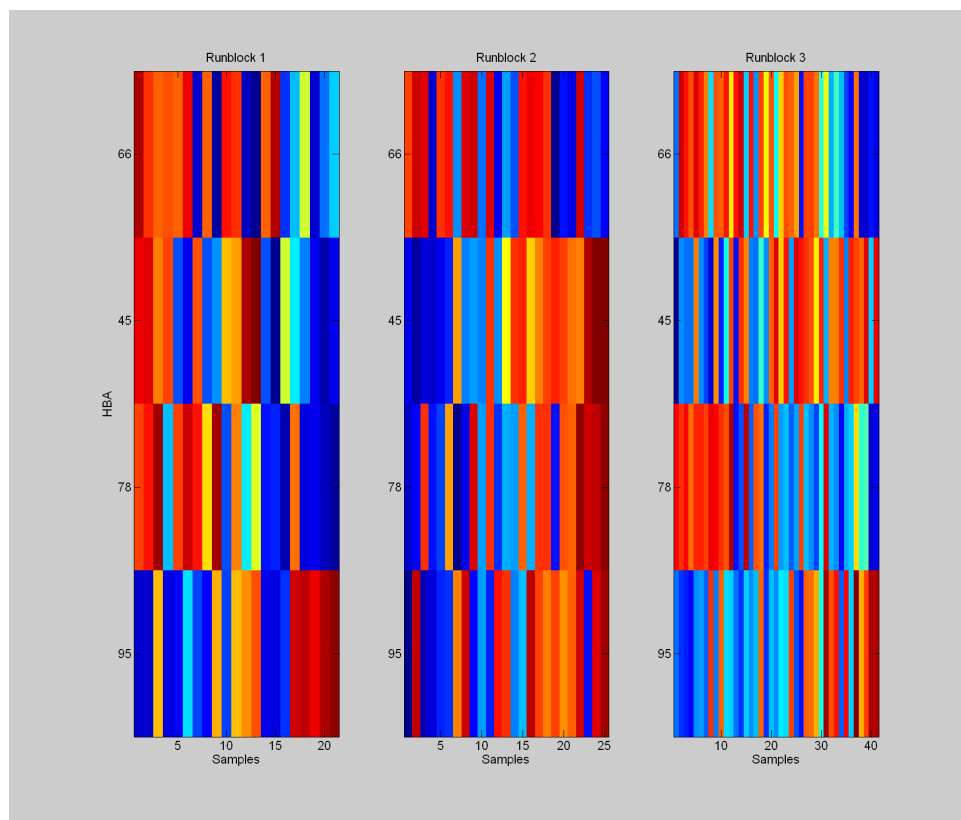

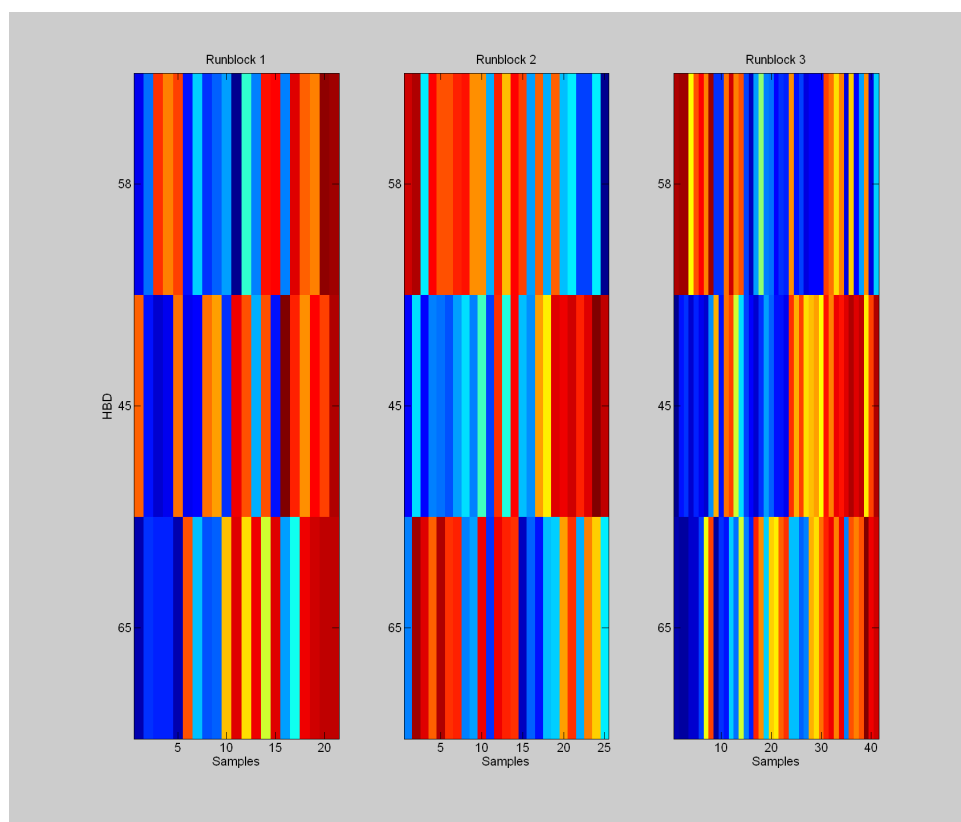

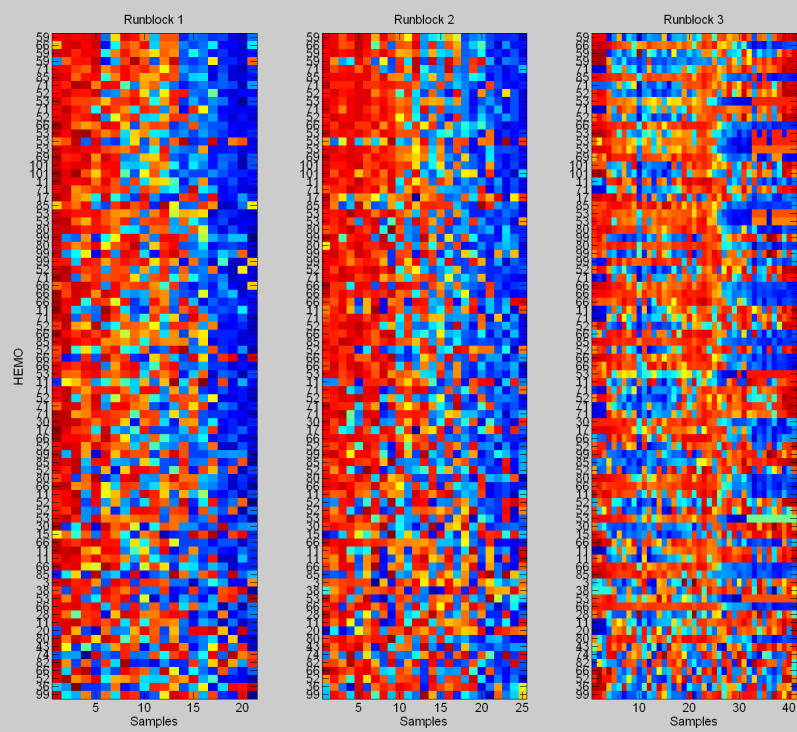

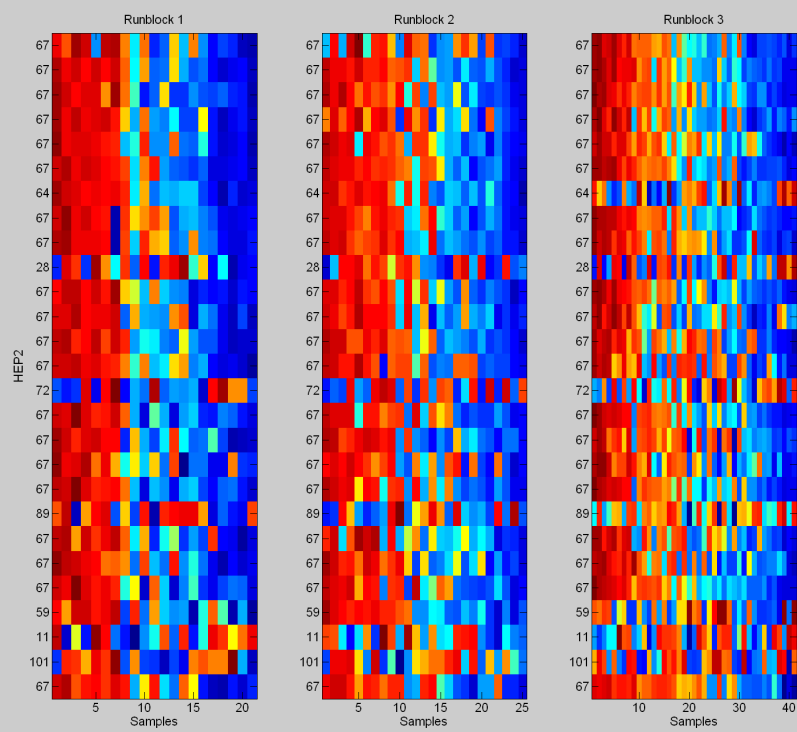

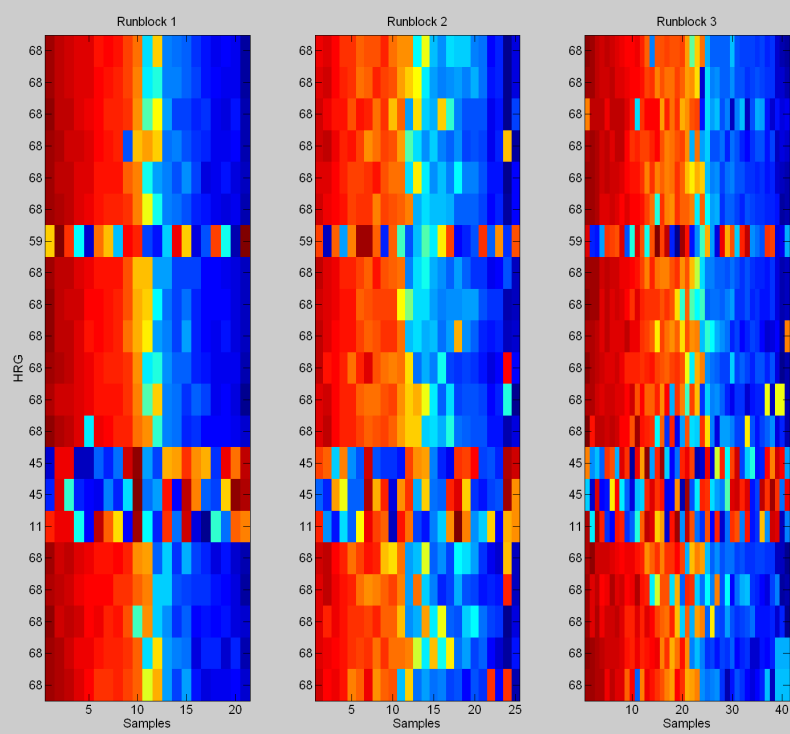

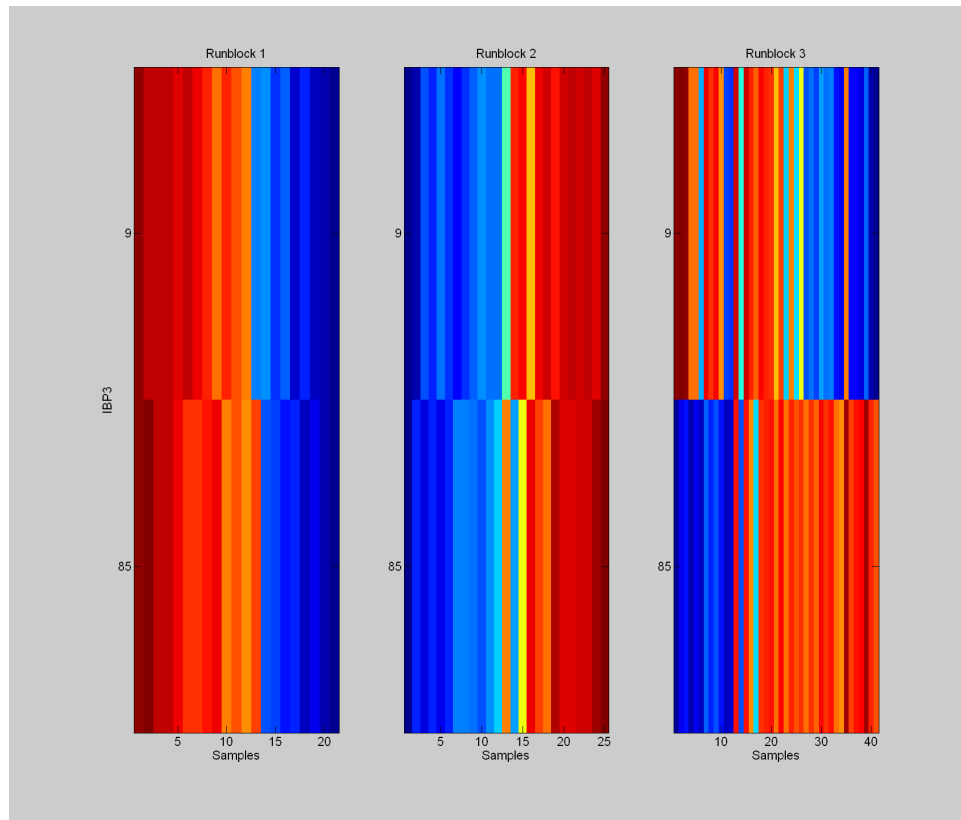

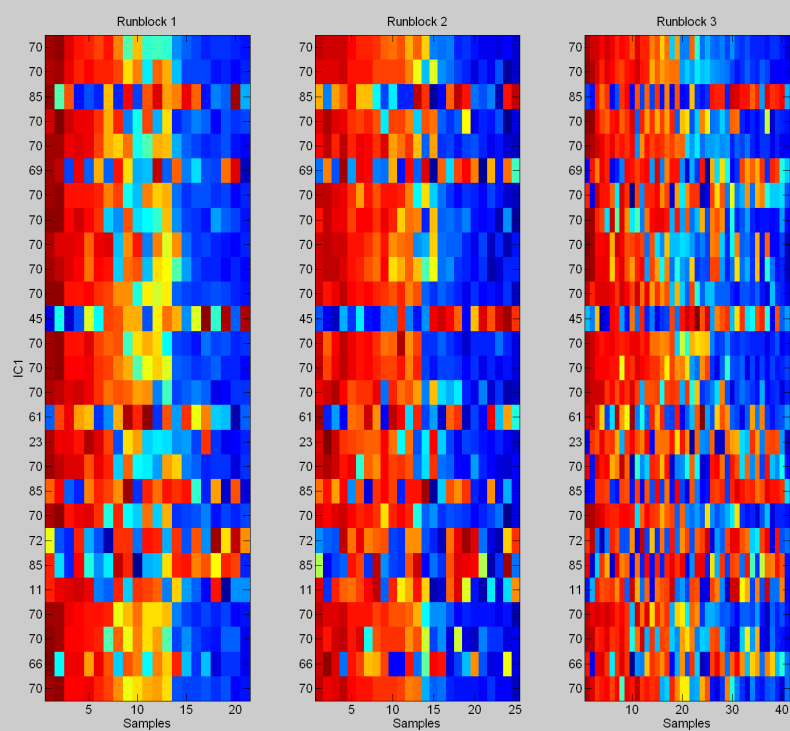

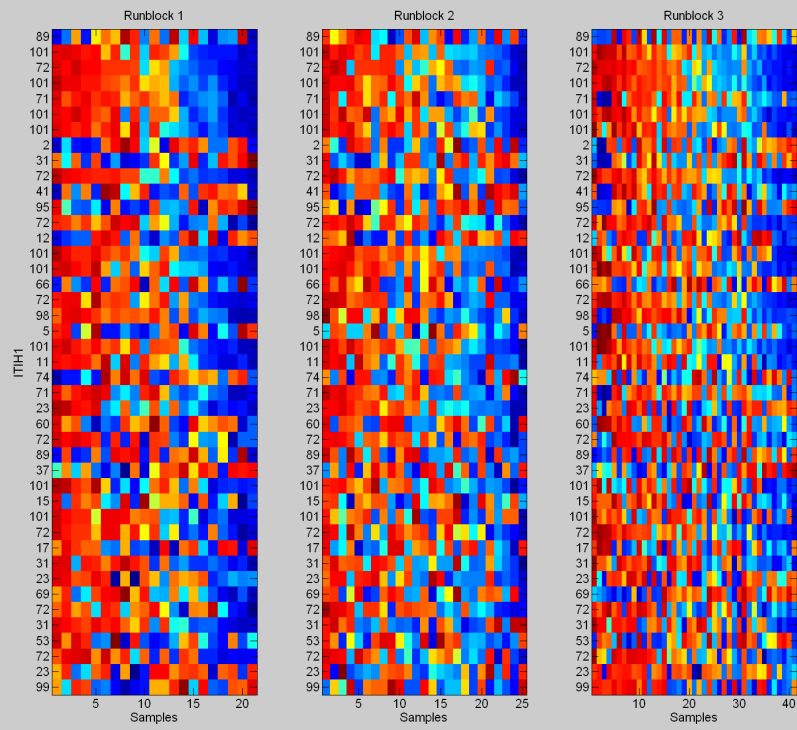

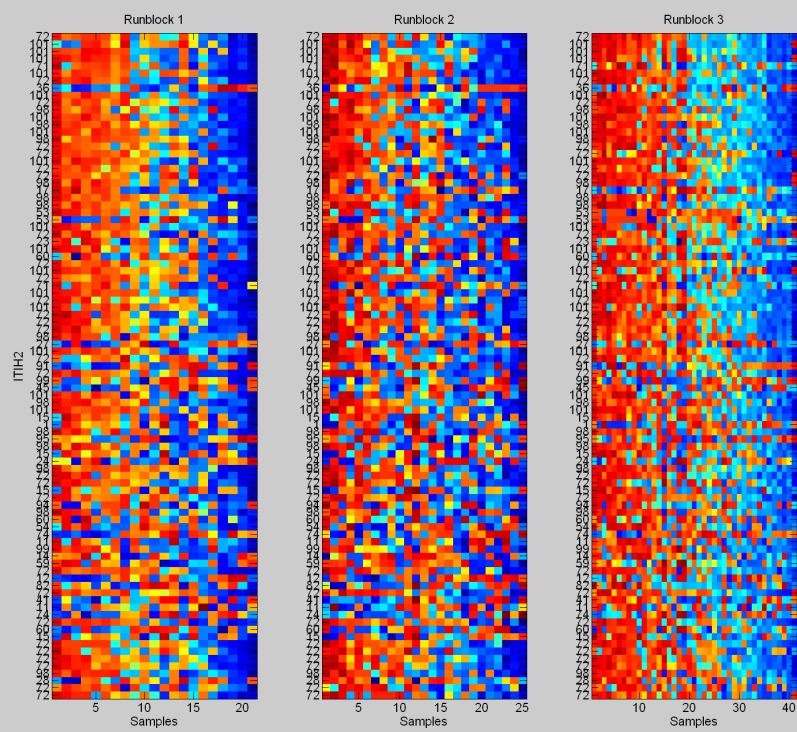

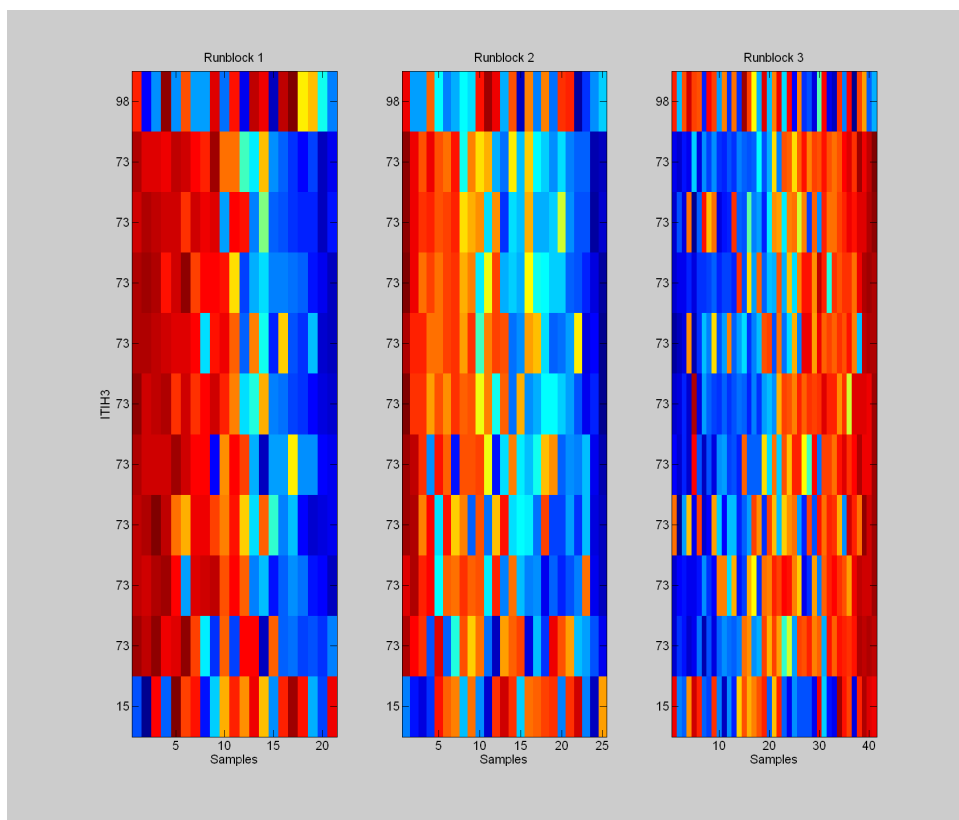

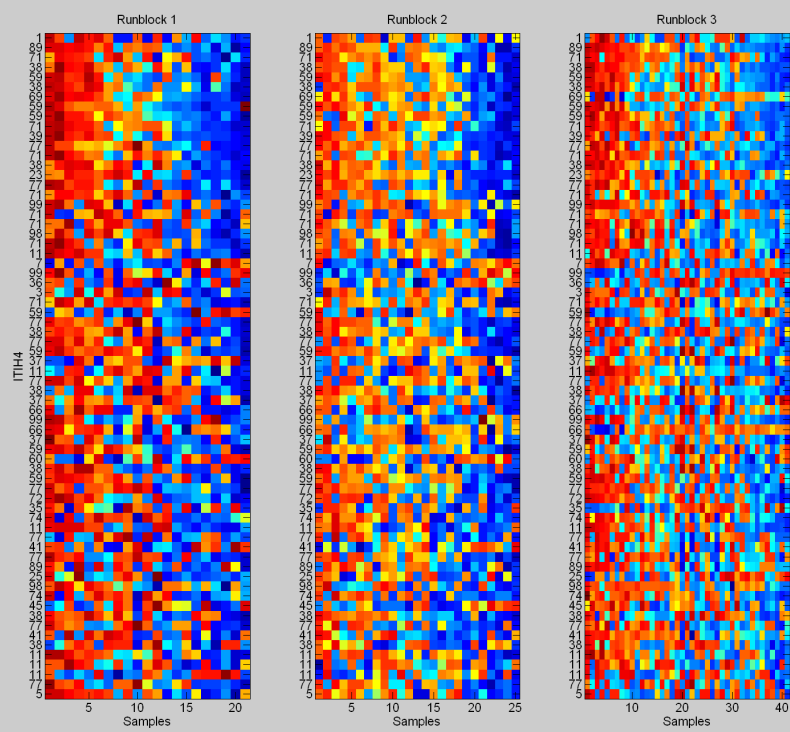

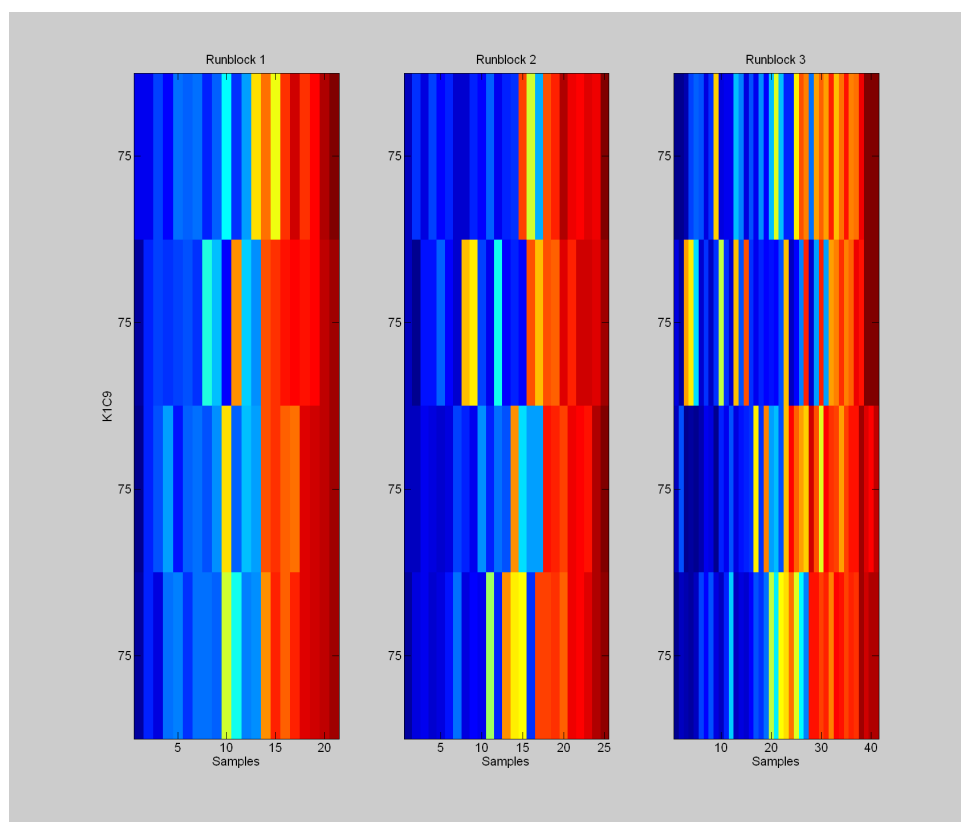

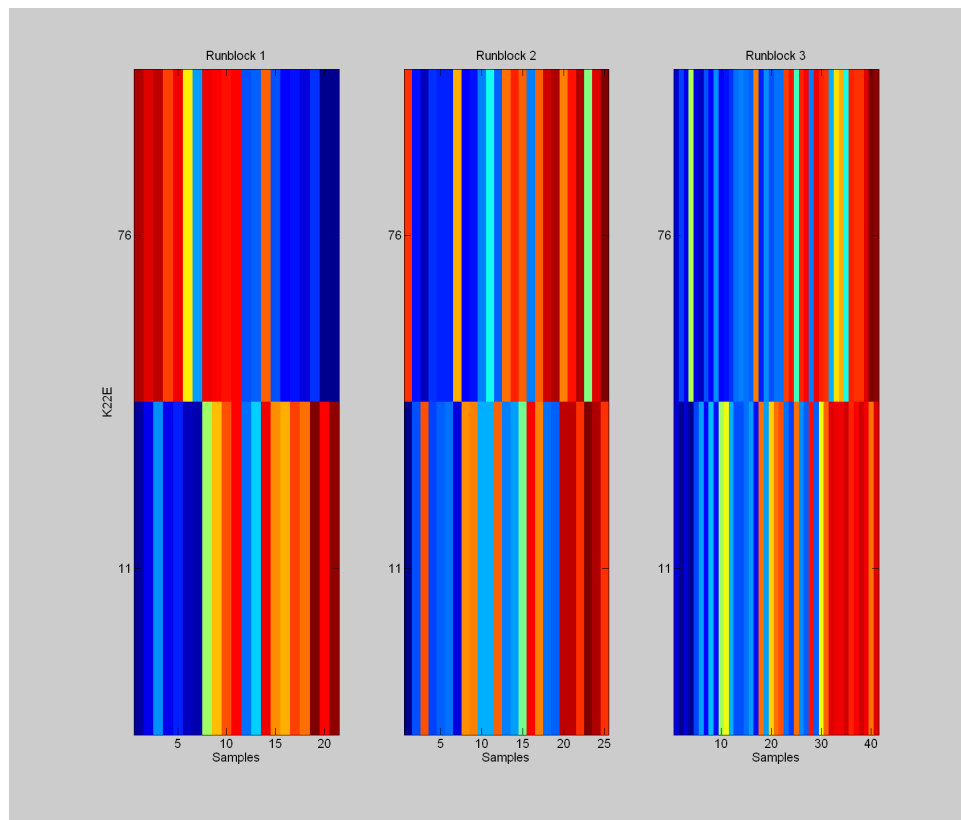

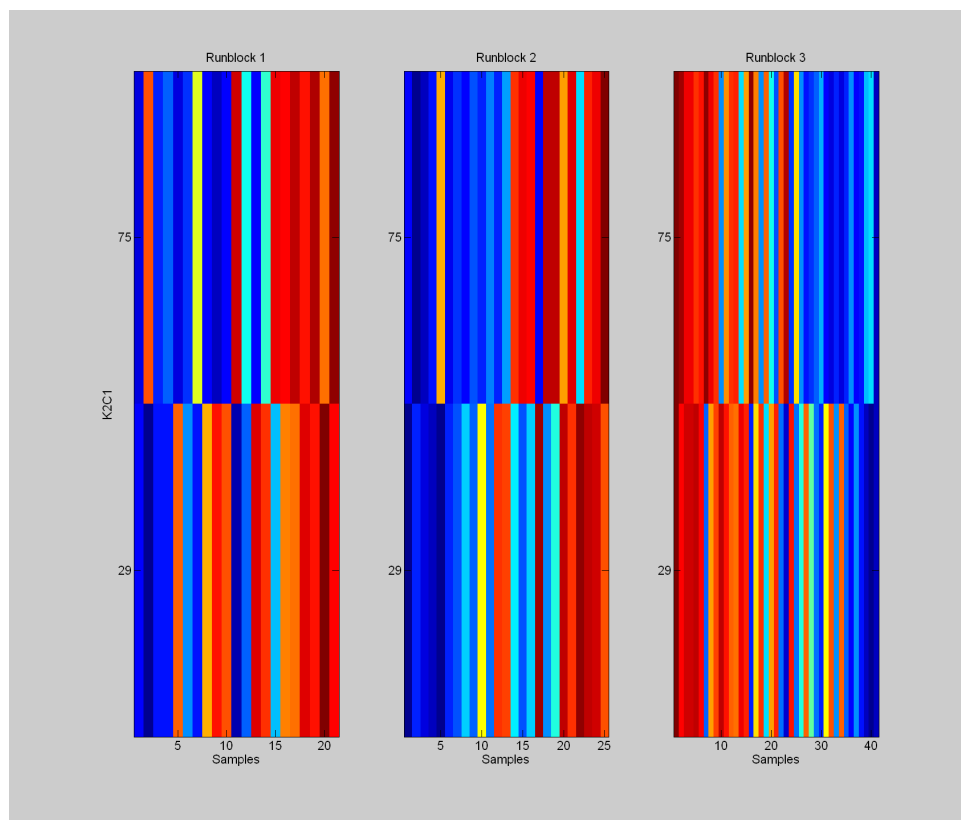

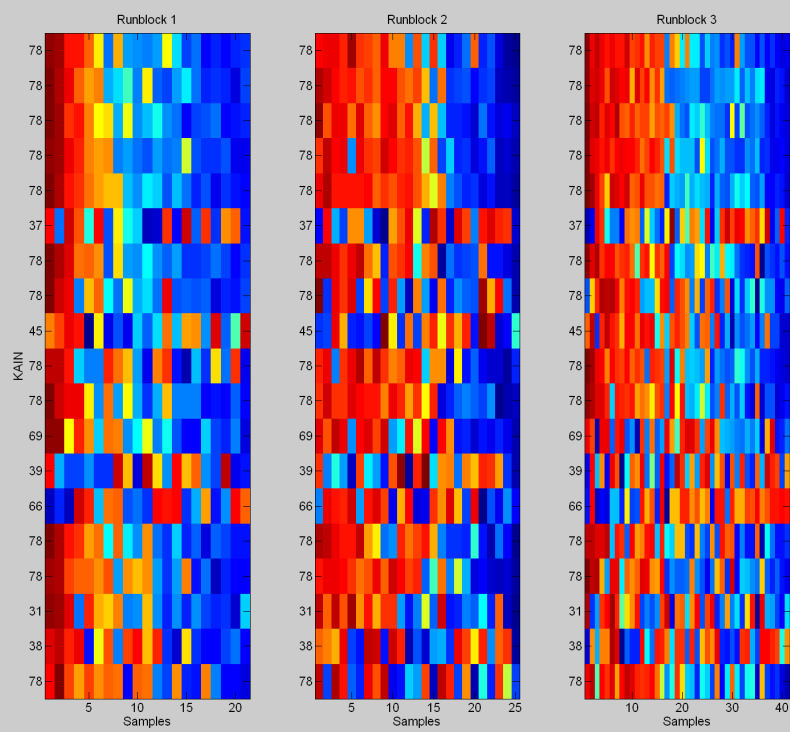

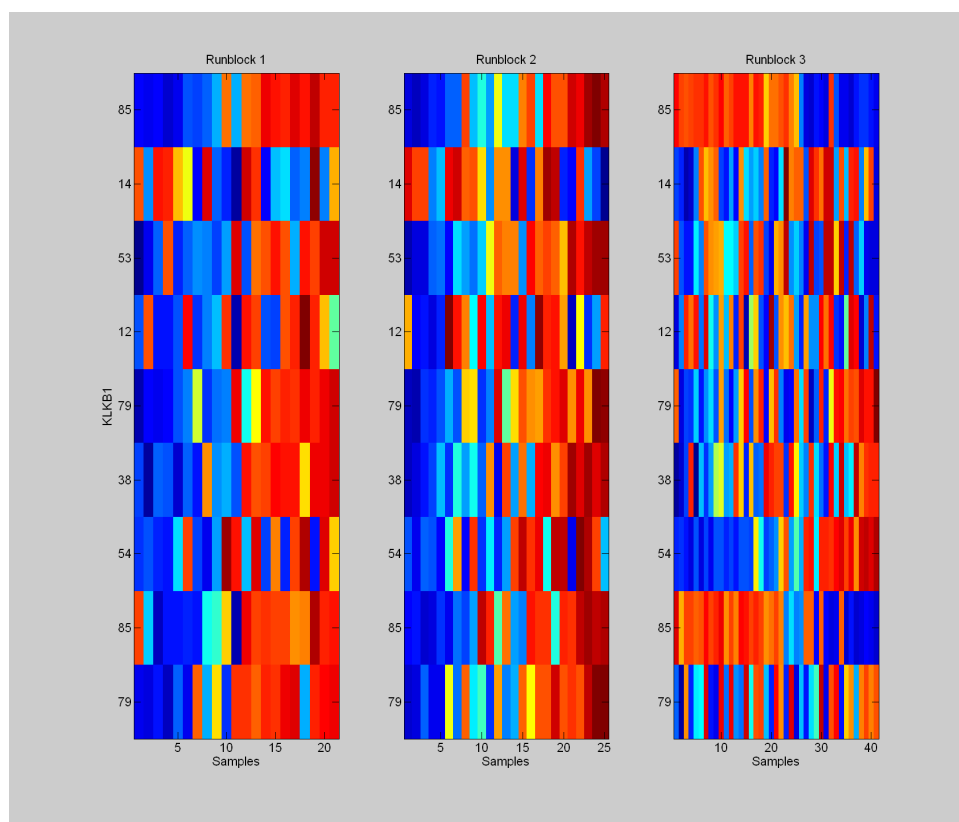

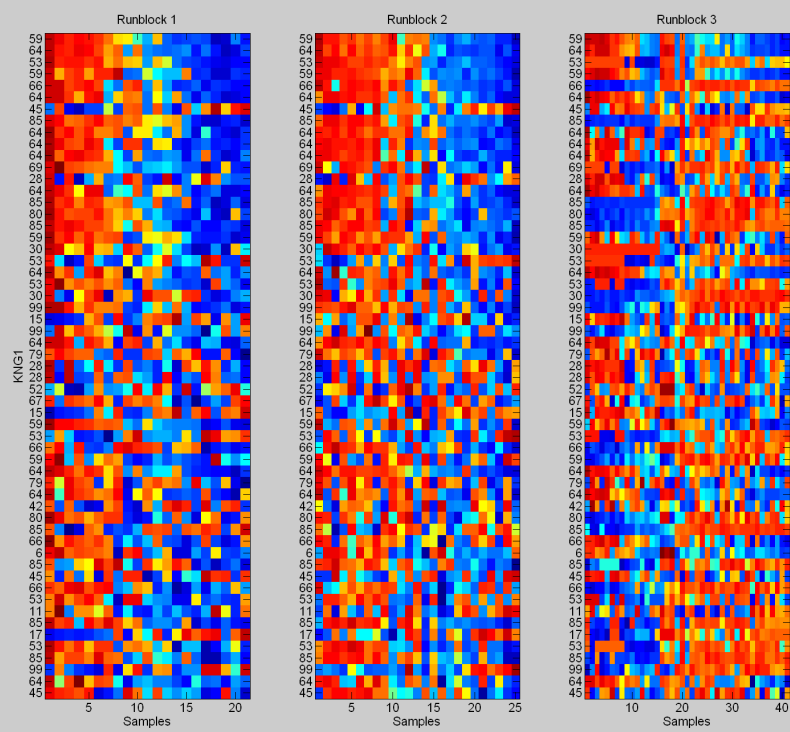

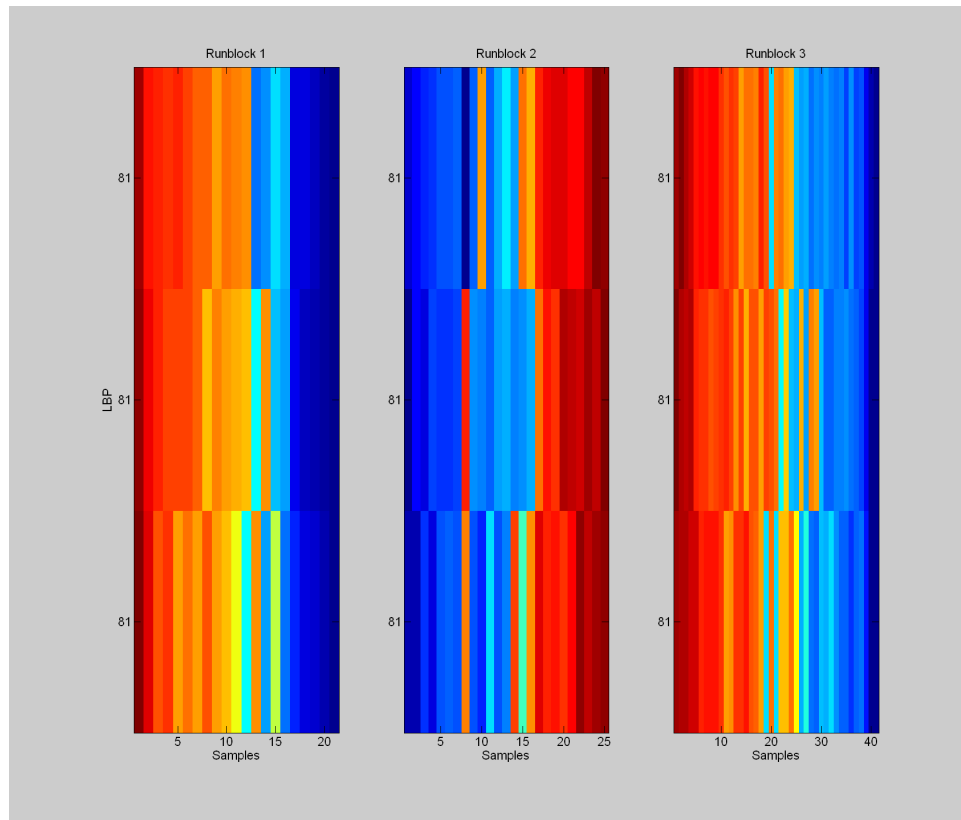

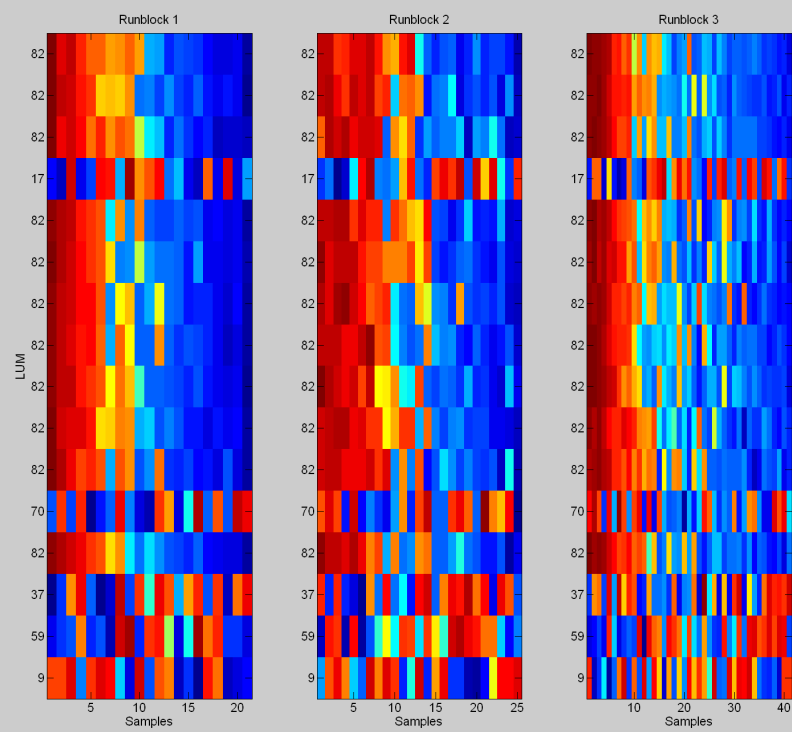

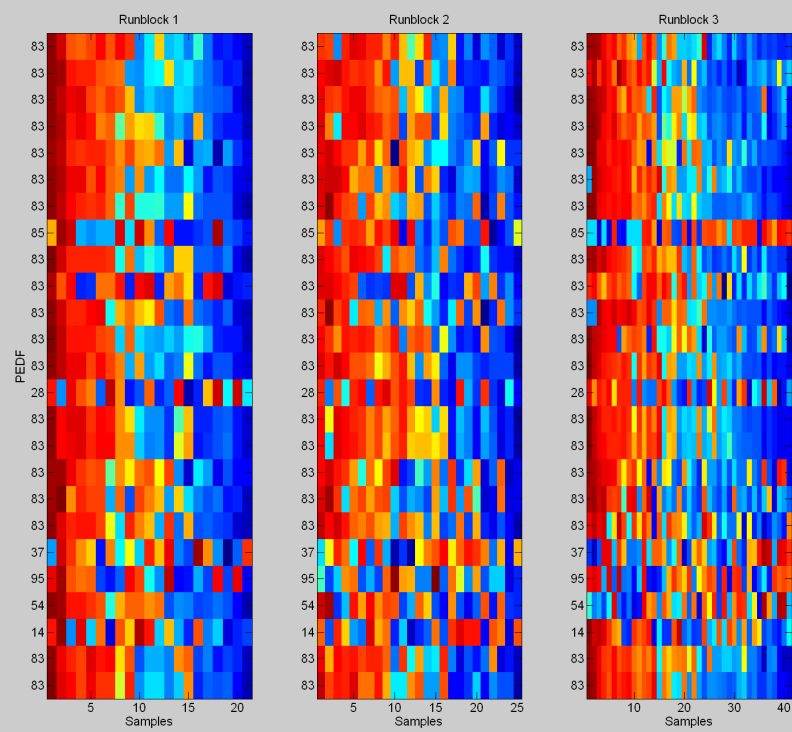

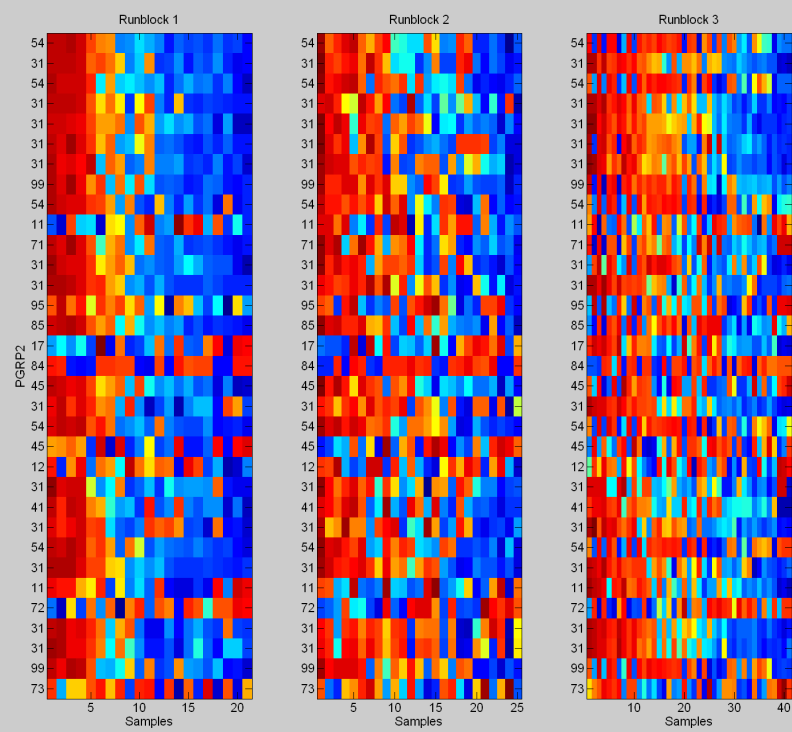

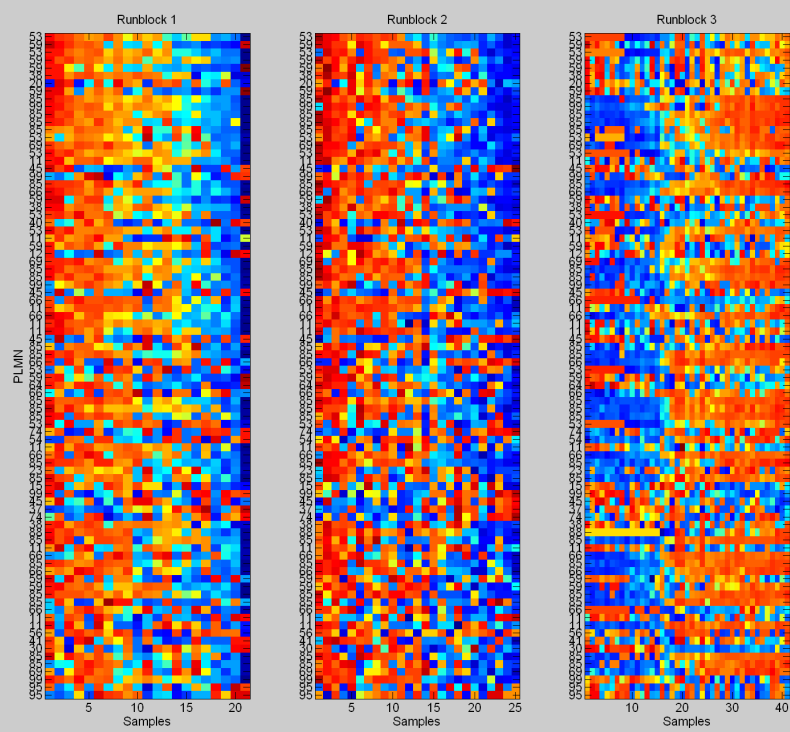

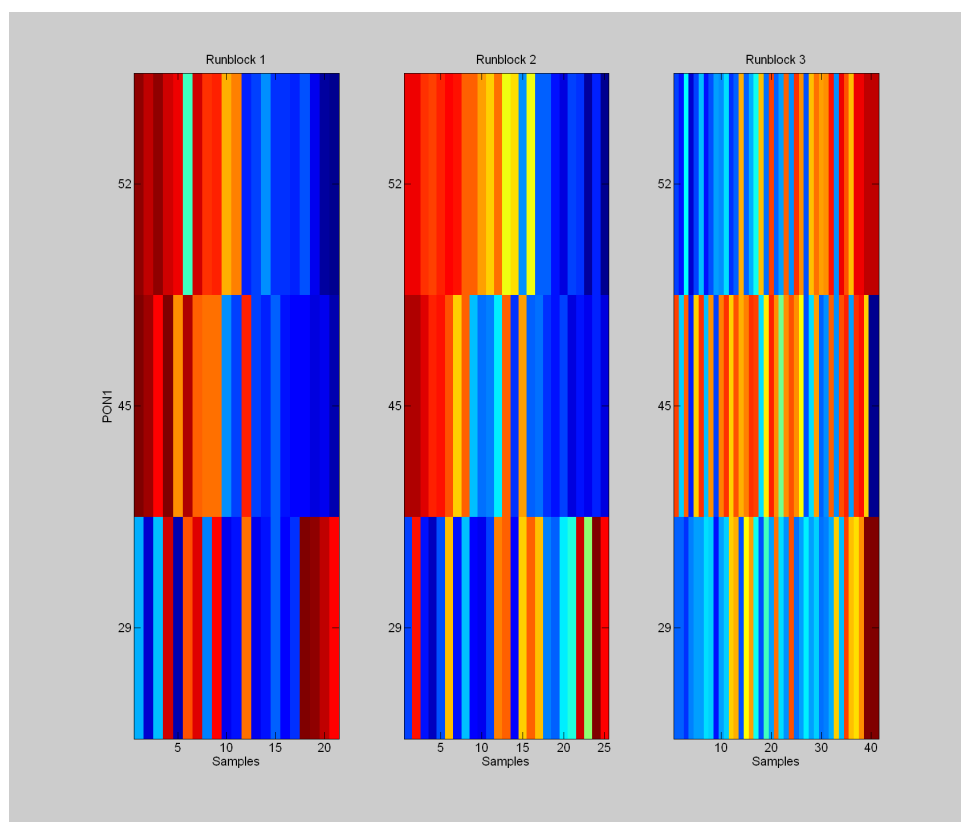

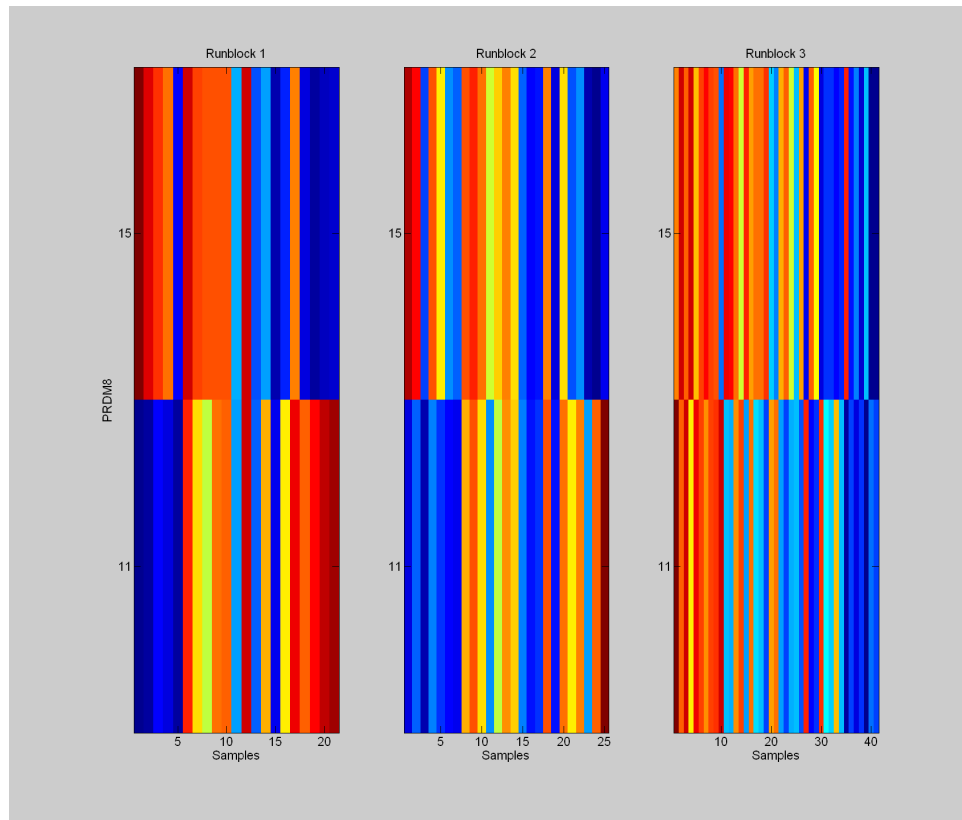

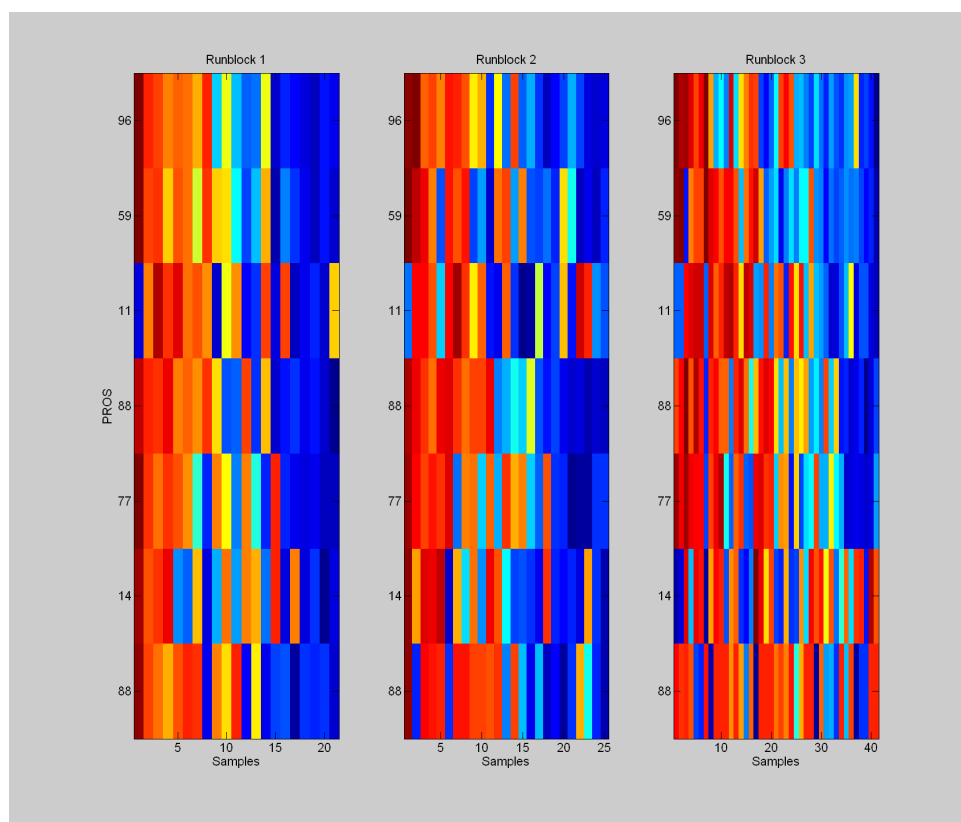

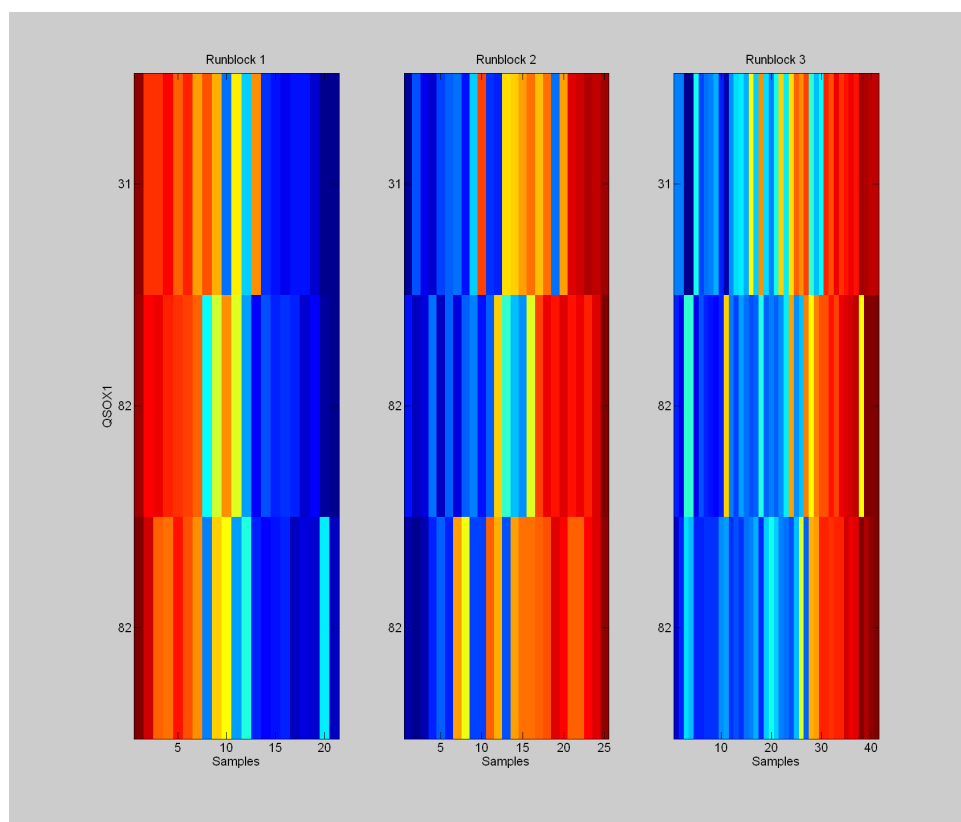

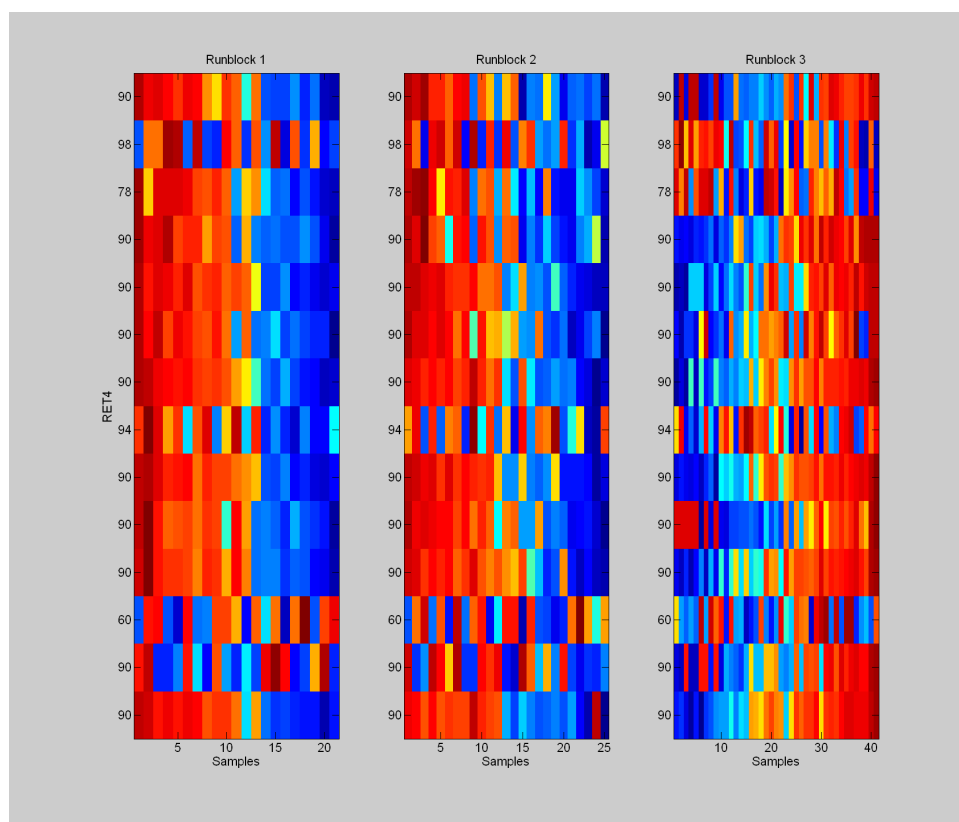

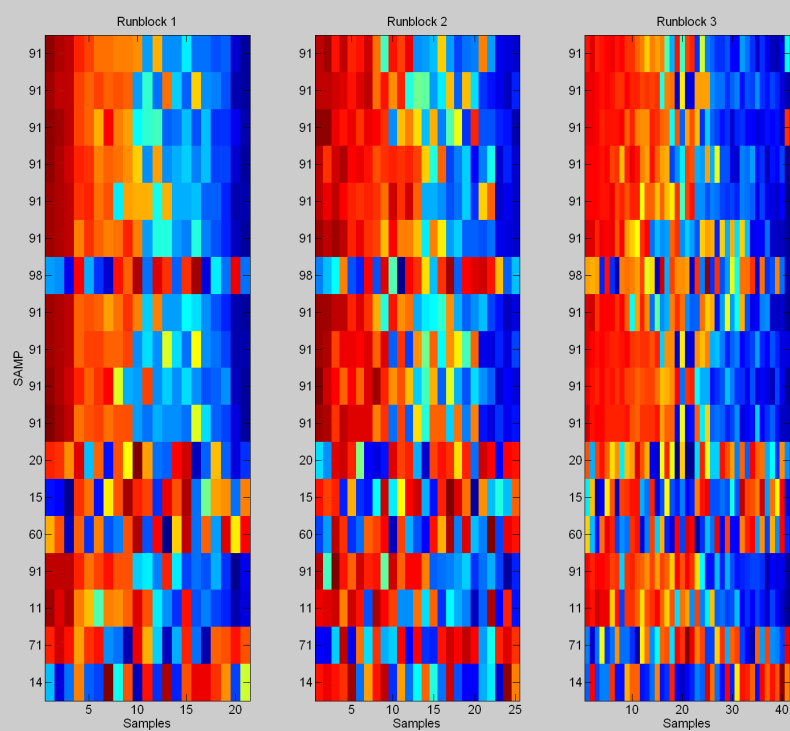

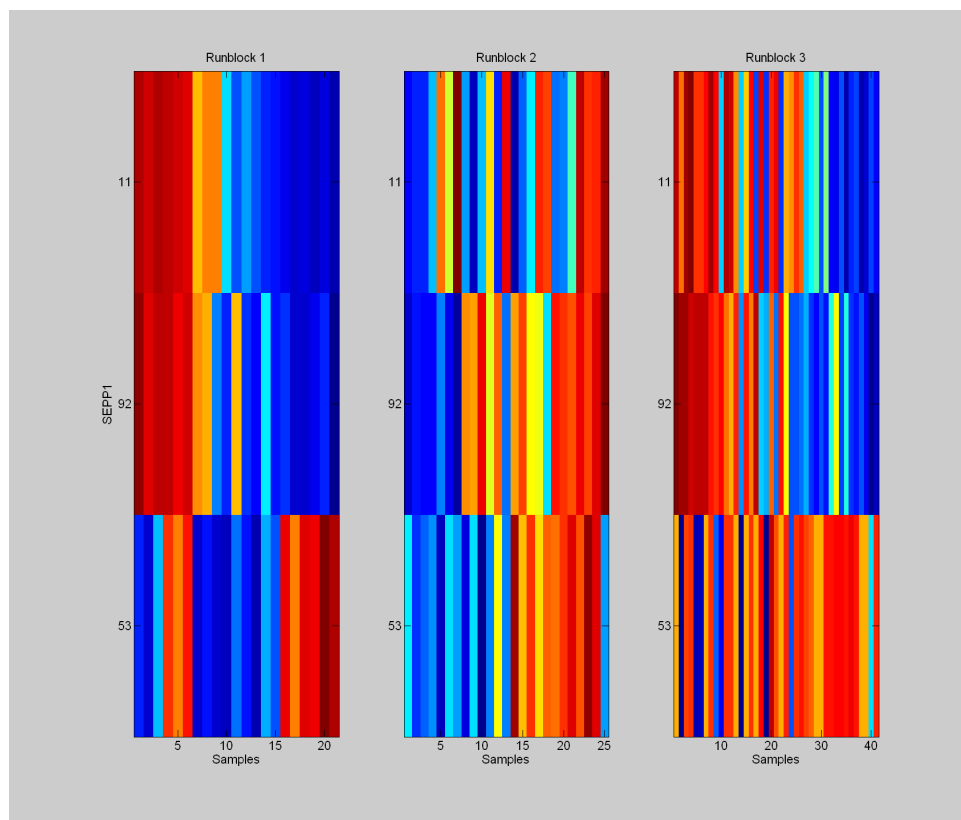

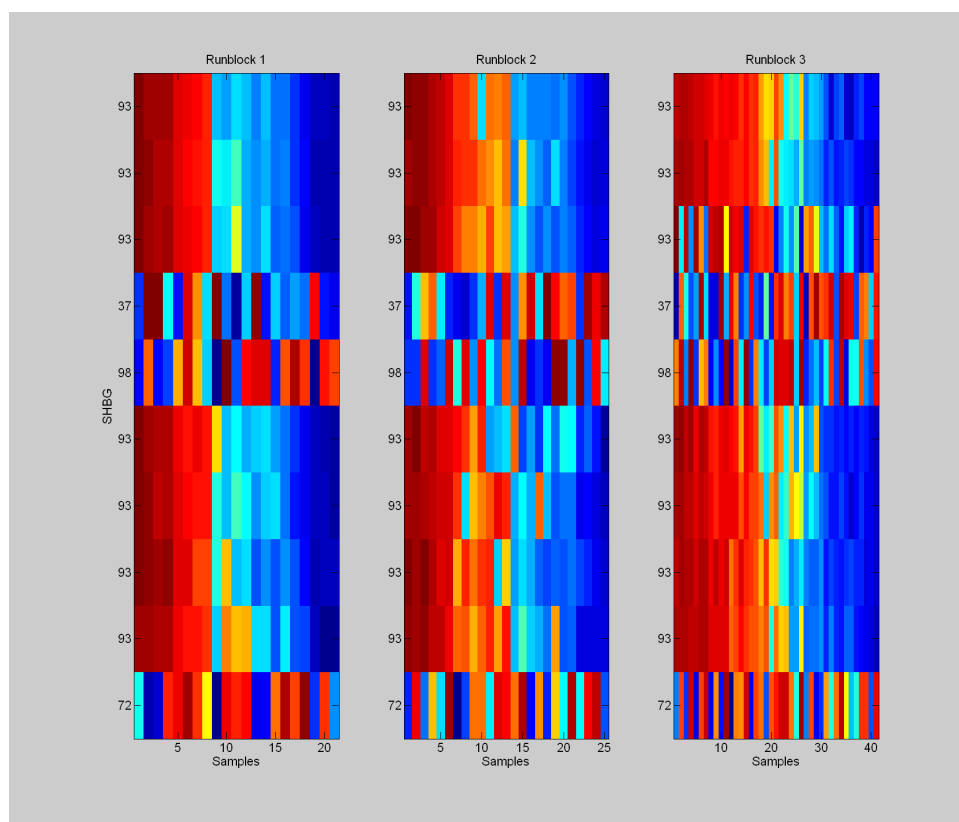

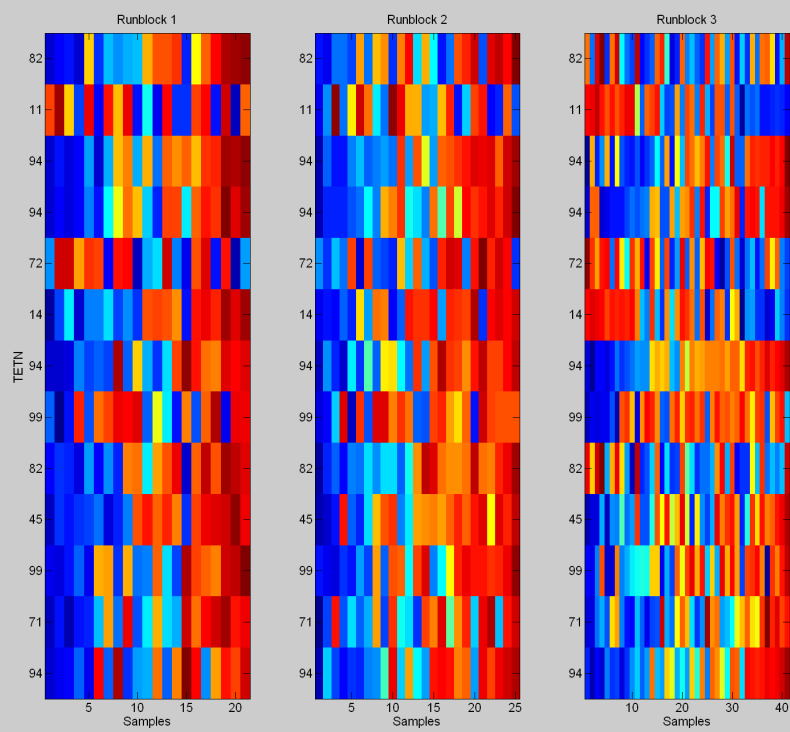

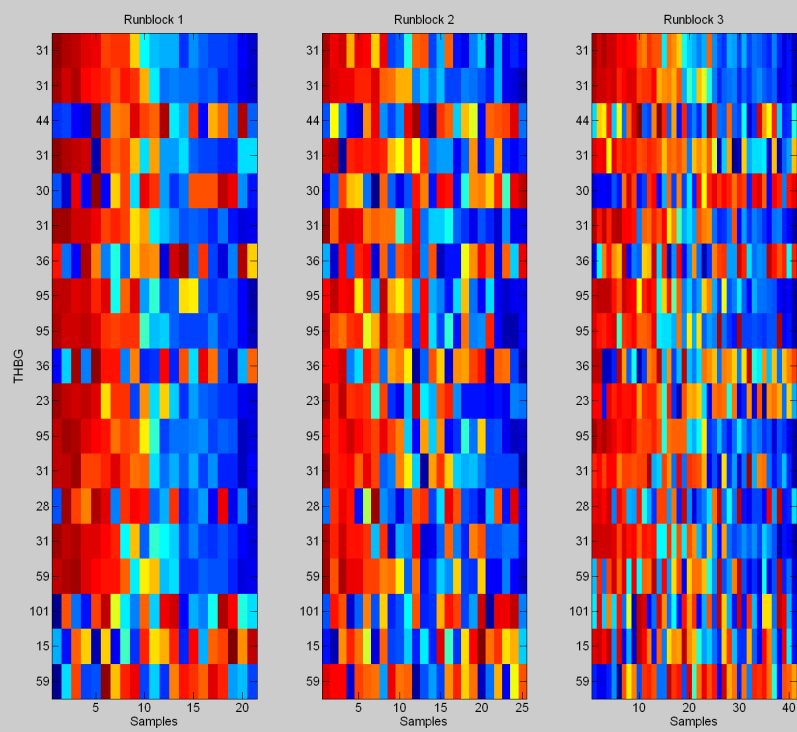

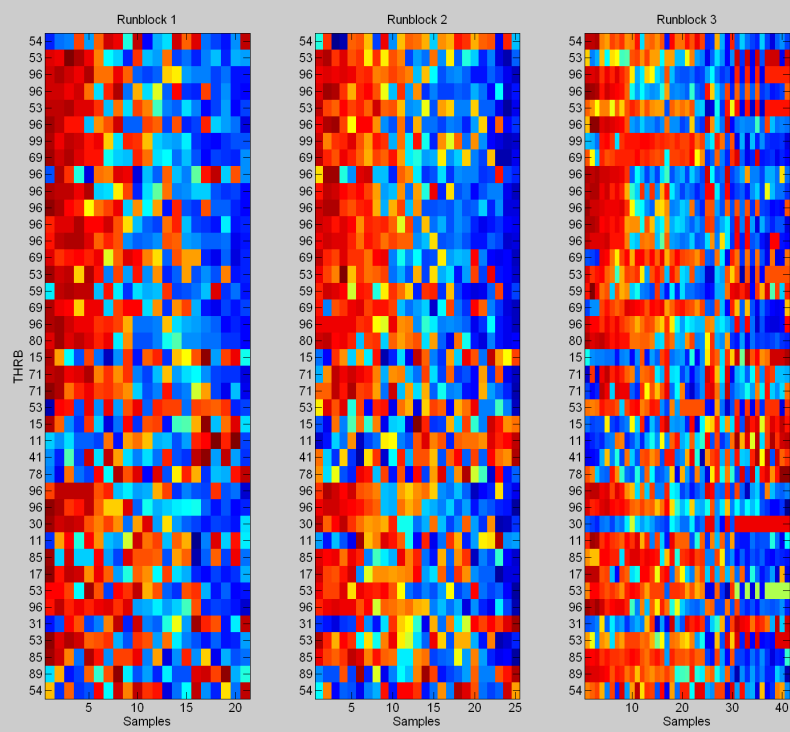

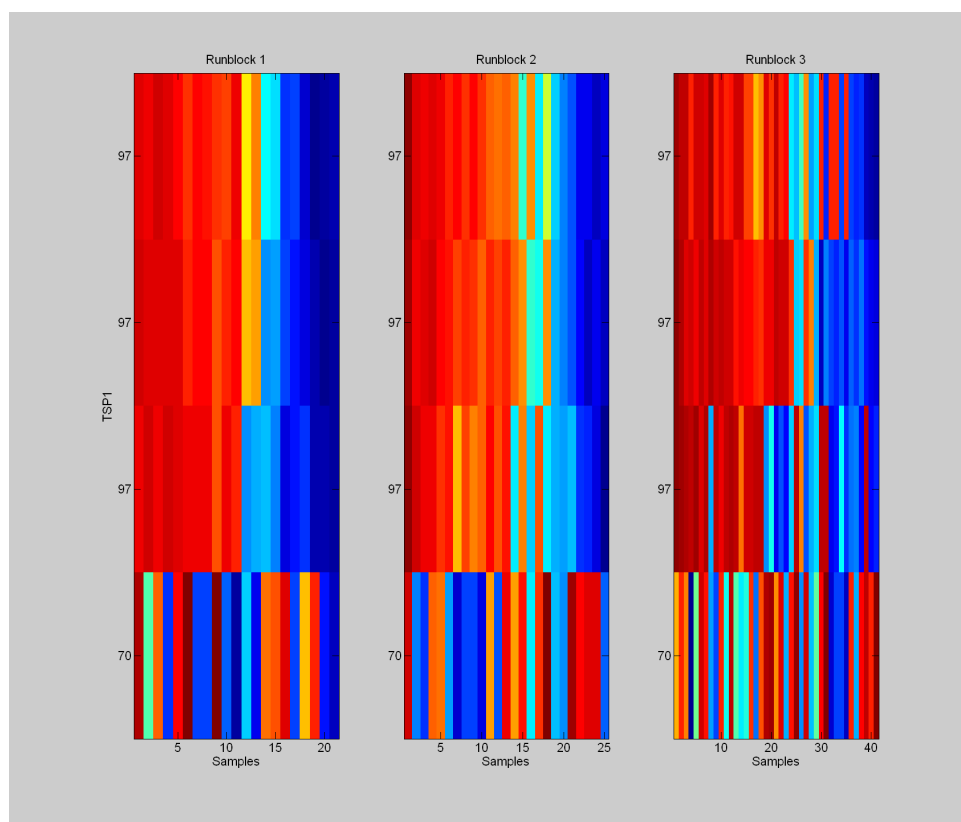

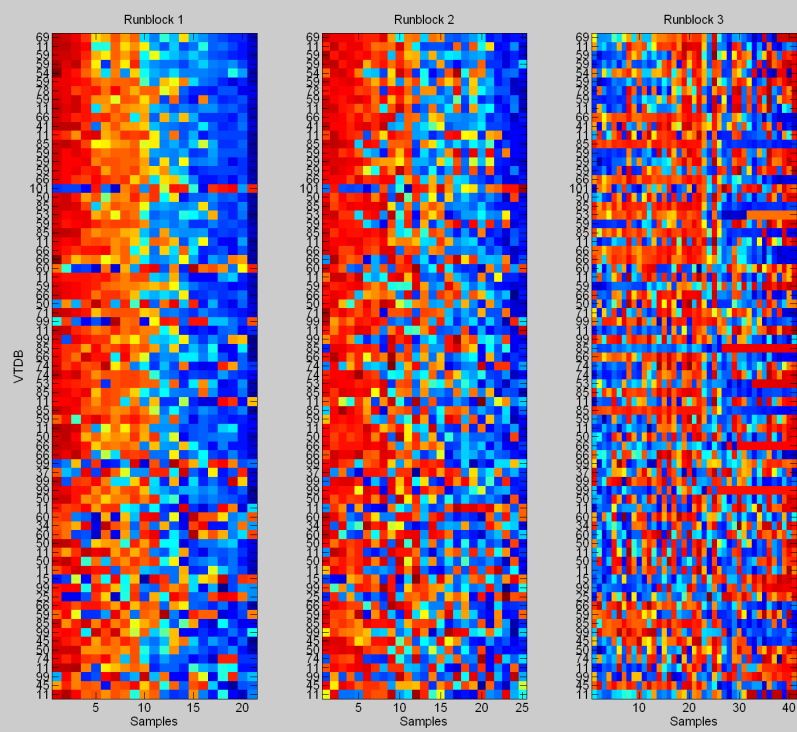

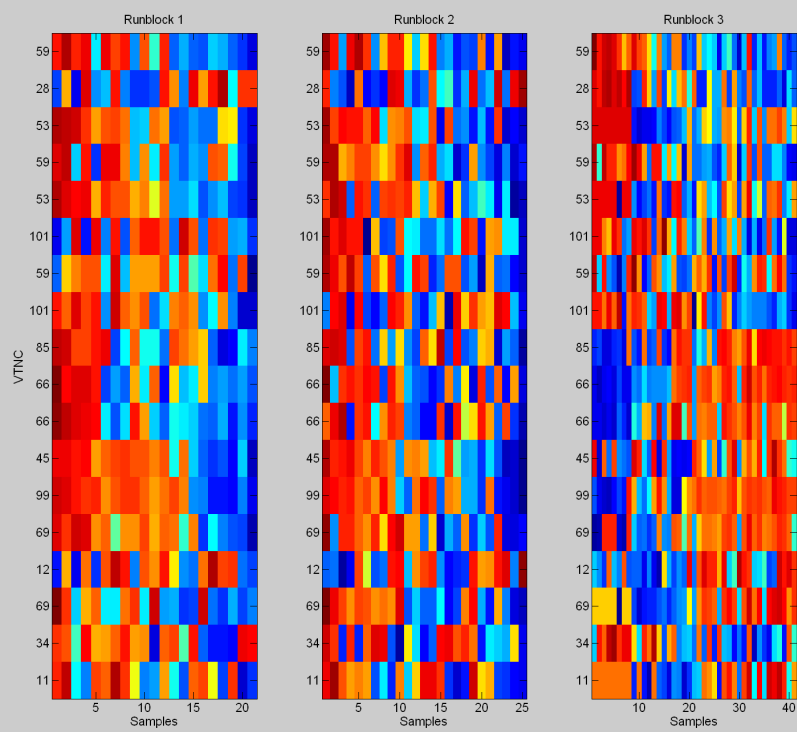

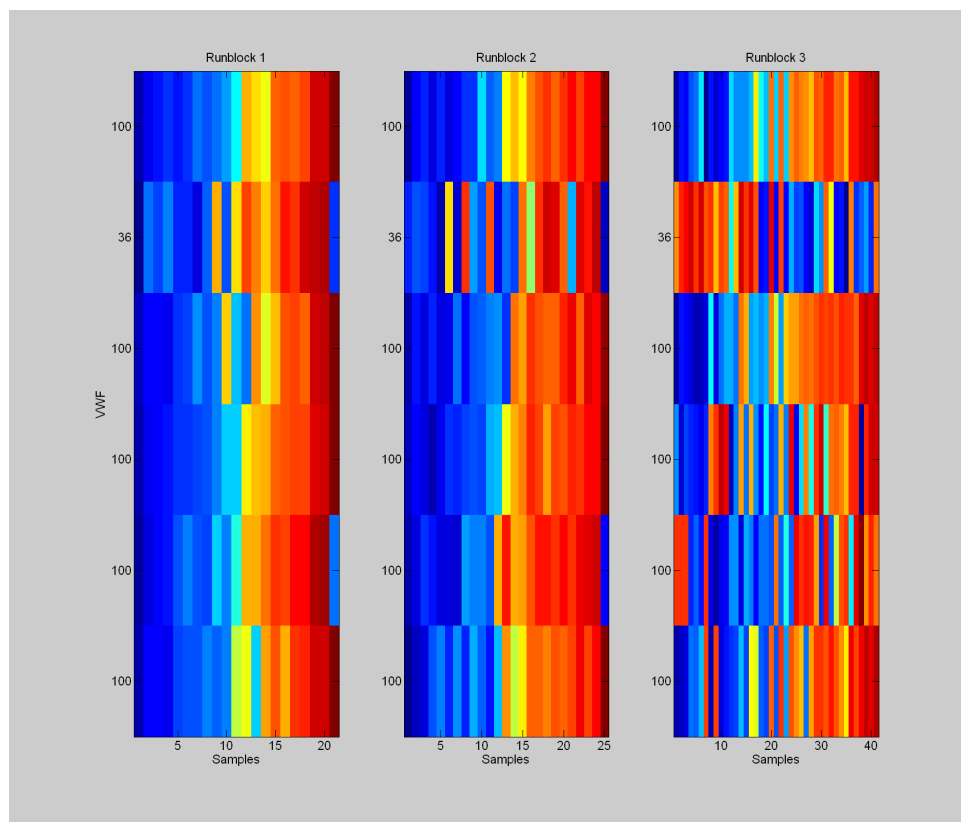

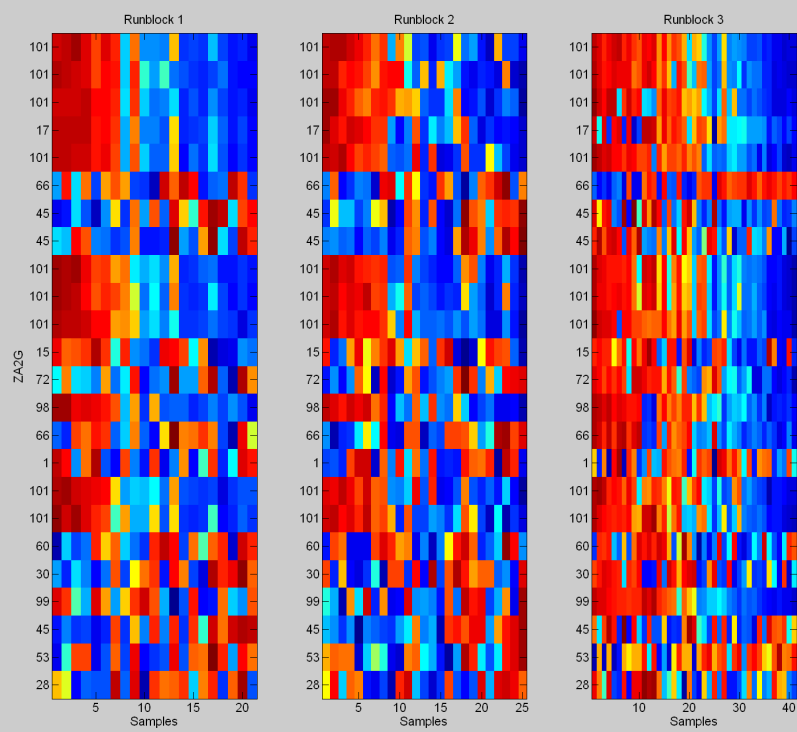

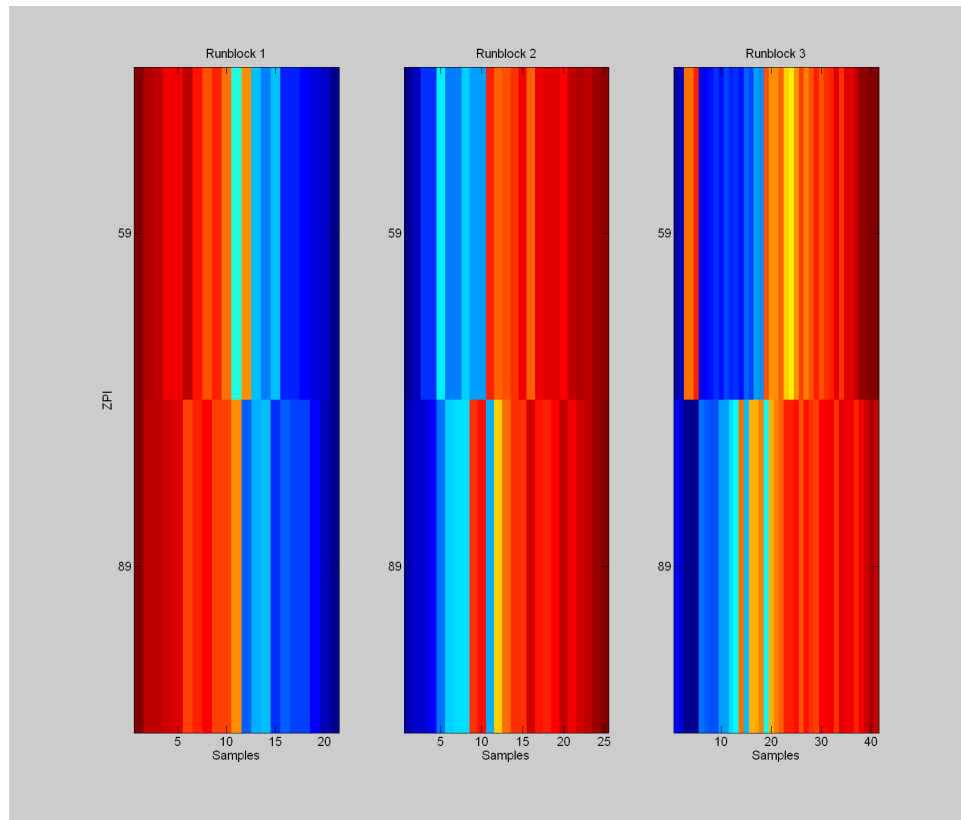

Supplement: Additional file 2 — Protein level, sorted by expression. Heatmaps for all identified isotope groups. The rows are sorted according from highest to lowest expression level and the columns are sorted so that the first principal component is increasing. Each isotope group is associated with a single metaprotein. Those metaproteins are labeled on the y-axis. [file 1471-2105-13-74-S2.pdf]
