# Supplementary material for: Metaprotein expression modeling for label-free quantitative proteomics
Source: BMC Bioinformatics. 2012 May 4;13:74. doi: 10.1186/1471-2105-13-74 (PMC3436780; doi:10.1186/1471-2105-13-74)
Supplement: Additional file 3 — Analysis of spike-in data. Estimates of the protein levels of spiked in samples from the Super Hirn experiment. Also included are the results of one of the label substitution experiments. [file 1471-2105-13-74-S3.pdf]

Supplemental

E-mail:

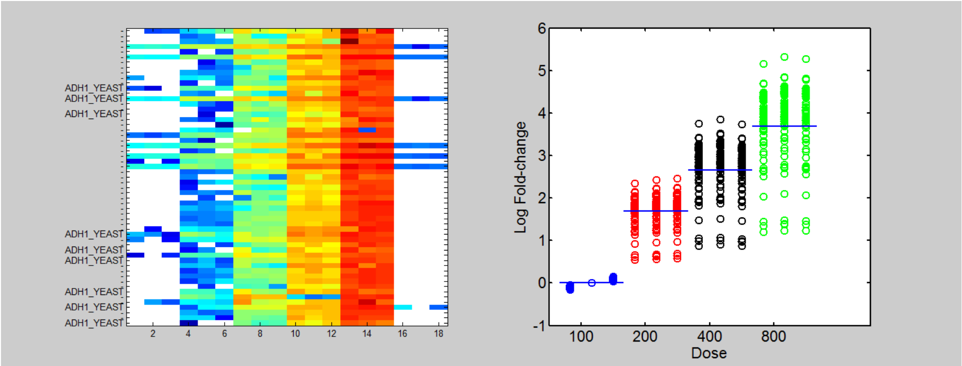

Figure 1: The left figure shows a heatmap of all of the isotope groups in the data set that are associated with the metaprotein ADH1_YEAST. The labeling on the y-axis indicates the original identification. Each row in each heatmap has been standardized to have mean zero and standard deviation one. Red is a relatively high level of expression and blue a low level of expression. The white color corresponds to missing data. The right figure shows the factor scores by group. This vector represents the estimated fold change based on meta-protein expression level and factor loadings. Each different spot is the estimated fold change associated with one of the isotope groups pictured in the heatmap on the right. We note that typical techniques for estimating fold change of a protein from a collection of isotope groups (such as median (maxQuant) or mean would be usable in this context. The lines in the scatterplot show the average estimated log fold change across all replicates and all isotope groups.

*To whom correspondence should be addressed

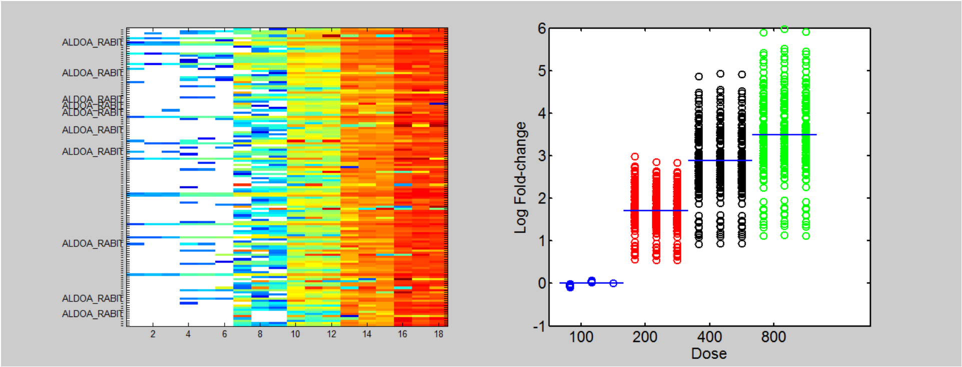

Figure 2: The left figure shows a heatmap of all of the isotope groups in the data set that are associated with the metaprotein ALDOA_RABIT. The labeling on the y-axis indicates the original identification. Each row in each heatmap has been standardized to have mean zero and standard deviation one. Red is a relatively high level of expression and blue a low level of expression. The white color corresponds to missing data. The right figure shows the factor scores by group. This vector represents the estimated fold change based on meta-protein expression level and factor loadings. Each different spot is the estimated fold change associated with one of the isotope groups pictured in the heatmap on the right. We note that typical techniques for estimating fold change of a protein from a collection of isotope groups (such as median (maxQuant) or mean would be usable in this context. The lines in the scatterplot show the average estimated log fold change across all replicates and all isotope groups.

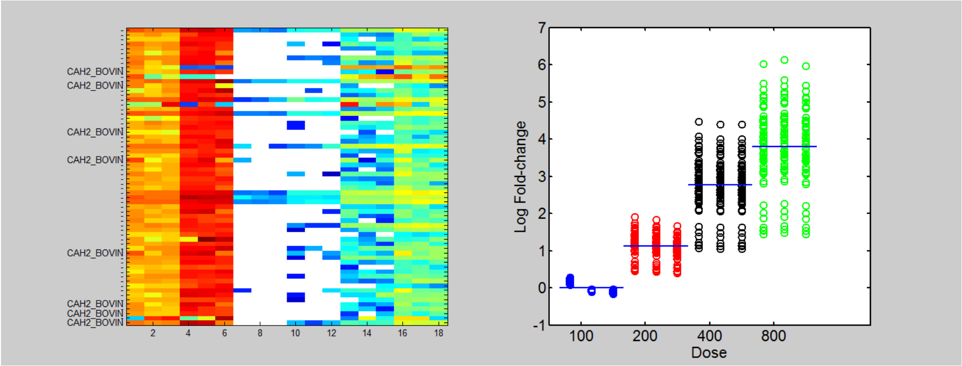

Figure 3: The left figure shows a heatmap of all of the isotope groups in the data set that are associated with the metaprotein CAH2_BOVIN. The labeling on the y-axis indicates the original identification. Each row in each heatmap has been standardized to have mean zero and standard deviation one. Red is a relatively high level of expression and blue a low level of expression. The white color corresponds to missing data. The right figure shows the factor scores by group. This vector represents the estimated fold change based on meta-protein expression level and factor loadings. Each different spot is the estimated fold change associated with one of the isotope groups pictured in the heatmap on the right. We note that typical techniques for estimating fold change of a protein from a collection of isotope groups (such as median (maxQuant) or mean would be usable in this context. The lines in the scatterplot show the average estimated log fold change across all replicates and all isotope groups.

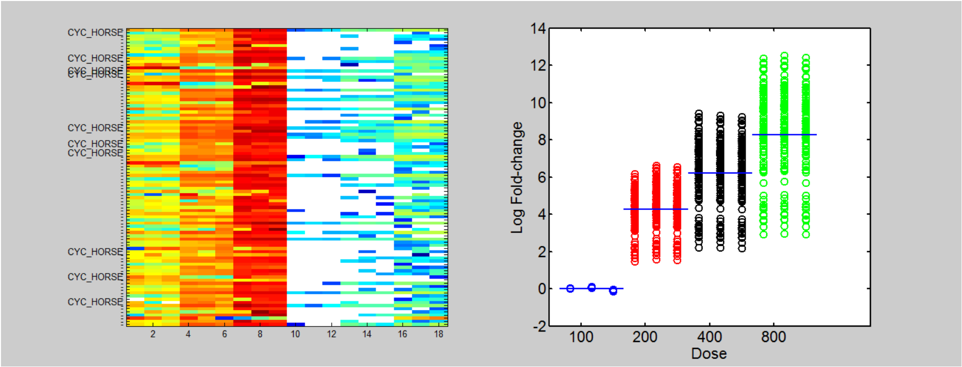

Figure 4: The left figure shows a heatmap of all of the isotope groups in the data set that are associated with the metaprotein CYC_HORSE. The labeling on the y-axis indicates the original identification. Each row in each heatmap has been standardized to have mean zero and standard deviation one. Red is a relatively high level of expression and blue a low level of expression. The white color corresponds to missing data. The right figure shows the factor scores by group. This vector represents the estimated fold change based on meta-protein expression level and factor loadings. Each different spot is the estimated fold change associated with one of the isotope groups pictured in the heatmap on the right. We note that typical techniques for estimating fold change of a protein from a collection of isotope groups (such as median (maxQuant) or mean would be usable in this context. The lines in the scatterplot show the average estimated log fold change across all replicates and all isotope groups.

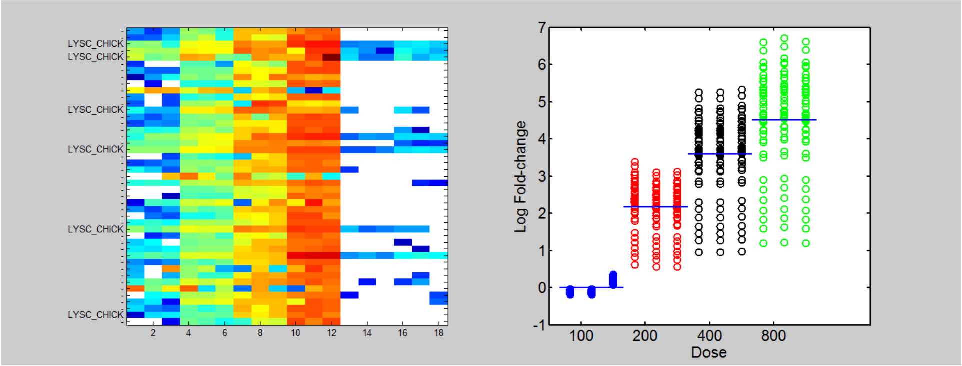

Figure 5: The left figure shows a heatmap of all of the isotope groups in the data set that are associated with the metaprotein LYSC_CHICK. The labeling on the y-axis indicates the original identification. Each row in each heatmap has been standardized to have mean zero and standard deviation one. Red is a relatively high level of expression and blue a low level of expression. The white color corresponds to missing data. The right figure shows the factor scores by group. This vector represents the estimated fold change based on meta-protein expression level and factor loadings. Each different spot is the estimated fold change associated with one of the isotope groups pictured in the heatmap on the right. We note that typical techniques for estimating fold change of a protein from a collection of isotope groups (such as median (maxQuant) or mean would be usable in this context. The lines in the scatterplot show the average estimated log fold change across all replicates and all isotope groups.

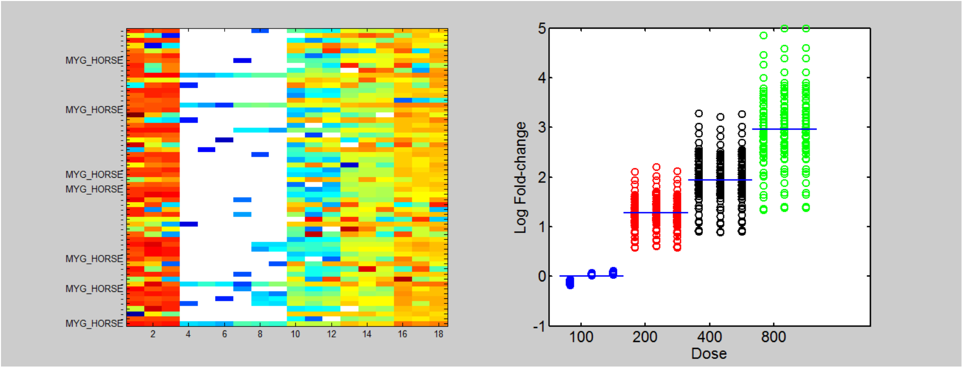

Figure 6: The left figure shows a heatmap of all of the isotope groups in the data set that are associated with the metaprotein MYG_HORSE. The labeling on the y-axis indicates the original identification. Each row in each heatmap has been standardized to have mean zero and standard deviation one. Red is a relatively high level of expression and blue a low level of expression. The white color corresponds to missing data. The right figure shows the factor scores by group. This vector represents the estimated fold change based on meta-protein expression level and factor loadings. Each different spot is the estimated fold change associated with one of the isotope groups pictured in the heatmap on the right. We note that typical techniques for estimating fold change of a protein from a collection of isotope groups (such as median (maxQuant) or mean would be usable in this context. The lines in the scatterplot show the average estimated log fold change across all replicates and all isotope groups.

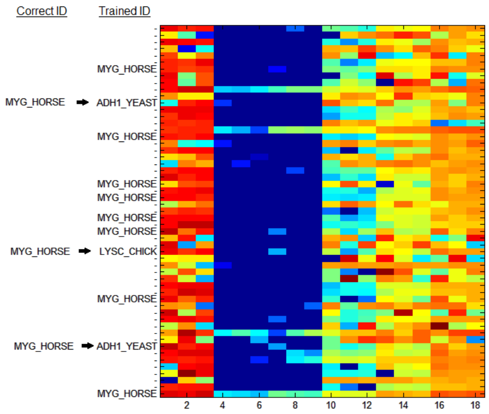

Figure 7: Model performance in the face of incorrect identifications. We permuted the identities of 10% of the isotope groups in the data and tested the ability of our model to recapitulate the correct identifications of the incorrectly identified features. This heatmap shows the MYG_HORSE metaprotein for one such permutation. Even in the face of 3 incorrect identifications, all of the identified features in the metaprotein were originally identified as originating from MYG_HORSE.
